# Ultrasonicated Atlantic herring side streams as source of multifunctional bioactive and bioavailable peptides

**DOI:** 10.1038/s41538-025-00388-w

**Published:** 2025-02-22

**Authors:** Gilda Aiello, Janna Cropotova, Kristine Kvangarsnes, Lorenza d’Adduzio, Melissa Fanzaga, Carlotta Bollati, Giovanna Boschin, Gabriella Roda, Carmen Lammi

**Affiliations:** 1 Department of Human Science and Quality of Life Promotion, Telematic University San Raffaele, Rome, Italy; 2 Department of Biological Sciences Ålesund, Norwegian University of Science and Technology (NTNU), Ålesund, Norway; 3 Department of Pharmaceutical Sciences, University of Milan, Milan, Italy

**Keywords:** Health care, Peptides

## Abstract

This study demonstrates the effectiveness of ultrasonication, as a pre-treatment technology, coupled to enzymatic hydrolysis of herring side streams, yielding multifunctional peptide mixtures with antioxidant, hypotensive (ACE inhibitory activity), and hypoglycemic (DPP-IV inhibitory and GLP-1 enhancer activity) properties. The ultrasound pre-treatment modulates the biological activity of the hydrolysates, enhancing certain bioactive properties (antioxidant, ACE inhibitory, and GLP-1 enhancer activities, respectively) while reducing others (DPP-IV inhibitory activity). The study also highlights the importance of simulating gastrointestinal digestion and using Caco-2 cells to assess the bioaccessibility, intestinal bioavailability, and metabolic resistance of herring peptides. These findings support the use of ultrasonication and enzymatic hydrolysis in obtaining multifunctional bioactive peptide mixture for the prevention of metabolic syndrome. Results clearly suggest that this approach represent sustainable solutions in food science and technology, since it allowed us to obtain a bioactive mixture of peptides starting from fish by-products pre-treated with green methodologies.

## Introduction

The utilization of Atlantic herring (*Clupea harengus*) side streams for producing protein hydrolysates has gained significant attention in recent years^1^. A review on the processing of functional proteins or peptides derived from fish by-products and their industrial applications. These hydrolysates, akin to those derived from other fish sources, represent rich reservoirs of bioactive peptides endowed with diverse health-promoting properties. Bioactive peptides sourced from herring protein hydrolysates have been particularly characterized for their ACE (angiotensin-converting enzyme) inhibitory activity, pivotal in regulating blood pressure by impeding vasoconstriction. Such functionality holds promise for mitigating hypertension and preventing associated cardiovascular disorders^2,3^. Additionally, peptides from herring protein hydrolysates have exhibited potent anti-inflammatory and antioxidant effects, presenting avenues for alleviating inflammation, a hallmark of numerous chronic conditions such as arthritis, diabetes, and cardiovascular disorders^4^. Interestingly, Durand and colleagues identified herring-derived bioactive peptides isolated from herring milt, able to decrease the inducible nitric oxide synthase activation in the macrophage cells, demonstrating their in vitro anti-inflammatory effect and making them good candidates for the prevention of the metabolic syndrome^5^. Further studies were conducted to validate their in-vivo bioactivity, demonstrating the ability of these herring bioactive hydrolysates to improve glucose metabolism and microbiota composition in presence of diet-induced obesity in mice models^6^.

The multifaceted health benefits offered by herring protein hydrolysates encompass cardiovascular support, anti-inflammatory effects, antioxidant prowess, and immune modulation^7,8^, attracting, therefore, considerable interest in the food and pharmaceutical industries. Ultrasonication, characterized by the application of high-frequency waves, has emerged as a novel technique for enhancing the extraction and bioactivity of peptides from various protein sources, including herring^9^. The application of ultrasound technology in an environmentally friendly manner, typically involving the use of lower energy consumption, non-toxic solvents, and sustainable processing methods. When applied to the recovery of valuable compounds from fish-side streams, green ultrasonication as a pre-treatment offers several important benefits. Indeed, first, by making the extraction process more sustainable, it reduces the generation of hazardous waste, lowering the environmental footprint of the whole process. This is particularly important in industries such as fisheries, where waste management and environmental sustainability are significant concerns. Ultrasonication improves the sustainability of fish processing operations by utilizing fish side streams that would otherwise be used for low value applications such as fish meal or discarded as waste. By extracting valuable compounds such as peptides, from fish side streams, it helps maximize resource utilization and reduce waste generation. Moreover, ultrasonication facilitates the breakdown of cell walls and the release of bioactive components, resulting in the efficient extraction of proteins, peptides, and other desirable compounds such as fish oil rich in polyunsaturated fatty acids^9^.

Despite the significant advancements in the field of herring peptides and ultrasonication, several challenges and opportunities remain to be addressed. Further research is needed to optimize ultrasonication parameters, such as frequency, power intensity, and processing time, to maximize the extraction efficiency and bioactivity of herring peptides. Moreover, a comprehensive characterization of herring peptide hydrolysates is essential to elucidate their structure-function relationships and mechanisms of action. Additionally, studies investigating the stability, bioavailability, and safety of herring peptides are warranted to ensure their efficacy and suitability for human consumption^10^.

Based on these considerations, recently, exploiting ultrasonication technique, herring hydrolysates have been produced and characterized (unpublished data). In the framework of research aimed at promoting the health benefits of herring hydrolysates, the main objectives of this study are to evaluate the impact of ultrasonication on the peptidomic profile and bioactivity modulation of hydrolysate fractions enriched in medium- and small-sized peptides, and to assess the bioaccessibility of these peptides under simulated gastrointestinal digestion (SGID). Additionally, this study aims to investigate the biostability of herring peptides during digestion and their bio-absorption potential across the Caco-2 cell monolayer. The results of this study, conducted at biochemical and cellular level, provide promising data suggesting that herring peptides produced by ultrasonication and enzymatic hydrolysis could potentially be used as functional ingredients. In this context, these results will be useful for carrying out the in vivo *proof of concept* on animal model to confirm their health promoting activity.

## Results and discussion

### Atlantic herring hydrolysates characterization

Since its well known that small peptides (<3 kDa) are those that show greater biological activity^11^, all the HH samples were ultrafiltrate using membranes with a 3-kDa molecular weight cut-off. The recovered peptides (HH ctrl, HH40, and HH80) have been, then, investigated to study their composition and, consequently, their biological properties. Initial peptidomic analyses have confirmed the presence of low molecular weight peptides in the samples, supporting the hypothesis that these peptides possess enhanced resistance to enzymatic breakdown during gastrointestinal transit. Furthermore, their small size facilitates intact absorption into the bloodstream, allowing efficient transport to target organs. Conversely, high molecular weight peptides may undergo degradation by digestive enzymes within the gastrointestinal tract, often resulting in structural alterations that diminish the peptide’s activity or render it inactive. The broad spectrum of structural diversity among these peptides elucidates the extensive array of functional activities they exhibit. Indeed, multifunctional peptides represent an emerging area with numerous potential applications thanks to their capacity to impart more than one physiological outcome by affecting different targets. Since the functionality of peptides is influenced by their amino acid composition, sequence, and length^12^, the first step of the work was to investigate the three HHs composition by peptidomics. Furthermore, since ultrasound treatment has been reported to impact the release of novel bioactive peptides with several health-promoting properties^13^, becomes important to evaluate the effect of ultrasonication as pre-treatment prior to enzymatic hydrolysis on the herring hydrolysates chemical composition and biological activities.

### Effect of ultrasonication on herring hydrolysates evaluated by peptidomics

**Fig. 1:**
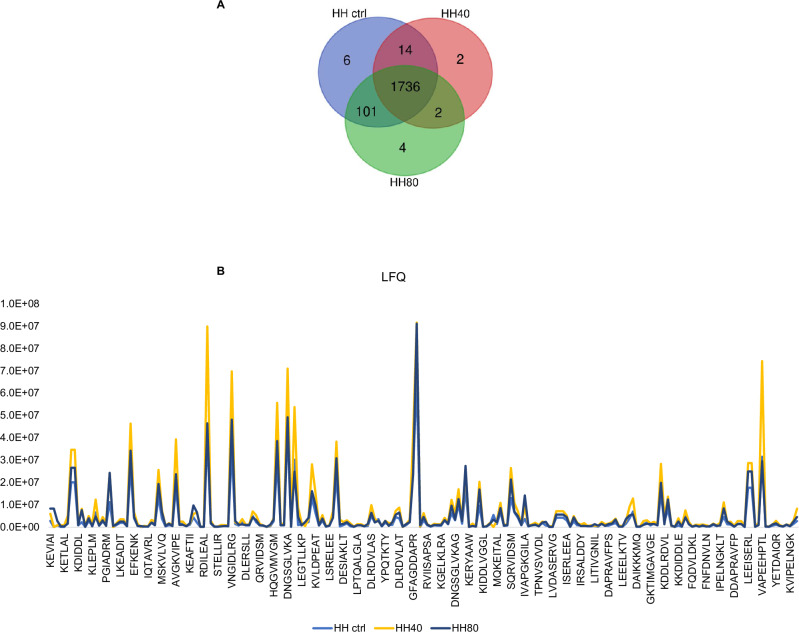
Peptidomic analysis of herring hydrolysates. The number of total peptides (<3 kDa MW) identified in herring protein hydrolysate after different ultrasonic power treatments (**A**). The trend of label free quantification (LFQ) intensity of HH, HH40, and HH80 peptides within the molecular weight range of 600 < MW < 1000 Da (**B**).

The impact of ultrasonication as pre-treatment prior to enzymatic hydrolysis on the recovered peptide mixtures is contingent upon various factors, including processing conditions, protein source, and desired product properties. Ultrasonication can improve the hydrolysis process by facilitating the rupture of cell walls of the fish flesh and further enhancing mass transfer between protein molecules, the solvent, and enzymes, thereby reducing potentially processing times and increasing the peptide yields and quality. Therefore, optimizing ultrasonication parameters is crucial to maximize benefits while mitigating drawbacks. In this study, we evaluated the effect of ultrasonication as pre-treatment before to enzymatic hydrolysis on the chemical composition of herring hydrolysate using LC-MS analysis. Figure 1 shows the number of peptides with MW < 3 kDa, identified under different ultrasonication conditions. A total of 1857, 1754, and 1843 peptides were identified in HH ctrl, HH40, and HH80, respectively (Fig. 1A). As underlined by Venn diagrams, 85% of identified peptides are common to the HH ctrl, HH40, and HH80, demonstrating that no substantial changes in peptide composition were observed across the ultrasonication-tested conditions. Supplementary Table S1 reports the identified peptide sequences in each sample with the parental protein IDs.

The analysis of peptide compositions in HH ctrl, HH40, and HH80 reveals that ultrasonication has minimal impact on altering these compositions. Nonetheless, ultrasonication markedly enhances the intensity of the amount released peptide, as shown in Fig. 1B. This increase in intensity is attributed to the mechanical forces induced by ultrasonic waves, which disrupt protein structures and make them more susceptible to enzymatic breakdown. Consequently, this process facilitates the liberation of additional peptides into the solution. Figure 1B distinctly illustrates the trend of LFQ (label-free quantification) intensity of ultrasonicated herring peptides within the molecular weight range of 600 < MW < 1000 Da. Most peptides identified by LC-MS analysis belong to myosin heavy chain, fast skeletal muscle; actin, alpha skeletal muscle and tropomyosin alpha-1 chain identified by 458, 383, and 90 unique peptides, respectively. Peptides derived from collagen alpha-1(XXVII) chain B and collagen alpha-1(XXVII) chain A, were also detected in HH ctrl, HH40 and HH80. Peptides derived from fish muscle proteins, collagen, and gelatin have been recognized for potential use in medical, pharmaceutical, and cosmeceutical industries due to their wide range of bioactivities^14^. Specifically, TEAPLNPK (FC_HH40/HHctrl_ = 9.6 and FC_HH80/HHctrl_ = 5.0) and MDAIKKK (FC_HH40/HHctrl_ = 11.4 and FC_HH80/HHctrl_ = 4.2), belonging to actin and tropomyosin alpha-1 chain, respectively, show high fold changes, particularly under the HH40 condition. These results suggest that ultrasound at this intensity might have triggered significant structural changes or enhanced peptide expression. Such a strong increase could be due to increased protein fragmentation or increased solubility of these peptides under the HH40 ultrasound treatment. SERVGLLH, LGSIAVIL, and IKSKIQLE exhibit fold changes across both ultrasound conditions, particularly in HH80/HHCtrl (9.1; 7.5; and 3.5, respectively). These peptides are also moderately responsive to HH40, suggesting they may be relatively stable but are still sensitive to the higher intensity of ultrasound treatment. Moreover, some peptides, such as RGILTL, NNRFASF, and RVIDSM, show relatively low responses under the HH80 condition compared to HH40, suggesting that while the contribution of HH80 relative to HHctrl is significant, the increase in ultrasound intensity from HH40 to HH80 does not lead to a further substantial impact on these peptides. This could indicate that these peptides either reach a plateau in terms of structural changes or solubilization at the moderate ultrasound intensity (HH40), or that the higher intensity (HH80) does not provide additional benefits beyond those achieved with HH40.

### Herring hydrolysates in vitro antioxidant activities

**Fig. 2:**
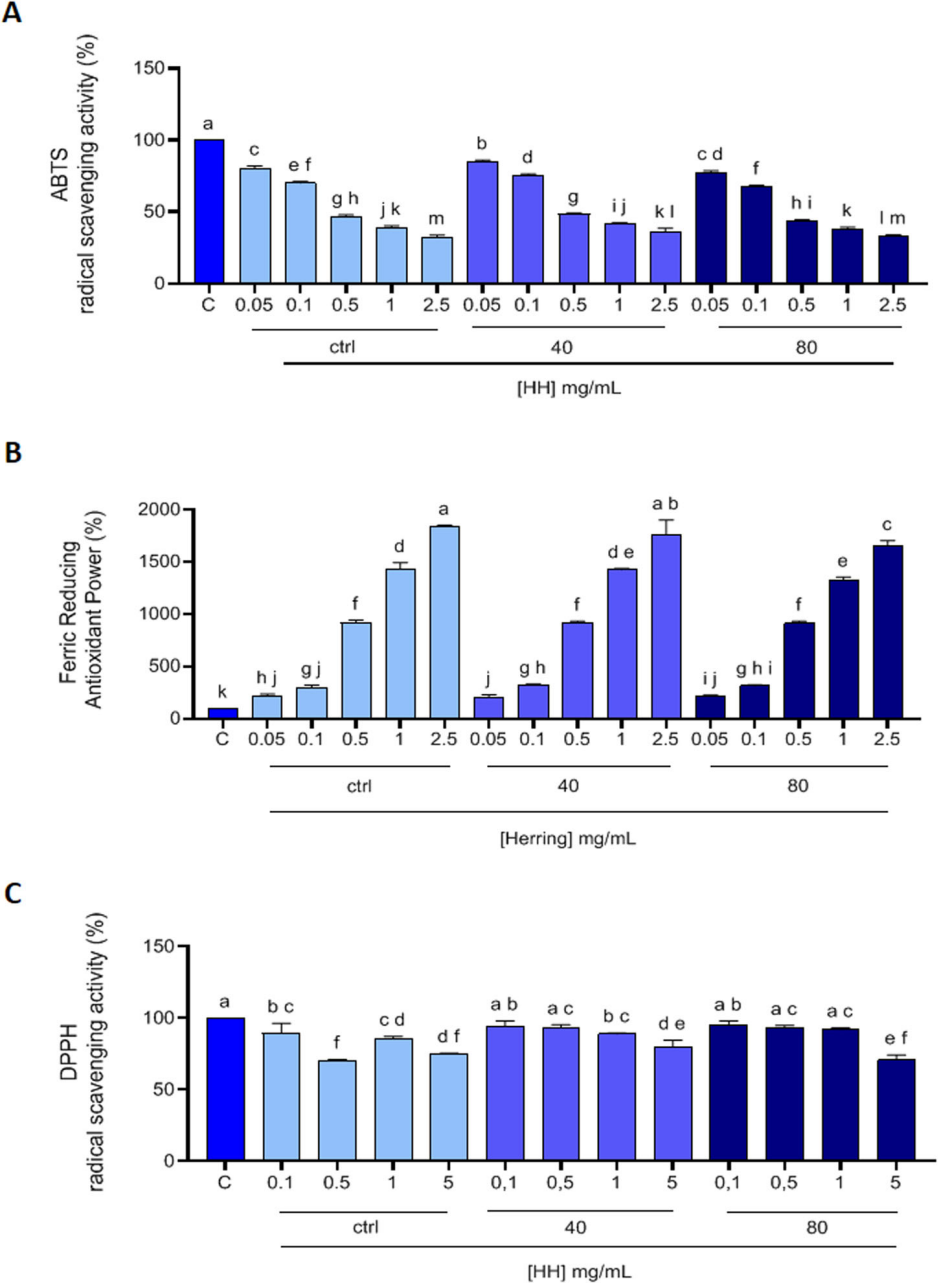
In vitro antioxidant power evaluation of the HHs ctrl, HH40, HH80. 2,2-Azino-bis-(3-ethylbenzothiazoline-6-sulfonic acid (ABTS) assay (**A**), ferric reducing antioxidant power (FRAP) assay (**B**), and 2,2-diphenyl-1-picrylhydrazyl (DPPH) assay (**C**). The data points represent the averages ± SD of three independent experiments performed in triplicate. All data sets were analyzed by One-way ANOVA followed by Tukey’s post-hoc test. C: control sample (H_2_O). Different lowercase letters indicate a significant difference (*p* < 0.05) between different concentrations.

Considering the very heterogeneous composition of the hydrolysates (Fig. 1B), their biological activities were evaluated. Since the HH compositions are very heterogeneous, and diversified peptide structures generally explain a wide range of functional activities^15^, we aimed to highlight the HH possible multifunctional activity. The in vitro antioxidant activity of HH ctrl, HH40, and HH80 were tested in the range of 0.05–2.5 mg/mL using the ABTS and the FRAP, while were tested in the range of 0.1–5.0 mg/mL for the DPPH assay. The HH ctrl decreased the ABTS radical up to 32.1 ± 1.6%; HH40 decreased the ABTS radical up to 36.4 ± 2.1% and HH80 decreased the ABTS radical up to 32.8 ± 1.2% (*p* < 0.0001, Fig. 2A). Figure 2B show that the HH ctrl increased the FRAP up to 1840 ± 13.1% at 2.5 mg/mL, HH40 increased the FRAP up to1764.0 ± 136.6% at 2.5 mg/mL; HH80 increased the FRAP up to 1652.0 ± 52.9% at the same tested concentration. Lastly, as shown by Fig. 2C, HH ctrl reduced the DPPH radical up to 75.2 ± 0.2%, HH40 reduced the DPPH radical up to 79.8 ± 4.9%, and HH80 reduced the DPPH radical up to 70.8 ± 3.4% at 5.0 mg/mL respectively (*p* < 0.0001, Fig. 2C). In ABTS and FRAP assays, the response was dose dependent. Detailed results are reported in Supplementary materials (Table S2).

Overall, the antioxidant activities measured by ABTS, FRAP, and DPPH assays reveal a dose-dependent response in most cases. Among the hydrolysates, HH40 generally exhibited the most balanced and potent antioxidant effects across all assays, suggesting that the conditions of ultrasound pre-treatment used for its production may enhance its bioactive potential. HH80 also displayed strong antioxidant properties, particularly in the DPPH assay.

### Characterization of antioxidant activity of HH at cellular level

**Fig. 3:**
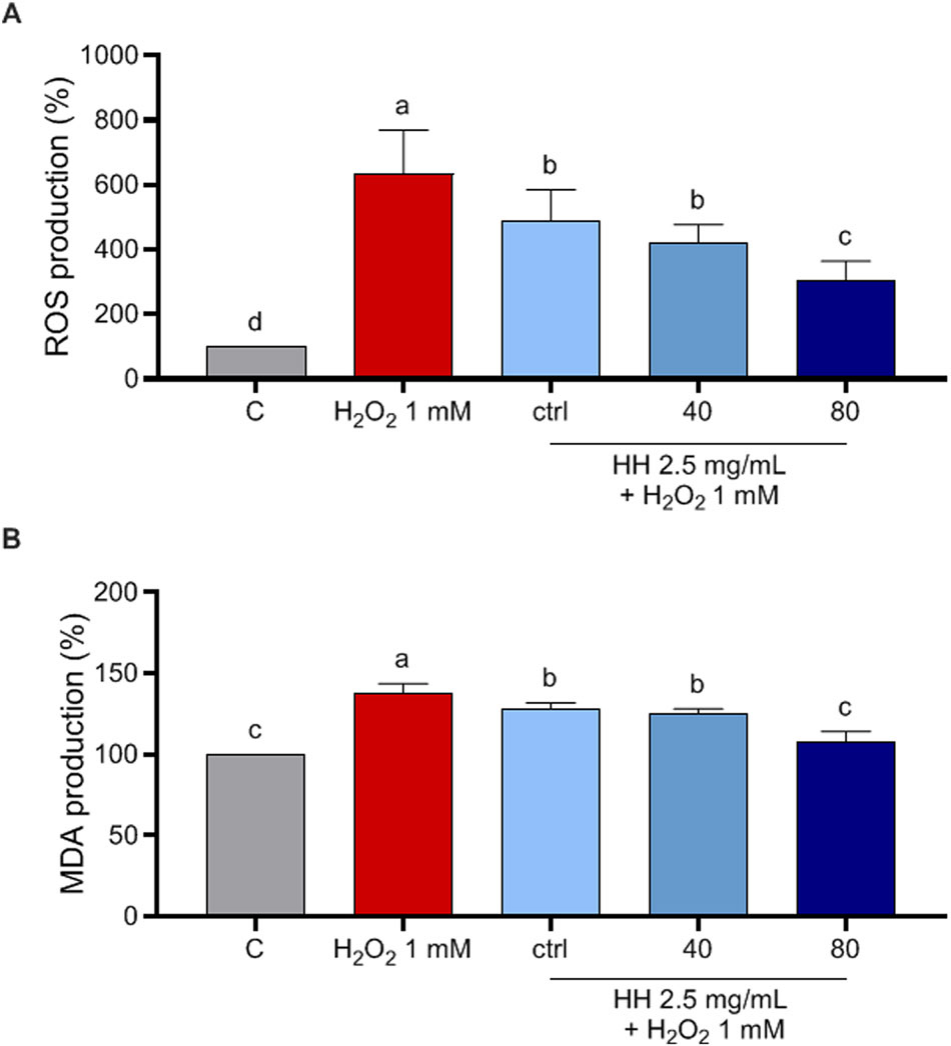
Antioxidant activity of herring hydrolysates in Caco-2 cells. Modulation of intracellular H_2_O_2_-induced ROS levels after the pretreatment with 2.5 mg/ml HH ctrl, HH40, and HH80 (**A**) and modulation of H_2_O_2_-induced MDA production after the pretreatment with 2.5 mg/ml of HH ctrl, HH40 and HH80 (**B**). The data are represented as the means ± SD of six independent experiments, performed in triplicate. Statistical analysis was performed by one-way ANOVA, followed by Tukey’s post-hoc test. Different lowercase letters indicate a significant difference (*p* < 0.05) between different concentrations.

Oxidative stress occurs when the production of oxidizing agents, free radicals, and reactive oxygen species (ROS) exceeds the antioxidant capacity of cellular antioxidants within a biological system. Cellular and organelle membranes, composed of various lipid classes, are especially prone to damage from ROS. This leads to a cascade wherein free radical species generate reactive intermediates, facilitating further reactions such as the formation of malondialdehyde (MDA) and related compounds, collectively referred to as thiobarbituric acid reactive substances (TBARS). Consequently, MDA serves as a well-established biomarker in biological and medical sciences for assessing oxidative stress associated with various health conditions. To deepen the antioxidant activities of HH peptides, their antioxidant effects at cellular level were investigated, using human intestinal Caco-2 cells as cellular system of interest, being intestinal cells the first physiological barrier that these peptides potentially may encounter upon consumption. Notably, for excluding potential cytotoxic effects, the cellular viability of Caco-2 cells treated with all HH (0.1–10 mg/ml) was assessed by MTT assays and Fig. S1A shows that after a 48-h treatment, no cytotoxic effect was observed up to 10 mg/mL versus control cells (C), indicating that all HH did not cause a cytotoxic effect in this dose range on Caco-2 cells. Then, to evaluate the protective role of HHs at human intestinal level by modulating ROS and MDA production induced by H_2_O_2_, experiments at cellular level were conducted. Our results of fluorometric Intracellular ROS assay clearly demonstrate that Caco-2 cells treated only with H_2_O_2_ showed an increase ROS levels up to 634.7 ± 134.1% *versus* the control cells, which was attenuated by the pre-treatment with HH ctrl, HH40 and HH80 2.5 mg/ml up to 488.9 ± 95.44%, 420.3 ± 57.02% and 304.6 ± 59.85%, respectively (Fig. 3A). These findings show that the pre-treatments with all the HHs preserve the Caco-2 cells against the increase of intracellular ROS induced by the H_2_O_2_ addition, thus restoring the ROS levels. These results are in agreement with our previous study^16^ where Rainbow trout-derived (*Oncorhynchus mykiss*) peptides show, among multifunctional properties, antioxidant activity on Caco-2 cells and with other investigations in which fish and fish byproduct hydrolysates show antioxidant properties^17,18^. It is worth mentioning that HH pre-treated with higher ultrasound power (HH40 and HH80) resulted to be significantly more effective in decreasing the H_2_O_2_-induced ROS production in Caco-2 cells compared with HH ctrl, when tested at the same concentration, thus suggesting that pretreatment of the fish rest raw material with this green and sustainable technique could be useful to improve the in vitro activity of HH, probably due to the different relative abundance of bioactive peptides generated in the final product, as highlighted by peptidomics analysis. These results are in line with the study conducted by Sae-leaw et.al, ^19^ who demonstrated that ultrasound pretreatment of salmon scale ossein increased the antioxidant ability of hydrolyzed fish collagen, in particular highlighting that ultrasound pretreatment conducted with intensity power of 750 W followed by heating treatment was a successful strategy to increase the release of collagenous material from ossein, which was subsequently hydrolyzed with alcalase, finally obtaining a mixture of peptides presenting a higher degree of hydrolysis than control sample and exhibiting the highest ABTS and FRAP bioactivity; moreover, Kangsanant *et. al*, demonstrated that the application of ultrasound pretreatment of Nile tilapia fish (*Oreochromis niloticus*) proteins combined with conventional hydrolysis could be applied as an innovative method for the production of fish-derived hydrolysates for the improvement of their bioactivity such as their antioxidant ability, reporting an augmented ability of the pretreated tilapia hydrolysate of inhibiting nitric oxide production and showing improved antioxidant activity at the cellular level, exploiting RAW264.7 cell line^20^. Considering these results, the capacity of HH to modulate the H_2_O_2_-induced lipid peroxidation in human intestinal Caco-2 cells was assessed by the MDA evaluation. According to the observed increase of ROS after the H_2_O_2_ treatment, a clear increase of lipid peroxidation was observed up to 138 ± 5.74% versus the control cells. More precisely, our results showed that the pretreatment with HH ctrl, HH40, and HH80 (2,5 mg/mL) resulted in a decrease in MDA levels by 127.9 ± 4%, 125 ± 3.14%, and 107.2 ± 6.9%, respectively, thus restoring the lipid peroxidation baseline levels (Fig. 3B).

### Effects of herring peptides on the in vitro ACE and DPP-IV inhibitory activity

There are numerous studies in the literature regarding the multifunctional activity of bioactive peptides, conferred by their chemical-physical properties and by the heterogeneous peptide composition of the mixture of food-derived hydrolysates^12^. In detail, the antioxidant, DPP-IV inhibitory, and ACE inhibitory activity are often correlated, thanks to the common characteristics necessary to exert these beneficial activities^15,21^, as the low molecular weight, the presence of specific amino acids such as the hydrophobic, aromatic, and acidic ones^22–24^.

The multifunctionality of peptide mixtures fits well in the context of metabolic syndrome, where oxidative stress is a key component along with inflammation, which subsequently involves hyperglycemia and hypertension^25^. Moreover, many studies have focused on the anti-diabetic and hypotensive potential of fish peptides showing DPP-IV and ACE inhibitory activity. Hong and colleagues identified a bioavailable anti-diabetic peptide derived from silver carp swim bladder hydrolysates (WGDEHIPGSPYH), in particular identified in the protein hydrolysate obtained with Neutrase enzyme, showing the highest DPP-IV inhibitory activity; this identified peptide was able to cross intact the Caco-2 cell monolayer, besides showing good inhibition for soluble DPP-IV and promoted insulin secretion at the cellular level, while in silico studies^26^. Moreover, Dong et al. carried out an interesting study, obtaining a novel ACE inhibitory peptide (VGLFPSRSF) from steam-exploded fish skin hydrolysates, showing gastrointestinal enzyme hydrolysis resistance as well^27^.

**Fig. 4:**
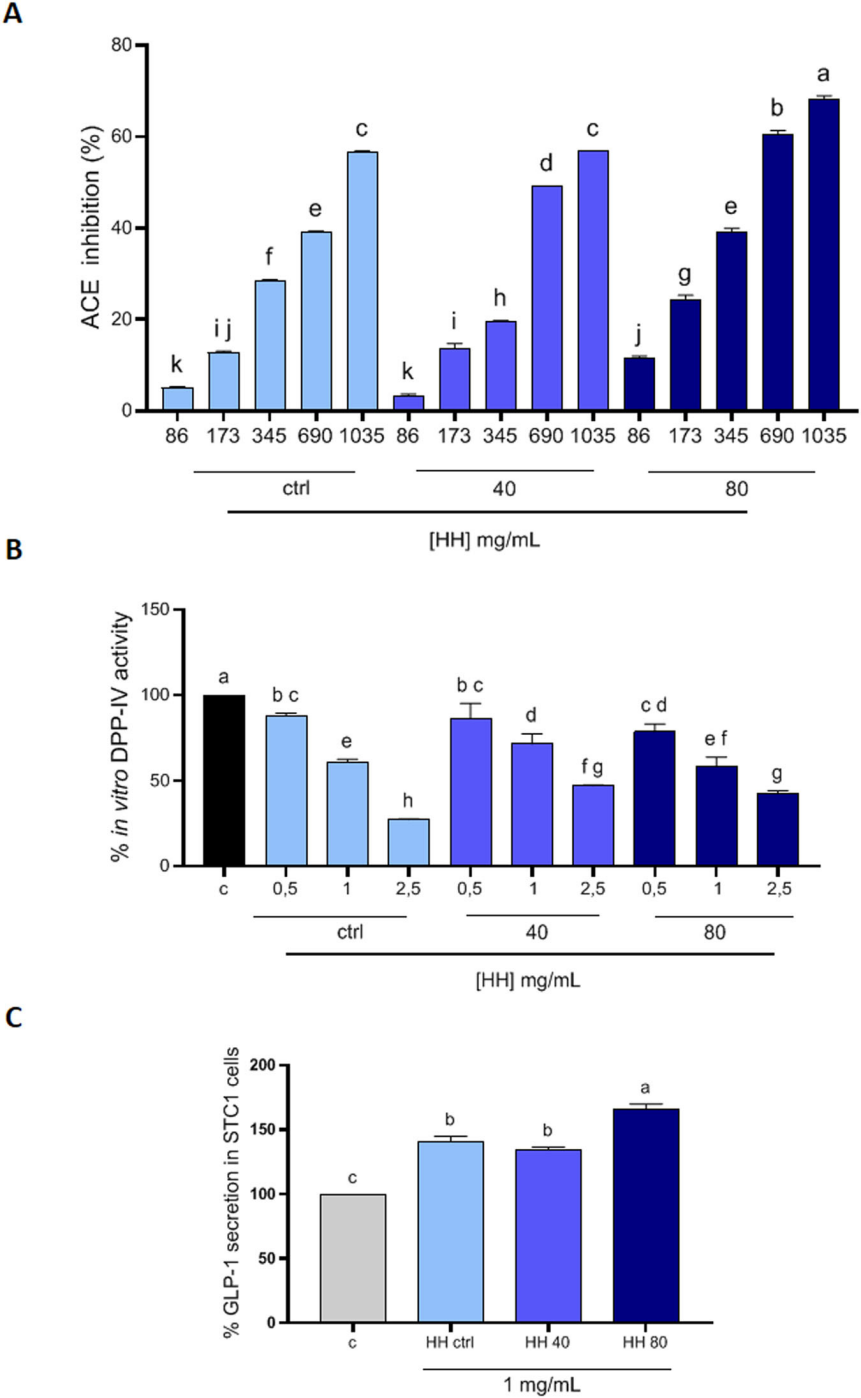
Evaluation of in vitro ACE and DPP-IV inhibitory activity and GLP1 secretion in STC1 cells of the HHs. Results of % ACE inhibition of HH ctrl, HH40 and HH80 (**A**) concentrations (µg/ml) vs control; inhibition of the activity of human recombinant DPP-IV (**B**); Effect of HHs on the GLP-1 concentration levels in both STC1 cells (**C**). Data are represented as the means ± SD of six independent experiments, performed in triplicate. Statistical analysis was performed by one-way ANOVA, followed by Tukey’s post-hoc test. Different lowercase letters indicate a significant difference (*p* < 0.05) between different concentrations.

In light of these considerations, in our study, we evaluated both the in vitro DPP-IV and ACE inhibitory activity of HH peptides, intending to evaluate, furthermore, whether the use of the increasing power of ultrasound pretreatment could exert a positive effect on these biological activities, as found for the antioxidant activity. The results showed a dose-dependent activity, in particular suggesting that HH ctrl exhibited ACE inhibitory activity of up to 56.81% ± 0.10 at a concentration of 1035 µg/mL (Fig. 4A). HH40 reduced ACE activity by up to 56.88% ± 0.002 at the same concentration (Fig. 4A), while HH80 demonstrated the highest inhibition of up to 68.36% ± 0.57 at 1035 µg/mL (Fig. 4A). Detailed results are reported in Supplementary materials (Table S3).

Interestingly, in herring hydrolysates treated with the highest ultrasound powers, there is a greater ACE inhibitory capacity, especially at the highest concentrations, confirming the bioactivity trend assessed for the antioxidant activity. Among the peptides identified in HH40 and HH80, some of them, have a structural motif that makes them good candidates as ACE inhibitors i.e., KLQPSII, TEAPLNPK, VDKGVVPL, DAPRAVFPS, AGFAGDDAPR, which contain hydrophobic amino acids (e.g., proline, leucine, or isoleucine) at the C-terminal or penultimate position of peptide sequence. Additionally, these peptides exhibit greater intensity in HH40 and HH80 compared to the HH ctrl. However, further bioassays or molecular docking studies would be needed to verify their actual inhibitory properties.

To study the in vitro DPP-IV inhibitory activity of HHs, experiments were performed using the purified human recombinant DPP-IV enzyme and H-Gly-Pro-AMC substrate, whose fluorescence signal was monitored at 465 nm (excitation 350 nm). The results indicated that HH ctrl inhibited the DPP-IV activity by 72.72 ± 0.53% at a concentration of 2.5 mg/mL (Fig. 4B). HH40 inhibited the enzyme activity by 52.92 ± 0.49% at the same concentration (Fig. 4B). HH80 demonstrated the highest inhibition of the DPP-IV activity by 57.68 ± 1.84% at 2.5 mg/mL (Fig. 4B). Detailed results are reported in Supplementary materials (Table S4). Interesting to observe that there was no improvement in the DPP-IV inhibitory activity for the HHs at higher ultrasound power. Indeed, the samples obtained using ultrasounds were demonstrated to be less active as DPP-IV inhbitors, clearly suggesting that the use of ultrasound pre-treatment coupled with enzymatic hydrolysis led to the enrichement of those peptides which are much more responsable of certain biological activity (i.e., antioxidant, ACE inhibitory activity) compared to other ones.

### Effects of herring peptides on GLP1 secretion in STC1 cells

The release of satiety hormones by enteroendocrine cells in reaction to the nutrient’s presence in the gastrointestinal tract is crucial for maintaining energy balance. Glucagon-like peptide-1 (GLP-1) is a hormone produced by intestinal L cells with insulin-releasing and antidiabetic action, rapidly degraded by DPP-IV^28^. It is well known that GLP-1, along with GIP (glucose-dependent insulinotropic peptide), triggers the incretin effect by binding to the GLP-1 receptor on pancreatic β-cells, so inducing an increase of the intracellular calcium levels that promote the insulin release in response to glucose. Moreover, GLP-1 promotes glycemic control by limiting glucagon secretion and enhancing glucose utilization in peripheral tissues. Since intestinal secretin tumor cells (STC-1) share numerous characteristics with native intestinal enteroendocrine cells, they are frequently employed in screening compounds that influence the secretion of gastrointestinal hormones in vitro. As the inhibition of DPP-IV and prolongation of GLP-1 half-life is of greater interest in the development of functional foods, the encouraging results of the HHs on in vitro DPP-IV inhibitory activity brought us to investigate their ability to modulate GLP-1 production in STC-1. MTT experiments (Supplementary Fig. S1B) reveal the absence of cytotoxic effects on STC-1 cells treated with up to 5 mg/ml of HH ctrl, HH40, and HH80, respectively. The concentration of 1 mg/mL was chosen to evaluate the impact of the hydrolysates on GLP-1 secretion. As shown in Fig. 4C, HH ctrl, HH40, and HH80 significantly increase the GLP-1 production up to 140.8 ± 4.09%, 134.1 ± 2.35%, 165.6 ± 4.32%, respectively, versus control. The GLP-1 secretion seems to be affected by all the herring hydrolysates and these results are in line with recent investigations where other fish hydrolysates, specifically salmon (*Salmo salar*) and blue whiting (*Micromesistius poutassou*) hydrolysates, increase GLP-1 secretory potential in GLUTag and STC-1 cells, respectively, in particular by activating the signaling pathway for GLP-1 through the stimulation of the increase in intracellular calcium levels, even though it was demonstrated that these fish hydrolysates diminished their secretagogue effect after simulated gastrointestinal digestion, remaining safe and not affecting the barrier integrity of differentiated Caco-2/HT-29MTX co-cultured cells^29^. Nevertheless, our results indicate that the highest ultrasounds pre-treatment (HH80) significantly boosted the ability to induce GLP-1 secretion compared to control conditions and HH ctrl, suggesting the direct implication of the ultrasound treatment on HHs in modulating the biological effect.

### Effect of the simulated gastrointestinal digestion (SGID) on ultrasonicated herring hydrolysates

**Fig. 5:**
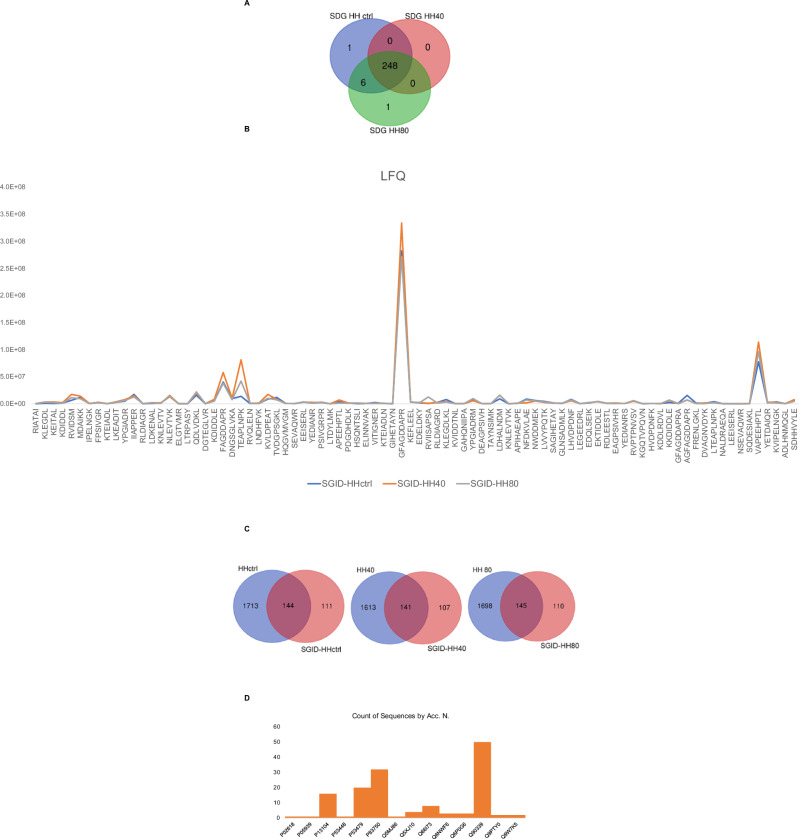
Peptidomic analysis of digested herring hydrolysates. The number of total peptides identified in digested herring protein hydrolysate after different ultrasonic power treatments (**A**). The trend of LFQ intensity of digested ultrasonicated and not herring peptide within the molecular weight range of 600 < MW < 1000 Da (**B**). The number of peptides resistant to gastrointestinal digestion (**C**). Peptide distribution to parent herring proteins (**D**).

Food peptide bioaccessibility evaluation is essential for understanding the health benefits associated with consuming bioactive peptides in foods or supplements. In this study, the simulation of gastrointestinal digestion by using pepsin followed by trypsin, was employed to investigate the fate of ultrasonicated herring protein hydrolysates. The peptide stability during digestion is crucial for determining bioaccessibility. The stability study was conducted using LC-MS/MS to evaluate the susceptibility of peptides to degradation by enzymes or pH changes during digestion. The three different herring protein hydrolysates HH ctrl, HH40, and HH80 were digested producing SGID-HH ctrl, SGID-HH40, and SGID-HH80, respectively. The digested samples were then analyzed using HPLC-MS/MS. Overall, 253 peptides with molecular weights below 3 kDa were identified after simulated gastrointestinal digestion, with 97% of these peptides being common across all conditions (Fig. 5A, Supplementary Table S5). Most of the identified peptides even after the SGID, belong to the myosin heavy chain, fast skeletal muscle, actin, tropomyosin alpha-1 chain, and myosin regulatory light chain 2 that were identified by 80, 30, 18, and 14 unique peptides, respectively. Although no change in the composition of the peptide mixtures is observed after ultrasonication as seen in the initial herring protein hydrolysates, even after gastrointestinal digestion, the peptides in the ultrasonicated samples are more abundant compared to those in the non-ultrasonicated sample (Fig. 5B). This finding is consistent with observations from the initial herring protein hydrolysates.

As shown in Fig. 5C, a relevant number of peptides, specifically 144 were stable to conditions of gastric hydrolysis by comparing HH ctrl *versus* SGID-HH ctrl. Similarly, 141 and 145 are stable peptides after ultrasonication and SGID versus HH40 and HH80. Among the bioaccessible peptides in SGID-HH ctrl, SGID-HH40, and SGID-HH80, most of them derived from Q90339 (myosin in heavy chain, fast skeletal muscle, with 50 peptides), followed by P83750 (actin, cytoplasmic 1, with 32 peptides), and P53479 (actin, alpha skeletal muscle, with 20 peptides). Among the 111, 107, and 110 peptides detected in SGID-HH ctrl, SGID -HH40, and SGID-HH80, respectively, 75 are breakdown peptides derived from enzymatic cleavage of longer parent peptides detected in the HH ctrl, HH40, and HH80 hydrolysate.

### Intestinal trans-epithelial transport of simulated digested herring peptides and analysis of herring peptides trans-epithelial transported by differentiated Caco-2 cells

The oral bioavailability of fish peptides has received limited attention in existing literature. It is well known that the intestinal epithelium acts as the primary barrier to absorption for all food components. Understanding the bioavailability of fish peptides has significant implications for human health and nutrition. These peptides exhibit various bioactive properties, including antioxidant, antimicrobial, and antihypertensive activities^30^, however, their efficacy largely depends on their ability to be absorbed through the intestinal barrier. For this reason, investigating their resistance to gastrointestinal enzymes and their intestinal bioavailability is crucial. In this scenario, simulated gastrointestinal digestion conducted on fish peptides with proper enzymes and further absorption studies carried out with the Caco-2 cell model, serve as valuable tools for offering insights into this biological aspect, contributing to updating the traditional bottom-up approach for the food hydrolysate production.

Shimuzo et al. studied the intestinal permeability of fish collagen peptides employing Caco-2 cells, highlighting that the amount of permeated fish peptides was inversely proportional to their molecular size, demonstrating that they are probably transported via the paracellular pathway across the intestinal epithelium^31^. Moreover, Taroncher *et al*., evaluated the antioxidant and cytoprotective effect of the pure and the bioavailable fraction of salmon (*Salmo salar*), mackerel (S*comber scombrus*), and herring (*Clupea harengus*) protein hydrolysates, revealing that for both types of samples, there was the same bioactivity trend since there were no significant differences in terms of toxicity and antioxidant ability; Notably, in this study, it was demonstrated that herring protein hydrolysate was able to significantly suppress H_2_O_2_ induced ROS and lipid peroxidation production, besides resulting cytoprotective against the T-2 mycotoxin in experiments conducted on Caco-2/TC7 cells^18^. Furthermore, Tian et al. generated an alcalase fish hydrolysate enriched in antiplatelet peptides, ten of which were identified in the absorbate of the basolateral side of Caco-2 cells treated with the peptides following the simulated gastrointestinal digestion, suggesting that these bioactive fish peptides were bioavailable as well^32^.

**Fig. 6:**
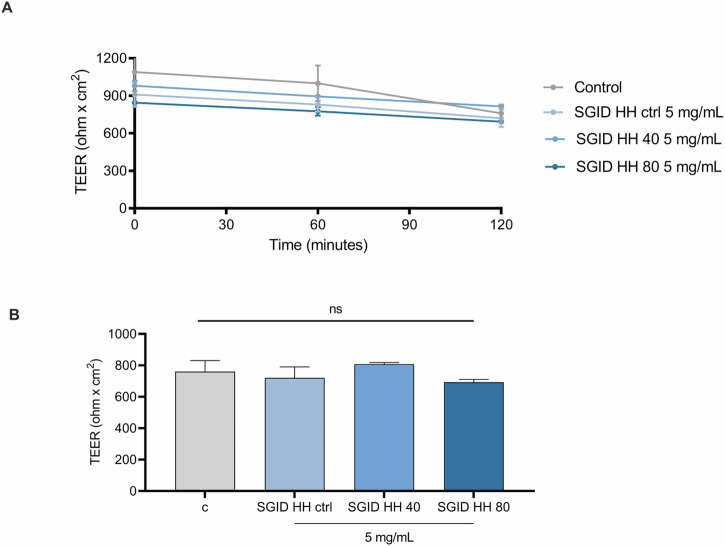
TEER measurements in Caco-2 monolayer. **A** Time course of TEER changes recorded in 2 h in untreated (control), SGID-HH ctrl, SGID-HH40, and SGID-HH80-treated Caco-2 cells. **B** TEER values after 120 min. Data are the mean ± s.d. of three experiments performed in duplicate. ns: not significant.

Differentiated Caco-2 cells, which mimic the morphology and function of mature enterocytes and express brush border peptidases and transporters, provide a valuable in vitro model for studying this barrier. When grown on filters, these cells create a two-compartment system where the apical (AP) side mimics the intestinal lumen, while the basolateral (BL) side represents the intestinal vascular and lymphatic circulation in vivo^12^. Hence, this model assesses the ability of peptides to cross the intestinal epithelium and enter systemic circulation. The transport of peptides across the cell monolayer was evaluated, and the number of peptides that cross the barrier represents their bioaccessibility. However, peptides may be cleavage by brush-border peptidases that are responsible for breaking down larger peptides into smaller components that can be absorbed by the intestinal cells. Peptides that are resistant to degradation by these brush border enzymes have a higher chance of being absorbed intact into the bloodstream, where they can exert their physiological effects. For trans-epithelial transport experiments, SGID-HH ctrl, SGID-HH40, and SGID-HH80 (5 mg/ml) were incubated in the AP compartment. To evaluate the quality and integrity of the Caco-2 cells polarized epithelial cell monolayer during the trans-epithelial transport experiments, the Transepithelial electrical resistance (TEER) was monitored over the experimental time. The results shown in Fig. 6A, B, whose values (ohm × cm^2^) are reported in Table 1, indicate that all the herring hydrolysates did not alter the intestinal monolayer permeability.

**Table 1:** Trans-epithelial electric resistance (TEER) values (ohm × cm^2^) measured after 0, 60, 120 min

Time (min)	*C*	SGID HH Ctrl 5 mg/ml	SGID HH 40 5 mg/ml	SGID HH 80 5 mg/ml
	mean (ohm × cm^2^) ± SD	mean (ohm × cm^2^) ± SD	mean (ohm × cm^2^) ± SD	mean (ohm × cm^2^) ± SD
0	1090 ± 183.8	910 ± 99.0	980 ± 42.4	845 ± 7.1
60	1000 ± 141.4	830 ± 56.6	895 ± 7.1	775 ± 35.4
120	760 ± 70.7	720 ± 70.7	815 ± 0.0	692.5 ± 17.7

Data are the mean ± s.d. of three experiments performed in duplicate.

*C* Control, *SGID* simulated gastrointestinal digestion.

Peptides collected from the basolateral (BL) side of Caco-2 cell monolayers were identified using an Orbitrap Fusion™ Tribrid™ mass spectrometer, with the apical (AP) side serving as the control.

**Fig. 7:**
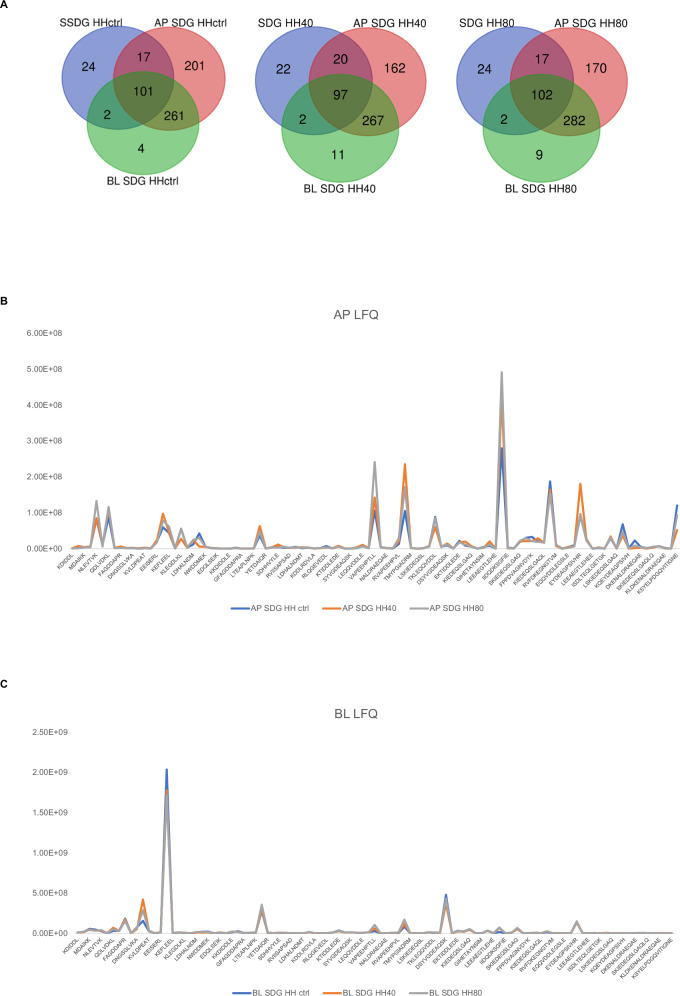
Peptidomic analysis of AP and BL solutions. Peptides identified in AP and BL side of HH ctrl, HH40, and HH80 (**A**). The trend of LFQ intensity of AP (Apical side of Caco-2 cells, **B**) and BL peptides (Basolateral side of Caco-2 cells, **C**).

The analysis revealed 101, 97, and 102 peptide sequences, as listed in Supplementary Table S6, on both the AP and BL sides for each condition (Fig. 7A). This finding indicates that these peptides can resist gastrointestinal digestion, enduring enzymatic cleavage by brush border membrane peptidases where they are in contact with the AP compartment, and being transported intact across Caco-2 cell monolayers. The molecular weight of the stable peptides to gastrointestinal conditions, to brush border peptidases and absorbed by BL compartment hovers around 900–1200 Da. Although the effect of ultrasonication is not impactful in terms of peptide composition, ultrasonication induces a greater release of peptides in terms of their abundance, which is reflected both in the initial composition of the herring protein hydrolysate and in AP and BL peptide mixture. Figure 7 shows the LFQ trend of peptides in AP and BL sides (Fig. 7B, C) highlighting higher LFQ values in HH40 and HH80 compared to HH ctrl.

Detailed analysis revealed that the Caco-2 cells absorbed 70% of the peptides originating from Q90339 (myosin heavy chain, fast skeletal muscle) that were present in the SGID-HH ctrl. This absorption was followed by 80% of the peptides (comprising 25 peptides) from P83750 (actin, cytoplasmic 1), and 85% of the peptides (comprising 17 peptides) from P53479 (actin, alpha skeletal muscle). Moreover, some peptides that have crossed the intestinal lumen are found to be enriched in the BL compartment from a comparison between the LFQs demonstrating the role the role of Caco-2 cells not only as a model for intestinal absorption but also in assessing the sieving and enriching properties of peptides during their transit from initial hydrolysates to the BL compartment. Peptides like IIAPPER, FAGDDAPR, and TEAPLNPK derived from actin, cytoplasmic 1 (P83750) were found strongly enriched in the BL compartment. Among the peptides absorbed, several are reported to exhibit various bioactivities according to the BIOPEP database search. Both actin, alpha skeletal muscle (P53479), and actin, cytoplasmic (P83750) are sources of antioxidant peptides. The peptides AGPSIVH (actin, alpha skeletal muscle, [213–219]), AGDDAPR (actin, cytoplasmic, [398–404]), IIAPPER (actin, cytoplasmic, [388-394]), VAPEEHPV (actin, cytoplasmic, [527–534]), and WDDMEK (actin, cytoplasmic, [449–454]) have been identified as antioxidants. These findings are corroborated by experimental results observed in initial herring hydrolysate and are supported by database comparisons. Furthermore, peptides such as MYPGIA ([539-544]), EKSYELP ([665-671]), and IVGRPR ([495-500]) from actin, cytoplasmic 1 have shown ACE-inhibitor activity according to the BIOPEP database. Notably, EKSYELP, derived from enzymatic hydrolysis of smooth-hound viscera using Esperase®, has demonstrated blood pressure-lowering effects in hypertensive rats^33^. Additionally, IVGRPR, isolated for the first time from thermolysin-digested dried bonito, significantly reduced the systolic blood pressure of SHRs by 25 mmHg 6 h after administration at a dose of 10 mg/kg body weight^34^. Our dataset also includes DPP-IV inhibitory peptides such as VAPEEHPT (actin, alpha skeletal muscle, [178-185]). The intestinal absorption and DPP-IV inhibitor activity of this peptide were assessed by Ravellec et al. ^35^. Overall, LC-MS analysis highlighted that Caco-2 cells showing selective permeability effectively can be used to evaluate and enrich peptides with potential biological activities, making them valuable in the screening process for developing functional foods or nutraceuticals. These findings underscore the role of Caco-2 cells not only as a model for intestinal absorption but also in assessing the sieving and enriching properties of peptides during their transit from initial hydrolysates to the basolateral compartment, demonstrating selective absorption and potential enhancement of specific bioactive properties.

Our study provides clear evidence that ultrasonication, a promising pre-treatment technology applied prior to enzymatic hydrolysis to produce fish protein hydrolysate from herring side streams represents a valuable and sustainable strategy for obtaining peptide mixtures endowed by multifunctional in vitro bioactivity. It is generally accepted that ultrasonication significantly improves the solubility and the yield of food component extraction, such as proteins. In this study, focusing the attention on the medium- and small-sized enriched fractions of herring hydrolysates, it was clearly demonstrated that ultrasonication coupled to enzymatic hydrolysis did not modify the qualitative composition of peptide mixtures but instead, it was effective in changing the relative abundance of peculiar peptides. These important results show a significant impact on the bioactivity of the samples obtained by ultrasound pre-treatment before enzymatic hydrolysis (HH40 and HH80) compared to the control hydrolysate sample obtained by only enzymatic hydrolysis (HH-ctrl). Indeed, the findings suggest that through enzymatic hydrolysis, multifunctional peptide mixtures are obtained from herring side streams endowed with in vitro antioxidant, hypotensive, and hypoglycemic activity. In this context, the ultrasound pre-treatment significantly modulates the biological activity. In fact, the herring hydrolysates obtained by ultrasound pre-treatment of fish side streams prior to enzymatic hydrolysis led to samples selectively exhibiting much effective antioxidant, ACE inhibitory, and GLP-1 level enhancer activity, respectively, on the contrary, it reduced the DPP-IV inhibitory peptides. Importantly, we demonstrated by a comprehensive and multidisciplinary in vitro and in situ approach, that peptides obtained from this process not only exhibit bioactivity but are also bioaccessible, bioavailable, and metabolically stable, in Caco-2 cell models. Indeed, our findings underscore the role of Caco-2 cells not only as a model for intestinal absorption but also in assessing the sieving and enriching properties of peptides during their transit from initial hydrolysates to the basolateral compartment, demonstrating selective absorption and potential enhancement of specific bioactive properties. These findings validate the use of ultrasound technology coupled to enzymatic hydrolysis as an effective approach for obtaining multifunctional peptide mixtures. This advancement contributes to the food science and technology bringing us closer to conducting in vivo studies using animal models more responsibly and ethically. Despite challenges, ongoing research continues to expand our understanding of herring peptides and ultrasonication, paving the way for innovative and sustainable solutions in food science and technology.

## Material and methods

### Chemicals

All chemicals and reagents were of analytical grade and from commercial sources. Dulbecco’s modified Eagle’s medium (DMEM), L-glutamine, fetal bovine serum (FBS), phosphate-buffered saline, penicillin/streptomycin, 24 or 96-well plates were purchased from Euroclone (Milan, Italy). Morpholinoethane sulfonic acid, (4-(2-hydroxyethyl)-1-piperazineethanesulfonic acid, hydrochloric acid (HCl), hippuric acid, hippuryl-histidyl-leucine, ACE from porcine kidney, 3-(4,5-dimethylthiazol-2-yl)-2,5- diphenyltetrazolium bromide (MTT), ROS and lipid peroxidantion (MDA) assay kits were from Sigma- Aldrich (St. Louis, MO, USA). The DPP-IV enzyme and the substrate solution [5 mM H-Gly-Pro conjugated to aminomethylcoumarin (H-Gly-Pro-AMC)] were provided by Cayman Chemicals (Michigan, USA). GLP-1 ELISA kit catalog no. EGLP-35K was from Millipore (Watford, UK).

### Preparation of fish raw material

The residual raw material of Atlantic herring (*Clupea harengus L*) used in this study was delivered directly from the fish filleting facility of Fosnavåg Pelagic (Fosnavåg, Norway) in February 2023. The rest raw material mainly consists of bones, fillet cuts, heads and backs. The fish side streams were minced using a mincer with 4.5 mm hole size (Hobart A 200N), divided into batches of 1 kg, and immediately frozen and stored at −80 °C until enzymatic hydrolysis. The day before the experiment, the frozen herring mince was placed in a cold room at a temperature of 4 ± 1 °C for 24 h for thawing.

### Ultrasonication and enzymatic hydrolysis of herring side streams

The ultrasound treatment of herring side streams was performed before enzymatic hydrolysis at 600 W (40% max ultrasonic power) and 1200 W (80% max ultrasonic power) with a 20 kHz probe (Sonics & Materials Inc., Danbury, CT., USA, model: VCX 1500). The probe has a vibrating titanium tip of 1.2 cm which was immersed in the herring raw material mixed with water in a ratio 1:1 followed by its irradiation with an ultrasonic wave directly from the horn tip. After the ultrasonication of herring mince, the enzymatic hydrolysis was performed in 4 L closed glass vessels placed in a water bath at 52 °C. The mixture was stirred at 150 rpm with an overhead stirrer. When the temperature of the mixture was 50 °C, Alcalase was added at levels of 0.1%. The pH value of the herring mixture at the end of enzymatic hydrolysis was 6.7. After 60 min of hydrolysis, bones were removed by filtering the hydrolysate through a sieve before the enzymes were inactivated by heating up to 90 °C for 10 min in a microwave oven. The mixture was cooled down and transferred to 1 L centrifugation bottles and then centrifuged at 4100 *g* at 4 °C for 30 min. The liquid fraction (lipids and water-soluble proteins) was separated from the insoluble fraction. The liquid fraction was placed in a separatory funnel and allowed to settle, and then separated into oil and water-soluble proteins. The water-soluble protein phase representing fish protein hydrolysate (FPH) was collected and dried in the laboratory vacuum freeze-dryer (Labconco Freezone Console 12 L Freeze Dry System). The Atlantic herring hydrolysates (HH) investigated in the study were: control sample of hydrolysate (HH ctrl) extracted without ultrasound pre-treatment and two other hydrolysates recovered with the use of ultrasound pre-treatment prior to enzymatic hydrolysis at 600 W (HH40) and 1200 W (HH80) corresponding to 40% and 80% of the maximum ultrasound power.

### Simulate the gastrointestinal digestion (SGID) of herring protein hydrolysates

Trypsin and pepsin enzymes were sequentially used to simulate the gastrointestinal digestion of Atlantic herring hydrolysates. In detail, the pH of the solution was raised to 2 or 8, respectively, for pepsin and trypsin digestion [1:100 (w/w) E/S ratio] by adding 1 M HCl or 1 M NaOH. Pepsin was rendered inactive by raising the pH to 8 following 2 h of peptic digestion at 37 °C. In contrast, tryptic digestion (2 h) was halted by heating the mixture to 95 °C for 5 min. Using a Millipore UF system, herring hydrolysate was run through ultrafiltration membranes with a 3 kDa cut-off (Millipore, Bedford, MA, USA). After being lyophilized, the recovered peptides (<3 kDa) were kept at −80 °C until needed.

### Cell culture

Caco-2 cells, obtained from INSERM (Paris, France) and STC-1, bought from ATCC (HB- 8065, ATCC from LGC Standards, Milan, Italy) were routinely sub-cultured following a previously optimized protocol^36^ and maintained at 37 °C in a 5% CO_2_ atmosphere in DMEM containing 25 mM of glucose, 3.7 g/L of NaHCO_3_, 4 mM of stable L-glutamine, 1% non-essential amino acids, 100 U/L of penicillin and 100 μg/L of streptomycin (complete medium), supplemented with 10% heat-inactivated FBS. Regarding the co-culture, STC-1 and Caco-2 cells were cultured in a ratio of 1 to 5 for a total of 24 h before the treatment.

### 3-(4,5-Dimethylthiazol-2-yl)-2,5-diphenyltetrazolium bromide (MTT) assay

3 × 10^4^ Caco-2 cells/well were cultured in 96-well plates and treated with 0.1, 0.5, 1, 5, 10 mg/mL of hydrolysates and/or vehicle (H_2_O) in complete growth medium for 48 h at 37 °C in a 5% CO_2_ atmosphere, as previously reported^37^. A total of 6 × 10^3^ STC-1 cells/well were placed in 96-well plates and treated with 0.5, 1, 2.5, 5 mg/mL of hydrolysates and/or vehicle (H_2_O) under the same conditions as mentioned beforehand.

### Antioxidant activity of herring hydrolysate

#### Diphenyl-2-picrylhydrazyl Radical (DPPH) assay

The DPPH assay was carried out using a conventional procedure with a slight modification. In brief, 45 μL of 0.0125 mM DPPH solution (which was dissolved in methanol) was mixed with 15 μL of herring hydrolysates with final concentrations of 0.1, 0.5, 1, 5, mg/mL. The reaction for scavenging DPPH radicals took place in the dark at room temperature, then the absorbance was measured at 520 nm after 30 min incubation.

#### 2,2′-Azino-bis (3-ethylbenzothiazoline-6-sulfonic acid) diammonium salt assay

The decrease of the 2,2-azino-bis-(3-ethylbenzothiazoline-6-sulfonic) acid (ABTS) radical caused by antioxidants is the basis of the Trolox equivalent antioxidant capacity assay. A 7 mM ABTS solution (Sigma-Aldrich, Milan, Italy) was combined with 2.45 mM potassium persulfate (1:1) to create the ABTS radical cation (ABTS+•), which was then kept for 16 h at room temperature and in the dark. The ABTS+• was diluted in 5 mM phosphate buffer (pH 7.4) to obtain a stable absorbance of 0.700 (±0.02) at 730 nm, which was employed to prepare the ABTS reagent^38^. For the experiment, 70 μL of diluted ABTS+• was mixed with 5 μL of herring hydrolysates at final concentrations of 0.05, 0.1, 0.5, 1, and 2.5 mg/mL. After 30 min of 30 °C incubation, the absorbance at 730 nm was measured using a microplate reader Synergy H1 (Biotek).

#### FRAP assay

The FRAP assay measures a sample’s capacity to convert ferrous ions (Fe^2+^) from ferric ions (Fe^3+^)^39^. Thus, 70 μL of FRAP reagent was combined with 5 μL of herring hydrolysates at the final concentrations of 0.05, 0.1, 0.5, 1, and 2.5 mg/mL. The FRAP reagent was generated by combining 1.3 mL of 0.3 M acetate buffer (pH 3.6), 1.3 mL of 20 mM FeCl_3_ × 6 H_2_O, and 1.3 mL of a 10 mM TPTZ (Sigma-Aldrich, Milan, Italy) solution in 40 mM HCl. The absorbance at 595 nm was measured after the microplate was incubated for 30 min at 37 °C. Microplate reader Synergy H1 (Biotek) was used to record absorbances.

#### Fluorometric intracellular ROS assay

30.000 Caco-2 cells were seeded in growth media for the whole night on a black 96-well plate. The following day, the medium was eliminated, then 50 μL of Master Reaction Mix and 50 μL of complete DMEM were added to each well, and the cells were incubated for 1 h in the dark at 37 °C and 5% CO_2_. After adding the herring hydrolysates to achieve the desired concentrations of 2.5 mg/mL, the mixture was incubated for a full day at 37 °C. To produce reactive oxygen species (ROS), cells were exposed to 1.0 mM H_2_O_2_ for 60 min at 37 °C in the dark. Fluorescence signals (ex./em. 490/525 nm) were then recorded using a Synergy H1 microplate reader (Biotek).

#### Malondialdehyde (MDA) assay

After being seeded in a 24-well plate, Caco-2 cells (2.5 × 10^5^ cells/well) were treated with the herring hydrolysates for 24 h at 37 °C in an atmosphere of 5% CO_2_. After incubating with H_2_O_2_ at a concentration of 1 mM or with vehicle (H_2_O) for 1 h, the cells were collected and homogenized in 150 μL of ice-cold MDA lysis solution that contained 3 μL of butylated hydroxytoluene. Samples were centrifuged at 13,000 *g* for 10 min, then each vial containing 100 μL of samples was filled with 300 μL of the TBA solution to generate the MDA-TBA adduct. The vials were then incubated at 95 °C for 60 min, then cooled to room temperature for 10 min in ice. Each reaction mixture was pipetted into a transparent 96-well plate containing 100 μL for measurement. The absorbance was measured at 532 nm using the Synergy H1 microplate reader (Biotek).

### Antidiabetic and antihypertensive activity of herring hydrolysates

#### In vitro measurement of the DPP-IV inhibitory activity

Using previously adjusted conditions, the experiments were run in triplicate in a half volume 96 well solid plate (white)^40^. A microcentrifuge tube was filled with 50.0 μL of each reaction, 30.0 μL of 1 × assay buffer (20 mM Tris-HCl, pH 8.0, containing 100 mM NaCl and 1 mM EDTA), 10.0 μL of each sample (at the final concentration of 0.5, 1, 2.5 mg/mL), 1.0 μM of sitagliptin (positive control), and 10.0 μL of purified human recombinant DPP-IV enzyme. Subsequently, the reagents were transferred in each well of the plate, and every reaction was initiated by adding 50.0 μL of the substrate solution (5 mM H-Gly-Pro-AMC). The reaction was then incubated for 30 min at 37 °C. The Synergy H1 fluorescent plate reader from Biotek was used to measure the fluorescence signals (excitation/emission wavelength 360/465 nm).

#### Evaluation of the GLP-1 stability and secretion at cellular level

An active GLP-1 ELISA kit (catalog no. EGLP-35K; Millipore, Watford, UK) was used to measure the GLP-1 secretion of STC-1. More precisely, the 96-well plates were seeded with 6 × 10^3^ STC-1 cells/well. After 24 h, the cells were treated for 1 h with either vehicle (C), herring hydrolysates (1 mg/mL), in growth medium. Following the treatment, the supernatant was gathered, centrifuged for 5 min at 500G, 4 °C, and then incubated for 20–24 h at 4 °C in 96-well microplates coated with a monoclonal antibody. The detection conjugate was added for 2 h after the wells had been washed. After washing the wells, the substrate solution was applied and left for 20 min. The plate was read using a Synergy H1 microplate reader (Biotek Instruments, Winooski, VT, USA) at an excitation/emission wavelength of 355 nm/460 nm after the reaction was stopped using a stop solution.

#### In vitro measurement of the ACE inhibitory activity

In vitro ACE inhibitory activity was tested by measuring with HPLC the formation of hippuric acid from hippuryl-histidyl-leucine, used as mimic substrate for ACE I. Test was performed in 100 mM Tris-HCOOH, 300 mM NaCl pH 8.3 buffer, and using ACE from the porcine kidney. HHs were tested at five different concentrations 86, 173, 345, 690, and 1035 µg/mL; IC_50_ values were calculated from the plot of percentages of ACE-inhibition vs log_10_ sample concentrations. All experimental details of sample preparation and analysis conditions have been published elsewhere^41^.

### Caco‐2 cell culture and differentiation and monolayer integrity evaluation

Caco-2 cells were grown following an earlier technique^42^. To facilitate the establishment of a confluent cell monolayer, cells were seeded on a Transwell at a density of 3.5 × 10^5^ cells/cm^2^ in a complete medium supplemented with 10% FBS in both the AP and BL compartments for 2 days. Cells were seeded, and on day three, they were moved to an FBS-free medium in the AP compartment. They were then allowed to differentiate for 18–21 days, with three weekly medium changes in between^43^. TEER of differentiated Caco-2 cells was measured at 37 °C using the Millicell voltmeter device (Millipore Co., Billerica, MA, USA) both before and after the transport experiments for monitoring the integrity of the cell monolayers.

### Trans‐epithelial transport of digested herring hydrolysates

TEER measurement was used to verify the integrity and differentiation of the cell monolayer before transport experiments. According to previously published conditions^42^, herring hydrolysates trans-epithelial transit was assessed in differentiated Caco-2 cells in transport buffer solution (137 mM NaCl, 5.36 mM KCl, 1.26 mM CaCl_2_, and 1.1 mM MgCl_2_, 5.5 mM glucose). The apical (AP) solutions were kept at pH 6.0 (buffered with 10 mM morpholinoethane sulfonic acid) and the basolateral (BL) solutions were kept at pH 7.4 (buffered with 10 mM N‑2‑hydroxyethylpiperazine‐N‑4‑butanesulfonic acid) to replicate the pH conditions found in vivo in the small intestinal mucosa. Before the transport assay, cells were equilibrated in HBSS for 15 min at 37 °C. In the AP compartment with the AP transport solution (500 μL) and the BL compartment with the BL transport solution (700 μL), digested herring hydrolysates (5 mg/mL) were added. All BL and AP solutions were collected after 2 h of absorption experiment carried out at 37 °C, and they were kept at −80 °C before analysis. Transport experiments were performed in duplicate.

### High-resolution LC-MS/MS analysis and data elaboration

Herring protein hydrolysate digested and not digested, ultrasonicated and not, were desalted using zip-tip (C18) microcolumns. All samples were dried and then dissolved with 50 μL of a solution of 99% water and 1% ACN containing 0.1% formic acid. All samples have been analyzed at UNITECH OMICs (University of Milano, Italy) using: Dionex Ultimate 3000 nano-LC system (Sunnyvale CA, USA) connected to Orbitrap Fusion™ Tribrid™ mass spectrometer (Thermo Scientific, Bremen, Germany) equipped with nanoelectrospray ion source. Peptide mixtures were pre-concentrated onto an Acclaim PepMap 100–100 µm × 2 cm C18 (Thermo Scientific) and separated on EASY-Spray column ES900, 25 cm × 75 µm ID packed with Thermo Scientific Acclaim PepMap RSLC C18, 3 µm, 100 Å using mobile phase A (0.1% formic acid in water) and mobile phase B (0.1% formic acid in acetonitrile 20/80, v/v) at a flow rate of 0.300 µL/min. The following gradient profile was used: 0.00 min, 4% B; 3.00 min, 4% B; 43.00 min, 28% B; 53.00 min, 40% B; 55.00 min, 95% B; 69.00 min, 95% B; 90.00 min, 4% B. The temperature was set to 35 °C and the samples were injected in triplicates. MS spectra were collected over an *m*/*z* range of 375–1500 Da at 120,000 resolutions (*m/z* 200), operating in the data-dependent mode, cycle time of 3 s between master scans. HCD was performed with collision energy set at 35 eV. Polarity: positive. Protein Discoverer 2.5 analyzed MS data by using Danio Renio (v2024) without any specific enzymatic cut, max missed cleavage sites: 2, min. peptide length: 4. Dynamic Modification: Oxidation/+15.995 Da (M), −1. N-Terminal Modification: Acetyl/+42.011 Da (N-Terminus) N-Terminal Modification: Met-loss+Acetyl/−89.030 Da (M). N-Terminal Modification: Met-loss/−131.040 Da (M).

### Statistical analysis

All results were expressed as the mean ± standard deviation (s.d.), where *p*-values < 0.05 were significant. Statistical analyses were performed by one-way ANOVA followed by Tukey post hoc test and two-way ANOVA followed by Šidák correction (GraphPad Prism 9, GraphPad Software, La Jolla, CA, USA).

## Supplementary information

Supplementary information

## Data Availability

Data will be available on specific requests to the authors.
