# Supplementary material for: Ultrasonicated Atlantic herring side streams as source of multifunctional bioactive and bioavailable peptides
Source: NPJ Sci Food. 2025 Feb 22;9:25. doi: 10.1038/s41538-025-00388-w (PMC11847024; doi:10.1038/s41538-025-00388-w)
Supplement: Supplementary file 1 — Supplementary information [file 41538_2025_388_MOESM1_ESM.pdf]

Supplementary materials

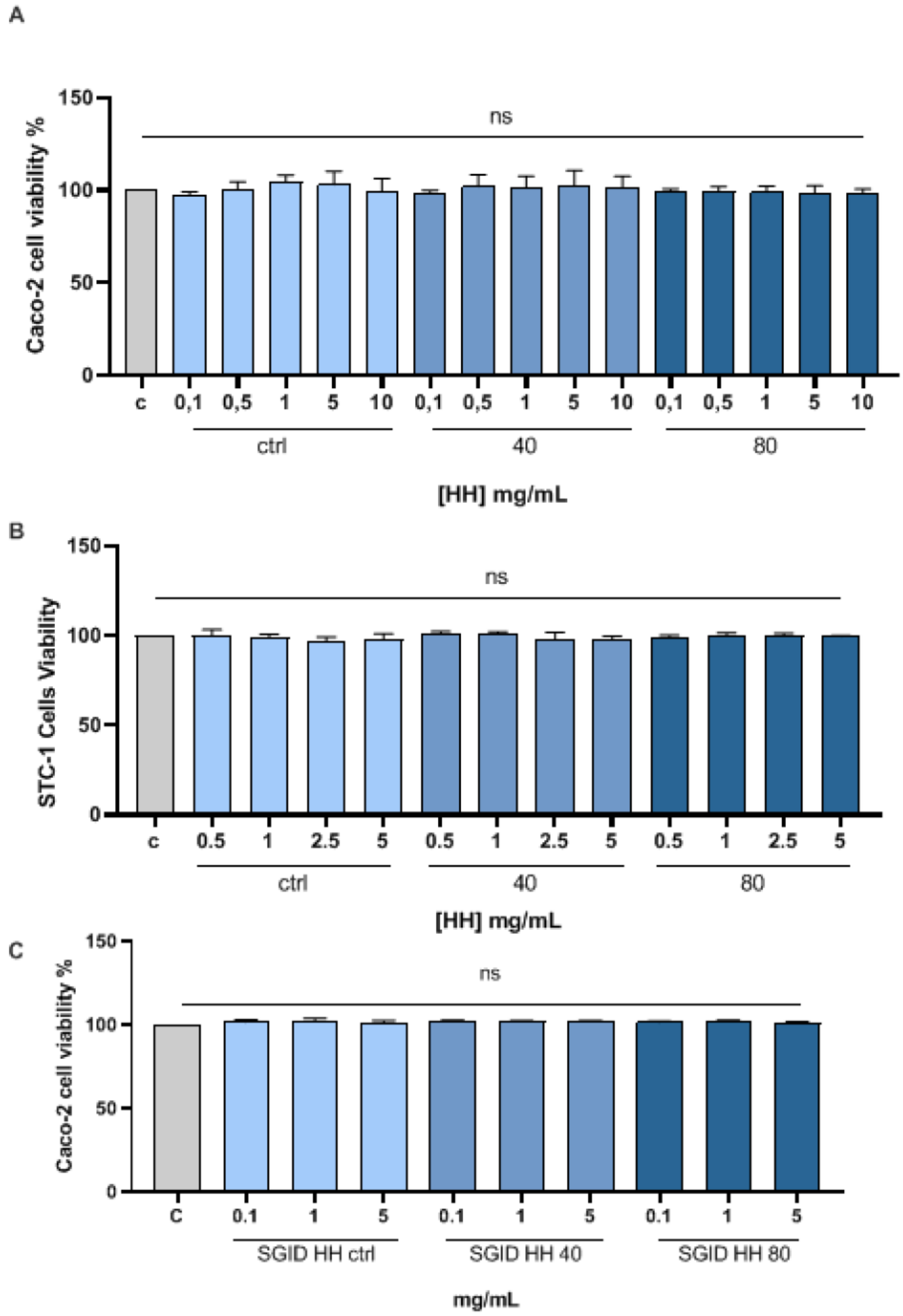

Figure S1. MTT assay. Effect of herring hydrolysates on Caco-2 and STC-1 cells viability. (A) Bar graphs indicating the results of cell viability of Caco-2 cells after HHs treatment (0.1–10 mg/mL) for 48 h, (B) STC-1 cell viability after 24 h treatment with HHs (0,5-5 mg/ml) and Caco-2 cells viability after digested HHs (0.1-5 mg/ml) treatment for 48 h and (C). The data points represent the averages ± SD of three experiments in triplicate, statistical analysis was performed by one-way ANOVA. C: control sample (H_2O), ns: not significant. (*) $p < 0.05$, (****) $p < 0.0001$.

Table S2. *In vitro* antioxidant power evaluation of the HH ctrl, HH40, HH80 by 2,2-diphenyl-1-picrylhydrazyl (DPPH), ferric reducing antioxidant power (FRAP), and 2,2-azino-bis-(3-ethylbenzothiazoline-6-sulfonic acid (ABTS) assays. The data points represent the averages ± SD of three independent experiments performed in triplicate. All data sets were analyzed by One-way ANOVA followed by Tukey's post-hoc test. **** p < 0,0001; *** p < 0,001; ** p < 0,01; * p < 0,05.

	**DPPH**	
**Sample**	**Concentration (mg/mL)**	**Mean ± SD (%)**
HH ctrl	0,1	89.43 ± 7.02
	0,5	70.06 ± 1.05 (**** vs HH 40 and HH 80)
	1	85.54 ± 1.65
	5	75.22 ± 0.24
HH 40	0,1	94.58 ± 3.49
	0,5	93.32 ± 1.98 (**** vs HH ctrl)
	1	88.89 ± 0.65
	5	79.79 ± 4.94 (* vs HH 80)
HH 80	0,1	95.30 ± 2.59
	0,5	93.21 ± 1.64 (**** vs HH ctrl)
	1	92.01 ± 1.20
	5	70.81 ± 3.45 (* vs HH 40)
	**FRAP**	
**Sample**	**Concentration (mg/mL)**	**Mean ± SD (%)**
HH ctrl	0,05	220.0 ± 15.32
	0,1	300.0 ± 20.13
	0,5	916.0 ± 24.00
	1	1432 ± 61.97 (* vs HH 80)
	2,5	1840 ± 13.06 (**** vs HH 80)
HH 40	0,05	208.0 ± 22.64
	0,1	320.0 ± 13.96
	0,5	916.0 ± 15.00
	1	1424 ± 13.06 (* vs HH 80)
	2,5	1764 ± 136.6 (** vs HH 80)
HH 80	0,05	216.0 ± 9.23
	0,1	316.0 ± 8.00
	0,5	912.0 ± 18.48
	1	1328 ± 26.13 (* vs HH 40 and vs HH ctrl)
	2,5	1652 ± 52.86 (** vs HH 40; **** vs HH ctrl)
	**ABTS**	
**Sample**	**Concentration (mg/mL)**	**Mean ± SD (%)**
HH ctrl	0,05	80.07 ± 1.81 (**** vs HH 40)
	0,1	70.06 ± 1.05 (**** vs HH 40)
	0,5	46.36 ± 1.51
	1	39.16 ± 1.20

	2,5	32.13 ± 1.55 (*** vs HH 40)
HH 40	0,05	85.16 ± 0.88 (**** vs HH ctrl; **** vs HH 80)
	0,1	75.50 ± 0.90 (**** vs HH ctrl; **** vs HH 80)
	0,5	48.46 ± 0.57 (**** vs HH 80)
	1	41.79 ± 0.70 (** vs HH 80)
	2,5	36.35 ± 2.10 (*** vs HH ctrl; ** vs HH 80)
HH 80	0,05	77.61 ± 0.90 (**** vs HH 40)
	0,1	67.25 ± 1.20 (**** vs HH 40)
	0,5	43.55 ± 0.99 (**** vs HH 40)
	1	37.75 ± 1.55 (** vs HH 40)
	2,5	32.84 ± 1.20 (** vs HH 40)

Table S3. *In vitro* ACE inhibitory activity. Evaluation of *in vitro* ACE inhibitory activity of the HHs: results of % ACE inhibition of HH ctrl, HH40 and HH80 concentrations (µg/ml) vs control. Data are represented as the means ± SD of six independent experiments, performed in triplicate. Statistical analysis was performed by one-way ANOVA, followed by Tukey's post-hoc test. **** p < 0,0001; *** p < 0,001; ** p < 0,01; * p < 0,05.

ACE inhibitory activity (%)		
**Sample**	**Concentration (µg/mL)**	**Mean ± SD (%)**
HH ctrl	86	5.10 ± 0.17 (* vs HH 40; **** vs HH 80)
	173	12.84 ± 0.22 (**** vs HH 80)
	345	28.46 ± 0.32 (**** vs HH 40 and vs HH 80)
	690	39.13 ± 0.23 (**** vs HH 40 and vs HH 80)
	1035	56.81 ± 0.10 (**** vs HH 80)
HH 40	86	3.40 ± 0.26 (* vs HH ctrl; **** vs HH 80)
	173	13.76 ± 0.95 (**** vs HH 80)
	345	19.53 ± 0.23 (**** vs HH ctrl and vs HH 80)
	690	49.32 ± 0.01 (**** vs HH ctrl and vs HH 80)
	1035	56.88 ± 0.002 (**** vs HH 80)
HH 80	86	11.73 ± 0.22 (**** vs HH 40 and vs HH ctrl)
	173	24.49 ± 0.82 (**** vs HH 40 and vs HH ctrl)
	345	39.26 ± 0.62 (**** vs HH 40 and vs HH ctrl)
	690	60.62 ± 0.75 (**** vs HH 40 and vs HH ctrl)
	1035	68.36 ± 0.57 (**** vs HH 40 and vs HH ctrl)

Table S4. *In vitro* DPP-IV inhibitory activity. Evaluation of *in vitro* DPP-IV inhibitory activity of the HHs: results of % of inhibition of the activity of human recombinant DPP-IV. Data are represented as the means ± SD of six independent experiments, performed in triplicate. Statistical analysis was performed by one-way ANOVA, followed by Tukey's post-hoc test. **** p < 0,0001; *** p < 0,001; ** p < 0,01; * p < 0,05.

DPP-IV inhibitory activity (%)		
**Sample**	**Concentration (mg/mL)**	**Mean ± SD (%)**
HH ctrl	0,5	11.97 ± 1.26
	1	39.25 ± 1.92 (* vs HH 40)
	2.5	72.72 ± 0.53 (** vs HH 40)
HH 40	0,5	13.48 ± 8.65
	1	28.44 ± 6.01 (* vs HH ctrl; ** vs HH 80)
	2.5	52.92 ± 0.49 (** vs HH ctrl; * vs HH 80)
HH 80	0,5	21.20 ± 4.51
	1	41.52 ± 5.46 (** vs HH 40)
	2.5	57.68 ± 1.84 (* vs HH 40)

Table S1. Peptides Sequences identified in HH ctrl, HH40 and HH80.

Sequence	Master Protein Accessions	Theo. MH+ [Da]	HH ctrl	HH40	HH80	Confidence (by Search Engine): Sequest HT	m/z [Da] (by Search Engine): Sequest HT	RT [min] (by Search Engine): Sequest HT	XCorr (by Search Engine): Sequest HT
NRDGIISKDDLRDVLATM	Q66I73	2032,05	5,66E+04	4,60E+03	3,37E+04	High	508,76898	86,4855	3,16
TALEEAEGTLEHEESKILR	Q90339	2155,09	4,25E+04	4,81E+03	1,50E+05	High	539,52869	88,3888	3,56
AFTIIDQNRDGIISKDDLRDVLATM	Q66I73	2820,46	1,67E+06	4,82E+03	4,17E+05	High	705,87091	108,4613	3,41
IQTALEEAEGTLEHEESKIL	Q90339	2240,13	4,62E+05	5,94E+03	1,80E+05	High	747,38232	105,3201	2,66
DLSRELEEISERL	Q90339	1588,82	1,07E+05	6,20E+03	2,86E+04	High	530,27875	109,0254	2,87
GVGIISEGNETVEDIAARL	P25489	1943,01	4,96E+05	6,83E+03	1,63E+05	High	972,01019	111,7825	3,24
ERINRTHHFSTTPVQLAQIMVMSKA	Q8AXB3	2927,50	2,00E+07	7,05E+03	#DIV/0!	High	586,30884	90,2972	3,46
LGTHECLRGNSRALSTVTAIIDGTGSIGA	Q7SY29	2927,50	2,00E+07	7,05E+03	#DIV/0!	High	586,30884	90,2972	3,4
QAPAGIQGSKGSVSGNHGVKANQISPGNPGL	Q67FY3	2927,51	2,00E+07	7,05E+03	#DIV/0!	High	586,30841	89,3833	2,71
SAALTASSQKQTMVISAGGSSVAPGPGPVAVT	Q7ZUV7	2927,51	2,00E+07	7,05E+03	#DIV/0!	High	586,30872	90,4129	4,35
LEQIGELGRGAYGVVDKMRHVPSGVIM	Q9DGE0	2927,52	2,00E+07	7,05E+03	#DIV/0!	High	586,30841	89,3833	2,82
DLTDYLMKIL	P83750	1224,65	8,09E+05	7,41E+03	2,49E+05	High	612,83228	120,2424	2,23
VEKQNPSIQPVPMLEVDPTVFEKRF	Q09178	2927,53	3,32E+07	8,02E+03	1,89E+07	High	586,30798	90,4539	3,75
TERGYSFVTTAEREIVRDIKE	P53479	2499,28	1,29E+05	8,48E+03	7,74E+04	High	625,57758	83,0174	2,7
EIVAINDPFIDL	Q5XJ10	1358,72	8,37E+04	9,24E+03	1,12E+04	High	679,86548	117,3382	1,96
DKSGFIEEEELKLFLQ	P05939	1924,99	1,69E+05	9,81E+03	3,09E+04	High	642,33679	107,6209	2,75
TALEEAEGTLEHEESKILRVQ	Q90339	2382,21	2,43E+05	9,98E+03	1,97E+05	High	596,31122	95,5521	4,25
NVLSGGTTMYPGIADRMQKEITALAPSTM	P53479	3053,51	6,25E+06	1,01E+04	1,34E+06	High	1018,51074	111,9638	3,14
KLEKTIDDLEDELYAQ	P13104	1922,96	1,65E+05	1,08E+04	9,64E+04	High	641,65924	83,084	3,58
KDRIEYVDAGTPITNQHYIAAPRGEI	Q5BLE8	2927,50	2,00E+07	1,11E+04	6,49E+06	High	586,30792	90,373	3,85
VFLTMFGEKLKGADPEDVIVS	O93409	2295,19	4,75E+05	1,17E+04	1,50E+05	High	765,73828	102,1018	2,89
LSPVHVFVSDNYQRPPIDAVL	A0A0R4IES7	2366,25	2,77E+05	1,19E+04	7,66E+04	High	592,32123	77,627	2,78
LDLAGRDLTDYLMKIL	P83750	1850,01	5,84E+06	1,29E+04	3,07E+05	High	617,34235	117,6008	3,26
LTTDKEAPGLVRMHTLAYLSGFPASL	Q5RGJ8	2788,47	6,89E+06	1,29E+04	1,41E+06	High	697,87775	113,2014	4,66
TIIDQNRDGIISKDDLRDVLATM	Q66I73	2602,35	3,54E+05	1,33E+04	1,37E+05	High	868,12335	98,354	3,05
FFIPVAGLTGFHVVLVAR	Q2THW0	1943,13	5,93E+05	1,65E+04	2,09E+04	High	648,38373	85,8264	2,3
FTIIDQNRDGIISKDDLRDVL	O93409	2446,29	2,86E+05	1,67E+04	8,57E+04	High	612,33057	90,2855	3,63
QELVDASERVGLL	Q90339	1428,77	5,57E+04	1,73E+04	5,95E+04	High	714,89001	78,9124	2,05
ADLSRELEEISERL	Q90339	1659,85	1,78E+05	1,82E+04	1,01E+05	High	553,95813	103,0955	2,83

NQYHITNSVERWSLQGGPWRSTAPL	Q3ZB90	2897,44	7,01E+06	2,00E+04	3,90E+05	High	966,48846	115,7849	2,59
GKSQLGGQQDVTAAACQPHIPVGERRH	Q1LV19	2897,45	7,01E+06	2,00E+04	3,90E+05	High	966,48914	115,7834	2,42
GTTASAAAAAAAAAAAAAAAAAAAGVSGSVAGSGTVPAAA	Q7SYL3	3054,55	3,47E+05	2,01E+04	1,24E+05	High	611,71686	73,9304	2,78
EAFTIIDQNRDGIISKDDLRDVLA	O93409	2717,41	4,03E+05	2,03E+04	1,29E+05	High	680,10864	94,5547	3,44
AFRVPVADVSVVDL	Q5MJ86	1486,83	1,23E+05	2,13E+04	5,12E+04	High	743,91833	100,5368	2,15
SSLEKSYELPDGQVITI	P83750	1878,97	1,39E+05	2,21E+04	7,44E+04	High	939,99103	104,8846	2,07
AGRDLTDYLMKIL	P83750	1508,81	6,93E+05	2,27E+04	2,80E+05	High	503,61096	110,3839	3,3
TKLEQQVDDLEGSLEQEKKLRM	Q90339	2617,35	2,56E+05	2,31E+04	2,34E+05	High	655,09363	77,8492	2,86
APPERKYSVWIGGSIL	P83750	1772,97	6,52E+05	2,35E+04	1,12E+05	High	591,66284	79,8807	2,82
AISEELDHALNDMTSI	P13104	1758,82	3,27E+05	2,69E+04	2,76E+05	High	879,91541	106,6409	2,21
LEQTERGRKVAEQELVDASERVGLLH	Q90339	2962,57	1,80E+05	2,75E+04	1,37E+05	High	593,3194	72,9885	3,76
SLEKSYELPDGQVITIGNERFR	P83750	2551,32	2,44E+05	3,05E+04	8,40E+04	High	638,58533	80,548	4,27
SAHGLYLGPGGAGPTSLTVATMLQRTEE	Q1LWL6	2814,41	4,12E+05	3,16E+04	6,59E+04	High	563,69073	77,262	3,04
KEAFTIIDQNRDGIISKDDLRDVLAT	Q66I73	2946,55	4,62E+05	3,19E+04	2,47E+05	High	737,39441	85,4358	4,09
KVAEQELVDASERVGLL	Q90339	1856,01	1,46E+05	3,19E+04	7,52E+04	High	619,34296	82,8832	3,06
MGKIIFYEDRNFQGRSY	P28022	1992,99	1,12E+05	3,21E+04	#DIV/0!	High	499,00528	53,7346	2,64
GKIIFYEDRNFQGRSY	P28022	1992,99	1,12E+05	3,21E+04	#DIV/0!	High	499,00528	53,7346	2,64
TKLEQQVDDLEGSLEQEKKLRMD	Q90339	2732,38	2,06E+05	3,25E+04	1,55E+05	High	683,85101	77,3357	4,97
DKSGFIEEEELKLF	P05939	1683,85	4,30E+05	3,53E+04	1,90E+05	High	561,95538	95,3272	2,62
ELPDGQVITIGNERF	P83750	1687,86	1,11E+05	3,54E+04	6,61E+04	High	844,43689	88,6452	2,07
AANLDKKQRNFDKVLAEWKQK	Q90339	2530,39	1,70E+05	3,73E+04	3,25E+05	High	506,88547	42,9484	4,08
ITQVSDLSTGFGNLLSSPPKCV	Q502L1	2320,19	1,03E+06	3,82E+04	1,17E+05	High	580,80536	73,1997	2,65
LGEQIDNLQRVKQKLEKEKSEYK	Q90339	2803,53	1,01E+05	3,87E+04	8,46E+04	High	468,09525	53,8562	2,76
IVPIVEPEILPDGDHDLK	P53448	1999,07	7,78E+04	3,98E+04	4,41E+04	High	667,03088	82,0519	2,33
KAKTKLEQQVDDLEGSLEQE	Q90339	2288,16	3,01E+05	4,03E+04	1,31E+05	High	763,39465	73,4361	3,51
DLEDALQRAKQDMAR	Q6NWF6	1759,88	5,29E+04	4,13E+04	#DIV/0!	High	440,72586	68,9221	2,6
IIDQNRDGIISKDDLRDVLAS	O93409	2356,25	1,01E+05	4,16E+04	6,99E+04	High	786,08929	73,1345	2,67
AFTIIDQNRDGIISKDDLRDVLAT	Q66I73	2689,42	7,91E+05	4,43E+04	4,50E+05	High	673,11108	93,1279	2,82
SGGTTMYPGIADRMQKEITSL	P83750	2256,10	3,26E+05	4,47E+04	1,86E+05	High	752,70642	92,0518	3,37
NAGEVVIGDGGFVF	Q32LQ4	1380,68	1,84E+05	4,67E+04	1,42E+05	High	690,84467	112,1162	2,32
IEKVAL	Q6DHU8	672,43	#DIV/0!	4,82E+04	8,30E+06	High	336,71869	40,3031	2,12
GWVIGEHGDSSVPVWSGVN	Q9W7K5	1981,94	1,55E+05	5,03E+04	8,61E+04	High	991,47693	90,9663	2,11
GGNVGDGGGAADRVINQIL	Q7ZU99	1782,91	1,72E+05	5,07E+04	9,95E+04	High	891,96027	86,3625	1,92

KVEIVAINDPFIDL	Q5XJ10	1585,88	9,55E+05	5,09E+04	2,62E+05	High	793,44696	111,4968	3,37
SYELPDGQVITIGNERFR	P83750	2094,06	2,82E+05	5,14E+04	1,39E+05	High	698,69403	82,4718	3,2
AFTIIDQNRDGIISKDDLRDVL	O93409	2517,33	8,05E+05	5,15E+04	3,00E+05	High	630,08923	92,4598	3,66
AVRNDEELNKLLGGVTIA	Q7ZUY3	1912,05	3,26E+05	5,26E+04	1,51E+05	High	638,02289	86,7022	3,26
SPTSSTPLRTTSTPLPKPNRDSGQGGVSV	A0A8M9QN1(	2924,51	3,27E+06	5,43E+04	1,13E+06	High	975,51031	113,2514	2,81
KEAFTIIDQNRDGIISKDDLRDVL	O93409	2774,47	9,58E+05	5,60E+04	2,44E+05	High	694,37494	84,4322	5,44
GIITNWDDMEKIWHH	P53479	1894,89	4,38E+05	5,63E+04	2,46E+05	High	632,30261	91,2909	3,38
IITNWDDMEKIWH	P53479	1700,81	3,36E+05	5,70E+04	5,04E+04	High	567,60944	92,1389	2,88
LTTDKEAPGLVRMHTLAYLSGFPAS	Q5RGJ8	2675,39	8,37E+05	5,72E+04	4,35E+05	High	669,60657	92,55	3,14
AFTIIDQNRDGIISKDDLRDVLAS	O93409	2675,40	8,37E+05	5,72E+04	4,35E+05	High	669,60687	92,5408	2,63
AKRVTIMPKDIQLARRIRGERA	Q6PI20	2578,52	8,17E+05	5,85E+04	5,87E+05	High	430,59418	36,2248	3,93
DDMEKIWHHTFYNEL	P83750	1977,88	2,98E+05	5,88E+04	1,48E+05	High	659,96619	87,0185	3,04
ETDHRMVVPMESPVRIL	O78682	2009,03	7,09E+04	5,91E+04	6,50E+04	High	670,35065	74,1838	2,74
ELEEELKTVTNNMKSL	P13104	1877,95	1,87E+05	6,14E+04	3,91E+05	High	626,65704	89,3521	2,62
RAALEQTERGRKVAEQELVDASE	Q90339	2585,33	1,41E+05	6,17E+04	1,87E+05	High	647,08807	45,3862	2,63
GPPGLPGPPGIP	C7DZK3	1055,59	4,31E+04	6,22E+04	7,08E+04	High	528,29901	81,8557	2,03
GPPGPPGLPGLP	A5PMY6	1055,59	4,31E+04	6,22E+04	7,08E+04	High	528,29871	81,8188	2,08
GVMVGMGQKDSYVGDEAQSKRGILTL	P83750	2739,38	3,70E+05	6,30E+04	1,37E+05	High	685,60144	72,3219	4,98
KSYELPDGQVITIGNERF	P83750	2066,06	4,81E+05	6,40E+04	1,99E+05	High	689,3576	88,4824	3,79
VEKQRADLSRELEEISERLEE	Q90339	2558,31	6,57E+05	6,52E+04	5,74E+05	High	640,3338	84,5303	3,83
RVVVSAPSPDAPMFVM	Q5MJ86	1702,87	1,77E+05	6,81E+04	1,90E+05	High	851,93866	89,2335	2,85
KALSDHHVYLEGTLLKPN	Q8JH70	2035,10	1,12E+05	6,90E+04	1,43E+05	High	679,03949	47,9053	2,98
GEQIDNLQRVKQKLEKEKSEYKM	Q90339	2821,49	2,41E+05	6,93E+04	2,98E+05	High	706,12817	56,8043	3,05
ASSSSLEKSYELPDGQVITIGNE	P83750	2424,18	4,01E+05	7,00E+04	1,38E+05	High	808,73254	93,5051	3,17
IKGGAKRVIISAPSADAPMFVM	Q5XJ10	2259,24	2,47E+05	7,06E+04	1,93E+05	High	565,5661	72,037	2,77
KDVKKSPLIETTTTGDNNQSVKPLK	Q6DRG7	2741,50	9,61E+04	7,14E+04	1,67E+05	High	457,75671	31,6601	2,67
SSSSLEKSYELPDGQVITIGNERFR	P83750	2812,41	5,42E+05	7,30E+04	1,69E+05	High	703,86102	80,2642	4,73
LGRIPSAVGYQPTLATDM	Q9PTY0	1889,98	2,01E+05	7,48E+04	9,79E+04	High	945,49756	81,341	2,11
RVAPEEHPTLLTEAPLNPKANRE	P53479	2582,37	1,52E+05	7,52E+04	1,28E+05	High	646,34845	45,8927	5,56
QVDDLEGSLEQEKKLRMDLE	Q90339	2375,18	4,11E+05	7,73E+04	1,71E+05	High	594,55145	73,13	4,76
GDDAPRAVFPSIVGRPRHQGVMVGM	P83750	2649,35	4,08E+05	7,78E+04	2,92E+05	High	663,09473	66,9152	3,4
RADLSRELEEISERL	Q90339	1815,96	7,41E+05	7,78E+04	4,91E+05	High	605,99164	89,3705	3,92
VGDEAQSKRGILTL	P83750	1486,82	6,62E+04	7,82E+04	2,46E+05	High	496,28036	47,6147	2,73

EPKRNRRSRSRSGSRRDRGGSPPDRT	Q6P5L7	3023,59	#DIV/0!	7,86E+04	1,02E+05	High	504,77539	43,2939	2,61
NVLSGGTTMYPGIADRMQKEITAL	P53479	2582,30	1,31E+06	7,91E+04	6,38E+05	High	861,43719	102,1842	2,91
GIVPIVEPEILPDGDHDLK	P53448	2056,10	1,78E+05	7,96E+04	1,11E+05	High	686,039	88,5384	2,36
EGSLEQEKKLRMDLERAKRKLE	Q90339	2686,47	3,25E+05	8,19E+04	2,94E+05	High	538,10071	48,3761	2,75
KGGAKRVIISAPSADAPMF	Q5XJ10	1916,04	1,17E+05	8,27E+04	#DIV/0!	High	479,76709	54,98	3
TFYNELRVAPEE	P83750	1467,71	1,10E+05	8,44E+04	7,39E+04	High	734,36206	67,0366	2,73
YVVESTGVFTTIEKA	Q5XJ10	1643,85	2,20E+05	8,65E+04	1,41E+05	High	822,43256	80,6784	2,24
DDAPRAVFPSIVGRPR	P83750	1752,95	6,68E+04	8,72E+04	8,10E+04	High	438,99496	56,004	3,19
RANLENQIAEAE	Q6NWF6	1357,67	6,39E+04	8,76E+04	8,56E+04	High	679,33984	42,7561	2,04
KQRADLSRELEEISERLEE	Q90339	2330,19	2,99E+05	8,79E+04	2,38E+05	High	583,30627	80,3869	3,3
ELGEQIDNLQRVKQKLE	Q90339	2040,11	1,52E+05	8,94E+04	1,57E+05	High	680,71027	62,4624	2,93
AKRVIISAPSADAPMF	Q5XJ10	1673,90	1,89E+05	9,40E+04	3,09E+04	High	558,64099	64,2863	3,02
NVLSGGTTMYPGIADRMQ	P53479	1910,91	1,65E+05	9,48E+04	1,17E+05	High	955,96021	77,0622	3,21
EKTIDDLEDELYA	P13104	1553,72	2,69E+05	9,70E+04	#DIV/0!	High	777,36633	84,1292	1,91
VIISAPSADAPMFVM	Q5XJ10	1548,78	3,48E+05	9,94E+04	4,55E+05	High	774,89557	113,28	2,02
GFAGDDAPRAVFPSIVGRPRHQGV	P83750	2506,31	5,05E+05	9,96E+04	2,07E+05	High	502,06812	58,9762	3,6
KEAFTIIDQNRDGIISKDDLRDVLAS	O93409	2932,54	7,84E+05	9,96E+04	3,53E+05	High	733,89221	85,0028	3,34
FQPSFIGMESAGIHET	P53479	1750,81	2,04E+05	1,02E+05	1,23E+05	High	875,90784	82,464	2
NVKNEELEAMVK	Q66I73	1403,72	6,47E+04	1,04E+05	1,28E+05	High	702,36633	38,7145	2
YHSALTGGNLRSDQGKTVSGIPSFML	E7FGT5	2752,37	2,02E+05	1,04E+05	2,99E+05	High	551,28101	43,1499	2,66
EQELVDASERVGLL	Q90339	1557,81	2,58E+05	1,04E+05	2,43E+05	High	779,41187	83,5105	2,66
IIDQNRDGIISKDDLRD	O93409	1986,03	1,33E+05	1,05E+05	7,66E+04	High	497,26285	43,3906	2,94
NGPAPWGFRLQ	Q6P7E4	1242,64	1,30E+05	1,05E+05	1,82E+05	High	621,82446	77,7428	2,2
TEAPLNPKANR	P83750	1210,65	4,68E+04	1,05E+05	8,25E+04	High	404,22418	16,8616	2,6
AFRVPTPNVSVVDLTVR	Q5XJ10	1870,05	6,85E+05	1,05E+05	4,74E+05	High	624,0246	86,0045	3,68
DLAGRDLTDYLMKIL	P83750	1752,92	2,25E+06	1,07E+05	1,48E+06	High	584,97992	114,877	3,27
KYLYAFNIIPM	Q5RGJ5	1372,73	9,26E+04	1,08E+05	2,11E+05	High	686,86963	69,7041	1,91
GWLDKNKDPLNDSVVQLY	Q90339	2104,07	5,43E+05	1,08E+05	3,76E+05	High	702,03003	92,5696	2,68
RVAPEEHPTLLTEAPLNPKAN	P53479	2297,22	1,22E+05	1,09E+05	8,70E+04	High	575,06464	52,9322	3,12
KSYELPDGQVITIGN	P83750	1633,84	3,41E+05	1,10E+05	2,51E+05	High	817,42694	95,2209	2,42
NFDKVLAEWK	Q90339	1249,66	1,03E+05	1,10E+05	8,55E+04	High	625,3335	65,7609	2,26
DFHLLPSGIIN	Q568F6	1225,66	2,38E+05	1,11E+05	1,32E+05	High	613,33417	100,417	1,99
DLEGSLEQEKKLRMDLE	Q90339	2033,02	3,02E+05	1,13E+05	2,07E+05	High	678,34723	71,0479	3,1

GIISKDDLRDVLA	O93409	1414,79	2,39E+05	1,14E+05	2,17E+05	High	472,26962	71,0417	2,92
LVTWYDNEFGYSNR	Q5XJ10	1763,80	2,31E+05	1,15E+05	1,45E+05	High	882,40643	83,3158	3,11
RIEELEEEIE	Q90339	1288,63	1,28E+05	1,15E+05	1,47E+05	High	644,81915	58,8295	2,08
NVLSGGTTMYPGIADRMQKEITA	P53479	2453,22	7,85E+05	1,16E+05	2,23E+05	High	818,41284	84,0873	2,68
EISDLTEQLGETGKSIHEL	Q90339	2099,05	3,19E+05	1,16E+05	2,51E+05	High	700,35773	83,3471	3,38
ASLSTFQQMWITKQ	P53479	1668,84	2,23E+05	1,17E+05	1,56E+05	High	834,92712	89,8552	2,57
DDAPRAVFPSIVGRPRHQGVMVGM	P83750	2592,33	4,46E+05	1,17E+05	3,86E+05	High	648,84003	69,7737	3,04
EGNETVEDIAARL	P25489	1416,70	1,34E+05	1,18E+05	1,11E+05	High	708,85345	82,6521	2,22
WITKQEYDEAGPSIVHRK	P53479	2157,11	3,21E+05	1,19E+05	2,74E+05	High	432,22949	41,3511	3,16
LRAEPPTLCSRQKHSRPYSELTIMSL	Q9YHZ7	3070,59	2,34E+05	1,21E+05	4,62E+05	High	512,60968	40,0946	3,22
AGFAGDDAPRAVFPSIV	P83750	1689,86	1,79E+05	1,21E+05	1,42E+05	High	845,43457	93,8145	2,6
ELEEEIEAERAARA	Q90339	1615,79	1,24E+05	1,21E+05	3,32E+05	High	539,27026	49,856	2,96
AVSEGTKAVTKYTSSK	Q6PC60	1656,88	1,44E+05	1,22E+05	1,70E+05	High	552,96729	22,1293	3,06
TIIDQNRDGIISKDDLRDVLAS	O93409	2457,29	6,03E+05	1,23E+05	3,60E+05	High	819,77081	80,214	3,57
DKENALDRAEQ	P13104	1288,61	6,29E+04	1,23E+05	1,31E+05	High	644,81128	19,0639	2,11
DAIKKKMQM	P13104	1092,59	6,96E+04	1,23E+05	1,65E+05	High	546,80005	57,7853	2
MDAIKKKMQM	P13104	1092,59	6,96E+04	1,23E+05	1,65E+05	High	546,80005	57,7853	2
IDVSKAACILTTQTLMKTLRSKEAAA	Q6NVJ5	2836,53	2,91E+05	1,23E+05	2,86E+05	High	473,5983	39,4491	2,68
ETVLEISHLPRLERLNLSSTSLSEI	Q5U378	2836,54	2,91E+05	1,23E+05	2,86E+05	High	473,5983	39,4491	2,63
VEKVSFTGSVPTGKKIMEMASRGVKAV	Q802W2	2836,54	2,91E+05	1,23E+05	2,86E+05	High	473,5983	39,4491	2,66
NLLKPMGKLISYGAANMLAGQKKNLF	Q8JFV8	2836,56	2,91E+05	1,23E+05	2,86E+05	High	473,59827	39,2889	2,66
RIPSAVGYQPTLATDM	Q9PTY0	1719,87	1,93E+05	1,23E+05	1,33E+05	High	860,4433	73,0677	2,46
LNELPTVETEPLPHAVLQSFSSQFEK	B2ZFP3	2940,50	5,19E+05	1,24E+05	2,38E+05	High	588,901	72,6308	2,61
FFCWGPALLLATMMLVKPDVIEGKMG	A2BGS3	2940,49	1,09E+06	1,24E+05	8,49E+05	High	588,89978	75,0035	3,3
LEQTERGRKVAEQELVDASERVGLL	Q90339	2825,51	1,93E+06	1,29E+05	1,09E+06	High	565,90997	79,4576	4,55
LEKSYELPDGQVITIGNE	P83750	2005,01	1,10E+06	1,29E+05	4,35E+05	High	1003,01404	96,8727	2,19
SGAGKTVNTKRVIQYF	Q90339	1768,97	2,14E+05	1,30E+05	2,77E+05	High	590,3288	42,709	2,47
SVLDPDEGIRF	Q7ZVY5	1247,63	1,48E+05	1,31E+05	5,83E+04	High	624,31793	79,5925	2,21
AELGEQIDNLQRVKQKLE	Q90339	2111,15	2,58E+05	1,31E+05	2,04E+05	High	528,54395	65,7714	2,86
LERAKRKLEGDLKL	Q90339	1669,01	1,85E+05	1,31E+05	3,05E+05	High	418,01001	26,2622	2,68
IADIMRAL	Q6P0G6	902,51	9,88E+04	1,32E+05	2,14E+05	High	451,76083	70,3869	2,16
GYALPHAIMRL	P53479	1241,68	1,70E+05	1,33E+05	1,97E+05	High	621,34644	69,6626	2,68
KQRADLSRELEEISER	Q90339	1959,03	1,56E+05	1,35E+05	1,82E+05	High	490,51367	48,3311	3,2

ILTTMLATRNFSSKNPYKKPDGVKES	Q6DEH3-2	2925,55	1,98E+05	1,38E+05	3,15E+05	High	585,91266	57,485	2,79
LQPPPPPPPHPASAASLQHHGQFPPLLP	Q6NYU2	2925,55	1,98E+05	1,38E+05	3,15E+05	High	585,91205	57,6848	2,63
TSPPLQLTASPASPILMPVSSPVSPPSPGL	Q1ECW2	2925,56	1,98E+05	1,38E+05	3,15E+05	High	585,91339	57,5239	3,7
TKLEQQVDDLEGSL	Q90339	1574,79	3,69E+05	1,39E+05	2,49E+05	High	787,90009	80,958	2,08
VGDEAQSKRGILT	P83750	1373,74	6,52E+04	1,41E+05	1,43E+05	High	687,37433	25,6554	2,04
DIVLVGGSTRIPKIQ	Q90473	1595,95	1,58E+05	1,41E+05	1,48E+05	High	532,65546	71,9392	2,54
NRDGIISKDDLRDVL	O93409	1728,92	2,33E+05	1,42E+05	1,08E+05	High	576,98065	63,2769	3,61
AGFAGDDAPRAVFPSIVGRPR	P83750	2156,14	1,72E+05	1,42E+05	1,45E+05	High	539,79102	66,7993	3,25
DIDFGTYPFVTS	Q568F6	1361,63	5,06E+05	1,44E+05	3,21E+05	High	681,31848	114,2868	1,93
TALEEAEGTLEHEES	Q90339	1644,72	1,13E+05	1,44E+05	1,19E+05	High	822,86755	68,8941	2,09
APLNPKANREKMTQIMF	P83750	1989,04	3,65E+05	1,46E+05	6,60E+05	High	663,68768	51,9882	2,83
APRAVFPSIVGRPRHQ	P83750	1788,01	2,71E+05	1,46E+05	4,68E+05	High	447,76028	34,3651	3,57
FAGDDAPRAVFPSIVGRPRHQGVMVG	P83750	2736,42	1,69E+06	1,46E+05	3,82E+05	High	684,86163	65,1559	2,7
GDDAPRAVFPSIVGRPRHQ	P83750	2075,09	1,18E+05	1,46E+05	1,74E+05	High	415,82501	43,416	3,29
WVIGEHGDSSVPVWSGV	Q9W7K5	1810,88	4,95E+05	1,46E+05	2,15E+05	High	905,94366	94,8124	2,6
KPLNITIEKMGN	F1R237	1373,75	1,46E+05	1,48E+05	6,78E+05	High	687,37189	97,9268	2,04
NFDKVLAEWKQKYEE	Q90339	1926,96	4,49E+05	1,48E+05	2,08E+05	High	642,99231	74,8118	3,49
FAGDDAPRAVFPSIVGRP	P83750	1871,98	4,01E+05	1,50E+05	1,18E+05	High	624,6651	74,7668	4,71
LKGYTSWAIGMSVADL	Q9PVK5	1711,87	6,23E+05	1,50E+05	3,39E+05	High	571,29468	75,8257	2,32
SEELDHALNDMTSI	P13104	1574,70	2,57E+05	1,51E+05	4,42E+05	High	787,85541	84,9753	3,2
KERYAAW	Q90339	923,47	7,71E+04	1,51E+05	1,52E+05	High	462,2417	25,207	1,91
VRNDNSSRFGKFIRIH	Q90339	1946,05	1,55E+05	1,52E+05	1,64E+05	High	390,01675	35,4517	2,9
VLSGGTTMYPGIADRMQKEITAL	P53479	2452,26	2,79E+06	1,53E+05	7,85E+05	High	818,09222	97,2795	2,7
INKIIFKSGELQMSPYAEFPKSNRVS	Q6R8J2	2999,57	2,52E+05	1,53E+05	3,80E+05	High	600,71875	39,3448	2,85
MPVCVMMAELDEKLLLQF	Q7ZUZ0	2036,03	3,11E+06	1,54E+05	2,81E+05	High	1018,5174	114,2574	1,96
PVCVMMAELDEKLLLQF	Q7ZUZ0	2036,03	3,11E+06	1,54E+05	2,81E+05	High	1018,5174	114,2574	1,96
GAFSAMGMGHLLASVSGAGGLENGSLSAQGTGST	Q7ZTU9	3096,44	1,13E+05	1,54E+05	9,47E+04	High	620,09369	19,9735	2,77
DFPSPEWDTVTPEAK	Q6DEH3-2	1718,79	1,47E+05	1,54E+05	1,49E+05	High	859,90094	81,2218	2,19
LEQQVDDLEGSLEQ	Q90339	1602,75	2,33E+05	1,57E+05	1,92E+05	High	801,8808	75,8118	2,32
VAVPVLVR	Q90481	852,57	1,34E+05	1,58E+05	1,53E+05	High	426,78763	60,5821	1,92
ESRASSVSSAPVSI	Q6Q7X9	1376,70	9,08E+04	1,59E+05	1,52E+05	High	459,57355	30,2988	2,39
QTGDALGKALASI	A0A0R4IBL7	1244,68	3,24E+05	1,62E+05	3,18E+05	High	622,84723	89,3293	1,99
SIEQRLKWAGGANPALAP	C5J7W8	1879,02	3,86E+05	1,62E+05	1,80E+05	High	470,51147	58,2229	2,69

LTTQTLMKTLRSKEAAA	Q6NVJ5	1879,03	3,86E+05	1,62E+05	1,80E+05	High	470,51147	58,2229	2,68
SYELPDGQVITIGNER	P83750	1790,89	1,48E+05	1,63E+05	1,14E+05	High	895,953	83,9959	2,14
RSDKRKDRVEISPEQL	A0A8M3B525	1956,06	4,23E+05	1,64E+05	3,02E+05	High	652,69263	76,9772	2,38
NVLSGGTTMYPGIADRMQKEIT	P53479	2382,18	7,45E+05	1,64E+05	3,22E+05	High	794,73303	80,228	2,58
RDYIWNTL	Q7ZVY5	1080,55	2,14E+05	1,66E+05	1,66E+05	High	540,77844	78,5105	2,1
KTVDGPSGKLWRDGRGASQNIIPA	Q5XJ10	2523,34	3,03E+05	1,67E+05	2,52E+05	High	631,5932	51,3972	3,19
RAARAKVEKQRADLSRE	Q90339	1984,12	2,00E+05	1,69E+05	1,43E+05	High	397,63092	10,751	4,89
NNRFASFIDKVR	Q6NWF6	1466,79	1,25E+05	1,71E+05	7,75E+04	High	489,6015	51,5349	3,17
GENTVRTIAMDGTE	Q9PTY0	1493,69	9,34E+04	1,72E+05	9,98E+04	High	747,34949	44,5324	2,54
GAAKAVGKVIPE	Q5MJ86	1139,68	1,04E+05	1,73E+05	1,01E+05	High	570,34491	26,7035	1,92
EQTERGRKVAEQELVDA	Q90339	1957,99	1,56E+05	1,73E+05	1,59E+05	High	653,33783	32,3888	2,82
NSFVNDIFER	Q6PC60	1240,60	2,47E+05	1,75E+05	1,96E+05	High	620,80322	79,3763	2,39
VVESTGVFTTIEKA	Q5XJ10	1480,79	1,59E+05	1,75E+05	1,79E+05	High	740,89911	68,9475	2,08
GRIGRLVTR	Q5XJ10	1027,65	4,29E+04	1,75E+05	1,33E+05	High	343,22214	15,5504	2,39
QKDSYVGDEAQSKRGIL	P83750	1893,97	1,34E+05	1,75E+05	1,39E+05	High	631,99615	35,0389	2,94
LDSFVTPAVTRQSILNV	M1GL27	1860,02	3,16E+05	1,76E+05	4,28E+05	High	465,76349	51,9609	2,63
VPTPNVSVVDLTVR	Q5XJ10	1495,85	1,77E+05	1,76E+05	2,01E+05	High	748,4278	78,1775	2,79
DAGAGIALNDHFVKL	Q5XJ10	1540,81	2,54E+05	1,76E+05	2,88E+05	High	514,27679	77,2468	2,79
DIDIRKDLYANNVL	P53479	1661,89	5,36E+05	1,77E+05	3,45E+05	High	554,63464	85,8466	3,53
GQMNEPPGARAR	Q9PTY0	1283,63	3,55E+04	1,79E+05	8,50E+04	High	428,54822	14,646	2,32
FTIIDQNRDGIISKDDLRDVLA	O93409	2517,33	2,15E+06	1,79E+05	1,30E+06	High	630,08948	90,0055	4,62
MNGLAGPLHGL	Q7ZVY5	1079,57	2,02E+05	1,80E+05	1,96E+05	High	540,28857	68,5369	2,48
VDIGIPDATGRLE	Q7ZU99	1355,72	1,59E+05	1,81E+05	1,54E+05	High	678,3631	74,0721	2,09
VDETGVEVK	Q8JIU7	975,50	1,21E+05	1,81E+05	1,34E+05	High	488,25522	23,8811	2,19
SLQSGLLL	Q566X6	830,50	1,05E+05	1,83E+05	2,87E+05	High	415,7543	45,1522	2,11
EAFTIIDQNRDGIISK	O93409	1819,95	1,46E+05	1,84E+05	1,25E+05	High	607,32416	65,4101	2,39
ISYGAANMLAGQ	Q8JFV8	1195,58	1,53E+05	1,87E+05	1,67E+05	High	598,29309	45,5059	1,91
KEAFTIIDQN	O93409	1178,61	1,12E+05	1,87E+05	1,20E+05	High	589,80896	53,3506	1,97
DKKQRNFDKVLA	Q90339	1461,82	9,13E+04	1,87E+05	5,33E+05	High	487,94534	19,2772	2,62
WAAFPPDVAGNVDYK	O93409	1649,80	1,76E+05	1,87E+05	1,89E+05	High	825,40332	81,8325	2,53
GNETVEDIAARL	P25489	1287,65	1,66E+05	1,88E+05	1,80E+05	High	644,33179	78,4666	2,6
TIAMDGTEGLVRGQ	Q9PTY0	1447,72	1,10E+05	1,90E+05	1,60E+05	High	724,36688	57,3086	2,47
SLANVPPGEQRSRFLAVGLVDNTV	Q1LVE8	2539,36	4,55E+05	1,90E+05	4,44E+05	High	508,68094	43,6257	2,71

TVLSGGTTMYPGIADRM	P83750	1769,86	2,88E+05	1,92E+05	2,76E+05	High	885,435	78,2733	3,89
GFAGDDAPRAVFPSIVGR	P83750	1831,94	3,12E+05	1,92E+05	2,24E+05	High	611,32092	75,3551	3,44
AVRNDEELNKLLGGVT	Q7ZUY3	1727,93	4,08E+05	1,92E+05	2,35E+05	High	576,64948	72,1391	3,41
SKIQLEAKLK	Q90339	1157,73	1,32E+05	1,93E+05	3,73E+05	High	386,58167	24,1321	2,9
NITPVEVPFGGFKASGIGRENGQVTI	Q802W2	2687,42	1,52E+06	1,93E+05	3,89E+05	High	538,29303	68,5519	3,13
KPGWIGWTKRIEYTPGSGSIPLFP	A0A8M9PFP2	2687,43	1,52E+06	1,93E+05	3,89E+05	High	538,29309	68,6133	3,08
PRIMTGPTPVMPNAALRTPAPTAPTL	Q6P104-2	2687,44	1,52E+06	1,93E+05	3,89E+05	High	538,29303	68,5519	3,08
GAPIRIPVGPETL	Q9PTY0	1319,77	2,19E+05	1,94E+05	1,48E+05	High	660,38934	80,6819	2,2
GLTPPPGGAPGGGVAFCIESPPVL	Q2I6J1	2247,15	4,00E+05	1,94E+05	3,11E+05	High	450,23782	30,663	2,79
DEAQSKRGILTL	P83750	1330,73	1,02E+05	1,94E+05	3,61E+05	High	665,87128	47,136	2,55
EQIDNLQR	Q90339	1015,52	1,37E+05	1,95E+05	#DIV/0!	High	508,26389	26,0063	2,03
KVLKQVHPDTGISSK	Q6PC60	1636,94	1,72E+05	1,95E+05	2,08E+05	High	546,31842	15,9927	2,61
RNDNSSRFGKFIRIH	Q90339	1846,98	2,40E+05	1,98E+05	3,12E+05	High	462,50131	33,5983	3,49
GSGPRSGLGELILPE	Q7SX99	1481,80	2,17E+05	1,98E+05	1,20E+05	High	741,4032	79,2298	1,91
DEAGPSIVHR	P53479	1080,54	8,83E+04	1,98E+05	1,36E+05	High	540,77747	23,945	2,64
KNNNNDIVNAIM	Q8JIU7	1359,67	1,07E+05	1,98E+05	1,35E+05	High	680,33856	61,372	2,08
VREIAQDFKTDLRFQ	Q6PI20	1865,99	3,12E+05	1,99E+05	2,36E+05	High	622,66888	61,7121	3,04
DLEESTLQHE	Q90339	1200,54	9,31E+04	1,99E+05	8,34E+04	High	600,77399	41,5252	2,03
DAQLHLDEAVRGQ	Q90339	1451,72	1,08E+05	1,99E+05	#DIV/0!	High	484,58051	57,3498	2,4
ANLDKKQRNFDKVLA	Q90339	1759,98	1,00E+05	2,00E+05	4,74E+05	High	587,33356	26,862	2,59
TALEEAEGTLEH	Q90339	1299,61	1,12E+05	2,00E+05	1,84E+05	High	650,30835	58,1808	2,61
YVGDEAQSKRGILT	P83750	1536,80	1,26E+05	2,00E+05	2,47E+05	High	768,90533	34,8802	2,43
TQKTVDGPSGKLWRDGRGASQNIIPA	Q5XJ10	2752,45	3,39E+05	2,00E+05	3,37E+05	High	688,86902	51,7763	3,34
SGGTTMYPGIADRMQKEIT	P83750	2055,98	2,89E+05	2,01E+05	2,50E+05	High	686,00055	65,5445	2,31
ETDAIQRTEELEE	Q90339	1562,72	1,12E+05	2,01E+05	1,11E+05	High	781,86395	49,9589	2,85
KDIDDLELTLA	Q90339	1245,66	3,17E+05	2,01E+05	3,65E+05	High	623,33398	82,1631	2,75
LKKDIDDLELTLA	Q90339	1486,84	3,11E+05	2,01E+05	5,69E+05	High	496,28494	71,3157	2,33
IRPIYSNPPMNGARIA	Q7SYK7	1769,95	1,83E+05	2,01E+05	1,74E+05	High	590,65631	53,6617	3,05
AFRVPTPNVSVV	Q5XJ10	1285,73	1,92E+05	2,02E+05	2,53E+05	High	643,3678	76,4195	2,06
KKIKELQARIE	Q90339	1355,84	1,28E+05	2,03E+05	3,39E+05	High	452,61829	17,0834	2,51
DVNQQEFVRAL	Q90YQ4	1318,67	1,20E+05	2,03E+05	1,21E+05	High	659,84222	70,8652	2,74
RDLEESTLQHEAT	Q90339	1528,72	1,16E+05	2,04E+05	1,23E+05	High	510,24741	32,5906	2,75
WVIGEHGDSSVPVWSGVN	Q9W7K5	1924,92	5,71E+05	2,05E+05	2,59E+05	High	962,96545	88,602	2,29

EELEKSRDEQE	U3JAG9	1391,63	2,24E+05	2,05E+05	1,98E+05	High	696,31458	43,1625	1,91
VGSERNVLIFDLGGGTFDVSI	P47773	2195,13	1,54E+05	2,05E+05	2,31E+05	High	549,53772	36,6858	2,65
GGAKRVIISAPSAD	Q5XJ10	1341,75	1,56E+05	2,06E+05	1,15E+05	High	447,92258	29,4239	2,36
LIDLGIEDVSLAMQKSDK	B8JKP6	1991,04	9,54E+05	2,06E+05	3,65E+05	High	664,35498	75,5508	2,41
QALRGGSSGGGAGSPRSSLE	A1YB07	1830,91	3,38E+05	2,07E+05	2,60E+05	High	610,974	69,5706	2,3
RRDLEESTLQHEA	Q90339	1583,78	1,35E+05	2,07E+05	1,91E+05	High	528,59869	25,4006	2,31
EAEVEAEQRRGADAVKGVRKY	Q90339	2361,23	2,05E+05	2,07E+05	2,32E+05	High	591,06488	32,6036	3,15
KGQPGDAGEQGFPGVLGIFGPQGPPGDFGPV	A0MSJ1	2979,46	4,12E+05	2,07E+05	8,09E+05	High	497,42072	38,1762	2,87
NIINYFVTGGGGGGGGGGGGGGVAVTGGNGKAKSQ	Q5TZE2	2979,47	4,12E+05	2,07E+05	8,09E+05	High	497,42072	38,1762	2,87
SMPVASPGWTASPKAAMPSPGGPPSVKVTGN	Q67FY3	2979,47	4,12E+05	2,07E+05	8,09E+05	High	497,42072	38,1762	2,97
TRDKMNINHEGQTTVPPMITQPTLQL	A0MSJ1	2979,50	4,12E+05	2,07E+05	8,09E+05	High	497,41986	37,731	2,61
IIDQNRDGIISKDDLRDVLAT	Q66I73	2370,26	6,87E+05	2,08E+05	4,12E+05	High	593,32343	73,9468	3,85
KMIESLSKERVQAGDVITIDKATG	P83571	2589,39	6,26E+06	2,08E+05	2,45E+06	High	648,10767	95,5983	3,63
KLQPSII	Q503W7	798,51	9,03E+04	2,09E+05	4,89E+05	High	399,75888	51,5866	2,03
RANLENQIAEAEE	Q6NWF6	1486,71	2,03E+05	2,10E+05	1,97E+05	High	743,86249	46,427	2,75
LTDYLMK	P83750	883,46	1,38E+05	2,10E+05	1,33E+05	High	442,23465	45,8519	1,9
KPKEALEDGVITDKADIF	Q6DHU8	1989,05	5,86E+05	2,11E+05	9,10E+05	High	498,01724	51,9862	2,77
ETDAIQRTEE	Q90339	1191,55	9,77E+04	2,13E+05	9,98E+04	High	596,27966	20,2338	1,99
GKVIPELNGKLTGM	Q5MJ86	1456,82	1,47E+05	2,14E+05	2,71E+05	High	486,27957	59,9359	2,63
AGDDAPRAVFPSIVGRP	P83750	1724,91	2,65E+05	2,14E+05	1,16E+05	High	575,64294	65,6718	3,45
FQPSFIGMESAGIH	P53479	1520,72	2,61E+05	2,15E+05	1,74E+05	High	760,86511	83,8107	2,96
GVQDFLLLE	A5PF48	1033,56	7,99E+05	2,15E+05	2,73E+05	High	517,28326	112,8296	2,1
DAPRAVFPSIVGRPRHQGVMVGM	P83750	2477,30	8,77E+05	2,15E+05	5,67E+05	High	620,08258	66,8275	3,9
MDAIKKKMQMLK	P13104	1506,82	1,80E+05	2,21E+05	1,88E+06	High	502,94681	63,1443	3,68
AQSKRGILTL	P83750	1086,66	1,62E+05	2,21E+05	3,58E+05	High	543,83685	33,9195	2
RDGIISKDDLRDVLA	O93409	1685,92	3,18E+05	2,24E+05	3,39E+05	High	562,646	64,0597	4,46
DLTEQLGETGKSIHE	Q90339	1656,81	1,46E+05	2,24E+05	1,62E+05	High	828,90936	57,0697	2,71
NKVPSNQSSLPPPPPSMDFLPPPPP	Q6PFT9	2637,34	8,96E+05	2,24E+05	6,29E+05	High	528,26849	43,2515	3,17
YAIITGNGAASATPLTDFVDLQTER	Q6JAN0	2624,32	4,33E+05	2,25E+05	2,30E+05	High	525,66638	49,7065	2,77
ADRQDRMSKAKELFVLCDKEGK	A1A600	2624,33	4,33E+05	2,25E+05	2,30E+05	High	525,66742	49,7791	2,86
VEKQRADLSRELEEISER	Q90339	2187,14	3,48E+05	2,25E+05	3,57E+05	High	547,5412	52,9175	4,07
RVTIMPKDIQLAR	Q6PI20	1540,90	1,53E+05	2,26E+05	1,71E+05	High	514,30627	46,9283	2,98
TKLEQQVDDLEGSLEQE	Q90339	1960,93	1,11E+06	2,26E+05	7,09E+05	High	980,97369	83,675	2,1

MVDREQLVQ	Q6PC29	1028,54	1,13E+05	2,26E+05	1,60E+05	High	514,77429	51,5015	2,06
IIAPPERKYSVWIGGSIL	P83750	1999,14	1,68E+07	2,27E+05	4,48E+06	High	667,05194	90,4461	2,58
RSDLEMQIE	Q90303	1120,53	1,57E+05	2,28E+05	1,52E+05	High	560,76965	48,3369	1,94
WADLSPGSGPVKK	Q90487	1341,72	1,17E+05	2,29E+05	1,05E+05	High	671,36273	33,2384	1,96
APRAVFPSIVGRPRHQGVMVGM	P83750	2362,27	1,12E+06	2,30E+05	4,59E+05	High	591,32562	59,4689	3,59
LGEQIDNLQRV	Q90339	1284,69	1,46E+05	2,30E+05	1,60E+05	High	642,85077	58,1449	2,65
DIRLEAL	Q5TZ18	829,48	1,54E+05	2,30E+05	1,48E+05	High	415,2439	62,301	1,95
RDILEAL	Q1LUA6	829,48	1,54E+05	2,30E+05	1,48E+05	High	415,24377	62,4874	2,16
GFAGDDAPRAVFPSIVGRPRHQG	P83750	2407,24	3,65E+05	2,30E+05	1,31E+05	High	482,25461	54,7591	3,74
GQKDSYVGDEAQSKRGI	P83750	1837,90	1,62E+05	2,31E+05	1,26E+05	High	460,23209	25,0791	2,62
KIEDEQSLGAQLQKKIKEL	Q90339	2198,24	4,84E+05	2,31E+05	1,01E+06	High	550,31659	69,8616	4,12
MLAENIPVPSSPVPIATIDLVQN	B0UYT5	2418,29	2,92E+05	2,32E+05	5,01E+05	High	605,32349	41,3863	2,82
SGGTTMYPGIADRMQ	P83750	1584,71	2,16E+05	2,33E+05	2,65E+05	High	792,86273	57,2547	3,1
SGGTTMYPGIADRMQKEITA	P53479	2127,02	7,58E+05	2,33E+05	1,99E+05	High	709,68024	69,9854	2,66
AKESEAGAGDAIEPAPAAEGEAAKPEGEATKE	Q6NWH2	3081,45	3,26E+05	2,34E+05	4,58E+05	High	514,41437	20,1207	3,55
DDLQAEEDKVNTL	Q90339	1489,70	1,49E+05	2,34E+05	3,00E+05	High	745,35565	64,6139	2,18
FAGDDAPRAVFPSIVGRPRHQGVM	P83750	2580,33	6,01E+05	2,34E+05	4,83E+05	High	516,87305	62,3862	4,05
REIVRDIKEKL	P83750	1398,84	2,63E+05	2,36E+05	2,33E+05	High	350,46732	31,345	2,92
EGSLEQEKKLRMD	Q90339	1562,78	9,94E+04	2,37E+05	1,47E+05	High	521,60126	27,417	3,08
VGMGQKDSYVGDEAQSKRGILTL	P83750	2452,25	3,41E+05	2,37E+05	2,57E+05	High	613,81903	60,2374	4,48
LDVSENRL	Q4H4B6	945,50	3,51E+05	2,37E+05	8,03E+04	High	473,25519	30,8724	2,19
VFPSIVGRPRHQGVMVGM	P83750	1967,05	7,32E+05	2,37E+05	5,29E+05	High	656,35504	61,0766	2,67
MVNLPPLDFKPALMIETFSLNA	A0A0R4IES7	2493,28	6,53E+05	2,38E+05	4,73E+05	High	499,46536	63,5184	2,87
SVNGIDLRGATHEQAAAALKNAGQT	Q5PYH5	2493,28	6,53E+05	2,38E+05	4,73E+05	High	499,46536	63,5184	2,83
QSVLLQQQQQQLQQQQQR	Q4KME6	2493,29	6,53E+05	2,38E+05	4,73E+05	High	499,46552	63,6604	2,62
MLPIPTAAPNISMAVGPFEILVDP	Q32PW3	2493,31	6,53E+05	2,38E+05	4,73E+05	High	499,46536	63,5184	2,85
QVLPSLALGI	Q98864	1010,62	1,47E+05	2,39E+05	1,56E+05	High	505,81802	62,0712	1,97
TVDGPSGKLWRDGRGASQNIIP	Q5XJ10	2324,21	1,42E+06	2,39E+05	2,72E+05	High	581,81055	60,9506	2,99
ELPDGQVITIGNER	P83750	1540,80	2,09E+05	2,39E+05	2,50E+05	High	770,90308	63,9857	2,05
SAGIHETAYN	P53479	1062,49	1,40E+05	2,39E+05	1,27E+05	High	531,7478	22,6478	1,9
DTGAPIRIPVGPET	Q9PTY0	1422,76	1,69E+05	2,41E+05	7,39E+04	High	711,88489	72,0228	2,16
EYKEAFTIIDQNRDGIISK	O93409	2240,16	3,29E+05	2,41E+05	3,95E+05	High	747,3924	66,7722	3,76
NSEVAQWR	Q90339	989,48	1,41E+05	2,47E+05	1,59E+05	High	495,24573	29,0651	2,56

IADLNRMIQ	Q6NWF6	1073,58	1,61E+06	2,47E+05	1,64E+06	High	537,29413	55,0327	2,55
FQDVLDKL	Q6NZS4	977,53	2,15E+05	2,49E+05	1,89E+05	High	489,27057	66,8009	2,06
LEEISERLEEAGGAT	Q90339	1603,78	2,42E+05	2,49E+05	3,93E+05	High	802,39642	61,4478	4,15
SLYASGRTTGIVL	P53479	1337,74	2,45E+05	2,50E+05	2,47E+05	High	669,37665	66,2021	2,71
IIDQNRDGIISKDDLRDVL	O93409	2198,18	9,86E+05	2,51E+05	2,33E+05	High	550,30188	72,2466	3,51
GEHGDSSVPVWSGV	Q9PVK5	1412,64	1,47E+05	2,52E+05	1,75E+05	High	706,82666	63,5928	2,11
KDEEMEQIKRNSQRVIDSM	Q90339	2336,13	4,21E+05	2,53E+05	4,02E+05	High	584,78992	64,1063	3,48
EQIDNLQRVKQKLEKE	Q90339	1998,10	3,12E+05	2,54E+05	2,70E+05	High	500,28033	42,5646	3,26
AGDDAPRAVFPSIVG	P83750	1471,75	2,59E+05	2,57E+05	2,88E+05	High	736,38226	77,4416	2,9
PTPAGTPIMNIIRPTQMATVLP	Q6P0D0	2351,25	1,81E+06	2,57E+05	9,24E+05	High	588,56616	70,0214	2,69
TSLINTKKKLE	Q90339	1274,77	1,94E+05	2,57E+05	2,74E+05	High	425,59601	21,2529	3,16
VEKQRADLSRELEEISERL	Q90339	2300,22	2,16E+06	2,57E+05	1,55E+06	High	575,81146	81,0352	3,96
AGFAGDDAPRAVFPSIVGRPRHQGVMVGM	P83750	2995,51	1,97E+06	2,60E+05	8,83E+05	High	749,63562	75,6492	3,14
KSYELPDGQVITIGNERFR	P83750	2222,16	3,74E+06	2,61E+05	1,87E+06	High	556,29596	74,4815	5,57
ADVLDLAETMVASDGLVYEPVVFD	Q7T2D4	2568,24	4,36E+05	2,61E+05	5,22E+05	High	642,81842	41,4338	2,87
FPSIVGRPRHQGVMVG	P83750	1736,94	4,91E+05	2,62E+05	1,43E+05	High	579,65247	46,9397	3,12
KLEKTIDDLEDE	P13104	1447,72	6,14E+05	2,62E+05	5,80E+05	High	724,36426	43,9919	2,42
TSVGNILGSLIAGVYVSSAWGLSFIV	Q7SY29	2610,42	5,08E+05	2,63E+05	7,42E+05	High	522,88812	32,2086	2,61
VDDLEGSLEQEKKLRMD	Q90339	2004,99	2,84E+05	2,64E+05	3,12E+05	High	502,00522	57,0326	4,59
AANLDKKQRNFDKVLAEW	Q90339	2146,14	5,15E+05	2,64E+05	5,94E+05	High	716,05371	56,8422	2,35
VDDLEGSLEQEKKLRM	Q90339	1889,96	2,21E+05	2,65E+05	2,70E+05	High	473,24753	56,8201	4,08
KSSSLTRTGNSVGADVLTEHPLLSEP	A0A0R4IES7	2695,39	5,54E+05	2,66E+05	7,50E+05	High	539,88788	36,3024	2,6
NQQGAKFQLVSGAANAGGSPQV	B0R0I6	2129,07	4,31E+05	2,67E+05	4,28E+05	High	533,02734	63,4276	2,78
SLEKSYELPDGQVITIG	P83750	1848,96	1,55E+06	2,67E+05	6,98E+05	High	924,98547	98,1774	1,9
KVAGEVWVADSDGEI	Q0P4A4	1574,77	2,44E+05	2,69E+05	3,52E+05	High	525,59973	35,4103	2,37
KMEGGAENDLRILE	Q66I22	1574,78	2,44E+05	2,69E+05	3,52E+05	High	525,59973	35,4103	2,36
AALEQTERGRKVAEQE	Q90339	1814,94	1,46E+05	2,70E+05	2,25E+05	High	605,65088	20,6378	2,58
KQLRNVQGQLKDA	Q90339	1497,85	1,73E+05	2,71E+05	2,62E+05	High	499,95609	18,3582	2,4
LEKTIDDL	P13104	946,51	1,73E+05	2,72E+05	1,73E+05	High	473,75928	43,3679	2,08
LEENKLESLPNEIAYL	Q1L8Y7	1874,97	2,41E+05	2,72E+05	4,38E+05	High	469,50146	46,1617	2,77
KEFLEELL	O93409	1020,56	4,07E+05	2,72E+05	3,86E+05	High	510,78546	93,3245	2,22
DQNRDGIISKDDLRDVL	O93409	1972,01	3,98E+05	2,72E+05	1,34E+05	High	658,00928	69,4308	3,47
RHQGVMVGM	P83750	1014,50	1,87E+05	2,73E+05	1,89E+05	High	338,83813	25,3518	2,79

KALDVDASGFIEEEELK	Q804W2	1892,95	2,73E+05	2,75E+05	2,36E+05	High	631,6557	57,3994	2,37
DNEFGYSNR	Q5XJ10	1101,46	2,03E+05	2,75E+05	1,07E+05	High	551,23511	33,1227	2,42
ETAYNSIMK	P53479	1056,50	1,64E+05	2,75E+05	1,31E+05	High	528,7572	37,4949	1,91
ITGESGAGKTVNTKRVIQYF	Q90339	2169,17	3,30E+05	2,76E+05	4,03E+05	High	723,72955	51,2411	4
EEISERLEEA	Q90339	1204,57	2,33E+05	2,76E+05	2,67E+05	High	602,7901	45,4977	3,09
KIDDLVGGL	Q90487	929,53	2,20E+05	2,77E+05	1,56E+05	High	465,26999	68,929	2,17
SLINTKKKLE	Q90339	1173,72	1,23E+05	2,79E+05	2,67E+05	High	391,91232	19,2072	2,5
TNEKLQQFFN	Q90339	1268,63	1,95E+05	2,81E+05	9,76E+04	High	634,81854	58,5	2,05
IVGRPRHQGVMV	P83750	1348,76	1,62E+05	2,82E+05	7,11E+05	High	450,2608	20,8224	2,43
IKSKIQLEAK	Q90339	1157,73	3,35E+04	2,82E+05	1,97E+05	High	386,58148	16,5263	2,43
AGDDAPRAVFPSIVGRPRHQGVM	P83750	2433,26	3,40E+05	2,83E+05	3,77E+05	High	609,06952	54,3235	4,86
EYKEAFTIIDQNRDGIIS	O93409	2112,06	6,77E+05	2,84E+05	6,29E+05	High	704,6936	78,805	3,48
EVIVNEGVNSISYTLDEGLIEF	Q5RHH4	2440,21	4,57E+05	2,85E+05	4,85E+05	High	488,84399	39,2349	2,85
MREIPGVQGGHRAEGYLGAAAAAAA	Q90322	2440,22	4,57E+05	2,85E+05	4,85E+05	High	488,84473	39,3403	3,37
VLDPDEGIRF	Q7ZVY5	1160,59	2,87E+05	2,86E+05	1,41E+05	High	580,80255	71,1224	2,23
GALDDISKIPE	Q7SYK7	1157,60	2,04E+05	2,86E+05	1,60E+05	High	579,30756	68,3934	2,31
KQRNFDKVLAE	Q90339	1347,74	1,72E+05	2,86E+05	2,31E+05	High	449,91882	24,2751	2,42
LYASGRTTGIVL	P53479	1250,71	2,76E+05	2,88E+05	3,10E+05	High	625,8606	59,7483	2,55
TEQLGETGKSIH	Q90339	1299,65	1,50E+05	2,90E+05	2,42E+05	High	433,89102	20,4793	2,58
IQTAVRL	Q6PC60	800,50	2,11E+05	2,90E+05	2,36E+05	High	400,75427	39,3391	2,07
SERVGLLH	Q90339	910,51	9,74E+04	2,90E+05	8,90E+05	High	455,76089	24,0203	2,5
LIDGYPREVKQGEE	P12115	1632,82	2,26E+05	2,91E+05	2,54E+05	High	544,94751	38,5394	2,94
TGKSIHELE	Q90339	1013,53	1,66E+05	2,92E+05	2,94E+05	High	507,26865	21,2947	2,19
TIKKRLDLY	P12115	1149,70	2,01E+05	2,92E+05	5,10E+05	High	575,35492	25,69	2
GRKVAEQELVDAS	Q90339	1401,73	2,08E+05	2,93E+05	1,42E+05	High	467,91754	28,2189	2,44
GLNTRIGFAE	Q7SX99	1077,57	1,93E+05	2,94E+05	1,65E+05	High	539,29022	53,3678	2,43
DAGDGVTHNVPVYE	P53479	1472,67	2,90E+05	2,95E+05	2,64E+05	High	736,83771	52,3167	2,52
QTIAHQQQQLTNLQMAAVTMGFGDPLS	Q6IQE0	2928,43	2,09E+05	2,95E+05	2,42E+05	High	732,8576	28,7987	2,76
KKAAAEVNKDYGLDPKI	Q7SX99	1860,02	4,19E+05	2,95E+05	7,83E+05	High	620,6814	51,9147	2,3
SLNGAARLGTQKGKRGMV	A0A0R4IES7	1860,02	4,19E+05	2,95E+05	7,83E+05	High	620,68134	51,8884	2,59
FIMVILAPNPEAFGLLV	Q9I9R3	1860,03	4,19E+05	2,95E+05	7,83E+05	High	620,68134	51,8884	2,49
ADRKYEEVARKL	P13104	1477,81	1,64E+05	2,96E+05	3,27E+06	High	493,27753	25,1263	4,32
KKGIGPVYSAKAARSGL	Q568F6	1703,00	7,50E+05	2,97E+05	8,13E+05	High	568,33765	56,2734	2,41

TWYDNEFGYSNR	Q5XJ10	1551,65	3,85E+05	2,97E+05	2,76E+05	High	776,32904	69,5892	2,48
LNGPAPWGFRLQ	Q6P7E4	1355,72	4,01E+05	2,99E+05	3,82E+05	High	678,36542	85,8555	2,68
IVAINDPFIDL	Q5XJ10	1229,68	9,96E+05	2,99E+05	5,18E+05	High	615,34369	115,7903	2,33
AGFAGDDAPRAVFP	P83750	1390,67	2,25E+05	3,02E+05	1,45E+05	High	695,84271	71,3229	2,62
LNVKNEELE	O93409	1087,56	2,21E+05	3,02E+05	2,82E+05	High	544,28607	34,4985	1,93
GSLEQEKKLRMDLER	Q90339	1831,97	2,82E+05	3,03E+05	3,19E+05	High	458,74927	36,4125	3,38
DSYVGDEAQSKRGILTL	P83750	1851,94	2,85E+05	3,04E+05	7,44E+05	High	617,98743	67,1784	3,4
RVPTPNVSVVDLTVR	Q5XJ10	1651,95	4,02E+05	3,08E+05	4,80E+05	High	551,32257	66,7927	5,45
SELKKDIDDLE	Q90339	1304,66	2,87E+05	3,09E+05	2,75E+05	High	652,8338	42,4048	1,97
SLTDEINFLRQ	Q6NWF6	1335,69	4,63E+05	3,11E+05	2,51E+05	High	668,3501	90,7612	2,09
NELRVAPE	P83750	927,49	2,04E+05	3,12E+05	2,62E+05	High	464,24997	28,8129	1,97
KSYELPDGQVITIGNER	P83750	1918,99	8,40E+05	3,12E+05	5,58E+05	High	640,33533	77,0811	4,06
PESSSTTLMPHVGRKDKGGGGGGGGAPANSA	Q502M5	2853,35	7,01E+05	3,13E+05	7,27E+05	High	571,48224	46,2619	3,39
NMLERPSTPGKEPSSVEQAILSMPGTP	Q32PW3	2853,41	7,01E+05	3,13E+05	7,27E+05	High	571,48303	46,2334	3,39
KAKTKLEQQVDDL	Q90339	1515,84	3,02E+05	3,16E+05	2,23E+05	High	505,95181	32,4625	2,41
DAIKKKMQ	P13104	961,55	1,35E+05	3,16E+05	2,48E+05	High	481,28009	39,3462	2,31
MDAIKKKMQ	P13104	961,55	1,35E+05	3,16E+05	2,48E+05	High	481,28009	39,3462	2,31
ESERGMKVIENR	P13104	1447,73	1,68E+05	3,16E+05	1,63E+05	High	483,24991	18,934	2,59
DEAGPSIVHRK	P53479	1208,64	4,07E+04	3,18E+05	1,15E+05	High	403,55258	15,5084	2,4
DLSRELEEISE	Q90339	1319,63	4,48E+05	3,18E+05	2,61E+05	High	660,32178	84,1876	2,61
LDLAGRDLTDYLM	P83750	1495,75	1,94E+06	3,20E+05	1,07E+06	High	748,37817	107,2288	3,02
PPERKYSVWIGGSILA	P83750	1772,97	8,62E+05	3,21E+05	2,60E+05	High	591,66272	66,7137	2,69
IDNIFRFT	Q9PTY0	1025,54	2,63E+05	3,22E+05	2,31E+05	High	513,2757	80,7227	2,18
VGLSQSSKEALSDVNATGVAM	Q5BL29	2080,02	4,48E+05	3,23E+05	4,25E+05	High	694,01642	36,9259	2,33
SVTASSVASSTVSSSVSTSTTS	Q9I9L0	2019,96	2,41E+05	3,24E+05	5,03E+05	High	505,7403	38,6682	2,76
QIKIYELQE	Q5RFW0	1163,63	1,92E+05	3,27E+05	2,78E+05	High	582,32019	30,4283	2,21
GIGTVPVGRVETG	Q92005	1241,68	1,54E+05	3,28E+05	2,20E+05	High	621,34784	46,9978	1,91
SYGRALQASAL	Q8JH70	1136,61	2,88E+05	3,28E+05	3,76E+05	High	568,80804	45,5593	2,37
DSGDGVTHTVPIYEGY	P83750	1709,77	2,69E+05	3,29E+05	2,96E+05	High	855,38593	72,38	2,49
GYPREVKQGEE	P12115	1291,63	1,88E+05	3,31E+05	2,85E+05	High	646,31848	16,7514	2,06
ERLEDEEE	Q90339	1048,44	2,59E+05	3,31E+05	2,12E+05	High	524,72681	18,6996	2,41
NLSKSSTFSQVKETALFTL	Q1LX29	2101,12	6,18E+05	3,34E+05	7,00E+05	High	526,03436	47,1229	2,71
EREIVRDIKEKL	P83750	1527,89	3,61E+05	3,34E+05	3,01E+05	High	382,72827	39,2213	2,97

SLTDEINFLR	Q6NWF6	1207,63	4,80E+05	3,36E+05	3,53E+05	High	604,3208	89,2941	2,22
STELLIR	Q6PI20	831,49	1,55E+05	3,36E+05	2,02E+05	High	416,25192	39,5476	1,92
LSSGDLLR	P12115	860,48	1,73E+05	3,40E+05	1,46E+05	High	430,74686	43,1664	2,22
ASGRTTGIVM	P83750	992,52	1,76E+05	3,40E+05	3,08E+05	High	496,76462	30,2775	1,98
QEKKLRMDLER	Q90339	1445,79	2,94E+05	3,41E+05	3,88E+05	High	362,20367	19,7025	2,83
EAPLNPKANREKMTQIMFETF	P83750	2495,24	2,80E+06	3,41E+05	1,07E+06	High	624,56732	80,6091	2,61
EPGQNLPSFVEKWKFGAVVTDF	P34205	2495,26	2,80E+06	3,41E+05	1,07E+06	High	624,56726	80,5435	2,89
EVERKDISQSFKMEIS	G9G127	1925,96	6,16E+05	3,42E+05	4,50E+05	High	642,65424	72,9739	2,57
KFLCPQTPVESADKTQELLA	A2BID7	2275,16	7,51E+05	3,42E+05	6,37E+05	High	569,5517	62,3393	2,81
EKSYELPDGQVI	P83750	1377,69	3,01E+05	3,43E+05	4,55E+05	High	689,35059	70,3012	2,02
AGDDAPRAVFPSIVGRPRHQGVMVG	P83750	2589,35	1,02E+06	3,46E+05	2,99E+05	High	648,09344	57,7645	3,29
FLQHRILN	P59679	1040,60	2,70E+05	3,46E+05	1,96E+05	High	520,80414	59,72	2,03
KRHIMPMDNLQPI	A0A1D5NSK(	1592,84	5,39E+05	3,47E+05	5,92E+05	High	531,62225	59,9348	2,43
EVKIMMMELISRL	Q6NV26	1592,86	5,39E+05	3,47E+05	5,92E+05	High	531,62225	59,9348	2,4
YPGIADRMQ	P83750	1050,50	2,16E+05	3,49E+05	2,46E+05	High	525,7572	42,8904	2,44
QKTVDGPSGKLWRDGRGASQ	Q5XJ10	2143,10	2,74E+05	3,49E+05	3,30E+05	High	536,53186	26,8276	3,64
AVFPSIVGRPRHQGVMVGM	P83750	2038,08	9,28E+05	3,50E+05	5,78E+05	High	680,034	65,5128	2,37
LDRAQERLATAL	P13104	1356,76	1,86E+05	3,52E+05	4,54E+05	High	452,92496	44,241	2,5
AANLDKKQRNFDKVLAEWK	Q90339	2274,24	5,16E+05	3,53E+05	5,22E+05	High	455,65411	46,9043	3,63
MLRRFPRSLLIL	Q9I9H8	1514,94	3,97E+05	3,54E+05	3,82E+05	High	505,64514	43,0889	2,56
TERGRKVAEQELVDAS	Q90339	1787,92	2,58E+05	3,55E+05	3,11E+05	High	596,64728	27,1014	2,41
VSEGTKAVTKY	Q6PC60	1182,64	1,76E+05	3,58E+05	2,48E+05	High	591,82251	18,0327	2,59
EQLGETGKSIHE	Q90339	1327,65	1,87E+05	3,58E+05	1,16E+06	High	664,32983	23,6177	2,57
EDKYEEEIK	P13104	1182,55	1,94E+05	3,58E+05	2,15E+05	High	591,78064	22,9703	2,39
ELKKEQDTSAHLE	Q90339	1527,76	2,30E+05	3,58E+05	3,05E+05	High	509,92807	16,0915	2,44
EKSYELPDGQVIT	P83750	1478,74	2,52E+05	3,61E+05	1,28E+05	High	739,87286	63,5478	2,02
LQHRLDEAE	Q90339	1110,55	6,48E+04	3,62E+05	1,61E+05	High	555,78174	16,5921	2,14
KLEVMLEDNLTLPSNGKL	Q5RG82	2014,09	5,76E+05	3,63E+05	4,43E+05	High	672,03711	76,7616	2,6
TMFGEKLKGADPEDVIVS	O93409	1935,97	7,32E+05	3,64E+05	5,84E+05	High	645,99731	72,5646	2,77
ALEEAEGTLEHEESKIL	Q90339	1897,94	1,16E+06	3,66E+05	8,52E+05	High	633,3194	77,0599	2,68
AELEGAQKEARSL	Q90339	1401,73	2,21E+05	3,67E+05	3,68E+05	High	467,91675	33,9408	3,38
APLNPKANREKMTQI	P83750	1710,93	3,13E+05	3,68E+05	1,56E+05	High	570,98383	28,9345	2,56
TIIDQNRDGIISKDDLRD	O93409	2087,07	3,37E+05	3,69E+05	3,27E+05	High	522,52545	52,9363	3,73

TKAKTKLEQQVDDLEGS	Q90339	1889,98	3,27E+05	3,70E+05	4,63E+05	High	630,66766	41,5059	2,31
GFAGDDAPRAVFPSI	P83750	1519,75	4,36E+05	3,70E+05	3,12E+05	High	760,38293	86,5324	2,23
MSRPSSAGGAAGGLGAGKAGGSKHGGSGGTTA	Q7SYL3	2568,25	7,67E+05	3,71E+05	8,57E+05	High	514,45648	41,7397	3,92
AAASGARGAEPGEKGGISEVGPEVDVTG	Q5TZ18	2568,25	7,67E+05	3,71E+05	8,57E+05	High	514,45599	41,4472	2,89
TGTGLEGGVGALQGGVCFSLRKMEQ	F1QXM5	2568,25	7,67E+05	3,71E+05	8,57E+05	High	514,45685	41,633	3,79
RDTPGWVTVFGMAGSGKSVMAAEVV	Q9I9H8	2568,26	7,67E+05	3,71E+05	8,57E+05	High	514,45599	41,4472	3
MAAAAAAAAGTVTPTTNSVPPAAGATTEP	Q0P496	2568,26	7,67E+05	3,71E+05	8,57E+05	High	514,45685	41,633	3,92
FNRGAAFSAQFMAVPAFFSKSSSI	Q8AYM7	2568,27	7,67E+05	3,71E+05	8,57E+05	High	514,45599	41,4472	2,96
HHLSSPSGLDMGPLLGPEGVTPEQL	Q67FY3	2568,28	7,67E+05	3,71E+05	8,57E+05	High	514,45599	41,4472	2,9
TLTPSELTVEKDDGQGSPKPESPR	Q5RJ80	2568,28	7,67E+05	3,71E+05	8,57E+05	High	514,45685	41,633	3,97
DLRDVLATM	Q66I73	1049,53	1,43E+05	3,72E+05	2,36E+05	High	525,27026	66,57	2,39
LLSKIEDEQSL	Q90339	1274,68	2,08E+05	3,73E+05	3,24E+05	High	637,8476	54,1575	2,38
TLDDLQAEEDKVN	Q90339	1489,70	1,75E+05	3,73E+05	4,02E+05	High	745,35571	51,2351	2,98
IRPIYSNPPMNGA	Q7SYK7	1429,73	2,29E+05	3,75E+05	2,49E+05	High	715,36871	50,8955	2,34
VPGPSVPGTPLSAPGGPPEK	Q7ZU80	1840,98	1,40E+06	3,76E+05	5,81E+05	High	614,33514	71,8649	2,56
NRRIQLVEEELDRAQE	P13104	1998,04	1,11E+06	3,77E+05	1,61E+06	High	500,26563	53,3281	4,43
GGAKRVIISAPSA	Q5XJ10	1226,72	2,53E+05	3,78E+05	1,56E+05	High	409,57977	29,0851	2,89
RVIISAPSADAP	Q5XJ10	1196,66	5,00E+05	3,80E+05	5,68E+04	High	598,83698	47,6506	2,66
STSLSGIGSGLGMPTVSSDVFSARKMST	A1A5H6	2792,34	6,47E+05	3,82E+05	5,06E+05	High	559,27502	40,1222	3,24
SINSDVNNLMTVLSMSNALPEGLFPE	Q5RGU1	2792,35	6,47E+05	3,82E+05	5,06E+05	High	559,2746	39,7936	3,13
AIGGEGFTYPPHVGMSIGTSIDPVYVQ	Q5TZ24	2792,36	6,47E+05	3,82E+05	5,06E+05	High	559,27502	40,1222	3,13
KTVDGPSGKLW	Q5XJ10	1187,64	2,48E+05	3,84E+05	2,50E+05	High	594,32587	38,661	2,84
GDIDRDAWAVR	Q7SYK7	1273,63	1,47E+05	3,84E+05	1,60E+05	High	425,21545	42,6672	2,4
GRKVAEQELVDA	Q90339	1314,70	2,37E+05	3,85E+05	1,44E+05	High	438,90662	28,2478	3,33
YDEIKKVV	Q5XJ10	993,56	1,58E+05	3,87E+05	2,16E+05	High	497,28619	30,1689	2,23
EAPLNPKANRE	P83750	1238,65	5,94E+04	3,87E+05	1,63E+05	High	413,55579	15,3771	2,46
KAVGKVIPELNGKLT	Q5MJ86	1566,96	2,86E+05	3,89E+05	2,66E+05	High	522,99255	41,6173	2,38
GEQIDNLQRV	Q90339	1171,61	3,18E+05	3,90E+05	2,78E+05	High	586,30872	42,0413	2,56
RKLAEKDEEMEQIKRN	Q90339	2017,05	4,52E+05	3,91E+05	4,45E+05	High	404,21628	17,4862	3,02
ALEEAEGTLEHEE	Q90339	1456,64	3,53E+05	3,92E+05	2,37E+05	High	728,82672	47,7006	3,34
LEEISERLEEAGGA	Q90339	1502,73	3,58E+05	3,94E+05	6,67E+05	High	751,87189	61,2402	4,15
SEVAQWR	Q90339	875,44	1,94E+05	3,95E+05	2,40E+05	High	438,22318	27,5502	2,08
GESGAGKTVNTKRVIQ	Q90339	1644,90	2,66E+05	3,97E+05	2,46E+05	High	548,97394	16,1936	2,76

NEDSYKDSTLIM	Q6PC29	1415,64	1,89E+05	3,97E+05	3,76E+05	High	708,32233	57,2815	2,85
LDLAGRDLTDYL	P83750	1364,71	1,77E+06	3,97E+05	4,79E+05	High	682,85742	100,7105	3,42
EIQTAVRL	Q6PC60	929,54	2,77E+05	4,01E+05	2,92E+05	High	465,27451	46,9816	1,94
GIKVVDLLAPY	Q9PTY0	1187,70	6,76E+05	4,02E+05	5,52E+05	High	594,35669	93,6891	2,63
GFAGDDAPRAVFPSIVGRPRHQGVMV	P83750	2736,42	1,65E+06	4,02E+05	1,56E+06	High	548,09027	68,951	3,23
RRDLEESTLQH	Q90339	1383,70	2,27E+05	4,03E+05	4,59E+05	High	461,90607	18,9776	2,41
FHETLYQKA	P53448	1136,57	2,06E+05	4,04E+05	4,03E+05	High	568,79126	27,3758	3,13
VPTPNVSVVD	Q5XJ10	1026,55	4,05E+05	4,04E+05	6,82E+04	High	513,77826	58,7286	1,96
LDSLNSDQEKHIMKELLCLV	P0CI65	2385,22	7,94E+05	4,06E+05	4,97E+05	High	597,06097	60,1349	2,88
LSVGSIPRSMLIILE	F1QDI9	1643,94	3,58E+05	4,06E+05	5,38E+05	High	548,65021	35,9932	2,34
LQKKIKELQARIE	Q90339	1596,98	3,87E+05	4,07E+05	1,18E+06	High	400,0014	27,8547	3,09
ELKKDIDDLE	Q90339	1217,63	3,39E+05	4,08E+05	4,60E+05	High	609,31805	37,9742	2,05
EKTIDDLEDEL	P13104	1319,62	7,62E+05	4,08E+05	4,52E+05	High	660,31512	75,5985	2,37
HIASRTFNPDLLSRTPPGSP	Q5U3N0	2163,13	2,42E+06	4,09E+05	1,46E+06	High	541,54187	83,059	2,73
KQVEERDKEI	Q5RG45	1273,67	2,74E+05	4,09E+05	3,71E+05	High	425,23135	21,6608	2,39
KGGMDTFIE	Q803F5	997,47	2,22E+05	4,09E+05	2,89E+05	High	499,23819	42,8588	1,95
RCLLMHTGAVYLTSSASPELTACSI	A2BGS3	2754,33	4,81E+05	4,13E+05	4,74E+05	High	689,33344	39,4754	2,68
EERERLSSGGTPSEPSKRDEPKPAD	Q66I22	2754,33	4,81E+05	4,13E+05	4,74E+05	High	689,33484	39,7667	2,6
TAFGEFKFGPLFMRLCYELGLEE	Q566X6	2754,33	4,81E+05	4,13E+05	4,74E+05	High	689,33344	39,4754	2,78
SDHHVYLEGTLLKPNMVTA	Q8JH70	2125,07	8,92E+05	4,15E+05	7,89E+05	High	532,02655	67,8278	4,16
ELEEEIEAERAARAKVE	Q90339	1972,00	4,96E+05	4,15E+05	1,19E+06	High	658,00537	52,5704	3,34
LPESDLGAL	Q9W7R4	914,48	1,88E+05	4,16E+05	3,81E+05	High	457,74741	40,6352	2,16
ADMLRNISRSKDAVY	Q6DRL4	1754,89	6,33E+05	4,16E+05	9,88E+05	High	585,63574	76,6018	2,4
KVPSFAAGRVIGKGGKTV	Q9PW80	1772,05	3,71E+05	4,17E+05	5,26E+05	High	443,76685	22,6563	3,27
EKDEEMEQIKRN	Q90339	1548,73	2,50E+05	4,19E+05	2,65E+05	High	516,91718	17,2949	3,24
QGEALTQADIN	Q7ZVT5	1159,56	1,73E+05	4,20E+05	3,35E+05	High	580,2843	18,2763	1,94
RDLTDYLMK	P83750	1154,59	3,65E+05	4,20E+05	6,17E+05	High	577,79755	59,0918	2,44
AGFAGDDAPR	P83750	976,45	1,77E+05	4,21E+05	2,77E+05	High	488,72916	25,0537	2,26
LEQQVDDLEGSLEQEKKLRMD	Q90339	2503,23	1,60E+06	4,24E+05	1,09E+06	High	626,56488	70,9699	5,75
ADLSRELEEISE	Q90339	1390,67	6,46E+05	4,24E+05	5,62E+05	High	695,84064	78,1848	2,44
LAGRDLTDYLM	P83750	1267,64	5,71E+05	4,25E+05	5,97E+05	High	634,32251	85,2055	2,37
GIADRMQKE	P83750	1047,53	1,69E+05	4,26E+05	#DIV/0!	High	524,26868	19,6955	2,07
DTGAPIRIPVGPE	Q9PTY0	1321,71	4,95E+05	4,27E+05	3,01E+05	High	661,36072	71,9555	3,49

GEKLKGADPEDVIVSAF	O93409	1774,92	4,84E+05	4,27E+05	4,52E+05	High	592,31378	81,672	2,98
FYNELRVAPEEHPTLL	P53479	1927,99	1,15E+06	4,29E+05	7,23E+05	High	643,33673	76,5873	2,84
WADLSPGSGPVKKHGKTIMGA	Q90487	2137,12	4,69E+05	4,29E+05	5,89E+05	High	535,03723	37,0151	4,07
DRLATGIKAI	Q6NWF6	1057,64	3,40E+05	4,30E+05	6,44E+05	High	529,31744	66,0506	2,06
EKGKGKEQVTGAAAAAAMPVKK	Q7T370	2170,20	5,95E+05	4,32E+05	6,48E+05	High	434,84915	30,1201	2,84
KEKGKGKEQVTGAAAAAAMPVK	Q7T370	2170,20	5,95E+05	4,32E+05	6,48E+05	High	434,84927	29,6693	3,14
LEKEKKELQERLKNQEK	Q6PCR7	2170,22	5,95E+05	4,32E+05	6,48E+05	High	434,84915	30,1201	2,73
TIIDQNRDGIISKDDLRDVLAT	Q66I73	2471,31	1,83E+06	4,34E+05	1,27E+06	High	824,44427	81,1922	4,56
LTEAPLNPK	P83750	982,56	1,93E+05	4,34E+05	4,30E+05	High	491,78302	29,8673	2,09
KRENKNLQQEISDL	Q90339	1714,91	2,91E+05	4,35E+05	4,50E+05	High	572,30914	42,77	2,72
LEQTERGRKVAEQELVDASE	Q90339	2287,15	6,06E+05	4,39E+05	8,93E+05	High	572,54572	44,7301	3,32
EELKLFLQ	P05939	1019,58	5,77E+05	4,39E+05	4,31E+05	High	510,29364	77,7382	2,32
RKLLEGEEDRLAT	Q6NWF6	1529,83	3,53E+05	4,41E+05	3,32E+05	High	510,61572	33,3819	3,34
AADESERGMKVIENR	P13104	1704,83	2,77E+05	4,41E+05	1,91E+05	High	568,95099	28,968	3,44
ELDHALNDMTSI	P13104	1358,63	4,42E+05	4,43E+05	7,14E+05	High	679,81763	73,1013	2,47
SSLEKSYELPDGQVITIGNE	P83750	2179,08	2,85E+06	4,43E+05	1,08E+06	High	1090,0448	94,6458	4,31
GVQVDVDAPKGQ	Q7ZTS4	1212,62	#DIV/0!	4,45E+05	#DIV/0!	High	606,81696	34,5726	2,4
MGQKDSYVGDEAQSKRGILTL	P83750	2296,16	7,90E+05	4,45E+05	1,23E+06	High	574,79724	55,1514	4,87
LEQQVDDLEGSLEQEKKLRM	Q90339	2388,21	1,47E+06	4,45E+05	1,03E+06	High	597,80902	70,6543	6,72
ANREKMTQIMFET	P83750	1598,77	3,52E+05	4,46E+05	2,60E+05	High	533,5954	51,4663	2,85
ANREKMTQIMF	P83750	1368,68	3,56E+05	4,48E+05	8,28E+05	High	684,8432	47,4941	2,78
KLEQQVDDLE	Q90339	1216,61	2,90E+05	4,48E+05	1,95E+05	High	608,80774	39,9065	2,49
YPGIADRMQKE	P83750	1307,64	4,11E+05	4,48E+05	5,16E+05	High	654,32538	34,3405	2,35
GEHGDSSVPVWSGVNVA	Q9PVK5	1696,79	3,08E+05	4,48E+05	3,16E+05	High	848,90149	71,0658	2,5
GAELRIVL	Q6P0G6	870,54	1,82E+06	4,48E+05	2,21E+06	High	435,77539	69,6377	2,69
LEGEEDRLAT	Q6NWF6	1132,55	1,82E+05	4,51E+05	2,06E+05	High	566,77966	32,5905	1,9
TKLEQQVDDLEGS	Q90339	1461,71	3,05E+05	4,52E+05	4,15E+05	High	731,35956	51,3275	2,08
DIDIRKDLYANNVLSGGTT	P53479	2065,06	1,19E+06	4,52E+05	1,01E+06	High	689,02582	84,5147	4,41
RLDLAGRDLTDYLM	P83750	1651,85	2,29E+06	4,53E+05	1,69E+06	High	551,28833	93,2391	2,76
GERVGGF	Q7SYK7	721,36	1,06E+05	4,57E+05	1,74E+05	High	361,18622	25,5061	2,08
TLEDQLSEIK	Q90339	1175,62	2,76E+05	4,57E+05	4,27E+05	High	588,31226	61,7038	2,22
MASQDESIAKL	Q90339	1192,59	2,24E+05	4,58E+05	4,46E+05	High	596,79938	44,6101	2,62
RRDLEESTLQHEATAA	Q90339	1826,90	2,88E+05	4,58E+05	5,79E+05	High	609,63934	33,4736	3,12

SDLTEQLGETGKSIHELE	Q90339	1985,97	1,25E+06	4,58E+05	8,74E+05	High	662,66101	72,0071	3,58
SVLDPDEGIRFR	Q7ZVY5	1403,73	4,29E+05	4,60E+05	3,95E+05	High	468,58194	59,907	2,43
TIEDGIFEVK	Q90473	1150,60	3,39E+05	4,62E+05	4,49E+05	High	575,80505	68,0542	2,21
VGYDDIGGVR	Q7ZU99	1050,52	2,17E+05	4,63E+05	2,20E+05	High	525,76569	45,0581	2,01
LPEFAAEAKTSQDPPAVAMETDLPVSPPA	Q7ZU80	2979,47	5,50E+05	4,64E+05	1,19E+06	High	596,70227	38,0795	3,27
LSGIGSGLGMPTVSSDVFSARKMSTPGLNP	A1A5H6	2979,49	5,50E+05	4,64E+05	1,19E+06	High	596,70239	38,0322	2,82
ETGKSIHELE	Q90339	1142,57	2,76E+05	4,65E+05	2,58E+05	High	571,78943	19,9608	1,96
RIPSAVGYQPT	Q9PTY0	1188,64	2,78E+05	4,66E+05	3,57E+05	High	594,82349	40,6473	2,36
LVVYPQTKTY	P82315	1211,67	3,17E+05	4,68E+05	5,31E+05	High	606,33893	48,3102	2,06
AKTKLEQQVDDLEGSLE	Q90339	1902,97	1,70E+06	4,68E+05	1,26E+06	High	634,99542	78,3587	4,07
AEELKKEQDTSAHLERMKKNLEVTVK	Q90339	3070,62	6,18E+05	4,69E+05	1,16E+06	High	614,93085	40,6184	3,81
LDNFPTNLHPM	Q7ZVY5	1298,62	2,66E+05	4,73E+05	4,38E+05	High	649,81439	69,3201	1,91
RDLEESTLQHE	Q90339	1356,64	3,34E+05	4,75E+05	2,80E+05	High	678,82483	27,8865	2,39
VDDLEGSLEQEKKLR	Q90339	1758,92	4,42E+05	4,82E+05	4,06E+05	High	586,98083	43,6349	4,37
AEKDEEMEQIKRN	Q90339	1619,77	2,94E+05	4,84E+05	4,00E+05	High	540,59644	18,249	3,65
EAFTIIDQNRDGIIS	O93409	1691,86	6,33E+05	4,84E+05	3,39E+05	High	846,43555	81,1832	3,1
TERGRKVAEQELVDA	Q90339	1700,89	3,63E+05	4,88E+05	4,77E+05	High	567,63733	26,5942	2,64
EAEGTLEHEE	Q90339	1143,48	3,31E+05	4,88E+05	2,77E+05	High	572,24579	21,203	2,1
GRDLTDYLM	P83750	1083,51	3,35E+05	4,93E+05	5,59E+05	High	542,26154	75,2184	2,03
GLIKSKIQLE	Q90339	1128,70	5,38E+05	4,94E+05	8,54E+05	High	564,85443	40,6758	2,82
VIPELNGKLTGM	Q5MJ86	1271,70	3,70E+05	4,95E+05	6,10E+05	High	636,35712	71,8632	2,02
EYDESGPSIVH	P83750	1232,54	2,27E+05	4,95E+05	2,85E+05	High	616,77655	39,3659	2,65
FGGGRGGGFGGGRGGGFGGGRGGRG	Q7ZVE0	2111,01	4,80E+05	4,96E+05	5,78E+05	High	528,50757	30,175	3,14
FGGRGGGFGGRGGGGGGFRGGRGGG	Q7ZVE0	2111,01	4,80E+05	4,96E+05	5,78E+05	High	528,50757	30,175	3,16
AEKAADESERGMKVIEN	P13104	1876,91	2,89E+05	4,97E+05	3,10E+05	High	626,30823	28,7688	3,64
GIVLDAGDGVTH	P53479	1153,58	4,18E+05	4,99E+05	3,41E+05	High	577,29803	50,5422	2,97
WRDGRGASQNIIP	Q5XJ10	1469,76	5,60E+05	5,01E+05	1,75E+05	High	490,59308	44,0351	2,47
EYDESGPSIVHR	P83750	1388,64	2,14E+05	5,05E+05	3,43E+05	High	463,5546	32,1349	2,78
VDAAPQEDLSRVM	Q90303	1430,69	3,45E+05	5,07E+05	4,67E+05	High	715,85272	63,7001	2,81
KEAFTIIDQNRDGIISKDDLRDVLA	O93409	2845,51	6,29E+06	5,07E+05	3,35E+06	High	712,133	84,2126	5,91
FIGMESAGIHE	P53479	1190,55	3,46E+05	5,08E+05	3,13E+05	High	595,78082	60,6048	2,49
FKQSNVAKL	Q6AZB8	1034,60	2,34E+05	5,08E+05	9,14E+05	High	517,8045	30,3139	2,13
SYGRALQAS	Q8JH70	952,48	3,52E+05	5,09E+05	4,61E+05	High	476,74808	21,9434	1,93

LVDASERVG	Q90339	945,50	5,40E+05	5,10E+05	1,25E+05	High	473,2551	25,7886	2,3
KVGDKILGDLS	Q08BY1	1144,66	2,95E+05	5,11E+05	5,59E+05	High	572,83435	37,6629	1,94
ASGDIDRDAWAVR	Q7SYK7	1431,70	2,37E+05	5,13E+05	2,81E+05	High	477,90591	44,0191	3,27
KQKEVEDL	A2BGD5	988,53	7,16E+04	5,18E+05	1,40E+05	High	494,7709	17,9771	2,25
TAVDVLLSQG	A8WHR0	1002,55	2,68E+05	5,19E+05	3,41E+05	High	501,77872	56,9437	2,14
EELALRAEEAE	P85001	1259,61	3,31E+05	5,20E+05	3,47E+05	High	630,31049	36,8246	1,95
STGAAKAVGKVIPELNGKLTGM	Q5MJ86	2142,20	1,02E+06	5,20E+05	1,07E+06	High	714,7381	67,6418	5,17
RVAPEEHPTLLTEAPLNPK	P53479	2112,14	5,83E+05	5,21E+05	4,57E+05	High	704,72131	51,0093	5,66
REKMTQIMFETF	P83750	1560,75	2,01E+06	5,22E+05	1,00E+06	High	520,92395	74,9578	2,47
KIKIIAPPER	P83750	1164,75	3,85E+05	5,22E+05	2,82E+05	High	388,92148	26,3341	2,91
SEELDHALNDM	P13104	1273,54	2,97E+05	5,24E+05	5,44E+05	High	637,27283	58,7877	2,82
KQRADLSRELE	Q90339	1344,72	3,92E+05	5,24E+05	4,34E+05	High	448,91391	20,2561	2,92
NNNTIPPIQLAPTPASNSSLE	Q0VA82	2178,10	3,43E+05	5,26E+05	3,15E+05	High	726,70026	58,6208	2,39
NLRRQLDSLGNDKMKLE	Q6NWF6	2030,08	7,70E+05	5,27E+05	5,39E+05	High	508,27692	49,0631	4,94
IKSKIQLE	Q90339	958,59	1,93E+05	5,27E+05	6,84E+05	High	479,80151	22,2309	2,54
VTGDGVNDSPALK	P25489	1272,64	2,24E+05	5,27E+05	2,47E+05	High	636,82697	30,9583	3,24
ENSEISLKAGETVDVIEKSESGWWFV	Q1LYG0	2939,43	1,39E+06	5,31E+05	9,79E+05	High	588,68848	49,072	2,6
GIPGGKGEMGPPGPPGQEGLTGAPGTHGVKGE	A5PN28	2939,43	1,39E+06	5,31E+05	9,79E+05	High	588,68848	49,072	2,6
GGTHGAVSVQWNITRNSTDRSPVSADLN	Q6JAN0	2939,44	1,39E+06	5,31E+05	9,79E+05	High	588,68927	49,1244	2,91
SASGLHGRNRTLSHEYCVLGAGPAGLQM	B0UXS1	2939,44	1,39E+06	5,31E+05	9,79E+05	High	588,68927	49,1244	3,02
IAQGGVLPNIQ	Q7ZUY3	1109,63	3,79E+05	5,32E+05	4,15E+05	High	555,3208	68,1633	2,33
LIEKPMGIF	Q90339	1047,59	4,36E+05	5,32E+05	4,93E+05	High	524,30029	73,8286	2,01
RVTIMPKDIQLARRIRGERA	Q6PI20	2379,39	2,92E+06	5,34E+05	2,09E+06	High	397,4053	41,8597	4,1
KLDKENALDRAEQ	P13104	1529,79	2,90E+05	5,35E+05	5,88E+05	High	510,60367	20,891	3,25
WADLSPGSGPVKKHGKTIMG	Q90487	2066,09	5,90E+05	5,38E+05	6,09E+05	High	517,27869	36,6852	3,85
QSQMPTVSLASDLHTGPSVTPTPSSTQ	Q7SXE4	2754,33	7,54E+05	5,39E+05	7,13E+05	High	551,6687	39,535	4,02
ASQDESIAKL	Q90339	1061,55	2,51E+05	5,39E+05	4,06E+05	High	531,27887	37,9147	2,04
VTWYDNEFGYSNR	Q5XJ10	1650,72	7,63E+05	5,40E+05	7,15E+05	High	825,86469	74,4964	3,03
YETDAIQRTE	Q90339	1225,57	1,91E+05	5,47E+05	2,97E+05	High	613,29181	30,6306	2,66
TDSERLIGDAA	P47773	1147,56	2,88E+05	5,50E+05	2,54E+05	High	574,2851	38,0534	2,52
DDAPRAVFP	P83750	987,49	2,68E+05	5,53E+05	2,10E+05	High	494,24988	54,6312	1,97
AELGEQIDNL	Q90339	1101,54	3,08E+05	5,53E+05	4,17E+05	High	551,27716	73,332	2,02
LFPMKALGYFAVVTGKGS	Q5U3A7	1886,02	6,58E+05	5,56E+05	1,40E+06	High	472,25705	34,4063	2,65

KVNNVLL	E7FAW3	799,50	2,37E+05	5,56E+05	2,48E+05	High	400,25677	36,7297	1,93
GATTAQQGPSSASLHYPVTNQFTM	Q6DC04	2494,17	4,78E+05	5,57E+05	6,34E+05	High	499,6413	27,9866	2,82
SDVNNLMTVLSMSNALPEGLFPE	Q5RGU1	2494,18	4,78E+05	5,57E+05	6,34E+05	High	499,6413	27,9866	2,73
YSHLFTIAGTDADLEPYMLLAH	Q08BB2	2494,20	4,78E+05	5,57E+05	6,34E+05	High	499,64279	28,0145	2,85
GIVMDSGDGVTH	P83750	1187,54	2,52E+05	5,61E+05	4,50E+05	High	594,27264	39,1966	2,62
GGAGVGKTVLIM	Q9PTY0	1102,63	2,74E+05	5,62E+05	6,07E+05	High	551,81995	57,4231	2,82
ANLDKKQRNFDKV	Q90339	1575,86	3,67E+05	5,64E+05	1,58E+05	High	525,96051	15,9607	2,46
VDILPDHAFLQIFTHL	A1A5X2	1879,01	9,44E+05	5,65E+05	5,91E+05	High	627,01221	58,1323	2,31
MDLENEKQQSDEKIKKK	Q90339	2091,08	5,12E+05	5,65E+05	5,83E+05	High	523,52551	18,2994	3,01
LEEAEGTLEHE	Q90339	1256,56	3,02E+05	5,69E+05	4,26E+05	High	628,78699	33,7555	3,13
YETDAIQRTEELEE	Q90339	1725,78	3,72E+05	5,71E+05	4,96E+05	High	863,39636	59,1504	3,63
VFPSIVGRPR	P83750	1127,67	4,51E+05	5,72E+05	3,38E+05	High	376,56183	42,2879	2,72
LSGGTTMYPGIADRM	P83750	1569,74	6,76E+05	5,73E+05	5,66E+05	High	785,37512	67,5782	4
KSLEQDKESLLKETAENDQKL	G9G127	2446,27	1,06E+06	5,74E+05	6,52E+05	High	490,05835	41,8203	3,1
RMQKEITAL	P53479	1089,61	4,06E+05	5,77E+05	5,42E+05	High	545,30969	28,0039	2,49
GEQIDNLQRVKQKLEKEKSEYK	Q90339	2690,45	1,03E+06	5,79E+05	1,16E+06	High	673,36902	46,5732	4,39
LEEELKSL	Q6PBQ2	960,52	2,49E+05	5,79E+05	2,26E+05	High	480,76807	39,2747	2,12
LEEELKTV	P13104	960,52	2,49E+05	5,79E+05	2,26E+05	High	480,76764	39,3068	1,96
AEELKKEQDTSAHLE	Q90339	1727,84	3,67E+05	5,79E+05	4,48E+05	High	576,62115	22,1399	4,97
VIGEHGDSSVPVWSGV	Q9W7K5	1624,80	6,43E+05	5,82E+05	4,94E+05	High	812,90442	73,0808	3,23
DSYVGDEAQSK	P83750	1198,52	3,13E+05	5,82E+05	4,04E+05	High	599,76611	22,0525	2,36
GLIKSKIQLEAK	Q90339	1327,83	7,20E+05	5,85E+05	1,23E+06	High	443,28323	31,8381	3,98
KAKTKLEQQVDDLE	Q90339	1644,88	4,97E+05	5,85E+05	3,95E+05	High	548,96588	36,3881	3,97
IVAPGKGILAADE	Q8JH70	1253,71	4,66E+05	5,85E+05	5,53E+05	High	627,35968	54,6289	2,46
GQKDSYVGDE	P83750	1097,47	1,07E+05	5,86E+05	5,52E+05	High	549,24213	18,2719	2,78
RVKQKLEKEKSEYKME	Q90339	2053,11	9,45E+05	5,89E+05	1,16E+06	High	514,03461	12,9462	3,1
ELEEEIEAERA	Q90339	1317,62	3,55E+05	5,90E+05	1,02E+06	High	659,31378	50,1621	3,16
AFRVPVADVS	Q5MJ86	1060,58	3,05E+05	5,95E+05	3,76E+05	High	530,79449	58,0113	2,12
DLTEQLGETGKSIHELE	Q90339	1898,93	1,06E+06	5,96E+05	8,64E+05	High	633,65112	76,2151	2,66
SYLEIYNEKVLDLLSPGSQDLP	A8WFU8	2493,28	1,50E+06	5,97E+05	1,30E+06	High	624,07892	63,485	2,67
LTRDKMNINHEGQTTVPPMITQPTLQ	A0MSJ1	2979,50	7,16E+05	5,97E+05	1,31E+06	High	596,70343	37,7185	3,34
GEQIDNLQRVKQ	Q90339	1427,76	3,45E+05	6,01E+05	3,46E+05	High	476,59268	29,3076	2,94
GSLEQEKKLRM	Q90339	1318,71	2,75E+05	6,04E+05	4,58E+05	High	440,24457	23,5719	2,81

NVLSGGTTMYPGIADR	P53479	1651,81	6,84E+05	6,04E+05	5,41E+05	High	826,40991	67,2915	2,77
EYLENPKKYIPGTK	Q6IQM2	1679,90	4,61E+05	6,06E+05	5,63E+05	High	560,6402	46,0515	3,74
GGAKRVIISAPSADAPMFVM	Q5XJ10	2018,06	1,80E+06	6,08E+05	1,71E+06	High	673,35803	80,6023	4,22
GWLDKNKD	Q90339	975,49	4,18E+05	6,09E+05	7,76E+05	High	488,24982	23,1073	2
MVDREQLVQK	Q6PC29	1156,63	2,86E+05	6,10E+05	4,56E+05	High	578,82117	35,1807	2,46
VDGPSGKLWRDGRGASQ	Q5XJ10	1785,90	4,17E+05	6,11E+05	3,48E+05	High	595,97412	31,3901	3,21
GLSVEDVERIAYQSTVQLMFK	A0JMD4	2429,24	5,89E+05	6,12E+05	7,40E+05	High	608,06354	30,7001	2,71
RVIISAPSADAPMFVM	Q5XJ10	1704,88	2,60E+06	6,12E+05	2,64E+06	High	852,94623	96,4446	3,28
TKAKTKLEQQVDDLEGSLEQE	Q90339	2389,21	3,79E+06	6,14E+05	2,80E+06	High	598,05902	73,6331	3,83
LGEQIDNLQRVKQKLEKEKSE	Q90339	2512,37	1,12E+06	6,15E+05	1,08E+06	High	503,28204	50,2271	4,08
HDAVSAVLQ	Q90260	939,49	3,25E+05	6,16E+05	4,57E+05	High	470,24933	30,2449	2,24
LEQQVDDLEGSLEQEKKLR	Q90339	2257,17	1,27E+06	6,19E+05	8,25E+05	High	565,04889	60,3217	5,66
VSQLGAEPAGDTEQQLKKA	Q5TZ80	1970,02	2,14E+06	6,19E+05	9,07E+05	High	657,349	70,8787	2,59
KSAETLWGVQ	Q9PVK5	1118,58	3,82E+05	6,22E+05	5,98E+05	High	559,79694	52,8068	1,92
EDLLLKR	A5PF48	886,54	3,68E+05	6,23E+05	3,81E+05	High	443,77322	41,2062	1,96
KMGRMKSHGMTGAQTRFTF	A0A0R4IES7	2188,06	5,38E+05	6,26E+05	5,63E+05	High	547,7691	29,5634	2,79
VFPEGLGESWGHVSRTTSLE	Q98SJ5	2188,07	5,38E+05	6,26E+05	5,63E+05	High	547,7688	29,8958	2,91
SSTLRRASQRPESSGSVL	E7F6T8	1918,01	6,23E+05	6,28E+05	8,75E+05	High	480,25842	57,3697	3,08
KGVTVVSNSLLDNSVEEK	P85001	1918,01	6,23E+05	6,28E+05	8,75E+05	High	480,25812	57,3092	2,92
LAPLHEGSLGTIVGGAVGGALF	Q58EG3	2036,12	1,14E+06	6,28E+05	1,74E+06	High	408,03009	21,0595	3,63
SDVFSARKMSTPGLNP	A1A5H6	1706,85	3,01E+05	6,33E+05	4,59E+05	High	569,62134	21,3236	2,32
KTVLGMFPQLTVAP	Q08BB2	1501,84	8,24E+05	6,34E+05	6,60E+05	High	501,2905	48,4687	2,48
YPGIADR	P83750	791,40	3,99E+05	6,42E+05	4,30E+05	High	396,20712	28,6291	1,98
GATALQGVGTISVSPREVETR	A2VD33	2128,14	1,94E+06	6,43E+05	1,04E+06	High	710,05414	70,0288	2,42
RLDLAGRDLTDYL	P83750	1520,81	2,66E+06	6,46E+05	8,26E+05	High	507,60822	87,1959	2,46
TVRNDNSSRFGKF	Q90339	1527,77	4,48E+05	6,49E+05	1,66E+05	High	509,92871	25,5248	2,79
MGQKDSYVGDEAQSKRGIL	P83750	2082,03	7,12E+05	6,52E+05	9,32E+05	High	694,68188	39,1926	4,54
DNSRNLDMDAIVA	Q6NWF6	1449,66	3,62E+05	6,57E+05	4,06E+05	High	725,33777	66,0644	2,11
VIGEHGDSSVPVWSGVN	Q9W7K5	1738,84	5,83E+05	6,57E+05	4,46E+05	High	869,92413	66,4305	2,52
LLSKIEDEQSLGAQ	Q90339	1530,80	4,49E+05	6,59E+05	6,96E+05	High	765,90601	52,0036	2,65
RALYFFEQAITTPAMAVSH	Q6P2U9	2153,08	1,50E+06	6,60E+05	1,37E+06	High	718,36383	90,8227	2,71
STGVRNITALGNLISW	Q803A6	1701,93	5,17E+05	6,63E+05	5,22E+05	High	567,97729	31,02	2,3
DEAQSKRGILT	P83750	1217,65	3,00E+05	6,64E+05	5,02E+05	High	609,32953	21,6177	2,2

LEQQVDDLEGSLEQEKKLRMDLE	Q90339	2745,36	1,05E+07	6,65E+05	4,25E+06	High	687,09698	81,6288	6,01
FVEKWKFGAVVTDFNPLRIP	P34205	2363,29	1,07E+06	6,65E+05	7,00E+05	High	591,58362	39,137	3,13
LITIVGNIL	Q90WY5	955,62	5,27E+05	6,66E+05	1,33E+06	High	478,31406	77,4269	1,91
FDKVLAE	Q90339	821,44	4,78E+05	6,67E+05	3,97E+05	High	411,22488	35,0168	2,03
RLQGEVEDL	Q90339	1058,55	5,83E+05	6,70E+05	2,96E+05	High	529,77917	42,0033	2,18
KEDKYEEEIKVL	P13104	1522,80	4,93E+05	6,72E+05	7,00E+05	High	508,27286	45,9699	3,65
TDAIQRTEE	Q90339	1062,51	1,03E+05	6,74E+05	3,92E+05	High	531,75842	16,9523	2,11
TTMYPGIADRM	P83750	1255,58	3,55E+05	6,78E+05	4,39E+05	High	628,29602	59,8369	2,32
RGPALPSL	Q08CH3	810,48	3,33E+05	6,79E+05	5,63E+05	High	405,74637	30,3213	1,96
KDEEMEQIKRN	Q90339	1419,69	1,06E+05	6,80E+05	3,38E+05	High	473,90317	15,1964	4,15
AFTIIDQNRDGIISKDDLRDVLA	O93409	2588,37	8,76E+06	6,81E+05	4,97E+06	High	647,84882	91,9166	5,6
VLSGGTTMYPGIADRM	P83750	1668,81	8,94E+05	6,83E+05	6,95E+05	High	834,90918	72,6352	4,18
AKSNGLEELE	F1QWM2	1089,54	5,22E+05	6,86E+05	4,89E+05	High	545,27728	23,51	2,08
SAVEASGAGVLE	E7EZG2	1089,54	5,22E+05	6,86E+05	4,89E+05	High	545,27612	23,5342	1,93
GFAGDDAPRAVFPSIVGRPRHQGVMVGM	P83750	2940,47	2,60E+06	6,88E+05	1,26E+06	High	735,87457	74,742	3,23
ESQRKPSFNGELNGVLGSDLMGAAMV	Q1LUT1	2707,32	1,34E+06	6,91E+05	1,45E+06	High	452,0574	37,3357	3,3
QATPTGSSALTTSSSPLSVLTSSGTPSGQ	Q9IBD0	2707,33	1,34E+06	6,91E+05	1,45E+06	High	452,05682	37,0783	2,69
AYEPVWAIGTGKTASPQ	Q90XG0	1775,90	7,88E+05	6,91E+05	1,03E+06	High	888,45331	69,9923	2,03
KDRSSESNILPSFDPETVLQTAWS	Q7SX85-2	2707,32	1,27E+06	6,93E+05	1,49E+06	High	677,58112	36,8108	2,62
GGAKRVIISAPSADAPMFV	Q5XJ10	1887,02	1,06E+06	6,97E+05	5,80E+05	High	629,67767	71,1525	3,31
SLGVPVSGAQVTPQQQTQQITAVTQQAAG	B0R0I6	2893,50	1,36E+06	6,98E+05	1,33E+06	High	579,50916	54,7473	2,71
TAPAASPPLSLPQTPTTPTAPLTPLSQTH	Q2LE08	2893,53	1,36E+06	6,98E+05	1,33E+06	High	579,50916	54,7473	2,63
LGETGKSIHE	Q90339	1070,55	9,26E+04	7,01E+05	2,60E+05	High	357,52145	16,5438	2,43
TEAPLNPKANREKMT	P83750	1699,88	4,36E+05	7,05E+05	5,85E+05	High	567,2995	21,0699	3,21
EAFTIIDQNRDGIISKD	O93409	1934,98	7,93E+05	7,07E+05	5,67E+05	High	645,66711	67,1127	3,62
DDLRDVLATM	Q66I73	1148,56	8,13E+05	7,07E+05	9,16E+05	High	574,78607	97,0465	2,64
GPSIVHRK	P83750	893,53	3,43E+05	7,07E+05	4,81E+05	High	447,27054	24,6421	2,22
NNRFASF	Q6NWF6	855,41	3,43E+05	7,07E+05	1,03E+06	High	428,21017	38,8686	2,55
LRVAPEEHPTL	P53479	1261,69	4,18E+05	7,11E+05	3,97E+05	High	421,23608	40,0245	2,81
LSYDKVDFSSAEAINQEFFATRS	P31393	2625,25	1,24E+06	7,17E+05	1,61E+06	High	657,07288	39,1193	2,84
GADVLDLAETMVASDGLVYEPVVFD	Q7T2D4	2625,26	1,24E+06	7,17E+05	1,61E+06	High	657,07458	38,7942	3,29
AATMAAGTTAAIMAFLFDLKALVDMMSI	Q5PR34	2923,43	1,55E+06	7,26E+05	1,44E+06	High	585,49109	43,5259	2,67
KLEQQVDDLEGSLE	Q90339	1602,79	1,84E+06	7,27E+05	9,58E+05	High	801,89893	68,2989	2,97

KSPCRGSAGEAGAVFSAAPPADDTSAPVSG	A5WWA0	2817,31	4,35E+05	7,28E+05	6,10E+05	High	705,08911	16,5261	2,64
SVGVSGTEGVDRPGAIPHHGAGV	Q67FY3	2156,08	2,41E+06	7,31E+05	1,36E+06	High	719,37231	74,5473	2,52
VIGEHGDSSVPVWSGVNVA	Q9W7K5	1908,95	9,70E+05	7,33E+05	9,75E+05	High	954,9787	78,1024	2,93
GIADRMQKEITAL	P53479	1445,78	1,49E+06	7,33E+05	1,07E+06	High	723,39435	73,4457	2,27
IIDQDKSGFIEE	P02618	1393,68	5,64E+05	7,36E+05	5,95E+05	High	697,34644	51,3163	1,98
AENVLDILVDQ	Q1LXC9	1228,64	4,71E+05	7,40E+05	4,90E+05	High	614,82581	80,7158	1,96
GNAKTVRNDNSSRFGKFI	Q90339	2011,05	6,06E+05	7,42E+05	3,66E+05	High	503,51865	32,8211	3,26
LEEAEGTLEHEE	Q90339	1385,61	3,88E+05	7,44E+05	4,71E+05	High	693,30817	36,9029	3,01
KQGEKGESGPKGDKGDTGFPGL	A5PN28	2189,08	4,33E+05	7,48E+05	4,71E+05	High	548,02661	52,1476	2,68
QRLQGEVEDL	Q90339	1186,61	7,02E+05	7,48E+05	5,92E+05	High	593,80872	44,9749	2,93
KVEKEKHATENKVKNL	Q90339	1895,07	2,61E+05	7,52E+05	3,25E+05	High	474,5256	10,7384	5,46
SYVGDEAQSK	P83750	1083,50	1,12E+05	7,53E+05	4,38E+05	High	542,25226	17,4949	2,1
NTSLINTKKKLE	Q90339	1388,81	3,62E+05	7,56E+05	6,94E+05	High	463,60995	22,2871	3,38
RIINEPTAA	P47773	984,55	3,97E+05	7,56E+05	6,05E+05	High	492,77866	29,9656	2,17
ERGYSF	P83750	758,35	6,22E+05	7,64E+05	7,29E+05	High	379,67783	30,0161	1,96
KGEQGVTGKAGPMGERGL	C7DZK3	1771,91	3,41E+05	7,64E+05	7,68E+05	High	591,30914	35,4215	2,63
KTIDDLEDELY	P13104	1353,64	1,24E+06	7,66E+05	1,22E+06	High	677,32562	77,109	3,08
KSYELPD	P83750	851,41	6,25E+05	7,67E+05	7,79E+05	High	426,21219	32,1374	2,12
GFAGDDAPRAVFPSIVGRP	P83750	1929,00	1,82E+06	7,67E+05	5,85E+05	High	643,67224	77,0577	5,61
VTQASVATVIDETVPATI	A0MS83	1814,97	1,16E+06	7,68E+05	7,30E+05	High	605,65985	66,9283	2,33
TALEEAEGTL	Q90339	1033,50	6,32E+05	7,73E+05	6,55E+05	High	517,25775	66,516	2,03
KRNSQRVIDSM	Q90339	1333,70	3,34E+05	7,73E+05	5,38E+05	High	445,23956	15,8607	2,44
RKPSSADLK	Q8AXB3	1001,57	3,01E+05	7,75E+05	4,59E+05	High	501,28601	20,2751	2,05
GPHEGRVEVL	Q5RFW0	1092,58	9,60E+05	7,76E+05	6,47E+05	High	546,79095	74,8026	2
LAEKDEEMEQIKRNSQRVIDSM	Q90339	2649,30	2,90E+06	7,77E+05	2,46E+06	High	663,08118	70,9142	5,19
RADLSRELEEISE	Q90339	1546,77	9,13E+05	7,77E+05	1,06E+06	High	516,263	63,2992	3,4
RVPVADVS	Q5MJ86	842,47	3,42E+05	7,81E+05	4,78E+05	High	421,74191	25,4449	2,11
LSKIEDEQSLGAQLQ	Q90339	1658,86	5,76E+05	7,82E+05	1,01E+06	High	829,93427	56,4972	3,63
KVIPELNGK	Q5MJ86	997,60	4,19E+05	7,85E+05	3,94E+05	High	499,30731	26,5376	2,9
TVDGPSGKLWRDGRGASQNIIPA	Q5XJ10	2395,25	1,52E+06	7,96E+05	1,29E+06	High	799,08942	59,3681	2,38
GGAKRVIISAPSADAPM	Q5XJ10	1640,88	8,47E+05	7,97E+05	2,17E+05	High	547,63318	46,1428	2,76
KDIDDLELTL	Q90339	1174,62	1,22E+06	7,97E+05	1,21E+06	High	587,81494	84,5843	2,81
DLTEQLGETGKSIHEL	Q90339	1769,89	1,35E+06	7,98E+05	9,07E+05	High	590,63666	74,6148	3,97

DAIQRTEELEE	Q90339	1332,63	6,45E+05	7,99E+05	5,08E+05	High	666,81921	45,4698	2,47
RGRKVAEQELVDASE	Q90339	1686,88	7,66E+05	8,12E+05	1,07E+06	High	422,47653	25,8576	2,77
RDIQETLVHMMSSAAESKLAHWL	Q8JFV4	2669,32	1,17E+06	8,13E+05	7,86E+05	High	534,66858	45,7079	3,12
GLYGERVGGF	Q7SYK7	1054,53	6,15E+05	8,16E+05	5,72E+05	High	527,77167	57,5534	2,64
GNSHTMVAKATGLQ	L7VG99	1430,71	5,31E+05	8,16E+05	6,31E+05	High	715,86194	49,5667	2,07
KMTQIMFETF	P83750	1275,61	1,47E+06	8,17E+05	1,14E+06	High	638,31055	84,7328	2,04
TNLLSAVPYMGDTLVQWIWG	Q8SGQ9	2264,14	9,96E+05	8,18E+05	1,24E+06	High	566,79175	39,2185	2,94
VASAKSENGKSLYNAGVMVELP	Q1L8U8	2264,16	9,96E+05	8,18E+05	1,24E+06	High	566,79211	39,5173	2,88
LDKNKDPLNDSVVQ	Q90339	1584,82	4,55E+05	8,20E+05	4,91E+05	High	528,94727	39,0604	2,59
LGEQIDNLQRVKQKLEKE	Q90339	2168,20	1,42E+06	8,20E+05	1,56E+06	High	542,80811	54,4384	4,46
QEKKLRMDLE	Q90339	1289,69	6,00E+05	8,21E+05	7,73E+05	High	645,34845	23,5841	2,11
NRRIQLVEE	P13104	1156,64	6,24E+05	8,21E+05	7,21E+05	High	386,22086	26,8637	2,49
EGVNQLHFIPVDLGSPIVHCSV	A0A0R4IC37	2417,23	9,11E+05	8,23E+05	9,68E+05	High	484,24954	32,2741	2,73
PAEELTSINGVPVAAPGIDVWNPA	A9JRE2	2417,23	9,11E+05	8,23E+05	9,68E+05	High	484,24954	32,2741	2,62
VIISAPSADAPMF	Q5XJ10	1318,67	1,12E+06	8,23E+05	1,99E+05	High	659,84009	96,2892	1,94
LSRELEE	Q90339	875,45	6,55E+05	8,25E+05	6,68E+05	High	438,22864	22,7894	2,23
GWLDKNKDPLN	Q90339	1299,67	4,94E+05	8,26E+05	8,68E+05	High	650,34009	50,2461	2,09
DLEDALQRAK	Q6NWF6	1158,61	4,59E+05	8,27E+05	4,57E+05	High	579,81055	45,5813	2,32
LTVLADNIP	Q6JAN0	955,55	4,82E+05	8,32E+05	4,20E+05	High	478,27762	52,3771	2,06
FNFDNVLN	P22316	982,46	4,90E+05	8,33E+05	2,53E+05	High	491,73431	25,5505	2
QELEQRVIE	Q00IB7	1143,60	6,02E+05	8,36E+05	5,84E+05	High	572,30536	48,9058	1,91
VGDEAQSKRGIL	P83750	1272,69	4,06E+05	8,40E+05	4,31E+05	High	424,90298	26,2874	2,64
GGGRGGGFNRGGGGGRGGGFGGG	Q7ZVE0	1864,87	6,11E+05	8,47E+05	8,63E+05	High	466,97031	20,345	3,14
ILTERGYSF	P83750	1085,56	5,89E+05	8,48E+05	8,42E+05	High	543,28674	57,7157	1,92
GPGGLPGLEGDQGPVGPAGAPG	A0MSJ1	1856,91	5,50E+05	8,53E+05	8,14E+05	High	928,96826	53,4635	2,08
KLEAQKLRDEQKL	P85001	1598,92	9,94E+05	8,55E+05	9,91E+05	High	533,64771	43,6591	2,36
DLAGRDLTDYLM	P83750	1398,66	6,67E+05	8,56E+05	7,32E+05	High	699,83307	84,8702	2,61
PSIVHRK	P83750	836,51	4,42E+05	8,56E+05	5,42E+05	High	418,76016	24,5561	2,18
SDLTEQLGETGKSIHE	Q90339	1743,84	7,73E+05	8,57E+05	1,01E+06	High	581,953	53,5489	4,72
EQIDNLQRVKQK	Q90339	1498,83	4,80E+05	8,58E+05	5,85E+05	High	500,28403	20,8964	3,1
RVFDKEGNGTV	Q6P0G6	1221,62	6,20E+05	8,59E+05	2,29E+05	High	407,88037	23,1621	3,11
TIIDQNRDGIISKDDLRDVL	O93409	2299,23	4,17E+06	8,60E+05	1,21E+06	High	767,08252	79,4368	4,2
LSSGSRKSRGLYPPAL	Q6ZM89	1688,94	4,53E+05	8,68E+05	2,01E+06	High	563,65454	26,2601	2,79

NLDKKQRNFDKVLA	Q90339	1688,94	4,53E+05	8,68E+05	2,01E+06	High	563,65381	25,7605	3,04
LEEISERLEEA	Q90339	1317,65	7,98E+05	8,70E+05	1,21E+06	High	659,33203	56,3599	3,29
KMWNKDNGKVFSSIEPQANINDVCL	Q802W4	2923,41	2,10E+06	8,71E+05	2,01E+06	High	488,07733	42,6095	3,35
KKNQTTSTAATTDPGPLYFSPQGSPEIS	O42365	2923,43	2,10E+06	8,71E+05	2,01E+06	High	488,07733	42,6095	3,19
HTTRSPSASIQAGAAGDALDLSL	U3JAG9	2239,13	5,71E+05	8,71E+05	7,70E+05	High	560,53656	31,2817	2,91
MHTTRSPSASIQAGAAGDALDLSL	U3JAG9	2239,13	5,71E+05	8,71E+05	7,70E+05	High	560,53656	31,2817	2,92
VTNNRLFSEIQDTLGTFGLN	Q7ZVM1	2239,14	5,71E+05	8,71E+05	7,70E+05	High	560,53723	31,7988	3,19
KIKIIAPPERKY	P83750	1455,90	8,11E+05	8,74E+05	6,91E+05	High	485,97565	27,3211	2,92
ERGMKVIENR	P13104	1231,66	1,95E+05	8,74E+05	2,76E+05	High	411,22546	15,5271	2,86
EPVWAIGTGKTASPQ	Q90XG0	1541,80	6,77E+05	8,75E+05	8,48E+05	High	771,40344	58,8107	2,93
KEITAL	P53479	674,41	9,65E+04	8,77E+05	3,83E+05	High	337,70865	26,6863	2,03
KETLAL	Q7SY09	674,41	9,65E+04	8,77E+05	3,83E+05	High	337,70865	26,6863	2,04
ESAAKVLMAPPSMVSTEQRQHAE	Q802D3	2497,22	1,62E+06	8,77E+05	1,86E+06	High	500,24951	36,858	3,52
LAAGQKGDEGVGGRSPVEEEELRN	Q6NXD8	2497,23	1,62E+06	8,77E+05	1,86E+06	High	500,24924	37,202	3,36
RTLDMLIRSLYQDL	A5PF44	1752,93	1,58E+06	8,79E+05	1,42E+06	High	584,98077	31,6193	2,34
YPQTKTYFSH	Q90487	1271,61	5,02E+05	8,80E+05	7,35E+05	High	636,30798	33,4579	2,09
LDHALNDMTS	P13104	1116,50	3,88E+05	8,81E+05	3,51E+05	High	558,75513	36,5395	2,12
RGILTL	P83750	672,44	8,50E+05	8,86E+05	2,80E+06	High	336,72443	51,8047	1,97
SERGMKVIE	P13104	1048,55	3,03E+05	8,86E+05	3,95E+05	High	350,1875	20,4568	2,34
KVAEQELVDA	Q90339	1101,58	6,23E+05	8,89E+05	4,23E+05	High	551,29529	35,7663	2,38
ISDLTEQLGE	Q90339	1104,54	5,45E+05	8,96E+05	7,12E+05	High	552,77637	63,4448	2,06
RVIISAPSAD	Q5XJ10	1028,57	6,96E+05	8,97E+05	5,98E+05	High	514,79218	38,1764	2,69
FTNEKLQQFFNH	Q90339	1552,75	8,32E+05	9,02E+05	9,36E+05	High	518,25781	63,4383	2,96
INRNTTAEAKVEEISGVA	F1QWK4	1901,99	6,34E+05	9,02E+05	9,41E+05	High	634,67413	30,5493	2,35
STIPFSLMKVHLMVAVE	Q9W7R3	1902,02	6,34E+05	9,02E+05	9,41E+05	High	634,6745	30,9158	2,42
KSPEGLLGVLGHMLSGKSL	Q2PW47	1939,07	5,92E+05	9,19E+05	2,24E+06	High	485,52042	11,342	2,78
FPMKALGYFAVVT	Q5U3A7	1443,77	4,41E+05	9,20E+05	1,03E+06	High	481,93314	31,3196	2,42
RLEDEEEIN	Q90339	1146,53	5,31E+05	9,26E+05	6,51E+05	High	573,7699	28,4247	2,67
ALIKEGCEGTENRLILH	Q0P4A4	1953,02	6,38E+05	9,29E+05	9,64E+05	High	651,68176	29,9664	2,41
PPDVAGNVDYK	O93409	1174,57	5,44E+05	9,41E+05	3,07E+05	High	587,79266	35,8771	3,85
YPAGGTLPPMYPNPQAQSQVY	Q1ECZ4	2295,08	5,80E+05	9,44E+05	6,14E+05	High	765,69714	30,4526	2,39
TLFQPSFIGM	P53479	1140,58	1,98E+06	9,44E+05	1,17E+06	High	570,79291	113,3798	2,15
DDMEKIWHHT	P83750	1311,58	5,73E+05	9,45E+05	5,97E+05	High	437,86545	40,0149	2,35

APSADAPMFVM	Q5XJ10	1136,51	5,88E+05	9,47E+05	1,39E+06	High	568,76111	90,4173	2,87
YESELSIR	Q7ZTS4	996,50	5,74E+05	9,48E+05	6,36E+05	High	498,755	39,2521	2,38
TPNVSVVDL	Q5XJ10	943,51	7,41E+05	9,53E+05	3,43E+05	High	472,26007	75,1242	1,91
MYPGIADRMQ	P83750	1181,54	6,47E+05	9,54E+05	1,04E+06	High	591,27771	51,1819	2,49
KTIDDLE	P13104	833,43	4,89E+05	9,56E+05	3,64E+05	High	417,21753	28,6852	2,03
EYDESGPSIVHRK	P83750	1516,74	5,40E+05	9,57E+05	7,25E+05	High	506,2522	23,1416	3,98
IQRTEELEE	Q90339	1146,56	6,40E+05	9,58E+05	4,33E+05	High	573,78693	25,5584	2,08
LDDISKIPEKSVIL	Q7SYK7	1569,91	7,00E+05	9,59E+05	6,11E+05	High	523,9762	71,4187	2,88
KAKTKLEQQVDDLEGSLE	Q90339	2031,06	3,56E+06	9,62E+05	1,63E+06	High	677,69269	68,7943	4,48
DVAGNVDYK	O93409	980,47	4,71E+05	9,67E+05	2,31E+05	High	490,73907	27,7582	2,01
SGGTTMYPGIADR	P83750	1325,62	6,73E+05	9,67E+05	7,80E+05	High	663,31335	44,0104	3,4
EGSLEQEKKLRMDLE	Q90339	1804,91	1,09E+06	9,69E+05	1,14E+06	High	602,31006	45,0943	4,19
ELVDASERVGLL	Q90339	1300,71	1,58E+06	9,71E+05	1,63E+06	High	650,85925	77,0181	2,74
IDNLQRVKQKLE	Q90339	1483,86	6,44E+05	9,72E+05	1,12E+06	High	495,29303	33,787	3,9
DLAGRDLTDYLMK	P83750	1526,75	5,27E+05	9,79E+05	5,53E+05	High	763,88098	69,6179	2,52
AVSEGTKAVTKY	Q6PC60	1253,67	4,79E+05	9,79E+05	6,71E+05	High	627,34161	20,463	2,57
EKTIDDLEDELY	P13104	1482,68	2,70E+06	9,83E+05	2,16E+06	High	741,8468	82,4596	2,52
GSLEQEKKLRMD	Q90339	1433,74	5,92E+05	9,85E+05	7,16E+05	High	478,58731	24,2648	3,39
LKEADITAAL	P05939	1044,59	8,69E+05	9,97E+05	9,22E+05	High	522,80225	63,5745	2,07
VDDLEGSLEQEKKLRMDLE	Q90339	2247,12	3,93E+06	9,97E+05	2,64E+06	High	749,71252	71,8899	4,48
NVKNEELEAM	O93409	1176,56	5,21E+05	9,99E+05	8,92E+05	High	588,78363	36,2993	2,64
NDNSSRFGKF	Q90339	1171,55	7,77E+05	1,00E+06	1,42E+05	High	391,18927	29,4603	2,51
RDLEESTLQ	Q90339	1090,54	5,79E+05	1,00E+06	5,44E+05	High	545,77411	31,9713	2,84
SGGTTMYPGIADRMQKE	P83750	1841,85	8,69E+05	1,00E+06	1,08E+06	High	614,62347	47,9726	4,4
STDTTHPPRSPHPPP	Q2THW0	1623,79	6,22E+05	1,01E+06	7,35E+05	High	812,3996	58,7903	1,99
VGVEVHVNVEFVKLL	F1RA39-1	1680,97	7,11E+05	1,01E+06	1,98E+06	High	560,99799	36,1985	2,87
AEWKQKYEE	Q90339	1210,57	5,57E+05	1,01E+06	9,46E+05	High	605,79248	21,6642	2,61
APIHAEAPE	Q9PTY0	934,46	5,91E+05	1,01E+06	6,59E+05	High	467,73618	22,859	2,2
AFTIIDQNRDGIISK	O93409	1690,91	7,49E+05	1,01E+06	1,33E+06	High	564,30841	61,3004	4,22
IVGRPRHQGVMVGM	P83750	1552,82	6,18E+05	1,01E+06	9,88E+05	High	518,27948	25,0165	2,44
KDDLRDVLAS	O93409	1131,60	5,54E+05	1,02E+06	7,03E+05	High	566,3053	44,1223	3,13
NFAQLIKDRGI	Q8JH70	1274,72	6,27E+05	1,02E+06	7,04E+05	High	425,58163	24,413	2,45
KAMVMVLPPSEPRIT	B0S5G3	1684,91	5,37E+05	1,02E+06	8,98E+05	High	562,30957	35,9395	2,3

KAADESERGMKVIE	P13104	1562,78	6,01E+05	1,03E+06	6,40E+05	High	521,6015	24,4673	2,67
KMEGDLNEME	Q90339	1195,50	5,80E+05	1,03E+06	3,37E+05	High	598,25421	37,6137	2,18
LGEQIDNLQR	Q90339	1185,62	5,40E+05	1,03E+06	7,21E+05	High	593,31616	43,2973	3,14
VFCPFRTSSVELMAMHLQVNH	F1QQA8	2503,20	5,42E+05	1,03E+06	7,83E+05	High	835,07892	36,385	2,37
LPMELERPPSNASAATRTSGSTTE	B8JKP6	2503,21	5,42E+05	1,03E+06	7,83E+05	High	835,07874	36,4458	2,34
SKIEDEQSLGAQLQKKIKEL	Q90339	2285,27	2,41E+06	1,03E+06	5,11E+06	High	572,07477	70,402	4,21
GFAGDDAPRAVFPSIVGRPRHQGVMVG	P83750	2793,44	4,90E+06	1,04E+06	1,06E+06	High	699,11652	67,1105	2,84
GTLLKPNMVTA	Q8JH70	1144,64	6,95E+05	1,04E+06	1,01E+06	High	572,82544	52,2993	2,35
EQIDNLQRVKQKLEKEKSEYKME	Q90339	2893,51	2,21E+06	1,04E+06	1,90E+06	High	579,50958	54,9553	3,87
GDDAPRAVFP	P83750	1044,51	5,65E+05	1,04E+06	2,33E+05	High	522,76093	50,2734	2,48
RVAPEEHPVLL	P83750	1259,71	9,32E+05	1,05E+06	1,98E+06	High	420,57648	42,2387	2,59
DKENALDRAE	P13104	1160,55	1,93E+05	1,05E+06	5,85E+05	High	580,7818	17,3752	2,31
GIFLYMGITSLTGI	A0A096X8J7	1485,80	8,80E+05	1,05E+06	8,61E+05	High	495,93741	35,2012	3,29
YPGIADRMQKEITA	P53479	1592,81	1,55E+06	1,05E+06	3,78E+05	High	531,6098	57,5578	2,34
GMDELSEEDKLTVA	Q9PTY0	1536,71	1,05E+06	1,05E+06	7,81E+05	High	768,85944	66,9054	3
FTIIDQNRDGIIS	O93409	1491,78	8,13E+05	1,05E+06	9,53E+05	High	746,3949	73,9311	3,06
KIKIIAPPERKYS	P83750	1542,94	9,61E+05	1,05E+06	4,59E+05	High	514,98529	26,3409	3,34
AGRDLTDYLMKI	P83750	1395,73	9,15E+05	1,05E+06	7,97E+05	High	465,91647	67,9369	2,51
MKAKQLEALRISRTQ	Q803A6	1773,02	8,75E+05	1,06E+06	1,33E+06	High	444,00693	26,018	2,66
KSVAADFLE	Q561X9	979,51	6,37E+05	1,06E+06	7,07E+05	High	490,25977	49,2962	1,93
MLAQQSSLGQPLLIPLSMAGSV	P31367	2257,19	1,07E+06	1,06E+06	1,70E+06	High	565,05707	27,7784	2,73
KEAFTIIDQNRDGIISK	O93409	1948,05	9,98E+05	1,07E+06	1,38E+06	High	650,02277	53,5306	6,22
SIVGRPRHQGVMVGM	P83750	1623,86	1,16E+06	1,08E+06	1,53E+06	High	541,95856	35,2931	3,58
SLEKSYELPDGQVITIGNE	P83750	2092,04	6,93E+06	1,08E+06	2,95E+06	High	1046,52844	94,8079	4,57
TDAETKAFLKA	P09227	1194,64	5,33E+05	1,09E+06	8,34E+05	High	597,82385	34,4418	2,52
KLDKENALDRAEQA	P13104	1600,83	7,06E+05	1,09E+06	1,05E+06	High	800,92035	27,3322	3,76
EDKYEEEIKVL	P13104	1394,70	9,44E+05	1,10E+06	1,53E+06	High	697,85742	58,3097	2,97
SKRGILTL	P83750	887,57	5,11E+05	1,10E+06	1,38E+06	High	444,28876	33,5189	2,13
LEQQVDDLEGSL	Q90339	1345,65	1,43E+06	1,10E+06	1,20E+06	High	673,3299	78,6479	2,69
AGRDLTDYLMK	P83750	1282,65	7,30E+05	1,11E+06	1,07E+06	High	428,22113	57,2308	3,43
STRGLICGLGAGVAEAVVV	F1R4U0	1828,99	7,08E+06	1,11E+06	4,43E+06	High	610,33319	76,9231	2,35
MGQKDSYVGDEAQSKRGILT	P83750	2183,08	8,35E+05	1,11E+06	1,10E+06	High	546,52673	38,5869	5,21
WDDMEKIWHHT	P83750	1497,66	1,09E+06	1,12E+06	1,08E+06	High	499,89227	57,2161	2,38

ISERLEEAGGAT	Q90339	1232,61	5,36E+05	1,12E+06	1,06E+06	High	616,8111	32,0935	3,1
TFYNELRVAPEEHPTLL	P53479	2029,04	4,54E+06	1,12E+06	2,29E+06	High	677,0191	82,8323	2,56
DLERSLL	Q6NYU2	845,47	7,99E+05	1,13E+06	9,64E+05	High	423,24094	48,378	1,94
IDERLSL	Q0P496	845,47	7,99E+05	1,13E+06	9,64E+05	High	423,24152	48,358	2,11
RVIISAPSADAPMFV	Q5XJ10	1573,84	2,52E+06	1,13E+06	1,61E+06	High	787,42676	85,8445	3,02
EYKEAFTIIDQNRDGIISKD	O93409	2355,18	2,09E+06	1,13E+06	2,05E+06	High	785,73401	68,2184	4,34
KVLMADMVKLNSLLN	Q56A40	1688,94	8,06E+05	1,14E+06	2,57E+06	High	422,9931	26,2142	2,95
KLVESDMLEIL	Q6DGE9	1289,70	1,51E+06	1,14E+06	1,95E+06	High	645,35962	86,4726	1,9
GFAGDDAPRAVFPSIVG	P83750	1675,84	2,12E+06	1,15E+06	1,58E+06	High	838,42712	89,9605	3,96
LDDISKIPE	Q7SYK7	1029,55	4,68E+05	1,15E+06	4,37E+05	High	515,27808	48,5964	2,36
FNGPYNPPGVSPQPHSAY	Q7T163	1928,89	1,00E+06	1,15E+06	9,93E+05	High	643,63538	52,7277	2,35
GDDAPRAVFPS	P83750	1131,54	6,58E+05	1,16E+06	1,78E+06	High	566,27637	45,7197	2,28
IRSALDDY	E7FAW3	952,47	7,57E+05	1,16E+06	5,23E+05	High	476,74179	38,2358	1,99
KVIPELN	Q5MJ86	812,49	6,77E+05	1,16E+06	4,68E+05	High	406,74884	38,491	2,23
GFAGDDAPRAVFPSIVGRPRHQGVM	P83750	2637,35	3,17E+06	1,16E+06	2,18E+06	High	528,27692	64,2774	3,81
DQNRDGIISKDDLRDVLA	O93409	2043,05	1,34E+06	1,16E+06	1,38E+06	High	681,68909	69,1789	4,35
PPPPPPPAPAPAVNNPAGSVRK	Q6PFT9	2128,17	3,83E+06	1,17E+06	1,78E+06	High	532,79315	70,9788	2,85
AADFGTNYQLPSLDYAINHYGQPDVA	F1RA39-1	2840,32	8,67E+05	1,17E+06	1,09E+06	High	710,83722	28,5219	2,68
KEAFTIIDQ	O93409	1064,56	6,31E+05	1,17E+06	7,96E+05	High	532,78607	54,0266	2,88
TFYNELR	P83750	942,47	8,45E+05	1,18E+06	8,38E+05	High	471,73901	42,9996	1,9
RVIQYFAT	Q90339	997,55	9,83E+05	1,18E+06	1,16E+06	High	499,27838	49,3241	1,92
RLDIAGRDI	P83750	1028,58	7,39E+05	1,18E+06	9,03E+05	High	514,79742	43,3526	2,44
QRLGSGGGGAVGAGDVPKEDLNQR	Q5XJ54	2338,19	7,19E+05	1,20E+06	1,20E+06	High	780,06647	37,8141	2,75
SADDVKKAF	P02618	980,50	5,07E+05	1,20E+06	7,94E+05	High	490,75729	23,0488	2,72
IIDQNRDGIISKDDLRDVLA	O93409	2269,21	3,65E+06	1,20E+06	2,25E+06	High	568,06091	72,1817	4,65
VKQKLEKEKSEYKME	Q90339	1897,01	8,82E+05	1,20E+06	1,42E+06	High	475,00946	13,3166	2,73
EMVASNAEE	A8CVX7	995,40	6,86E+05	1,21E+06	1,22E+06	High	498,19965	31,4881	2,12
VTWYDNEFGYSN	Q5XJ10	1494,62	1,87E+06	1,21E+06	1,42E+06	High	747,81421	90,4495	2,67
TKQRLQGEVEDL	Q90339	1415,75	7,69E+05	1,21E+06	9,88E+05	High	472,58868	34,6125	2,38
KNNTPSVDTTRADKTPL	E7F568	1857,97	8,85E+05	1,21E+06	1,08E+06	High	619,99158	58,1604	3
TELMNFGANAGILSRLD	C6FG12	1821,92	1,07E+06	1,23E+06	1,06E+06	High	456,23651	28,1165	2,7
EEEMREL	Q58EE9	935,41	7,40E+05	1,23E+06	9,22E+05	High	468,21198	31,7156	1,9
EAPLNPKANREKMTQIMFE	P83750	2247,13	2,47E+06	1,23E+06	2,07E+06	High	749,71503	57,9199	3,1

LAEKDEEMEQIK	Q90339	1462,71	8,06E+05	1,23E+06	7,64E+05	High	488,24332	29,8515	3,32
DVGHVMPGALM	Q6NVJ5	1126,54	5,12E+05	1,24E+06	1,90E+06	High	563,77222	58,4103	2,1
HLDEAVRGQ	Q90339	1024,52	5,40E+05	1,24E+06	8,34E+05	High	512,76416	23,4191	2
HVRAHSSPASLQLGAVSPGALTSM	Q1L8J7	2374,23	1,72E+06	1,24E+06	2,57E+06	High	594,3158	51,8361	2,98
RAKRKLEGDLKL	Q90339	1426,89	1,70E+06	1,24E+06	3,05E+06	High	357,47809	17,0407	2,85
LAGRDLTDYLMK	P83750	1395,73	1,12E+06	1,25E+06	9,64E+05	High	465,91595	68,0083	2,87
KDDLRDVLATM	Q66I73	1276,66	1,22E+06	1,25E+06	1,43E+06	High	426,22491	74,3448	3,09
NWDDMEK	P83750	937,37	9,08E+05	1,25E+06	8,84E+05	High	469,19092	35,4397	2,07
VDGVQEQERQQQAGKNLGTTK	Q568F6	2314,17	9,75E+05	1,25E+06	1,45E+06	High	463,63843	26,1275	2,87
ASERVGLL	Q90339	844,49	7,26E+05	1,26E+06	1,15E+06	High	422,74942	40,2876	2,55
TVTNNMKSL	P13104	1007,52	7,24E+05	1,26E+06	1,15E+06	High	504,26468	29,1184	2,35
QNIPRNVLEGTYGINI	Q6NY89	1800,96	1,64E+06	1,26E+06	2,79E+06	High	450,99866	25,7525	2,66
KNWAKAASFVTSPPLSP	Q52PJ5	1800,96	1,64E+06	1,26E+06	2,79E+06	High	450,99866	25,7525	2,72
ERVQAGDVITIDKATGK	P83571	1800,98	1,64E+06	1,26E+06	2,79E+06	High	450,99908	26,4488	2,67
NKVNGVPTYTLAPVQVT	A4QP16	1800,99	1,64E+06	1,26E+06	2,79E+06	High	450,99863	27,7251	2,8
SMFEQSQIQ	O93409	1097,49	6,34E+05	1,26E+06	1,07E+06	High	549,25177	55,3488	2,34
NVLSGGTTMYPGIADRM	P53479	1798,85	1,09E+06	1,27E+06	8,99E+05	High	899,92883	70,896	2,8
LDIAGRDI	P83750	872,48	7,03E+05	1,27E+06	7,63E+05	High	436,7468	55,7952	2
AIQRTEELE	Q90339	1088,56	8,61E+05	1,28E+06	1,06E+06	High	544,78418	25,5417	2,97
EQEKKLRMDLE	Q90339	1418,73	7,53E+05	1,28E+06	9,93E+05	High	473,58331	27,9626	2,33
KGKDFLQNELSSSQL	Q98SJ5	1693,88	9,26E+05	1,28E+06	1,02E+06	High	565,29938	29,7376	2,32
VLAHVMKLLN	Q803X1	1153,68	1,12E+06	1,28E+06	7,87E+05	High	577,3465	73,5151	2
LVDASERVGLLH	Q90339	1308,73	6,61E+05	1,29E+06	3,62E+06	High	654,86926	48,2641	3,05
AFRVPTPNVS	Q5XJ10	1087,59	6,08E+05	1,29E+06	1,03E+06	High	544,29987	54,7316	2,3
TLDDLQAEEDKVNTL	Q90339	1703,83	1,39E+06	1,29E+06	2,27E+06	High	852,42285	74,888	2,2
QIKRNSQRVIDSM	Q90339	1574,84	1,13E+06	1,29E+06	1,51E+06	High	525,62134	22,1689	2,87
AQYEDIANR	Q6NWF6	1079,51	9,57E+05	1,29E+06	7,27E+05	High	540,26239	29,6267	1,9
ADESERGMKVI	P13104	1234,61	5,93E+05	1,29E+06	8,58E+05	High	617,80927	30,0382	2,23
SLSAGPATQPVQPNEPAAPMGPNPAPE	Q6NYI0	2625,26	2,35E+06	1,30E+06	2,81E+06	High	525,86072	39,0778	3,47
QELEAQLENQGESIHLFSEDVIP	Q6DHF7	2625,27	2,35E+06	1,30E+06	2,81E+06	High	525,86072	38,6394	3,14
AAQPAREQQQAASAAPHTPQTPSQPG	Q06725	2625,28	2,35E+06	1,30E+06	2,81E+06	High	525,86176	38,7015	2,9
MELGVAAAPGSSSSGLGVGPVGGALDAGPGS	Q08CH8	2625,28	2,35E+06	1,30E+06	2,81E+06	High	525,86035	39,0465	3,47
YPKPGDVPSASPASPRAQQNWLSTA	F6NSX9	2625,31	2,35E+06	1,30E+06	2,81E+06	High	525,86176	38,7015	3,01

TEAPLNPKANREKMTQI	P83750	1941,02	8,13E+05	1,30E+06	7,10E+05	High	647,68048	34,8681	3,22
LEEELKSLMEKSV	Q6PBQ2	1534,80	7,04E+05	1,31E+06	1,01E+06	High	512,27667	32,0022	2,93
TVRNDNSSRFGKFIR	Q90339	1796,95	9,75E+05	1,31E+06	1,22E+06	High	449,99463	28,5919	3,76
KVEALLKN	Q7ZU92	914,57	6,47E+05	1,31E+06	9,56E+05	High	457,78824	27,457	1,97
KGELKLRA	O13034	914,58	6,47E+05	1,31E+06	9,56E+05	High	457,78833	27,4412	2,11
DNGSGLVKAGFAGDDAPRAVFPS	P53479	2248,10	2,28E+06	1,31E+06	1,12E+06	High	750,03986	78,3583	4,18
QAIQRADDI	Q29RB1	1029,53	6,67E+05	1,33E+06	7,52E+05	High	515,27203	31,6534	2,11
DLEDALQR	Q6NWF6	959,48	6,62E+05	1,33E+06	5,86E+05	High	480,2446	48,9725	2,17
NDNSSRFGKFIR	Q90339	1440,73	9,40E+05	1,33E+06	1,12E+06	High	480,9173	32,3865	3,23
ISDLTEQLGETGK	Q90339	1390,71	8,11E+05	1,34E+06	1,11E+06	High	695,85803	50,5292	3,15
KQRADLSRELEEISE	Q90339	1802,92	2,02E+06	1,34E+06	2,08E+06	High	451,48782	50,9118	3,65
LDKENALDRAEQA	P13104	1472,73	6,69E+05	1,35E+06	1,71E+06	High	736,87201	32,6708	3,42
GGAKRVIISAPSADAPMF	Q5XJ10	1787,95	1,88E+06	1,35E+06	2,22E+05	High	596,65558	65,3615	4,09
LPTQALGLA	C5J7W8	883,52	8,53E+05	1,35E+06	8,38E+05	High	442,26727	38,1164	1,97
LSAAPAIQL	F1RD40	883,52	8,53E+05	1,35E+06	8,38E+05	High	442,26746	37,6948	1,93
IIAPPER	P83750	795,47	6,87E+05	1,37E+06	5,17E+05	High	398,24115	26,616	2,04
EDGRPAQINERKAPLCDCTC	Q5W8I8	2390,06	1,04E+06	1,37E+06	4,16E+05	High	797,36554	37,3154	2,31
GSIKKEFLEE	O93409	1179,63	8,20E+05	1,39E+06	1,17E+06	High	590,3186	33,6336	2,45
GLSDIRESYELVEFAK	E7FCP8	1855,94	1,18E+06	1,39E+06	1,52E+06	High	619,31915	28,7422	2,51
KALDVDASGFIEEEE	Q804W2	1651,77	1,24E+06	1,40E+06	1,33E+06	High	826,39056	54,6878	2,01
VGRPRHQGVMVGM	P83750	1423,74	1,10E+06	1,40E+06	1,68E+06	High	356,69144	24,9305	2,93
AQRWPQEPQEGVTP	F5HSE3	1622,79	8,27E+05	1,41E+06	1,08E+06	High	541,59991	45,1229	2,37
RVIISAPSA	Q5XJ10	913,55	8,72E+05	1,41E+06	1,18E+06	High	457,27835	37,6809	2,38
KLLAVVQDQCVEIRSA	Q5TYW4	1828,99	1,13E+06	1,41E+06	1,31E+06	High	610,33539	28,9617	2,4
ESKKTGNGVVILQIEDV	F1QSQ0	1829,00	1,13E+06	1,41E+06	1,31E+06	High	610,33539	29,2752	2,39
EKDNTNVLI	A2BGG1	1045,55	6,27E+05	1,41E+06	#DIV/0!	High	523,28131	42,2981	2,08
EKSYELPDGQVITIG	P83750	1648,84	3,96E+06	1,42E+06	3,00E+06	High	824,92706	93,3276	2,6
ELPDGQVITIGNERFR	P83750	1843,97	2,80E+07	1,43E+06	2,63E+06	High	615,32782	69,5426	3,64
ASSKTLEMGRSSKTNPLNAMGLD	Q6PCS4	2424,19	1,51E+06	1,43E+06	1,77E+06	High	606,80402	41,8634	2,66
DAGLEVEVKDPPKGMIPPGTQMV	F1RD40	2424,21	1,51E+06	1,43E+06	1,77E+06	High	606,80389	41,9299	2,65
KTEIADLN	Q6NWF6	903,48	9,20E+05	1,43E+06	1,38E+06	High	452,24438	28,6817	2,23
MEQIKRNSQRVIDSM	Q90339	1834,93	1,41E+06	1,43E+06	2,03E+06	High	612,31525	36,2749	4,22
KLEPLM	O42363	730,42	9,51E+05	1,44E+06	3,58E+05	High	365,71338	40,0497	2,08

GVGMGLIGPGGI	O42477	1027,56	6,91E+05	1,44E+06	7,34E+05	High	514,28162	31,7412	1,93
KDIDDL	Q90339	718,36	8,32E+05	1,44E+06	8,72E+05	High	359,68539	28,8351	2,13
GRDLTDYLMK	P83750	1211,61	6,99E+05	1,44E+06	1,11E+06	High	404,5426	56,7168	2,74
GIHETTFNSIM	P83750	1249,59	1,06E+06	1,45E+06	1,02E+06	High	625,29913	62,5068	3,09
YEDIANRS	Q6NWF6	967,45	7,89E+05	1,46E+06	8,62E+05	High	484,22888	21,3155	2,25
TNKLISLFEAIERGLI	A0A8M9PQ61	1817,05	2,10E+06	1,46E+06	2,38E+06	High	606,35254	57,4347	2,44
EEAEGTLEHE	Q90339	1143,48	8,72E+05	1,47E+06	9,76E+05	High	572,24518	22,6826	2,64
NVLSGGTTMYPGIADRMQKE	P53479	2168,05	2,18E+06	1,48E+06	1,59E+06	High	723,35675	66,5344	4,46
LEQQVDDLEGS	Q90339	1232,56	8,35E+05	1,48E+06	9,96E+05	High	616,78845	48,9773	2,72
IKKEFLEEL	O93409	1148,66	1,01E+06	1,48E+06	1,50E+06	High	574,83362	55,6208	2,54
ETVKAAKDAV	Q1LY77	1031,57	8,78E+05	1,48E+06	5,60E+05	High	516,29199	29,4757	2,09
AAAALKNAGQ	Q5PYH5	914,51	8,05E+05	1,49E+06	1,00E+06	High	457,75842	37,4131	1,92
KEAFTII	O93409	821,48	1,06E+06	1,50E+06	1,68E+06	High	411,24326	58,7217	2,11
PMDLKNFPMDVQTCIMQLESFGYT	O93430	2865,30	1,43E+06	1,51E+06	7,37E+05	High	573,86945	10,7416	3,8
PVNAGNMTDTKAAGEVPMETDVASSPLF	Q1ECW2	2865,33	1,43E+06	1,51E+06	7,37E+05	High	573,86938	10,7505	3,9
AAAQMQHMATINGLPGAPMTPTSGGSTPPG	Q6DGV1	2865,33	1,43E+06	1,51E+06	7,37E+05	High	573,86938	10,7505	4,08
DDLRDVLAS	O93409	1003,51	7,32E+05	1,51E+06	9,34E+05	High	502,258	62,032	1,9
LAGRDLTDY	P83750	1023,51	8,33E+05	1,51E+06	8,23E+05	High	512,26056	43,5637	2,91
QRVIDSM	Q90339	848,43	8,04E+05	1,52E+06	8,37E+05	High	424,71979	28,1005	2,62
GAQWGDEGKGKVVDLL	Q568F6	1671,87	2,19E+06	1,52E+06	1,87E+06	High	557,96283	68,5061	2,58
KAADESERGMKVIEN	P13104	1676,83	8,92E+05	1,53E+06	1,01E+06	High	559,61578	24,4294	3,53
QKEVEDL	A2BGD5	860,44	8,34E+05	1,54E+06	8,53E+05	High	430,72339	26,4986	2,42
SLGQDLDL	P59679	860,44	8,34E+05	1,54E+06	8,53E+05	High	430,72281	26,2419	2,26
DAPRAVFPS	P83750	959,49	6,63E+05	1,54E+06	1,38E+06	High	480,25253	44,9886	2,43
RELEEISERL	Q90339	1273,67	1,28E+06	1,54E+06	1,75E+06	High	637,34216	48,2831	2,87
KVWSSASWKML	Q9I9H8	1322,69	1,79E+06	1,55E+06	2,89E+06	High	661,84943	52,1792	1,91
HALNDMTSI	P13104	1001,47	8,25E+05	1,55E+06	8,10E+05	High	501,24097	46,813	2,46
IKKEFLEE	O93409	1035,57	8,02E+05	1,56E+06	1,54E+06	High	518,29126	27,9501	1,99
KLMASTQPFKEIIHNTSLAEL	Q7ZU92	2371,27	1,58E+06	1,56E+06	1,92E+06	High	593,56763	30,4419	2,68
PEIVISVAGLLN	Q1LXK4	1224,72	1,54E+06	1,57E+06	1,30E+06	High	612,86456	76,7845	2,31
DRMQKEITAL	P53479	1204,64	8,43E+05	1,57E+06	1,29E+06	High	602,82257	41,7157	3,04
WADLSPGSGPVK	Q90487	1213,62	6,94E+05	1,57E+06	8,92E+05	High	607,31543	44,3279	2
RELEEISERLEE	Q90339	1531,76	7,64E+05	1,58E+06	1,09E+06	High	511,2598	51,7654	3,43

LVEYGSNDILGSVRT	Q5RHH4	1622,84	1,22E+06	1,58E+06	1,78E+06	High	541,62061	54,2739	2,45
YETDAIQRTEE	Q90339	1354,61	6,56E+05	1,59E+06	1,15E+06	High	677,81177	33,1978	3,25
AVGKVIPEL	Q5MJ86	925,57	7,40E+05	1,60E+06	5,24E+05	High	463,29099	57,5654	2,23
EAPLNPKANREKMTQIM	P83750	1971,02	1,40E+06	1,62E+06	1,18E+06	High	657,67822	41,4025	2,45
RGQLALVLL	B3DIY3	982,64	1,10E+06	1,62E+06	1,32E+06	High	491,82526	61,4463	1,96
TKLEQQVDDL	Q90339	1188,61	8,51E+05	1,63E+06	1,20E+06	High	594,81042	46,3937	3,17
GNAKTVRNDNSSRFGKF	Q90339	1897,96	1,70E+06	1,63E+06	3,00E+05	High	475,24783	21,2461	4,11
TDAETKAF	P09227	882,42	3,46E+05	1,63E+06	8,53E+05	High	441,71536	22,412	2,05
RGRKVAEQELVDAS	Q90339	1557,83	1,12E+06	1,64E+06	1,65E+06	High	519,95197	22,697	2,45
EEISERLEE	Q90339	1133,53	1,39E+06	1,64E+06	1,90E+06	High	567,27203	38,2381	2,65
KTIDDLEDELYAQ	P13104	1552,74	3,12E+06	1,65E+06	3,63E+06	High	776,87384	78,2396	3,34
VDDLEGSLEQE	Q90339	1233,55	9,89E+05	1,66E+06	1,27E+06	High	617,27875	61,3582	2,18
LEKTIDDLEDE	P13104	1319,62	1,64E+06	1,67E+06	1,73E+06	High	660,31561	49,8348	3,17
GKTIMGAVGE	Q90487	962,50	7,89E+05	1,68E+06	1,34E+06	High	481,75433	36,9453	2,37
QSDFIPSSGELFFQPGVQE	Q6JAN0	2111,99	1,18E+06	1,68E+06	1,47E+06	High	528,75262	28,3049	2,68
KDSSALMQEEIFGPVTCVT	Q66I21	2112,00	1,18E+06	1,68E+06	1,47E+06	High	528,75293	28,2593	3,08
HLHDMDIVYRDMKPENV	Q49HM9	2112,00	1,18E+06	1,68E+06	1,47E+06	High	528,75293	28,6004	2,71
QTPGYPEVPSPQVNPQYNP	Q9DDT5	2112,00	1,18E+06	1,68E+06	1,47E+06	High	528,75293	28,6004	2,7
EEELKTVTNN	P13104	1176,57	7,00E+05	1,69E+06	8,51E+05	High	588,79187	24,8484	1,9
LKKDIDDLELT	Q90339	1302,72	1,18E+06	1,70E+06	1,61E+06	High	651,8623	49,197	2,45
LDVFLLQDTLL	A0A0G2KIZ8	1289,74	1,13E+06	1,70E+06	1,94E+06	High	430,58313	40,3245	2,48
LEQEKKLRMDLE	Q90339	1531,81	1,20E+06	1,70E+06	1,52E+06	High	511,27774	35,3337	3,72
LNRRIQLVEEE	P13104	1398,77	1,41E+06	1,70E+06	2,06E+06	High	466,92941	36,0213	2,66
QGGGATISAKPQII	Q6P0D5	1340,75	7,73E+05	1,70E+06	2,64E+06	High	670,8819	34,4134	2,06
CKILLTEQGI	A4QP16	1174,65	1,43E+06	1,71E+06	1,66E+06	High	587,82874	68,4952	1,91
GQKDSYVGDEAQSKRGILTLK	P83750	2293,21	1,84E+06	1,72E+06	2,67E+06	High	574,05988	40,5529	5,15
RKLEGDLKL	Q90339	1071,65	1,15E+06	1,72E+06	1,61E+06	High	357,89029	28,5324	3,31
KTNGNLGGEAL	Q6NXD8	1073,56	9,47E+05	1,73E+06	1,46E+06	High	537,27869	43,0439	2,4
LEGDLKL	Q90339	787,46	9,87E+05	1,73E+06	1,32E+06	High	394,23297	48,4406	1,99
GWLDKNKDPLNDSVVQ	Q90339	1827,92	1,64E+06	1,73E+06	1,94E+06	High	609,98022	66,9539	3,44
FPSIVGRPRHQGVMVGM	P83750	1867,98	2,02E+06	1,74E+06	1,62E+06	High	623,33203	58,2139	3,7
VGKVIPELNGKLT	Q5MJ86	1367,83	1,17E+06	1,74E+06	9,40E+05	High	456,61453	49,7313	2,71
DLQHRLDEA	Q90339	1096,54	8,77E+05	1,75E+06	1,21E+06	High	548,77454	30,652	2,05

LEQTERGRKVAEQELVDAS	Q90339	2158,11	1,99E+06	1,75E+06	2,26E+06	High	540,28442	40,8031	3,13
IADRMQKEITAL	P53479	1388,76	1,51E+06	1,76E+06	1,29E+06	High	463,59161	52,3568	3,05
AGDDAPRAVFPSIVGRPRHQGVMVGM	P83750	2720,39	6,71E+06	1,77E+06	5,09E+06	High	680,85327	66,7576	5,14
FTIIDQNRDGIISKD	O93409	1734,90	1,40E+06	1,77E+06	1,89E+06	High	867,95703	59,7609	2,97
EAGLAGEKGDRGEMGLPGPPGEKGST	C7DZK3	2497,20	3,05E+06	1,77E+06	3,93E+06	High	500,24905	37,157	3,08
YVGDEAQSKRGIL	P83750	1435,75	1,08E+06	1,78E+06	9,08E+05	High	479,25748	36,4079	3,56
FPVGRVH	Q7ZUY3	811,46	8,86E+05	1,78E+06	1,53E+06	High	406,23416	21,9577	1,96
TALEEAEGTLEHEESKIL	Q90339	1998,99	1,81E+07	1,78E+06	1,10E+07	High	667,00159	96,9134	3,29
ISSDPNYLKFGVNAILASTTRNASPP	Q9PTU0	2733,42	4,56E+06	1,78E+06	2,54E+06	High	456,40683	52,3929	3,16
RLMAGASPTRTHQLLRHRTHNYT	A3KNA7	2733,42	4,56E+06	1,78E+06	2,54E+06	High	456,40683	52,3929	3,16
RMLESLSRLAEAHARLMFRETVT	F1QDI9	2733,43	4,56E+06	1,78E+06	2,54E+06	High	456,40646	51,1773	3,43
KENLANVL	F1QSQ0	900,51	1,05E+06	1,78E+06	5,57E+05	High	450,76257	29,9665	2,47
LPDGQVITIGNE	P83750	1255,65	1,40E+06	1,79E+06	1,37E+06	High	628,33124	73,7832	2,52
GRDLTDYL	P83750	952,47	1,27E+06	1,82E+06	5,18E+05	High	476,74176	65,5379	2,48
GEQIDNLQR	Q90339	1072,54	1,07E+06	1,82E+06	1,26E+06	High	536,77417	27,2984	2,63
NELRVAPEEHPTL	P53479	1504,78	1,21E+06	1,83E+06	5,30E+05	High	502,2645	46,3722	3,53
FAGDDAPRAVFPSIVGRPRHQGVMVGM	P83750	2867,46	1,25E+07	1,84E+06	5,52E+06	High	717,62091	72,9924	2,84
DDLRDVLAT	Q66I73	1017,52	8,55E+05	1,84E+06	1,26E+06	High	509,26532	64,4762	2,26
YVLAGEVAKGSSGPSIVVSFL	Q5PR34	2080,13	4,30E+06	1,84E+06	1,80E+06	High	520,79041	64,4134	2,81
RPDQEVKVSVIGGGDLGIAAV	Q6DBY5	2080,14	4,30E+06	1,84E+06	1,80E+06	High	520,79041	64,4134	2,75
LDAVVVGSGIGGL	Q5BLE8	1156,66	2,63E+06	1,85E+06	1,98E+06	High	578,83356	78,9469	2,31
LEEELKTVTN	P13104	1175,62	6,05E+05	1,87E+06	7,78E+05	High	588,31293	38,3518	2,73
ISERLEEAGGA	Q90339	1131,56	9,15E+05	1,88E+06	1,82E+06	High	566,28705	30,9675	3,06
KIEDEQSLGAQLQ	Q90339	1458,74	1,25E+06	1,88E+06	1,79E+06	High	729,87726	48,3198	3,78
KVELEAKLE	Q6NWF6	1058,61	1,29E+06	1,89E+06	1,31E+06	High	529,8103	33,9707	3,67
SCMERLQAVAQVAEMGNGGGRGG	F1QQA8	2367,06	1,73E+06	1,89E+06	2,10E+06	High	474,2182	22,4088	3,25
TSQEGAARYGNGRIRCSSEHM	E7F4Z4	2367,07	1,73E+06	1,89E+06	2,10E+06	High	474,21863	22,7729	3,74
PSPSPVGYSPMTPGAPSPGGYNPH	Q9DDT5	2367,07	1,73E+06	1,89E+06	2,10E+06	High	474,21826	22,4938	3,38
RGYSFVTT	P53479	930,47	1,31E+06	1,90E+06	1,60E+06	High	465,73911	41,088	2,15
QLGETGKSIHE	Q90339	1198,61	1,02E+06	1,90E+06	1,26E+06	High	400,20886	19,1344	3,12
LDLAGRDLTDYLMK	P83750	1623,84	5,89E+06	1,91E+06	3,74E+06	High	812,42657	89,8017	3,96
KTVDGPSGKLWRDGRGASQ	Q5XJ10	2015,04	1,82E+06	1,91E+06	1,42E+06	High	504,51791	25,9122	5,52
EQLGETGKSIHEL	Q90339	1440,73	1,26E+06	1,91E+06	1,41E+06	High	480,91693	37,9525	3,2

RVPTPNVSVVD	Q5XJ10	1182,65	1,61E+06	1,92E+06	4,16E+05	High	591,82965	47,7556	2,3
DLSRELEE	Q90339	990,47	1,20E+06	1,92E+06	9,08E+05	High	495,74261	44,4855	2,46
TDAIQRTEELE	Q90339	1304,63	1,03E+06	1,92E+06	1,39E+06	High	652,82141	43,4762	3,58
ADIRNDIAE	A8WGB1	1016,50	1,12E+06	1,92E+06	1,80E+06	High	508,75577	22,8065	1,94
LWRDGRGASQNIIPA	Q5XJ10	1653,88	1,80E+06	1,93E+06	2,54E+06	High	551,96686	51,9168	2,5
ALTDAETKAFLKA	P09227	1378,76	1,32E+06	1,93E+06	1,55E+06	High	460,25891	56,3637	3,08
IEEIKRL	Q6DG88	900,55	9,84E+05	1,93E+06	2,16E+06	High	450,78049	28,7794	2,24
YASGRTTGIVL	P53479	1137,63	1,26E+06	1,94E+06	1,70E+06	High	569,3186	48,3216	2,52
LELQQGAAL	E7F3F0	942,53	9,34E+05	1,94E+06	1,11E+06	High	471,76813	26,7889	1,96
GLNSADMLK	Q90339	948,48	8,44E+05	1,95E+06	1,71E+06	High	474,74606	38,6417	2,28
TERGYSFVTTA	P53479	1231,60	1,17E+06	1,96E+06	1,55E+06	High	616,30304	46,5802	2,38
PDLTGEQRVMILVGGLEYSPG	B0S5N4	2247,13	3,58E+06	1,96E+06	2,66E+06	High	562,53821	57,6544	3,24
VHIMGGGSVAIMTKLMELLSQ	A2BGG1	2247,15	3,58E+06	1,96E+06	2,66E+06	High	562,5387	57,7504	3,17
SLQMSLLLCSFVAVAGGAFFL	Q7ZU13	2247,16	3,58E+06	1,96E+06	2,66E+06	High	562,5387	57,7504	3,28
TTNYLTIGKMALMGTPFLAGF	Q9MIY0	2247,16	3,58E+06	1,96E+06	2,66E+06	High	562,5387	57,7504	3,18
SYVGDEAQSKRGILTL	P83750	1736,92	1,98E+06	1,96E+06	3,02E+06	High	579,64606	60,5543	3,64
KSTEPKPTPQ	Q2LE08	1112,59	1,60E+06	1,96E+06	1,60E+06	High	556,80286	42,5938	2,06
IEDEQSLGAQL	Q90339	1202,59	1,44E+06	1,97E+06	1,32E+06	High	601,80035	64,9872	2,19
AGDDAPRAVFPSIVGRPR	P83750	1881,01	1,90E+06	1,97E+06	2,31E+06	High	471,00876	53,2907	5,13
LEQQVDDLEGSLEQE	Q90339	1731,79	4,72E+06	1,97E+06	3,21E+06	High	866,40137	79,607	2,32
KSPSMPVASPGWTASPKAAMP	Q67FY3	2098,05	1,25E+06	1,98E+06	9,87E+05	High	700,01697	26,7965	2,33
KQEYDEAGPSIVH	P53479	1472,70	1,07E+06	1,98E+06	1,40E+06	High	491,57346	33,6667	4,23
TNEKLQQF	Q90339	1007,52	1,03E+06	1,99E+06	1,34E+06	High	504,263	30,4481	2,7
NWDDMEKIWHH	P83750	1526,65	1,30E+06	1,99E+06	1,64E+06	High	509,55521	70,36	2,4
GEQIDNLQRVKQKLEKEKSE	Q90339	2399,29	2,76E+06	1,99E+06	3,26E+06	High	600,57892	40,4002	4,08
KDDLRDVLAT	Q66I73	1145,62	8,86E+05	1,99E+06	1,27E+06	High	573,31311	46,3773	3,65
IPELNGKLT	Q5MJ86	984,57	1,26E+06	2,00E+06	7,12E+05	High	492,7916	48,5653	2,28
ERRMEISGVDAPAVL	A1IH00	1642,86	1,13E+06	2,01E+06	1,50E+06	High	548,29138	36,0536	2,35
TMYPGIADRM	P83750	1154,53	1,05E+06	2,01E+06	1,92E+06	High	577,77179	57,6552	2,43
FAGDDAPRAVFPSIVGRPR	P83750	2028,08	3,41E+06	2,01E+06	2,74E+06	High	676,69873	62,8335	4,51
PSGPAALGFAPGKPPPSMPPNQAPIAAQMP	Q08C81	2926,46	2,19E+06	2,02E+06	2,44E+06	High	732,37225	30,3454	3,42
GGSGTTVSHNNLI	Q6DC04	1256,62	1,65E+06	2,02E+06	3,71E+06	High	628,81763	78,2219	1,99
SGGTTMYPGIADRM	P83750	1472,65	1,06E+06	2,02E+06	1,27E+06	High	736,83038	50,9187	3,22

LDLAGRDLTDY	P83750	1251,62	1,98E+06	2,05E+06	1,68E+06	High	626,31537	73,4305	3,6
GEKLKGADPEDVIVSA	O93409	1627,85	3,12E+05	2,05E+06	4,38E+05	High	543,29156	52,4282	2,9
KDSYVGDEAQSKRGILTL	P83750	1980,04	2,08E+06	2,06E+06	4,59E+06	High	660,68622	52,0123	8,15
FPSIVGRPR	P83750	1028,60	1,87E+06	2,06E+06	1,96E+06	High	343,53909	36,8386	2,57
DLQHGSLF	Q9PVK5	916,45	1,27E+06	2,07E+06	1,43E+06	High	458,7312	70,2526	1,95
TSDGTPLVGVNIS	Q9W7R3	1259,65	1,06E+06	2,08E+06	1,83E+06	High	630,32422	27,5598	2,04
KQEYDEAGPSIVHR	P53479	1628,80	1,12E+06	2,08E+06	1,51E+06	High	543,60736	28,3341	3,87
DHALNDMTSI	P13104	1116,50	1,30E+06	2,10E+06	1,06E+06	High	558,75476	57,0923	2,52
LEEAEGTLEHEESKIL	Q90339	1826,90	3,13E+06	2,10E+06	3,23E+06	High	609,63971	65,0653	2,37
AANLDKKQRNFDKV	Q90339	1646,90	1,51E+06	2,10E+06	8,64E+05	High	549,6391	16,926	3,25
IIDQNRDGIISK	O93409	1371,76	1,07E+06	2,11E+06	9,91E+05	High	457,92603	30,0063	3,32
GYPREVKQGEEFE	P12115	1567,74	1,82E+06	2,12E+06	1,73E+06	High	523,25177	34,428	2,78
APRAVFPS	P83750	844,47	1,40E+06	2,14E+06	2,79E+06	High	422,73837	33,5415	1,95
RKDGTLAPLGQAI	Q6DRJ9	1339,77	1,05E+06	2,14E+06	1,44E+06	High	447,26288	30,7397	2,35
VTTAEREIVRDIKE	P53479	1658,91	1,89E+06	2,15E+06	2,41E+06	High	553,64246	39,2749	3,37
NDEELNKLL	Q7ZUY3	1087,56	1,21E+06	2,17E+06	1,34E+06	High	544,28674	63,2725	2,78
LEGTLLKP	Q8JH70	870,53	1,21E+06	2,18E+06	7,96E+05	High	435,76923	45,0312	2,21
VEKQRADLSRELEEISE	Q90339	2031,04	4,02E+06	2,18E+06	4,28E+06	High	677,68494	55,7321	3,38
KNAIKNQK	F1Q4S1	943,57	7,09E+05	2,19E+06	2,39E+06	High	472,28928	21,3654	1,97
ILNHGYEAHDKHREAEPAAGSHALSR	Q7ZWK8	2866,41	2,15E+06	2,20E+06	2,51E+06	High	717,35468	34,6481	3,04
LRVAPEEHPTLL	P53479	1374,77	1,67E+06	2,21E+06	2,47E+06	High	458,93112	53,491	3,29
ELRVAPEEHPTL	P53479	1390,73	1,41E+06	2,22E+06	7,45E+05	High	464,25101	47,3303	3,56
AFRVPTPNVSVVDL	Q5XJ10	1513,84	6,61E+06	2,22E+06	5,21E+06	High	757,42322	94,745	3,58
LQAGADISMIGQFGVGFYSAYLVA	O57521	2494,23	2,28E+06	2,22E+06	2,66E+06	High	499,65305	33,1026	3,68
GGSKPRDVTNFTVGGFAPMSPRIS	Q9DDT5	2494,25	2,28E+06	2,22E+06	2,66E+06	High	499,6524	33,3171	3,68
NLNDRLATY	Q90303	1079,55	1,13E+06	2,22E+06	2,46E+06	High	540,27869	44,7201	2,88
FLPATPSTGAVVNHPVLLAACESTPSP	B3DIY3	2733,39	5,57E+06	2,23E+06	3,11E+06	High	547,48724	51,1368	2,85
NTAKTQSTAQSVRSAVALTSAEPTNVT	Q7T019	2733,40	5,57E+06	2,23E+06	3,11E+06	High	547,48694	51,0512	3,09
QALMQAQKRASRGQIYLNWVNKN	Q9I9H8	2733,44	5,57E+06	2,23E+06	3,11E+06	High	547,48785	51,0862	2,82
RRDLEESTLQHEAT	Q90339	1684,82	1,30E+06	2,23E+06	2,37E+06	High	562,28204	26,2072	3,46
LDKENALDRA	P13104	1144,60	1,05E+06	2,23E+06	2,38E+06	High	572,80267	22,4145	2,64
MLAHISIFLFLMGNAL	X1WBB5	1806,96	4,63E+06	2,23E+06	4,61E+06	High	602,99487	54,3392	2,59
KLMVAADIVTMFNLIQ	A0A140LFM6	1806,99	4,63E+06	2,23E+06	4,61E+06	High	602,99487	54,3392	2,47

LEQYSTKMEATECGTGLIGRGDS	Q1LYE3	2503,14	2,23E+06	2,24E+06	1,86E+06	High	835,06116	51,9391	2,53
TVDGPSGKLWRDGRGA	Q5XJ10	1671,86	1,53E+06	2,25E+06	1,63E+06	High	418,7207	32,6731	3,55
DESIAKLT	Q90339	876,47	9,17E+05	2,25E+06	1,43E+06	High	438,73846	39,9021	2,16
PGIADRM	P83750	759,38	9,53E+05	2,25E+06	5,74E+05	High	380,19562	28,264	2,09
GRPRHQGVMVGM	P83750	1324,67	1,61E+06	2,26E+06	2,71E+06	High	442,23056	19,9564	2,96
TEAPLNPKANREKMTQ	P83750	1827,94	1,25E+06	2,27E+06	2,15E+06	High	457,74112	21,7236	4,38
LAGRDLTDYL	P83750	1136,59	2,70E+06	2,31E+06	7,69E+05	High	568,80219	76,7284	2,6
AVFPSIVGRPRHQ	P83750	1463,82	1,88E+06	2,31E+06	3,23E+06	High	366,71252	36,8691	2,89
EKTIDDLEDELYAQ	P13104	1681,78	8,19E+06	2,32E+06	7,36E+06	High	841,3952	84,3133	3,45
LEQQVDDL	Q90339	959,47	1,53E+06	2,33E+06	1,49E+06	High	480,23883	49,3979	2,13
RVPTPNVSVV	Q5XJ10	1067,62	1,30E+06	2,33E+06	1,62E+06	High	534,31506	48,8167	2,21
LNDHFVKL	Q5XJ10	985,55	1,28E+06	2,33E+06	2,44E+06	High	493,27853	42,6003	2,78
DSYVGDEAQSKRGILT	P83750	1738,86	1,48E+06	2,34E+06	1,78E+06	High	580,29333	46,0905	4
FRENLGKL	Q90339	976,56	1,29E+06	2,35E+06	1,36E+06	High	488,78381	34,5112	1,93
GVFTTIEKA	Q5XJ10	965,53	1,68E+06	2,35E+06	1,41E+06	High	483,27036	50,7005	2,26
DASERVGLL	Q90339	959,52	1,30E+06	2,35E+06	1,61E+06	High	480,26208	53,4322	2,43
DESERGMKVIENR	P13104	1562,76	1,12E+06	2,36E+06	1,30E+06	High	521,5929	28,903	3,68
LTMFGEKLKGADPEDVIVS	O93409	2049,06	8,82E+06	2,36E+06	6,28E+06	High	683,69202	83,4915	2,79
KEFLEELLTTQ	O93409	1350,72	4,82E+06	2,39E+06	4,62E+06	High	675,86304	96,9073	2,92
AEEDKVNTL	Q90339	1018,51	1,35E+06	2,40E+06	1,30E+06	High	509,75723	26,3255	2,03
FAGDDAPRAVFPSIV	P83750	1561,80	3,36E+06	2,40E+06	3,47E+06	High	781,40613	89,8733	3,52
TNSITSPNRIGILGGVT	Q1JPZ3	1699,93	1,56E+06	2,41E+06	2,83E+06	High	567,32104	24,4157	2,44
KELLEGGELVSVLKGE	Q5XJ54	1699,95	1,56E+06	2,41E+06	2,83E+06	High	567,32153	24,7906	2,38
RYDILPLSPISRNR	Q5U395	1699,96	1,56E+06	2,41E+06	2,83E+06	High	567,32153	24,7906	2,46
AFPPDVAGNVDYK	O93409	1392,68	1,24E+06	2,41E+06	1,91E+06	High	696,84558	59,8803	2,13
ENRRLYRQL	Q8JH70	1247,70	1,50E+06	2,42E+06	3,02E+06	High	416,57159	19,8727	2,35
RLEDEEEINAE	Q90339	1346,61	1,37E+06	2,42E+06	2,70E+06	High	673,80859	35,8007	3,5
DTGAPIRIPVGPETL	Q9PTY0	1535,84	2,56E+06	2,43E+06	2,27E+06	High	768,42645	90,8271	3,83
NALDRAEQA	P13104	987,49	1,14E+06	2,43E+06	1,49E+06	High	494,24817	24,9807	2,87
SYELPDGQVITIG	P83750	1391,71	5,67E+06	2,47E+06	3,46E+06	High	696,35791	105,7803	2,15
LDHALNDMT	P13104	1029,47	1,07E+06	2,47E+06	1,88E+06	High	515,23871	36,8148	2,6
IKESDII	F1QBY1	817,47	1,43E+06	2,48E+06	1,02E+06	High	409,23819	46,0118	2,09
KEISDLI	A0A0R4IBL7	817,47	1,43E+06	2,48E+06	1,02E+06	High	409,23819	46,0118	1,99

YDNEFGYSNR	Q5XJ10	1264,52	1,38E+06	2,49E+06	9,02E+05	High	632,76685	38,5399	2,28
LDDGFPLPE	P59889	1002,48	1,52E+06	2,49E+06	1,50E+06	High	501,74728	19,7861	1,99
LQDLVDKL	Q90339	943,55	1,93E+06	2,49E+06	2,06E+06	High	472,27774	61,8864	2,68
EQLGETGKSIHELE	Q90339	1569,78	2,02E+06	2,50E+06	2,17E+06	High	523,93115	39,9391	3,33
KNLEVTV	Q90339	802,47	1,45E+06	2,51E+06	8,05E+05	High	401,73865	33,7556	2,43
LAGMAALNGGLGSTGLSNGSAGPMDALTQAY	Q6P0B1-3	2866,37	2,55E+06	2,51E+06	2,67E+06	High	478,57233	34,3378	4,28
KQQQEGKHFSENYMEALIEKWIN	Q6NYU2	2866,38	2,55E+06	2,51E+06	2,67E+06	High	478,57297	34,7432	4,1
KPECYLIHGGRTPNNELSSSLYMLS	O13034	2866,39	2,55E+06	2,51E+06	2,67E+06	High	478,5737	34,4326	4,64
PNTLNMIEQNLDHHIDKECRYLL	A0A0R4I9Y1	2866,40	2,55E+06	2,51E+06	2,67E+06	High	478,57233	34,3378	4,49
RSVTGGMCSVFLQDTEKVVSISSEHL	Q9DDT5	2866,41	2,55E+06	2,51E+06	2,67E+06	High	478,57233	34,3378	4,42
IIAPPERKYS	P83750	1173,66	1,50E+06	2,53E+06	9,85E+05	High	391,89371	26,8432	2,46
AGRDLTDYL	P83750	1023,51	#DIV/0!	2,53E+06	#DIV/0!	High	512,26093	66,9287	2,44
DIDIRKDLYA	P53479	1221,65	2,20E+06	2,53E+06	1,19E+06	High	611,32892	62,1463	2,88
LGEQIDNLQRVKQKLEKEKSEYKME	Q90339	3063,61	9,78E+06	2,57E+06	8,45E+06	High	613,53009	62,5178	5,78
RRIQLVEEE	P13104	1171,64	2,92E+06	2,57E+06	2,09E+06	High	391,22028	27,8384	3,22
TKAKTKLEQQVDDL	Q90339	1616,89	1,87E+06	2,58E+06	2,88E+06	High	539,63507	33,7013	3,69
ELKLFLQ	P05939	890,53	3,60E+06	2,59E+06	2,90E+06	High	445,77234	75,3858	2,1
RLDLAGRDLTDYLMK	P83750	1779,94	7,72E+06	2,61E+06	5,66E+06	High	593,98755	79,1366	3,44
KEPIRPSMASPILFLTAP	Q9MIZ0	1968,10	2,17E+06	2,62E+06	2,76E+06	High	492,78503	24,3806	2,62
RVAPEEHPTLLT	P53479	1362,74	1,49E+06	2,62E+06	3,61E+06	High	454,91846	37,0203	2,79
LEQTERGRKVAEQELVDA	Q90339	2071,08	3,22E+06	2,62E+06	3,43E+06	High	518,52649	39,2452	3,32
QGALKEGQEQLDASI	I6V1W0	1586,80	1,55E+06	2,63E+06	2,69E+06	High	529,61066	41,8795	2,4
FLRESGPSVHILLIQMD	A0A0R4I9Y1	1971,04	2,27E+06	2,63E+06	1,98E+06	High	493,51038	41,7244	2,73
AEREIVRDIKEKL	P83750	1598,92	2,78E+06	2,64E+06	2,86E+06	High	400,48715	42,0166	3,63
AVRNDEELNKLL	Q7ZUY3	1413,77	2,29E+06	2,64E+06	1,72E+06	High	471,92911	52,7953	3,02
FWGLMVGLVVGMVRMIME	A8WHP3	2116,05	3,30E+06	2,65E+06	3,93E+06	High	529,76306	38,6774	2,68
QDTTPIGMLSPSLSSPPLTSS	F6NSX9	2116,05	3,30E+06	2,65E+06	3,93E+06	High	529,76245	38,6194	2,67
QQYTEALMALTAAPSLRDH	B8JKP6	2116,05	3,30E+06	2,65E+06	3,93E+06	High	529,76306	38,6774	2,62
SGAGGLENGSLSAQGTGSTPSPFPFHL	Q7ZTU9	2573,23	1,75E+06	2,65E+06	2,52E+06	High	858,41156	39,3841	2,32
GESGAGKTVNTKRVIQY	Q90339	1807,97	1,86E+06	2,66E+06	2,03E+06	High	603,32867	27,0231	4,96
TDAIQRTEEL	Q90339	1175,59	1,32E+06	2,67E+06	1,44E+06	High	588,29968	41,3373	3,36
MGVGLVKGGVSAVAGGVTAVGSA	Q7T352	1960,05	3,24E+06	2,67E+06	2,96E+06	High	392,81903	31,0922	3,24
EAFAGIVSGALMLGVGAIVQG	A2RUW7	1960,06	3,24E+06	2,67E+06	2,96E+06	High	392,81903	31,0922	3,28

EQRSRFLAVGLVDNTVR	Q1LVE8	1960,07	3,24E+06	2,67E+06	2,96E+06	High	392,81903	31,0922	3,37
MKKTVPQTISKSLEPVTG	Q1LUT1	1960,08	3,24E+06	2,67E+06	2,96E+06	High	392,81906	31,1155	3,06
YNELRVAPEEHPTLL	P53479	1780,92	2,84E+06	2,68E+06	2,29E+06	High	594,31378	64,9341	4,03
EEAEGTLEHEE	Q90339	1272,52	1,38E+06	2,68E+06	1,60E+06	High	636,76666	26,4578	2,89
INMASVASSIK	Q561X9	1120,60	1,47E+06	2,69E+06	1,76E+06	High	560,80988	33,8827	2,02
EKTIDDLE	P13104	962,47	1,36E+06	2,71E+06	1,03E+06	High	481,73904	33,4522	2,35
GPAGVGVGVGMGLIGPGGIPSFYSQPA	O42477	2458,24	1,74E+06	2,71E+06	2,83E+06	High	820,0799	32,5858	2,88
NRSWQKEVQEL	Q7T019	1416,72	2,38E+06	2,71E+06	2,14E+06	High	472,91415	36,5547	2,38
DIDIRKDL	P53479	987,55	1,49E+06	2,72E+06	1,39E+06	High	494,2793	53,2196	2,35
LDDVKREL	Q7ZU99	987,55	1,49E+06	2,72E+06	1,39E+06	High	494,27924	53,4158	2,12
DSYVGDEAQSKRGIL	P83750	1637,81	2,21E+06	2,72E+06	2,07E+06	High	546,61108	47,4521	3,93
AAQMKGLSQEAEEL	P85001	1520,73	1,59E+06	2,72E+06	1,86E+06	High	507,58167	32,2389	2,97
SQMVFSQVPGAQEI	Q1L8G6	1520,74	1,59E+06	2,72E+06	1,86E+06	High	507,58221	31,881	2,43
KNETSTSRQFVHS	Q6P4P2	1520,75	1,59E+06	2,72E+06	1,86E+06	High	507,58191	32,3471	2,8
MYPGIADR	P83750	922,45	1,52E+06	2,74E+06	2,67E+06	High	461,72733	35,9575	1,99
ELEEEIEAERAARAK	Q90339	1743,89	1,89E+06	2,74E+06	6,05E+06	High	436,72919	40,42	2,97
LVAASTGIRSLPCVI	Q5RHH4	1556,88	1,43E+06	2,76E+06	2,53E+06	High	519,63483	25,1237	2,7
RLDLAGRDLTDY	P83750	1407,72	2,56E+06	2,77E+06	2,60E+06	High	469,9136	61,6919	3,28
KGMDPLVLIDAIAIC	A0A0R4ITC5	1644,87	1,82E+06	2,78E+06	2,13E+06	High	548,95923	43,3473	2,45
ITSPMFRNVPTAIPT	A1XQX8	1644,88	1,82E+06	2,78E+06	2,13E+06	High	548,95941	44,0254	2,31
DVASLLNRQVP	Q7ZYZ6	1211,67	1,61E+06	2,80E+06	1,70E+06	High	606,3421	55,6037	2,47
EEELKLFLQ	P05939	1148,62	4,28E+06	2,80E+06	2,84E+06	High	574,81531	82,7343	2,53
ANREKMTQIMFETF	P83750	1745,83	6,76E+06	2,80E+06	4,11E+06	High	582,61823	76,0491	4,77
RTEGLGRFLQTLQSKP	F1R345	1831,02	1,83E+06	2,81E+06	7,94E+06	High	458,51215	28,006	3,64
KITITNDKGRLSKEDI	Q90473	1831,03	1,83E+06	2,81E+06	7,94E+06	High	458,5116	27,9438	3,51
TDAETKAFLK	P09227	1123,60	1,23E+06	2,81E+06	1,76E+06	High	562,30469	27,7826	3,65
KKLRMDLE	Q90339	1032,59	5,70E+05	2,81E+06	1,61E+06	High	516,79926	19,3049	1,97
LQNFKAGARAL	P05939	1188,68	1,68E+06	2,82E+06	2,14E+06	High	396,90088	32,4582	2,89
YETDAIQR	Q90339	995,48	1,11E+06	2,83E+06	1,72E+06	High	498,2449	24,7448	2,38
DDAPRAVFPSIVGRPRHQ	P83750	2018,07	1,76E+06	2,83E+06	3,01E+06	High	505,27405	46,2817	3,26
GTTMYPGIADRM	P83750	1312,60	1,88E+06	2,83E+06	2,69E+06	High	656,8064	60,9567	2,36
KTKQRLQGEVEDL	Q90339	1543,84	2,59E+06	2,84E+06	2,25E+06	High	515,2876	26,8476	3,61
DLENEKQQSDE	Q90339	1334,57	1,79E+06	2,85E+06	1,83E+06	High	667,79077	18,606	2,21

YPQTKTY	P82315	900,45	1,96E+06	2,85E+06	2,76E+06	High	450,72839	20,6434	2,28
AGAGAGLNMSSLASMAALNGGLGSGG	Q9IBD0	2208,04	2,52E+06	2,86E+06	2,49E+06	High	736,68744	37,3481	2,5
DLTDYLMK	P83750	998,49	1,47E+06	2,87E+06	2,45E+06	High	499,74805	80,6067	1,94
LDKKQRNFDKVL	Q90339	1503,86	1,72E+06	2,87E+06	2,73E+06	High	501,96082	24,0259	3,06
MQKEITAL	P53479	933,51	1,42E+06	2,87E+06	2,21E+06	High	467,25851	38,4104	2,62
QQVDDLEGSLE	Q90339	1232,56	1,83E+06	2,87E+06	2,03E+06	High	616,78711	62,9773	2,52
LKGADPEDVIVS	O93409	1242,66	1,81E+06	2,88E+06	2,01E+06	High	621,83398	52,0404	2,52
RRDLEESTLQ	Q90339	1246,64	1,76E+06	2,89E+06	2,36E+06	High	416,21887	23,6649	2,85
TTRARIDVPVSSVIPVSLG	A0MSJ1	1967,13	4,94E+06	2,89E+06	4,23E+06	High	394,23532	32,4045	2,75
SNVKALGKSLEKQLAARGP	Q7T019	1967,14	4,94E+06	2,89E+06	4,23E+06	High	394,23505	32,028	2,71
GKTVTIVEAIKQVEKNIP	Q1LXK5	1967,15	4,94E+06	2,89E+06	4,23E+06	High	394,23505	32,028	2,75
RENLGKLM	Q90339	960,53	1,57E+06	2,90E+06	3,65E+06	High	480,76971	28,338	2,27
QNKQSII	A0A8M3B525	830,47	1,65E+06	2,90E+06	1,84E+06	High	415,74158	37,3436	2
EEKEKQEE	A0A0R4IC37	1048,48	1,79E+06	2,91E+06	2,09E+06	High	524,74194	24,0047	2,07
WYDNEFGYSNR	Q5XJ10	1450,60	2,63E+06	2,91E+06	2,24E+06	High	725,8064	60,6067	2,87
TNEKLQQFFNH	Q90339	1405,69	1,81E+06	2,92E+06	2,22E+06	High	469,23505	50,4618	2,85
AGFAGDDAPRAVFPS	P83750	1477,71	1,69E+06	2,92E+06	2,51E+06	High	739,35931	67,8496	4,08
DIRKDLYAN	P83750	1107,58	1,49E+06	2,92E+06	2,77E+06	High	554,29419	59,0084	2,64
SVIPEGQFIDNK	Q90339	1346,70	1,59E+06	2,95E+06	2,01E+06	High	673,8526	60,2137	2,85
EKSYELPDGQVITIGNE	P83750	1891,93	1,15E+07	2,96E+06	6,89E+06	High	946,47406	89,3466	3,06
GALQGGVCFSLRKME	F1QXM5	1652,82	1,69E+06	2,98E+06	2,91E+06	High	551,61273	24,4703	2,39
QLGETGKSIHEL	Q90339	1311,69	1,86E+06	3,02E+06	2,56E+06	High	437,90305	33,1604	3,15
SPTTSPREAETHGPDAGKSPAGSPSSSSGSLS	Q1L8W0	3026,39	2,39E+06	3,02E+06	2,79E+06	High	757,3537	28,882	3,01
RPGMAVVSGGTVLNATSP	B0R0I6	1729,89	3,11E+06	3,02E+06	2,58E+06	High	433,22513	30,9285	3,31
KETDIIY	F5HSE3	881,46	1,64E+06	3,03E+06	2,22E+06	High	441,23547	34,8849	1,95
TVAGEAKNMRAYLGGLGSREEL	F1QQA8	2338,18	2,20E+06	3,04E+06	3,03E+06	High	585,30182	38,0432	3,93
VSSAALTASSQKQTMVISAGGSSVA	Q7ZUV7	2338,19	2,20E+06	3,04E+06	3,03E+06	High	585,30212	37,9396	4
RHGQAFSPIYRFSLSDGTIVS	Q98TW1	2338,19	2,20E+06	3,04E+06	3,03E+06	High	585,30194	38,2669	4,15
IDSAPGLGDYLML	A1XQX8	1380,67	1,42E+06	3,04E+06	2,07E+06	High	690,84344	19,2312	2,07
DVDIRKDLYAN	P83750	1321,67	1,94E+06	3,06E+06	2,39E+06	High	661,34259	51,8706	3,25
WADLSPGSGPVKKHGKTIM	Q90487	2009,06	3,53E+06	3,07E+06	5,35E+06	High	503,02252	34,3638	4,38
RDLEESTL	Q90339	962,48	2,00E+06	3,08E+06	1,49E+06	High	481,74402	34,5206	2,52
MVLAGALESVRVLKSD	Q9YHT4	1703,94	2,33E+06	3,08E+06	2,83E+06	High	426,74301	26,8727	2,9

TTRARVTTPTRSLFP	C7DZK3	1703,96	2,33E+06	3,08E+06	2,83E+06	High	426,74277	27,2657	3,39
KSYELPDGQVITI	P83750	1462,78	7,46E+06	3,09E+06	9,22E+06	High	731,89459	94,4892	3,6
NSQRVIDSM	Q90339	1049,50	2,19E+06	3,11E+06	1,89E+06	High	525,25751	30,3599	3,29
AGDDAPRAVFPSIV	P83750	1414,73	2,45E+06	3,11E+06	3,39E+06	High	707,87189	80,5399	3,12
MLSTRFSGNFLVNLL	Q90ZM2	1711,92	3,09E+06	3,11E+06	2,38E+06	High	571,31354	65,7106	2,32
NSTVRKQAMHSLQVL	A0A0R4ITC5	1711,93	3,09E+06	3,11E+06	2,38E+06	High	571,31354	65,7106	2,34
FRENLGKLM	Q90339	1107,60	2,02E+06	3,16E+06	3,14E+06	High	554,30457	46,0343	2,59
SGGTTMYPGIADRMQKEITAL	P53479	2240,10	1,93E+07	3,18E+06	9,36E+06	High	747,37427	90,6446	3,6
NDNSSRFGKFIRIH	Q90339	1690,88	3,06E+06	3,18E+06	3,23E+06	High	564,29858	41,175	3,85
SSRAGLQFPVGRVH	Q7ZUY3	1510,82	3,24E+05	3,20E+06	6,49E+05	High	504,28076	39,2533	3,22
VVDLTVR	Q5XJ10	801,48	2,15E+06	3,26E+06	2,01E+06	High	401,24606	35,4131	2,15
EQIDNLQRVK	Q90339	1242,68	1,85E+06	3,27E+06	2,00E+06	High	414,89948	26,9731	3,15
SLNRRIQLVEEE	P13104	1485,80	2,57E+06	3,30E+06	5,67E+06	High	495,94092	40,5843	2,42
ELRVAPEEHPTLL	P53479	1503,82	3,21E+06	3,32E+06	7,27E+06	High	501,9451	58,8456	4,16
DAVAKFMLDLHINGIQ	A0A0R4IBK5	1800,93	4,13E+06	3,34E+06	3,28E+06	High	600,9848	56,1174	2,37
LEGMEYRPIPALNANL	Q7ZVM9	1800,93	4,13E+06	3,34E+06	3,28E+06	High	600,9848	56,2326	2,79
VTSLEEQIVHLRGDMDRQ	Q90303	2126,07	7,08E+06	3,34E+06	6,69E+06	High	709,35974	72,9268	2,96
AFTIIDQNRDGIIS	O93409	1562,82	3,35E+06	3,34E+06	4,38E+06	High	781,91412	77,2321	3,47
KLRMDLE	Q90339	904,49	2,32E+06	3,35E+06	2,23E+06	High	452,7515	29,3051	2,13
MDAIKKK	P13104	875,50	2,95E+05	3,36E+06	1,23E+06	High	438,25574	19,3669	2,39
TKLEQQVDDLE	Q90339	1317,65	2,32E+06	3,36E+06	3,02E+06	High	659,3316	47,8337	2,91
IEMNKKREAEFQ	Q90339	1522,77	2,36E+06	3,38E+06	2,98E+06	High	508,26328	21,2688	3,37
IVGRPRHQGVM	P83750	1249,69	1,18E+06	3,38E+06	3,29E+06	High	417,2377	15,2357	3,41
QSRRSVVCSVRGREFRPQ	Q08BB3	2204,16	2,77E+06	3,38E+06	2,44E+06	High	551,78943	34,7	2,78
LDKENALDRAEQAE	P13104	1601,78	2,31E+06	3,38E+06	4,91E+06	High	534,59857	36,4156	4,19
FAGDDAPRAVF	P83750	1165,56	2,46E+06	3,39E+06	3,54E+05	High	583,2865	58,7934	3,53
GKVIPELNGKLT	Q5MJ86	1268,76	2,11E+06	3,40E+06	3,45E+06	High	423,59161	44,905	2,86
SVDLPSSALTIMNTALP	Q8JIS6	1745,90	2,27E+06	3,42E+06	2,77E+06	High	582,64185	49,9729	2,55
APLNPKANREKMTQIM	P83750	1841,97	3,48E+06	3,42E+06	3,73E+06	High	614,66388	37,4268	2,8
IIAPPERKYSVW	P83750	1458,81	4,12E+06	3,42E+06	4,16E+06	High	486,94275	57,0049	2,99
RMKKNLEVTV	Q90339	1217,70	2,62E+06	3,43E+06	2,81E+06	High	406,57397	21,2129	4,23
RVQLELN	Q90339	871,50	2,28E+06	3,43E+06	4,18E+06	High	436,25461	36,8517	2,42
KQRADLSREL	Q90339	1215,68	2,24E+06	3,46E+06	3,00E+06	High	405,90015	19,068	3

SYELPDGQVITIGNE	P83750	1634,79	9,63E+06	3,46E+06	5,49E+06	High	817,90137	103,0257	2,95
KSNTSTPVRDKEEEPKPTVATPEPEV	Q5RGJ8	2865,45	6,58E+06	3,46E+06	6,64E+06	High	717,12	53,5804	2,7
TQYVIAEASTPVTAAVNSSQVKTTHYV	A2BID7	2865,46	6,58E+06	3,46E+06	6,64E+06	High	717,12	53,5804	2,62
TALEEAEGTLEHEE	Q90339	1557,69	3,47E+06	3,46E+06	4,01E+06	High	779,35004	69,2717	3,53
KKDIDDLELTLA	Q90339	1373,75	4,22E+06	3,52E+06	9,03E+06	High	458,59024	64,9581	4,35
LKEADIT	P05939	789,44	1,91E+06	3,52E+06	2,48E+06	High	395,22278	23,2379	2,02
LSQDLLT	Q58G59	789,44	1,91E+06	3,52E+06	2,48E+06	High	395,22278	23,2379	2,06
SKIEDEQSLGAQLQ	Q90339	1545,78	1,90E+06	3,52E+06	3,81E+06	High	773,39337	48,6579	3,74
DIDIRKDLY	P53479	1150,61	2,58E+06	3,57E+06	2,06E+06	High	575,81042	60,1659	2,92
DDLRDVL	O93409	845,44	2,09E+06	3,60E+06	1,17E+06	High	423,22256	62,0046	1,99
NELRVAPEEHPTLL	P53479	1617,86	2,96E+06	3,65E+06	4,69E+06	High	539,96021	58,0045	5,38
LGETGKSIHEL	Q90339	1183,63	1,80E+06	3,66E+06	2,24E+06	High	395,21667	30,6912	2,96
ALPHAIMRL	P53479	1021,60	2,83E+06	3,67E+06	3,90E+06	High	511,30402	51,3928	2,18
GESGAGKTVNTKRVIQYFAT	Q90339	2127,12	4,27E+06	3,67E+06	4,91E+06	High	532,53693	51,0485	3,26
GEQIDNLQRVKQKLEKE	Q90339	2055,12	3,55E+06	3,68E+06	4,22E+06	High	514,53711	44,4086	4,38
VTGQMNSTIAAKLTAVEGALKE	Q1LUT1	2232,19	4,30E+06	3,69E+06	3,99E+06	High	558,80652	39,3597	3,19
KEAFTIIDQNRDGIIS	O93409	1819,95	3,91E+06	3,69E+06	3,30E+06	High	910,48303	66,4624	4,77
NVPAMYVAIQ	P53479	1105,57	3,66E+06	3,72E+06	4,16E+06	High	553,29028	78,6548	2,26
RVIISAPSADAPMF	Q5XJ10	1474,77	6,53E+06	3,76E+06	1,15E+06	High	737,89142	78,8533	3,13
ELEEELK	P13104	889,45	2,16E+06	3,76E+06	2,09E+06	High	445,23053	30,222	2,02
PSGPNPAAQPAREQQQAAS	Q06725	1904,92	2,49E+06	3,77E+06	2,68E+06	High	635,65149	46,7398	2,34
ESAGIHETAYNSIM	P53479	1522,68	2,96E+06	3,77E+06	2,61E+06	High	761,84753	57,0347	3
RNSQRVIDSM	Q90339	1205,61	2,57E+06	3,79E+06	2,79E+06	High	402,54108	21,5018	2,81
LKKDIDDL	Q90339	959,54	1,86E+06	3,79E+06	3,39E+06	High	480,27542	27,7331	2,79
TALEEAEGTLEHE	Q90339	1428,65	3,07E+06	3,80E+06	3,77E+06	High	714,82928	65,8625	4,02
TIIDQNRDGIISKDDLRDVLA	O93409	2370,26	1,47E+07	3,83E+06	1,11E+07	High	790,76154	79,2031	4,77
IVRDIKEKL	P83750	1113,70	1,96E+06	3,84E+06	2,06E+06	High	371,90591	24,3716	2,63
AANLDKKQRNFDKVLA	Q90339	1831,02	2,47E+06	3,84E+06	1,09E+07	High	611,01355	28,0103	4,31
ASETVITLEN	A0A0N6WHT	1076,55	2,36E+06	3,85E+06	8,24E+05	High	538,77863	27,4579	2,04
QKYLEESTMRQFAMDAAATAAAQRDTT	A1YB07	3021,40	3,50E+06	3,89E+06	1,90E+06	High	605,08923	10,7484	2,95
MDEIEFLKKMHDEEIQDVQVSVQSQ	P31393	3021,42	3,50E+06	3,89E+06	1,90E+06	High	605,09003	10,7407	2,77
FYIVAYMAMGASRMSEQVTRHTLEDV	Q2EY13	3021,43	3,50E+06	3,89E+06	1,90E+06	High	605,08997	10,7333	2,72
GSQQQQQSLIYSQPGGFTVNGMLSPPGSQ	P79745	3021,44	3,50E+06	3,89E+06	1,90E+06	High	605,09137	10,734	3,53

SDHHVYLEGTLLKPN	Q8JH70	1722,88	3,85E+06	3,89E+06	4,88E+06	High	574,96643	49,0453	4,51
MYPGIADRMQKE	P83750	1438,68	2,49E+06	3,90E+06	4,29E+06	High	480,23386	40,5141	3,4
TKLEQQVDDLEGSLE	Q90339	1703,83	1,25E+07	3,91E+06	8,74E+06	High	852,42194	80,1759	3,47
LQDSLGGSAHSVMITNIAPEYKY	A8WFU8	2494,23	3,95E+06	3,96E+06	4,94E+06	High	499,65271	33,0693	4,3
QVEERDKEIE	Q5RG45	1274,62	2,17E+06	3,96E+06	3,62E+06	High	637,81622	27,3935	1,98
GQKGSSGVGQEQI	A1A5H6	1274,63	2,17E+06	3,96E+06	3,62E+06	High	637,81622	27,3935	1,93
KKDIDDLE	Q90339	975,50	7,42E+05	3,97E+06	2,50E+06	High	488,25513	19,5815	2,93
HQGVMVGM	P83750	858,40	2,61E+06	3,97E+06	3,29E+06	High	429,70294	35,3473	2,73
QLGETGKSIHELE	Q90339	1440,73	2,71E+06	3,98E+06	3,18E+06	High	720,87164	34,7075	2,66
SASTIEASPGVIANPFAAGIVRKNSME	Q4H4B6	2733,39	1,01E+07	4,01E+06	5,65E+06	High	456,40668	51,1374	3,28
QASAGRVMRRGAPPPPSVPNQTPPHQ	Q2TJA6	2733,41	1,01E+07	4,01E+06	5,65E+06	High	456,40668	51,1374	3,31
SSLSLASAINSKGIYVINAQNQ	A0A0R4IBK5	2278,20	7,94E+06	4,02E+06	4,10E+06	High	570,3103	58,4235	2,8
YDEAGPSIVHR	P53479	1243,61	1,53E+06	4,04E+06	2,38E+06	High	622,30853	30,3026	3,53
MFGEKLKGADPEDVIVS	O93409	1834,93	1,02E+07	4,07E+06	7,78E+06	High	612,3147	70,3877	3,99
VSSAAFLSGTNRHMQEVKGQD	A5WUY6	2262,09	3,00E+06	4,08E+06	2,61E+06	High	566,27905	20,6779	2,86
GQKDSYVGDEAQ	P83750	1296,57	2,34E+06	4,09E+06	3,82E+06	High	648,79016	20,3408	3,42
PQNAPGAAGYLELVDPPVCVT	A0JMF1	2168,07	2,56E+06	4,10E+06	3,19E+06	High	542,77527	28,3938	2,66
ADLSRELEE	Q90339	1061,51	1,95E+06	4,13E+06	1,81E+06	High	531,26038	41,2728	2,97
EQQVDDLEGSLE	Q90339	1361,61	3,54E+06	4,14E+06	2,71E+06	High	681,30829	67,3501	3,13
RRDLEESTLQHE	Q90339	1512,74	2,61E+06	4,15E+06	4,19E+06	High	504,91965	21,972	4,31
VATLAEATLQGGGQIVLAGETAAAVG	Q90X44	2368,27	8,26E+06	4,16E+06	7,48E+06	High	474,46069	47,3883	3,33
PLPAASLPPEYPWMKEKKASK	O42365	2368,27	8,26E+06	4,16E+06	7,48E+06	High	474,46054	47,4382	2,75
EAEGTLEHEESKIL	Q90339	1584,78	2,90E+06	4,17E+06	2,72E+06	High	528,93158	52,29	3
LGETGKSIHELE	Q90339	1312,67	2,45E+06	4,18E+06	3,14E+06	High	438,23108	32,2555	3,6
KLEGDLKL	Q90339	915,55	3,09E+06	4,18E+06	3,12E+06	High	458,28046	37,2232	3,65
YETDAIQRT	Q90339	1096,53	1,42E+06	4,18E+06	2,03E+06	High	548,76807	27,3649	2,67
YDEAGPSIVH	P53479	1087,51	1,74E+06	4,19E+06	2,04E+06	High	544,25763	37,5085	3,44
TPAAILEVLNA	Q2PW47	1111,64	3,10E+06	4,20E+06	3,11E+06	High	556,32288	69,8196	1,99
KDDLRDVL	O93409	973,53	2,32E+06	4,23E+06	1,31E+06	High	487,27069	42,9524	2,48
EEFREKIRADNSLVVVGGAD	Q499B3	2204,13	3,56E+06	4,24E+06	2,68E+06	High	441,63284	34,6937	4,2
EIRNTTPFVQSLDLSGNLLS	Q5U378	2204,16	3,56E+06	4,24E+06	2,68E+06	High	441,63284	34,6937	4,07
AVINETPLPIDLY	Q9W7R3	1457,79	1,19E+07	4,28E+06	8,29E+06	High	729,39905	113,7775	2,17
ANLDKKQRNFDKVL	Q90339	1688,94	2,52E+06	4,29E+06	2,95E+06	High	563,65363	26,9448	3,4

GFAGDDAPRAV	P83750	1075,52	1,53E+06	4,32E+06	1,51E+06	High	538,26373	38,0153	2,71
RVIISAPSADAPM	Q5XJ10	1327,70	4,16E+06	4,34E+06	9,37E+05	High	664,3576	57,2542	3,4
ELTSINGVPVAAPGI	A9JRE2	1437,79	2,19E+06	4,36E+06	2,87E+06	High	479,93796	24,8394	2,8
TKAKTKLEQQVDDLE	Q90339	1745,93	4,16E+06	4,40E+06	6,01E+06	High	582,64911	36,7546	4,02
SAGIHETAYNSIM	P53479	1393,64	3,29E+06	4,51E+06	2,98E+06	High	697,32605	54,3715	3,05
LGEQIDNLQRVKQK	Q90339	1668,94	2,79E+06	4,52E+06	5,02E+06	High	556,98627	35,0026	5,03
IIAPPERKY	P83750	1086,63	2,08E+06	4,52E+06	2,61E+06	High	362,88263	28,0012	2,63
KELSTDGKEVI	Q5GIT4	1218,66	2,33E+06	4,54E+06	3,53E+06	High	406,89163	21,3709	2,3
DHFVKL	Q5XJ10	758,42	2,51E+06	4,54E+06	3,04E+06	High	379,71454	31,8899	2,02
TERGYSFVT	P53479	1059,51	2,67E+06	4,55E+06	3,88E+06	High	530,26074	41,279	2,56
KGSTGHPGTPGEGGPPGPPGSPGSP	C7DZK3	2166,02	2,36E+06	4,57E+06	3,10E+06	High	722,67603	28,0395	2,37
LSKIEDEQSLGA	Q90339	1289,66	2,44E+06	4,60E+06	3,94E+06	High	645,33435	39,9795	2,96
AEKAADESERGMKVIENR	P13104	2033,01	3,16E+06	4,61E+06	3,11E+06	High	509,009	30,0133	5,22
KSWVSREALNV	A0A0R4IBK5	1288,70	2,16E+06	4,61E+06	3,16E+06	High	430,23465	25,1914	2,3
GTLEHEESKIL	Q90339	1255,65	3,27E+06	4,61E+06	4,27E+06	High	628,33179	39,5695	3,54
LAQIMVMSKALIVPNVT	Q8AXB3	1844,04	4,45E+06	4,64E+06	6,90E+06	High	461,76688	28,0752	2,86
MDLERAKRKLE	Q90339	1388,77	3,68E+06	4,68E+06	4,29E+06	High	463,59506	23,6462	2,59
GGTTMYPGIADRM	P83750	1369,62	3,56E+06	4,69E+06	3,64E+06	High	685,31726	61,5209	3,33
EEELKTVTN	P13104	1062,53	2,88E+06	4,70E+06	2,85E+06	High	531,77063	23,5318	2,9
ELEEEIEAE	Q90339	1090,48	3,80E+06	4,74E+06	4,63E+06	High	545,74591	55,407	2,45
RVAPEEHPVL	P83750	1146,63	2,69E+06	4,84E+06	6,07E+06	High	382,88132	29,3489	2,84
TVRNDNSSRFGKFIRIH	Q90339	2047,09	5,37E+06	4,93E+06	5,59E+06	High	512,5304	36,3457	6,95
KKMEGDLNEME	Q90339	1323,59	2,93E+06	4,96E+06	4,82E+06	High	441,87024	26,5872	3,17
GEIKAKTEALK	Q66HV4	1187,70	2,32E+06	4,96E+06	3,94E+06	High	396,57224	26,3339	2,3
GNAKTVRNDNSSRFGKFIRIH	Q90339	2417,29	8,16E+06	4,97E+06	8,96E+06	High	484,26578	31,2792	5,45
ISDLTEQLGETGKSIHELE	Q90339	2099,05	1,12E+07	4,98E+06	1,07E+07	High	700,35608	76,3367	5,23
PHAIMR	P53479	724,39	2,67E+06	5,00E+06	3,94E+06	High	362,70071	28,4995	2,03
LTGMKNIGN	A5PN09	947,50	2,59E+06	5,00E+06	4,39E+06	High	474,25394	30,1448	2,65
QDVGHVMPGALMCIVKPD	Q6NVJ5	1982,95	3,43E+06	5,00E+06	4,98E+06	High	496,49277	26,9774	2,9
ASAAPHTPQTPSQPGPPSTPG	Q06725	1982,96	3,43E+06	5,00E+06	4,98E+06	High	496,49258	27,4931	2,87
IYGQSFYLVLTVQAKE	A0A0G2KIZ8	1858,99	1,08E+07	5,01E+06	6,41E+06	High	465,50391	50,7026	2,82
VIFDVSENPDAIDQLN	A5WWA0	1788,87	5,46E+06	5,02E+06	9,29E+06	High	894,94189	57,578	2,1
CRTLVESGVDELPSNI	A5D8S5	1788,88	5,46E+06	5,02E+06	9,29E+06	High	894,94226	57,8505	2,19

LGVKGIP	Q6TLF6	683,45	3,55E+06	5,02E+06	1,54E+06	High	342,22702	28,0683	2
AVGKVIPELNGKLTGM	Q5MJ86	1626,92	5,96E+06	5,03E+06	6,86E+06	High	542,98138	67,9137	3,94
LEEELKTVTNN	P13104	1289,66	2,55E+06	5,07E+06	3,94E+06	High	645,33441	39,5785	2,63
MYPGIADRM	P83750	1069,48	2,07E+06	5,17E+06	3,82E+06	High	535,24542	54,5638	2,05
ISERLEEA	Q90339	946,48	3,03E+06	5,19E+06	4,42E+06	High	473,74661	28,1377	2,26
AVGKVIPELNGK	Q5MJ86	1224,73	2,96E+06	5,24E+06	5,29E+06	High	612,87061	37,0828	3,7
MDAIKKKM	P13104	1006,54	2,91E+06	5,27E+06	1,57E+06	High	503,77661	38,4576	2,81
FAGDDAPR	P83750	848,39	2,23E+06	5,29E+06	3,12E+06	High	424,70062	18,759	2,28
GQKDSYVGDEA	P83750	1168,51	3,37E+06	5,29E+06	4,37E+06	High	584,76105	21,3046	3,14
FMTQSGPTASKDSGSSNGGSIDSGI	Q7ZVS3	2388,06	2,59E+06	5,30E+06	2,93E+06	High	796,69556	33,9034	2,31
SGGQANPSSSGSENAPSSPRGSVSSP	B0V207	2388,07	2,59E+06	5,30E+06	2,93E+06	High	796,6958	33,7477	2,43
GSPWSSSPVGMTGSPQQQDVKNN	P79745	2388,09	2,59E+06	5,30E+06	2,93E+06	High	796,69556	33,9034	2,36
PWSSSPVGMTGSPQQQDVKNNSG	P79745	2388,09	2,59E+06	5,30E+06	2,93E+06	High	796,69556	33,9034	2,33
KVIPELNGKLT	Q5MJ86	1211,74	3,88E+06	5,33E+06	2,75E+06	High	606,37372	42,2544	2,82
LWRDGRGASQ	Q5XJ10	1145,58	9,18E+05	5,33E+06	2,32E+06	High	382,53311	17,0944	2,36
KEAFTIIDQNRDGIISKD	O93409	2063,08	6,08E+06	5,35E+06	6,18E+06	High	688,36523	55,1841	6,33
FRENLGKLMTN	Q90339	1322,69	5,28E+06	5,39E+06	8,25E+06	High	441,5683	52,0167	2,61
RLCKAREQELNYK	Q6P3L0	1707,90	3,02E+06	5,39E+06	5,47E+06	High	569,96643	35,7662	2,3
AQAPRIMTGPTPVMPNAAL	Q6P104-2	1936,01	7,85E+06	5,41E+06	9,03E+06	High	646,01074	62,5634	2,91
LENKDAAL	A0A1D5NSK(	873,47	2,85E+06	5,42E+06	3,88E+06	High	437,23935	39,3387	2,05
RGGGTVAGAPRGRSSSRGR	Q4KME6	1841,99	5,69E+06	5,43E+06	5,22E+06	High	461,24982	36,4114	2,61
KDIDDLELT	Q90339	1061,54	3,49E+06	5,46E+06	2,78E+06	High	531,27325	56,7457	2,59
YPGIADRMQKEITAL	P53479	1705,89	1,93E+07	5,47E+06	1,02E+07	High	853,45178	80,869	3,64
FPSIVGRPRHQ	P83750	1293,72	4,55E+06	5,53E+06	5,56E+06	High	431,91223	28,9171	2,8
SLMKVHLMVA	Q9W7R3	1144,62	2,19E+06	5,55E+06	7,84E+06	High	382,21448	24,2996	2,44
KSYELPDGQVIT	P83750	1349,69	4,08E+06	5,55E+06	1,82E+06	High	675,35254	60,6711	3,43
STGVFTTIEKA	Q5XJ10	1153,61	3,10E+06	5,57E+06	5,30E+06	High	577,31006	54,647	2,8
ISDLTEQLGETGKSIHEL	Q90339	1970,01	1,04E+07	5,59E+06	9,94E+06	High	657,34204	74,5485	5,04
KNTTATASLFSSQAVAGSDFITMTAQTT	Q6JAN0	2866,38	5,70E+06	5,59E+06	6,80E+06	High	574,08624	35,3877	3,12
EEKKEEEAKAEETPAAPVETPKAEEP	Q6NWH2	2866,38	5,70E+06	5,59E+06	6,80E+06	High	574,08563	34,726	4,93
GLKGDPGERGEPGWNGTKGGMGEPGKQGL	A5PN28	2866,39	5,70E+06	5,59E+06	6,80E+06	High	574,08643	35,4224	3,35
PQFQNMPSIVDVPENTTSGSSIYKVQ	Q6B3P0	2866,39	5,70E+06	5,59E+06	6,80E+06	High	574,08624	35,3877	3,26
IGESGEVGPKGFPGIQGPSGATGDKGIAGEP	C7DZK3	2866,42	5,70E+06	5,59E+06	6,80E+06	High	574,08582	34,3707	4,73

LDKKQRNFDKVLAE	Q90339	1703,94	4,07E+06	5,60E+06	5,22E+06	High	568,65466	26,9161	4,08
SYVGDEAQSKRGILT	P83750	1623,83	3,45E+06	5,62E+06	5,18E+06	High	541,95105	40,1991	4,12
YLAQQKSVMSSPVALTASSASPV	A5PLF5	2338,20	2,24E+06	5,63E+06	3,03E+06	High	585,30212	37,9396	4,21
GFTADEKISIY	Q90339	1243,62	5,05E+06	5,65E+06	6,25E+06	High	622,31555	58,237	2,3
GDEAQSKRGILT	P83750	1274,67	2,75E+06	5,70E+06	4,38E+06	High	637,83466	34,5823	2,19
KLDKENALDRAE	P13104	1401,73	3,22E+06	5,75E+06	4,89E+06	High	701,37164	19,4776	3,25
LSDLQGSNAINQ	E7EZG2	1259,62	2,30E+06	5,78E+06	2,17E+06	High	630,32019	55,8632	1,96
EAEKTCLLAPQ	A0A8M9QN1[illegible]	1259,63	2,30E+06	5,78E+06	2,17E+06	High	630,3197	55,7227	2,15
AFTIIDQNRDGIISKD	O93409	1805,94	6,86E+06	5,81E+06	9,60E+06	High	903,4743	63,1287	4,8
DKKQRNFDKVLAE	Q90339	1590,86	4,38E+06	5,83E+06	5,63E+06	High	530,96021	23,727	3,95
GETGKSIHEL	Q90339	1070,55	9,13E+05	5,85E+06	2,89E+06	High	535,77899	19,1715	2,91
SYVGDEAQSKRGIL	P83750	1522,79	3,78E+06	5,87E+06	4,23E+06	High	508,26834	41,7976	3,76
DNGSGLVKAG	P53479	917,47	3,08E+06	5,87E+06	3,36E+06	High	459,23898	22,4063	2,59
RVIQYF	Q90339	825,46	4,35E+06	5,89E+06	9,71E+06	High	413,23557	48,6343	2,05
APLNPKANREKMTQIMFE	P83750	2118,08	1,21E+07	5,99E+06	1,02E+07	High	530,27789	52,9922	3,52
VFDKEGNGTVM	Q6P0G6	1196,56	3,47E+06	6,01E+06	3,08E+06	High	598,78552	38,8984	3,87
RPRHQGVMVGM	P83750	1267,65	4,83E+06	6,02E+06	6,93E+06	High	423,22263	19,2093	2,99
KEVIAI	A4QP72	672,43	2,79E+06	6,02E+06	8,30E+06	High	336,71869	40,3031	2,18
LDFENEMATAA	P53479	1211,52	5,50E+06	6,04E+06	4,16E+06	High	606,26813	79,6789	2,2
EQIDNLQRVKQKLE	Q90339	1740,96	5,81E+06	6,05E+06	5,60E+06	High	580,99292	42,4372	3,47
LEEISERLEE	Q90339	1246,62	4,81E+06	6,06E+06	7,31E+06	High	623,81281	51,1269	2,92
DKENALDRAEQA	P13104	1359,65	2,97E+06	6,07E+06	3,81E+06	High	680,33026	26,8681	3,67
KSYELPDGQVI	P83750	1248,65	4,21E+06	6,08E+06	5,52E+06	High	624,82776	67,6679	3,31
IEDEQSLGAQ	Q90339	1089,51	2,81E+06	6,11E+06	2,80E+06	High	545,25812	34,6199	2,68
KSYELPDGQ	P83750	1036,49	2,56E+06	6,11E+06	3,90E+06	High	518,75244	33,6729	3,51
KIEDEQSLGAQL	Q90339	1330,68	4,19E+06	6,13E+06	5,03E+06	High	665,84735	52,1387	3,53
VEKQRADLSREL	Q90339	1443,79	3,89E+06	6,22E+06	5,68E+06	High	481,93686	25,0041	3,67
GSLEQEKKLRMDLE	Q90339	1675,87	4,97E+06	6,22E+06	5,24E+06	High	559,29657	42,6613	2,84
QGPEPVDGTVVEYEVTYYEKNQQ	O13146	2672,24	3,48E+06	6,22E+06	4,71E+06	High	891,41541	27,6925	2,32
EDELDKY	P13104	911,40	3,01E+06	6,25E+06	4,60E+06	High	456,20428	32,3212	2,03
QMLQQLKNLALEAE	Q0P4A7	1644,86	9,02E+06	6,32E+06	8,28E+06	High	548,95935	58,3822	2,46
AEGTLEHEESKIL	Q90339	1455,73	3,77E+06	6,37E+06	6,36E+06	High	728,37183	45,6021	4,14
MDAIKKKMQML	P13104	1378,73	5,34E+06	6,44E+06	6,90E+07	High	689,86719	78,1761	3,7

DKKQRNFDKVL	Q90339	1390,78	3,38E+06	6,47E+06	6,20E+06	High	348,45132	19,4055	3,55
WEGSKLQGGSAYLPGMPDIQW	Q98864	2336,10	4,49E+06	6,49E+06	5,75E+06	High	779,37366	38,2802	2,81
EFIGFTRDEATKQQFKDEF	Q6AZB8	2336,12	4,49E+06	6,49E+06	5,75E+06	High	779,37305	38,5082	2,58
TKAKTKLEQQVDDLEGSLE	Q90339	2132,11	2,54E+07	6,58E+06	2,00E+07	High	533,78394	68,1112	4,49
QAEEDKVNTL	Q90339	1146,56	3,06E+06	6,63E+06	4,18E+06	High	573,78705	28,2226	2,5
VDKGVVPL	Q8JH70	826,50	2,07E+06	6,63E+06	6,71E+06	High	413,75644	42,0551	2,25
EFKENK	E7EZG2	794,40	3,94E+06	6,67E+06	4,66E+06	High	397,70193	28,1999	1,99
REIVRDIKE	P83750	1157,66	3,25E+06	6,67E+06	4,99E+06	High	386,56058	17,6106	2,36
KIEDEQSLGA	Q90339	1089,54	3,48E+06	6,74E+06	3,40E+06	High	545,27625	28,9247	2,54
DDMEKIWHH	P83750	1210,53	3,48E+06	6,78E+06	5,75E+06	High	605,77002	39,838	3,49
FGEKLKGADPEDVIVS	O93409	1703,89	6,83E+06	6,87E+06	6,82E+06	High	568,63434	61,0825	3,3
SLQLQTKELKT	A0A2R8QCI3	1288,75	3,66E+06	6,93E+06	1,97E+06	High	430,25534	32,7554	2,32
KEKEKEKTRL	Q6DRG7	1288,76	3,66E+06	6,93E+06	1,97E+06	High	430,25534	32,7554	2,31
DSGDGVTHTVPIYE	P83750	1489,68	5,47E+06	6,96E+06	7,00E+06	High	745,34393	58,5131	1,91
LEHEESKIL	Q90339	1097,58	2,88E+06	6,98E+06	4,56E+06	High	549,29742	28,0636	2,86
FIDKAEIT	B0S5N4	936,50	4,19E+06	7,02E+06	4,91E+06	High	468,75677	43,0503	2,32
KGADPEDVIVS	O93409	1129,57	2,70E+06	7,03E+06	3,84E+06	High	565,29193	42,3713	1,98
KDIDDLE	Q90339	847,40	5,00E+06	7,05E+06	4,40E+06	High	424,20715	30,3311	2,41
IDIRKDLYAN	P53479	1220,66	3,82E+06	7,06E+06	4,04E+06	High	610,8363	48,1217	2,9
KKIELSDL	Q6Q7X9	945,56	4,20E+06	7,15E+06	5,60E+06	High	473,28619	30,9822	1,92
KSKELDII	Q7ZUV7	945,56	4,20E+06	7,15E+06	5,60E+06	High	473,28607	30,5185	2,07
VLDASSKIL	Q6JAN0	945,56	4,20E+06	7,15E+06	5,60E+06	High	473,28601	31,5025	2,02
TIIDQNRDGIISK	O93409	1472,81	4,26E+06	7,20E+06	6,17E+06	High	736,90857	40,0967	4,03
EEISERL	Q90339	875,45	4,74E+06	7,23E+06	4,22E+06	High	438,22836	34,6902	2,19
LGEQIDNL	Q90339	901,46	4,48E+06	7,31E+06	5,54E+06	High	451,23633	63,1286	2,14
EVNGDISIDTSTAKGLQKTSAEKESSVA	E7F4Z4	2865,43	1,52E+07	7,34E+06	1,37E+07	High	573,89777	53,3445	2,8
TDNRTKREKELQENSEALKTKMSE	Q5U3N0	2865,44	1,52E+07	7,34E+06	1,37E+07	High	573,89807	53,5759	2,68
KSETGAAYIRFINTHTENINVTVGTE	B0S6T2	2865,44	1,52E+07	7,34E+06	1,37E+07	High	573,89795	53,3789	2,67
ETEQASQKKFVRMVSVTDVQQGDVR	Q5PZ43	2865,45	1,52E+07	7,34E+06	1,37E+07	High	573,89801	54,362	2,77
KKDIDDLEL	Q90339	1088,58	5,59E+06	7,41E+06	6,28E+06	High	363,53354	44,3766	3,04
KAQLRDTELELSDL	Q6DRG7	1630,86	5,81E+06	7,41E+06	6,40E+06	High	544,29816	26,7377	2,41
REKMTQIMFE	P83750	1312,64	5,72E+06	7,46E+06	7,55E+06	High	438,21902	46,4274	2,89
AGDDAPRAVFPSIVGRPRHQ	P83750	2146,13	6,96E+06	7,49E+06	1,07E+07	High	430,03268	43,9571	5,68

TVDGPSGKLW	Q5XJ10	1059,55	4,35E+06	7,50E+06	4,73E+06	High	530,27863	54,2302	2,44
GFAGDDAPRA	P83750	976,45	5,24E+06	7,51E+06	4,35E+06	High	488,72903	27,7333	2,45
NFKAGARALTDAETK	P05939	1592,84	5,28E+06	7,53E+06	6,52E+06	High	531,61993	28,3877	4,03
YDEAGPSIVHRK	P53479	1371,70	3,49E+06	7,54E+06	5,28E+06	High	457,90674	21,253	3,34
MSKVLVQ	A9C3R9	804,46	3,81E+06	7,58E+06	6,76E+06	High	402,73755	32,8419	2,1
LGNAIMGSGILGLSYAMANTGIA	Q5SPB1	2211,11	4,85E+06	7,60E+06	9,02E+06	High	553,53577	36,0743	2,72
EISDLKNKV	A7MD70	1045,59	5,82E+06	7,61E+06	2,00E+06	High	523,2995	33,7753	1,97
KGSIHKFVPYLI	Q5RGV1	1401,83	7,35E+06	7,77E+06	8,85E+06	High	467,94971	32,4847	2,79
EKAGIMRKRALL	A5PF44	1401,84	7,35E+06	7,77E+06	8,85E+06	High	467,95001	32,5181	2,71
TRDKMNINHEGQTTVPPMITQPTLQ	A0MSJ1	2866,42	7,85E+06	7,79E+06	9,31E+06	High	574,08582	34,3707	4,84
ISDLTEQLGETGKSIHE	Q90339	1856,92	6,00E+06	7,85E+06	8,73E+06	High	619,64758	58,4883	5,17
TEAPLNPKANREKMTQIMFETF	P83750	2596,29	6,16E+07	7,91E+06	2,51E+07	High	866,10339	81,0552	3,96
TEAPLNPKANREKMTQIMFE	P83750	2348,17	1,48E+07	7,94E+06	1,41E+07	High	783,39716	58,8451	3,51
ALTDAETKAFLK	P09227	1307,72	4,68E+06	7,95E+06	6,10E+06	High	654,3653	50,3973	3,85
APEEHPTLL	P53479	1006,52	2,78E+06	8,00E+06	3,62E+06	High	503,76532	42,6313	2,28
FIGMESAGIHET	P53479	1291,60	6,51E+06	8,01E+06	5,47E+06	High	646,30426	56,891	2,71
QLMPAMVQKRMQEMESKAAEMAKAE	Q568N4	2866,38	8,25E+06	8,10E+06	9,48E+06	High	478,57233	34,3378	4,29
ELEEELKTVTNN	P13104	1418,70	4,78E+06	8,11E+06	5,61E+06	High	709,85468	56,7369	3,95
KDSYVGDEAQSKRGIL	P83750	1765,91	5,89E+06	8,13E+06	5,89E+06	High	589,30963	34,9077	7,46
KLKGADPEDVIVS	O93409	1370,75	5,46E+06	8,16E+06	7,14E+06	High	457,59058	40,7748	3,37
RADLSRELEE	Q90339	1217,61	4,94E+06	8,18E+06	7,62E+06	High	406,54355	31,1323	3,01
ANLDKKQRNFDKVLAE	Q90339	1889,02	7,39E+06	8,18E+06	7,82E+06	High	630,34723	30,3669	4,73
RVIDSM	Q90339	720,37	2,23E+06	8,24E+06	7,63E+06	High	360,68985	23,7927	2,07
SDHHVYLE	Q8JH70	999,45	2,75E+06	8,26E+06	4,53E+06	High	500,23135	21,5243	2,38
EREIVRDIKE	P83750	1286,71	4,32E+06	8,26E+06	6,47E+06	High	429,57495	21,4728	3,33
ADESERGMKVIEN	P13104	1477,70	4,56E+06	8,31E+06	3,92E+06	High	739,35284	29,3193	2,52
KDSYVGDEAQSKRGILT	P83750	1866,96	4,68E+06	8,47E+06	6,10E+06	High	622,99097	33,6672	7,72
RRDLEESTL	Q90339	1118,58	5,73E+06	8,49E+06	7,46E+06	High	373,53293	25,5651	2,84
VVYPQTKTY	P82315	1098,58	4,70E+06	8,49E+06	7,79E+06	High	549,79681	33,0496	2,02
AIQRTEELEE	Q90339	1217,60	5,90E+06	8,61E+06	6,72E+06	High	609,3053	28,8963	3,07
QSTDRLHELNTLRD	A2BIR6	1697,86	5,25E+06	8,65E+06	8,21E+06	High	566,6181	39,4648	2,55
DLRDVLAT	Q66I73	902,49	3,90E+06	8,73E+06	6,55E+06	High	451,75204	64,1789	2,42
TEAPLNPK	P83750	869,47	9,15E+05	8,76E+06	4,61E+06	High	435,24133	18,3667	2,37

AEREIVRDIKEK	P83750	1485,84	5,50E+06	8,77E+06	8,46E+06	High	372,21616	17,4291	2,81
GVQGERGKEGAPGVPGNSG	A0MSJ1	1752,86	3,87E+06	8,78E+06	6,10E+06	High	584,9566	18,8746	2,44
TLTPPTSPEEAQAVSQP	A2VCZ5	1752,87	3,87E+06	8,78E+06	6,10E+06	High	584,95703	18,9644	2,65
TAEREIVRDIKE	P83750	1458,79	5,34E+06	8,83E+06	6,81E+06	High	486,93646	29,1605	4,49
IIDQNRDGIIS	O93409	1243,66	4,19E+06	8,96E+06	3,13E+06	High	622,33734	41,1706	2,88
TEAPLNPKANRE	P83750	1339,70	4,53E+06	9,01E+06	7,99E+06	High	447,23825	17,9867	3,24
FAGDDAPRAVFPSIVGRPRHQ	P83750	2293,19	1,34E+07	9,04E+06	1,66E+07	High	459,44629	53,9428	5,79
KTIEANGKMVIPGGIDI	Q52PJ5	1771,96	1,06E+07	9,06E+06	8,48E+06	High	591,32495	41,1259	2,93
QLADAAAGFTLLALDMPGSAPQNLQP	B3DIY3	2626,32	7,98E+06	9,09E+06	9,98E+06	High	876,10858	38,2288	2,38
IVAPGKGILA	Q8JH70	938,60	3,66E+06	9,10E+06	1,42E+07	High	469,80667	48,1474	2,02
GGEGLRLEDGQ	Q1LYE3	1130,54	5,38E+06	9,10E+06	5,91E+06	High	565,77667	24,1606	2,46
KKDIDDLELTL	Q90339	1302,72	9,32E+06	9,12E+06	1,46E+07	High	651,86285	67,0837	3,03
EDQLSEIK	Q90339	961,48	3,91E+06	9,16E+06	5,26E+06	High	481,24652	30,544	2,39
GESGAGKTVNTKRVIQYF	Q90339	1955,03	1,03E+07	9,16E+06	1,19E+07	High	652,35077	45,6949	5
GFAGDDAPRAVF	P83750	1222,59	9,87E+06	9,19E+06	1,53E+06	High	611,79791	63,6395	3,67
ELPDGQVITIG	P83750	1141,61	7,36E+06	9,28E+06	7,25E+06	High	571,30981	81,1456	2,12
VIPEGQFIDNK	Q90339	1259,66	5,35E+06	9,29E+06	6,51E+06	High	630,336	50,8705	2,34
REKMTQIM	P83750	1036,53	4,33E+06	9,30E+06	7,67E+06	High	346,18158	24,8795	2,35
LSKIEDEQSL	Q90339	1161,60	5,10E+06	9,32E+06	7,76E+06	High	581,30469	38,7415	3,92
LEQQVDDLE	Q90339	1088,51	5,56E+06	9,43E+06	6,15E+06	High	544,76086	48,038	2,57
GQKDSYVGDEAQSKR	P83750	1667,80	3,36E+06	9,50E+06	7,10E+06	High	417,70599	13,5628	3,6
TDAIQRTEELEE	Q90339	1433,68	6,13E+06	9,76E+06	7,45E+06	High	717,34296	46,2346	4,22
SKIEDEQSLGAQL	Q90339	1417,72	5,75E+06	9,77E+06	9,42E+06	High	709,36389	53,5297	3,71
DLRDVLAS	O93409	888,48	4,97E+06	9,85E+06	6,44E+06	High	444,74426	61,9988	2,12
EKDNTNVLIT	A2BGG1	1146,60	4,51E+06	9,93E+06	7,41E+06	High	573,80585	38,1812	2,23
KQEYDEAGPSIVHRK	P53479	1756,90	6,85E+06	1,02E+07	7,74E+06	High	586,3056	20,7103	7,21
PPFAQGLASPQGAMTPGLP	Q7SXL3	1836,93	6,01E+07	1,04E+07	3,35E+07	High	612,98425	86,028	2,98
MYPGIADRMQKEITAL	P53479	1836,93	6,01E+07	1,04E+07	3,35E+07	High	612,98462	85,7391	2,69
KQRADLSRELEE	Q90339	1473,77	7,51E+06	1,05E+07	9,28E+06	High	491,92831	23,917	3,53
TALEEAEGTLE	Q90339	1162,55	7,93E+06	1,05E+07	9,20E+06	High	581,77917	65,8685	3,09
EKLKGADPEDVIVS	O93409	1499,80	6,95E+06	1,05E+07	5,18E+06	High	500,60492	44,4567	3,04
SASKLYQTEVSDANGIA	F1R237	1753,86	6,95E+06	1,06E+07	5,58E+06	High	585,28717	48,9554	2,59
GDVSFLKEDFEIQLN	Q803G5	1753,86	6,95E+06	1,06E+07	5,58E+06	High	585,28754	48,9482	2,59

PAGHAQGGVPAVAMPYFVVQSPLIAGAMP	Q5RIX9	2865,44	2,17E+07	1,08E+07	2,03E+07	High	717,12	53,5804	2,71
NFDKVLAE	Q90339	935,48	6,66E+06	1,08E+07	6,96E+06	High	468,24701	44,7614	2,47
FPPDVAGNVDYK	O93409	1321,64	1,04E+07	1,08E+07	8,50E+06	High	661,32666	62,2274	3,28
TEAPLNPKAN	P83750	1054,55	4,73E+06	1,08E+07	7,84E+06	High	527,78253	21,2592	2,33
GEQIDNLQRVKQKLEKEKSEYKME	Q90339	2950,53	2,83E+07	1,08E+07	2,61E+07	High	738,39001	55,413	4,9
IIDQDKSGFIE	P02618	1264,64	6,01E+06	1,09E+07	6,92E+06	High	632,82581	49,4263	2,85
KGDLFIAGL	A1XQX8	933,54	6,85E+06	1,09E+07	8,54E+06	High	467,27512	58,7265	1,9
IIDQNRDGIISKD	O93409	1486,79	6,94E+06	1,09E+07	5,57E+06	High	743,89795	31,4018	3,46
AVGKVIPELN	Q5MJ86	1039,61	5,50E+06	1,10E+07	7,18E+06	High	520,31238	50,6873	2,91
LEGTLLKPN	Q8JH70	984,57	4,53E+06	1,11E+07	8,38E+06	High	492,79138	39,6087	3,11
VPTPNVSVVDL	Q5XJ10	1139,63	1,13E+07	1,12E+07	6,99E+06	High	570,3205	87,6529	2,72
RKRDSLDKY	Q1LWL6	1180,64	6,44E+06	1,13E+07	1,07E+07	High	394,21808	34,7916	3
NALDRAEQAE	P13104	1116,53	5,12E+06	1,13E+07	7,94E+06	High	558,76978	29,5214	3,7
WADLSPGSGPVKKH	Q90487	1478,78	6,06E+06	1,13E+07	9,20E+06	High	493,59787	26,3693	2,52
FIGMESAGIH	P53479	1061,51	6,33E+06	1,13E+07	5,98E+06	High	531,25891	56,2245	2,93
ELEEELKTV	P13104	1089,57	5,25E+06	1,14E+07	4,84E+06	High	545,289	52,4914	3,16
GQAQPVEQDQFPGQ	Q98TW1	1528,70	4,64E+06	1,14E+07	6,73E+06	High	764,86157	35,8981	1,97
RSTFHALGCTGLEEL	Q7SY48	1690,82	9,77E+06	1,16E+07	1,08E+07	High	564,28308	48,9637	2,39
KDMLIVETADMLQAP	Q1L8G6	1690,84	9,77E+06	1,16E+07	1,08E+07	High	564,284	49,079	2,55
SKMAPQSSGSLRSNAGI	A0A0R4IES7	1690,85	9,77E+06	1,16E+07	1,08E+07	High	564,284	49,079	2,47
AGDDAPRAVFP	P83750	1115,55	6,95E+06	1,16E+07	4,72E+06	High	558,27936	50,6937	3,28
GFAGDDAPRAVFPSIVGRPR	P83750	2085,10	1,94E+07	1,17E+07	1,58E+07	High	695,70526	65,499	5,46
GLNSADMLKAL	Q90339	1132,60	1,07E+07	1,18E+07	1,38E+07	High	566,8064	78,7404	3,65
KAADESERGMKVIENR	P13104	1832,93	7,87E+06	1,20E+07	7,84E+06	High	611,6499	24,6515	4,35
LGEQIDNLQRVK	Q90339	1412,79	6,17E+06	1,21E+07	9,36E+06	High	471,60153	42,4193	5,05
LVDASERVGL	Q90339	1058,58	9,70E+06	1,21E+07	3,44E+06	High	529,797	48,9242	2,71
KMEGDLNEM	Q90339	1066,45	7,85E+06	1,22E+07	5,95E+06	High	533,73224	35,4064	2,76
DDLRDVLA	O93409	916,47	6,17E+06	1,22E+07	9,63E+06	High	458,74207	61,1206	1,95
KDDLRDVLA	O93409	1044,57	4,84E+06	1,23E+07	7,96E+06	High	522,78912	42,0595	2,99
MDAIKK	P13104	747,41	6,60E+06	1,23E+07	4,76E+06	High	374,20825	27,785	1,93
TVDGPSGKLWRDGRGASQ	Q5XJ10	1886,95	9,60E+06	1,24E+07	9,78E+06	High	472,4931	32,0893	4,25
IEKGDLKSL	Q5PZ43	1002,58	6,96E+06	1,26E+07	8,86E+06	High	501,79721	33,0687	2,54
LAEKDEEME	Q90339	1093,47	8,24E+06	1,26E+07	8,29E+06	High	547,24139	20,9011	2,95

WDDMEKIWHH	P83750	1396,61	1,06E+07	1,26E+07	1,35E+07	High	698,81024	57,6457	4,38
NLLSALMAYAGMVIGTAVG	Q6PEH9	1867,97	1,13E+07	1,26E+07	1,50E+07	High	467,74985	37,3038	2,86
DLAGRDLTDYL	P83750	1251,62	1,69E+07	1,28E+07	8,39E+06	High	626,31549	90,2609	2,75
FAGDDAPRAVFP	P83750	1262,62	9,54E+06	1,28E+07	7,06E+06	High	631,81317	66,108	3,34
KIEDEQSL	Q90339	961,48	6,98E+06	1,29E+07	5,70E+06	High	481,24628	26,2766	2,77
NLDKKQRNFDKVL	Q90339	1617,91	9,23E+06	1,29E+07	1,36E+07	High	539,97522	25,9503	4,14
TEQLGETGKSIHE	Q90339	1428,70	5,70E+06	1,31E+07	9,30E+06	High	476,90515	25,1606	4,22
LKKDIDDLE	Q90339	1088,58	6,80E+06	1,32E+07	1,36E+07	High	544,797	29,2236	2,85
NWDDMEKIWHHT	P83750	1611,70	1,65E+07	1,33E+07	1,63E+07	High	806,35632	69,8607	3,68
VDASERVGLL	Q90339	1058,58	9,80E+06	1,35E+07	1,21E+07	High	529,79706	55,3666	3,01
GEQIDNLQRVKQK	Q90339	1555,86	6,86E+06	1,35E+07	1,03E+07	High	519,29144	22,0349	3,64
GFAGDDAPRAVFPSIV	P83750	1618,82	2,34E+07	1,36E+07	2,27E+07	High	809,91626	92,3451	3,55
EEAEGTLEHEESKIL	Q90339	1713,82	1,17E+07	1,36E+07	1,39E+07	High	571,94519	57,9532	2,97
ASGRTTGIVL	P53479	974,56	7,95E+06	1,37E+07	1,24E+07	High	487,78656	37,6714	2,61
LGEQIDNLQRVKQKLE	Q90339	1911,07	1,73E+07	1,38E+07	2,10E+07	High	637,6944	55,2319	6,04
LAEKDEEMEQIKRN	Q90339	1732,85	8,10E+06	1,38E+07	1,10E+07	High	578,29059	25,5282	5,93
GIHETAYNSIM	P53479	1235,57	9,30E+06	1,39E+07	8,45E+06	High	618,29199	51,5747	3,5
VEKQRADLSRELEE	Q90339	1701,88	1,03E+07	1,39E+07	1,33E+07	High	567,96515	28,8227	3,74
NWDDMEKIWH	P83750	1373,59	1,75E+07	1,41E+07	5,42E+06	High	458,53738	78,9075	3,39
TEQLGETGKSIHEL	Q90339	1541,78	9,20E+06	1,42E+07	1,38E+07	High	514,59973	39,834	3,78
LEGAQKEARSL	Q90339	1201,65	6,60E+06	1,44E+07	1,10E+07	High	401,22418	21,1456	3,87
ALTDAETKAF	P09227	1066,54	7,04E+06	1,48E+07	8,66E+06	High	533,77625	46,3152	3,15
KVLDPEAT	Q66I73	872,47	6,29E+06	1,51E+07	9,83E+06	High	436,74155	22,9447	2,66
EYDEAGPSIVH	P53479	1216,55	7,07E+06	1,55E+07	1,06E+07	High	608,77844	41,3712	2,9
RIQLVEEE	P13104	1015,54	1,21E+07	1,57E+07	1,17E+07	High	508,27679	37,9139	2,45
LVDASERVGLL	Q90339	1171,67	1,47E+07	1,60E+07	2,59E+07	High	586,33929	65,1856	2,78
KLDKENALDRA	P13104	1272,69	8,69E+06	1,65E+07	1,31E+07	High	424,90234	16,0244	3,37
PTNVTGSPQL	Q6DGH9-2	1013,53	6,77E+06	1,66E+07	9,61E+06	High	507,26831	29,7951	1,9
KMTQIMFE	P83750	1027,50	9,77E+06	1,68E+07	1,74E+07	High	514,25281	55,6978	2,31
GQLNVKNEELE	O93409	1272,64	1,15E+07	1,69E+07	1,18E+07	High	636,82623	37,7493	2,97
ISDLTEQL	Q90339	918,48	1,01E+07	1,69E+07	1,25E+07	High	459,74451	64,6663	2,05
PSGFSEPNTFLAELE	Q1LWL6	1637,77	1,17E+07	1,72E+07	1,51E+07	High	546,59924	34,4562	3,18
KDFSAPGSVSAGSAGGVSG	Q4TVV3	1637,78	1,17E+07	1,72E+07	1,51E+07	High	546,60028	35,177	2,55

SKIEDEQSLGA	Q90339	1176,57	8,45E+06	1,72E+07	1,29E+07	High	588,79224	30,0318	3,29
AQVTYLAHLVEGLLNMING	Q802D3	2072,08	1,61E+07	1,76E+07	1,80E+07	High	518,77277	43,0085	3
IEYLHQNDI	Q696W0	1144,56	1,01E+07	1,79E+07	1,02E+07	High	572,78442	39,8488	1,92
EGEINQQPAEEAIQVDPAN	X1WE18	2051,95	7,42E+06	1,82E+07	1,17E+07	High	684,66156	33,0896	2,42
PEIVISVAGLLNA	Q1LXK4	1295,76	1,99E+07	1,83E+07	2,19E+07	High	648,38312	83,8843	2,33
SERGMKVIENR	P13104	1318,69	6,03E+06	1,88E+07	1,00E+07	High	440,23615	16,1363	3,74
TEQLGETGKSIHELE	Q90339	1670,82	1,27E+07	1,89E+07	1,71E+07	High	557,61383	41,0858	4,18
LDHALNDMTSI	P13104	1229,58	1,17E+07	1,89E+07	2,23E+07	High	615,29614	61,7652	3,25
DIDIRKDLYANN	P53479	1449,73	9,18E+06	1,92E+07	1,18E+07	High	725,37128	58,0289	3,75
RVFDKEGNGTVM	Q6P0G6	1352,66	1,11E+07	1,93E+07	1,81E+07	High	676,83655	31,5887	3,91
LSKIEDEQSLGAQ	Q90339	1417,72	9,64E+06	1,98E+07	1,66E+07	High	709,36444	38,2854	3,45
LDHALNDM	P13104	928,42	9,56E+06	2,03E+07	1,68E+07	High	464,71457	34,6347	2,35
DKENALDRAEQAE	P13104	1488,69	1,24E+07	2,22E+07	1,75E+07	High	496,90408	32,1671	4,46
TTVTTEVNGRGLPALSSRSSPMS	Q5TZ18	2348,19	3,33E+07	2,29E+07	2,96E+07	High	587,80005	58,765	2,63
GTAKACLTDNRTKREKELQE	Q5U3N0	2348,20	3,33E+07	2,29E+07	2,96E+07	High	587,79993	58,6325	2,74
ILEPTYSGDVKPVQGEIVFAEG	Q6JAN0	2348,20	3,33E+07	2,29E+07	2,96E+07	High	587,80084	58,6592	2,66
AEREIVRDIKE	P83750	1357,74	1,22E+07	2,30E+07	1,76E+07	High	453,25436	22,9282	3,82
EKGSTGHPGTPGEGGPPGPPGSPGSP	C7DZK3	2295,06	1,23E+07	2,33E+07	1,95E+07	High	765,69031	29,6985	2,48
ANREKMTQIMFE	P83750	1497,72	1,98E+07	2,34E+07	2,69E+07	High	499,91263	47,8268	3,95
SERVGLL	Q90339	773,45	1,12E+07	2,42E+07	2,43E+07	High	387,23056	38,9817	2,68
NLDKKQRNFDKVLAE	Q90339	1817,99	1,98E+07	2,45E+07	2,07E+07	High	606,66901	28,9993	4,14
KSYELPDGQVITIG	P83750	1519,80	7,47E+07	2,46E+07	5,37E+07	High	760,40521	92,9196	3,53
RVPTPNVSVVDL	Q5XJ10	1295,73	2,55E+07	2,51E+07	2,48E+07	High	648,37103	72,6512	3,13
TEAPLNPKANREKMTQIM	P83750	2072,06	2,33E+07	2,53E+07	2,62E+07	High	691,36115	43,5218	4,79
DLAGRDLTDY	P83750	1138,54	1,35E+07	2,55E+07	2,14E+07	High	569,77362	57,3942	3,06
NLEVTVK	Q90339	802,47	1,35E+07	2,56E+07	1,93E+07	High	401,73819	28,6907	2,07
YPGIADRM	P83750	922,45	1,70E+07	2,60E+07	2,74E+07	High	461,72745	45,7797	2,33
KTIDDLEDE	P13104	1077,49	1,66E+07	2,62E+07	2,07E+07	High	539,25281	35,6288	2,91
KIEDEQSLGAQ	Q90339	1217,60	1,38E+07	2,64E+07	1,61E+07	High	609,30597	27,2953	4,02
SQRVIDSM	Q90339	935,46	1,30E+07	2,65E+07	2,13E+07	High	468,23578	28,7449	2,5
DLQHRLDE	Q90339	1025,50	1,38E+07	2,67E+07	1,77E+07	High	513,25604	24,3848	2,15
EYDEAGPSIVHR	P53479	1372,65	1,13E+07	2,68E+07	1,68E+07	High	686,8291	33,7159	3,7
ANREKMTQIM	P83750	1221,61	1,36E+07	2,70E+07	2,28E+07	High	407,87579	26,7545	3,2

KGSKTSAASVKKESAIPA	Q5TZ18	1759,99	2,15E+07	2,74E+07	2,88E+07	High	440,75235	28,9272	3,46
TGIQYNLKELLAQGVI	A0A8M9PQ61	1760,00	2,15E+07	2,74E+07	2,88E+07	High	440,75266	28,0731	3,14
IMKDEGPKVLKEEVVE	Q5PZ43	1858,98	4,25E+07	2,76E+07	3,32E+07	High	620,33514	51,6405	2,46
VMDSKIVQVALNGLENI	Q503E9	1858,99	4,25E+07	2,76E+07	3,32E+07	High	620,33521	50,6523	2,97
NDHFVKL	Q5XJ10	872,46	1,31E+07	2,81E+07	1,61E+07	High	436,73618	29,0759	2,19
NAEKGQLVL	Q1LXK5	971,55	1,49E+07	2,83E+07	1,99E+07	High	486,28088	32,9203	1,96
LEEISERL	Q90339	988,53	1,76E+07	2,87E+07	2,49E+07	High	494,77106	49,5221	2,86
LSRELEEI	Q90339	988,53	1,76E+07	2,87E+07	2,49E+07	High	494,7706	48,4159	2,18
TERGYSFVTT	P53479	1160,56	1,59E+07	2,89E+07	2,22E+07	High	580,78418	43,4647	2,87
TIIDQNRDGIIS	O93409	1344,71	1,47E+07	2,89E+07	1,77E+07	High	672,86035	53,7465	3,01
ELPDGQVITIGNE	P83750	1384,70	2,35E+07	2,95E+07	2,57E+07	High	692,85345	80,1293	2,89
ELEEELKTVTN	P13104	1304,66	1,78E+07	2,99E+07	1,82E+07	High	652,83447	54,0682	3,44
KKDIDDLELT	Q90339	1189,63	1,98E+07	3,14E+07	2,01E+07	High	397,21643	41,2084	3,54
VAPEEHPTLL	P53479	1105,59	1,33E+07	3,18E+07	6,96E+07	High	553,30005	47,8113	2,19
RGATALQGVGTISVSPR	A2VD33	1669,93	3,20E+07	3,20E+07	2,88E+07	High	557,31824	57,0025	2,44
LELVEVLSEVRRVE	A3KPW9	1669,95	3,20E+07	3,20E+07	2,88E+07	High	557,31866	56,8885	2,35
SKIEDEQSL	Q90339	1048,52	1,65E+07	3,25E+07	1,94E+07	High	524,76337	28,3835	2,67
DLVMDILRVLTTPDLE	Q66HV4	1858,98	5,33E+07	3,26E+07	3,96E+07	High	465,50391	50,7026	2,7
LEQQVDDLEGSLE	Q90339	1474,69	5,21E+07	3,36E+07	4,02E+07	High	737,85101	76,0422	4,11
KALGTVL	A1A5V7	701,46	2,00E+07	3,46E+07	2,66E+07	High	351,23193	33,6772	2,06
KIQTVI	Q503E9	701,46	2,00E+07	3,46E+07	2,66E+07	High	351,23193	33,6772	1,97
TIIDQNRDGIISKD	O93409	1587,83	2,27E+07	3,62E+07	2,47E+07	High	794,42126	42,4678	4,25
AGDDAPRAVFPS	P83750	1202,58	1,30E+07	3,68E+07	2,74E+07	High	601,79498	45,8074	3,62
KLDKENALDRAEQAE	P13104	1729,87	2,62E+07	3,68E+07	3,39E+07	High	577,29755	31,2024	4,53
LESRLEE	A0A2R8QCI3	875,45	2,28E+07	3,83E+07	3,08E+07	High	438,22852	25,7192	2,24
NPEGKLDMVHIQNAALAGGVAVGTAGEMML	Q7T070	3026,47	2,51E+07	3,88E+07	2,97E+07	High	757,37164	30,1626	2,6
AVGKVIPE	Q5MJ86	812,49	1,78E+07	3,93E+07	2,37E+07	High	406,74857	28,2436	2,22
GEQIDNLQRVK	Q90339	1299,70	1,90E+07	3,98E+07	2,83E+07	High	433,90674	28,6112	3,89
GETGKSIHELE	Q90339	1199,59	2,12E+07	3,99E+07	2,83E+07	High	600,30023	20,7465	3,38
NRRIQLVEEE	P13104	1285,69	3,17E+07	4,03E+07	3,57E+07	High	429,23477	29,0473	2,68
KVIEEAVENVK	Q1L8I0	1257,70	2,05E+07	4,10E+07	3,07E+07	High	629,35754	29,3647	2,15
KVNPDTLETIK	A9C3R9	1257,70	2,05E+07	4,10E+07	3,07E+07	High	629,35803	29,9698	2,02
AANLDKKQRNFDKVL	Q90339	1759,98	3,16E+07	4,14E+07	4,45E+07	High	587,33405	28,14	5,83

GFAGDDAPR	P83750	905,41	2,69E+07	4,15E+07	2,39E+07	High	453,21063	22,4386	2,33
KKMEGDLNEM	Q90339	1194,55	2,44E+07	4,19E+07	4,04E+07	High	398,8555	23,8197	3,33
GQKDSYVGDEAQSK	P83750	1511,70	9,20E+06	4,34E+07	2,61E+07	High	756,35333	15,2446	4,14
AANLDKKQRNFDKVLAE	Q90339	1960,06	3,63E+07	4,37E+07	5,26E+07	High	654,02716	31,9734	5,68
KEFLEE	O93409	794,39	2,70E+07	4,63E+07	3,42E+07	High	397,70157	31,9252	2,04
GFAGDDAPRAVFPSIVGRPRHQ	P83750	2350,22	6,69E+07	4,85E+07	7,44E+07	High	470,85046	55,8175	5,24
KQDSYPQEPGAAGTAGA	F1QBY1	1647,76	2,50E+07	4,95E+07	3,17E+07	High	549,93036	27,7263	2,35
VEERMQGTEGSATVGP	Q2TJA6	1647,76	2,50E+07	4,95E+07	3,17E+07	High	549,93036	27,7263	2,35
EIENQEDLTGTQASL	Q6DRC3	1647,77	2,50E+07	4,95E+07	3,17E+07	High	549,93085	28,3443	2,31
NSRNTVEHDHSPAAI	Q6PCS4	1647,78	2,50E+07	4,95E+07	3,17E+07	High	549,93036	27,7263	2,36
FHALGCTGLEELMGI	Q7SY48	1647,79	2,50E+07	4,95E+07	3,17E+07	High	549,93085	28,3443	2,3
AVGKVIPELNGKLT	Q5MJ86	1438,86	4,13E+07	5,01E+07	4,63E+07	High	719,93732	53,5457	3,58
FAGDDAPRAVFPS	P83750	1349,65	2,84E+07	5,24E+07	5,44E+07	High	675,32941	61,8371	4,24
IVAPGKGIL	Q8JH70	867,57	3,01E+07	5,38E+07	2,48E+07	High	434,28796	49,8278	2,04
TERGYSF	P83750	859,39	2,94E+07	5,56E+07	3,86E+07	High	430,20261	31,0812	2,66
EKTIDDLEDE	P13104	1206,54	3,37E+07	6,18E+07	3,51E+07	High	603,77405	41,0715	2,63
GFAGDDAPRAVFP	P83750	1319,64	4,98E+07	6,27E+07	3,15E+07	High	660,32452	70,6176	4,23
KSYELPDGQVITIGNE	P83750	1762,89	1,88E+08	6,30E+07	1,39E+08	High	881,94977	93,0931	5,14
ADESERGMKVIENR	P13104	1633,80	3,37E+07	6,33E+07	3,61E+07	High	545,27167	27,8591	4,71
GEQIDNLQRVKQKLE	Q90339	1797,98	5,25E+07	6,41E+07	6,27E+07	High	599,99982	43,7977	4,36
SKIEDEQSLGAQ	Q90339	1304,63	3,20E+07	6,75E+07	4,48E+07	High	652,82153	29,3685	3,9
EYDEAGPSIVHRK	P53479	1500,74	3,38E+07	6,88E+07	4,67E+07	High	750,87732	24,5514	3,83
VNGIDLRG	Q5PYH5	843,47	3,43E+07	6,97E+07	4,81E+07	High	422,23917	34,7479	1,97
DNGSGLVKA	P53479	860,45	4,39E+07	7,10E+07	4,92E+07	High	430,7287	23,8454	2,55
AGDTEQQLKKALEDL	Q5TZ80	1658,86	6,70E+07	7,16E+07	5,60E+07	High	553,63013	37,5995	2,49
ESKLSRHSRSLMDI	Q6PCS4	1658,86	6,70E+07	7,16E+07	5,60E+07	High	553,63013	37,5995	2,42
QTRMQQQLQLQQL	Q7ZVN7	1658,86	6,70E+07	7,16E+07	5,60E+07	High	553,62982	38,2427	2,37
KEIARKMGQLQEDL	A1IH00	1658,89	6,70E+07	7,16E+07	5,60E+07	High	553,63	37,1665	2,69
VAPEEHPTL	P53479	992,50	3,15E+07	7,43E+07	2,95E+07	High	496,75797	32,624	2,37
QDLVDKL	Q90339	830,46	4,08E+07	8,98E+07	4,65E+07	High	415,7356	47,0918	2,59
KEFLEEL	O93409	907,48	6,53E+07	9,16E+07	9,10E+07	High	454,24335	60,2451	2,23
GQKDSYVGDEAQSKRGILTL	P83750	2165,12	1,02E+08	1,16E+08	1,26E+08	High	722,37964	52,1494	8,28
EGGSTGRPATADL	Q1L8W0	1231,59	6,13E+07	1,27E+08	1,01E+08	High	616,30048	37,3089	2,11

DLQHRLDEAE	Q90339	1225,58	7,35E+07	1,27E+08	9,87E+07	High	613,29608	34,492	2,94
RVAPEEHPTL	P53479	1148,61	5,56E+07	1,29E+08	8,80E+07	High	383,54147	26,4532	2,99
RVAPEEHPTLL	P53479	1261,69	7,42E+07	1,38E+08	1,92E+08	High	631,34973	37,1644	2,83
GQKDSYVGDEAQSKRGILT	P83750	2052,04	1,09E+08	1,57E+08	1,22E+08	High	684,68512	34,6916	7,62
GQKDSYVGDEAQSKRGIL	P83750	1950,99	1,09E+08	1,58E+08	1,24E+08	High	651,0022	35,2498	7,47
GEKLKGADPEDVIVS	O93409	1556,82	1,04E+08	1,72E+08	1,31E+08	High	519,61127	44,3097	3,69
GFAGDDAPRAVFPS	P83750	1406,67	1,77E+08	3,32E+08	3,09E+08	High	703,84015	65,5667	4,01
DIDIRKDLYAN	P53479	1335,69	3,08E+08	4,68E+08	3,47E+08	High	668,35046	59,7866	3,52
RLDLAGRDLTDYLMKI	P83750	1893,03	6,88E+04	#DIV/0!	8,13E+03	High	631,68066	99,5186	2,39
APSTMKIKIIAPPERKYSVWIGGSIL	P83750	2855,62	2,73E+05	#DIV/0!	9,17E+03	High	571,93207	86,604	2,81
KVEIVAINDPFIDLDYM	Q5XJ10	1995,01	9,74E+04	#DIV/0!	1,25E+04	High	998,01337	115,8235	2,71
ALPHAIMRLDLAGRDLTDYLMK	P53479	2513,34	2,16E+05	#DIV/0!	1,59E+04	High	629,09167	98,1407	2,7
GIISEGNETVEDIAARL	P25489	1786,92	4,97E+04	#DIV/0!	1,78E+04	High	893,9635	106,6453	2,41
DVLNLLDNFLIK	Q6PC29	1416,81	1,85E+05	#DIV/0!	3,37E+04	High	708,91034	120,1516	2,24
RGRKVAEQELVDASERVGL	Q90339	2112,15	9,08E+04	#DIV/0!	3,71E+04	High	528,79376	55,0953	2,79
AIAKGVGIISEGNETVEDIAARL	P25489	2326,26	1,07E+05	#DIV/0!	4,02E+04	High	776,0943	103,1202	3,29
SSLEKSYELPDGQVITIGNERFR	P83750	2638,35	1,32E+05	#DIV/0!	4,59E+04	High	660,34265	80,509	3,89
KYPIEHGIITNWDDMEKIWHH	P53479	2662,29	1,70E+05	#DIV/0!	4,99E+04	High	533,26489	81,2905	2,62
DDLEGSLEQEKKLRMDLE	Q90339	2148,05	1,21E+05	#DIV/0!	5,12E+04	High	716,69086	71,2485	2,81
RLDLAGRDLTDYLMKIL	P83750	2022,11	1,71E+05	#DIV/0!	5,49E+04	High	674,70715	111,7906	2,32
EYKEAFTIIDQNRDGIISKDDLRDVL	O93409	3066,57	2,64E+05	#DIV/0!	6,90E+04	High	767,40076	92,3495	3,97
QTERGRKVAEQELVDASERVGLL	Q90339	2583,38	8,92E+04	#DIV/0!	7,24E+04	High	646,60254	69,8073	2,96
ESLPPPPPDPVASLPPPPAFTSAGGAPPPP	Q6PFT9	2951,52	1,65E+05	#DIV/0!	7,35E+04	High	738,63171	81,2239	2,6
EEAEGTLEHEESKILR	Q90339	1869,92	#DIV/0!	#DIV/0!	7,68E+04	High	623,97925	49,6869	2,58
ASVVPSPAKHRAKMDDIVVVAQGTQSLRN	Q5XIX8	3074,65	8,49E+04	#DIV/0!	7,78E+04	High	440,09961	62,7208	2,61
KMVFGPNSAPKKNRGSSADSAMKVEKKPA	A0A0R4IBK5	3076,60	3,52E+05	#DIV/0!	7,87E+04	High	616,12311	112,8651	2,69
LEQQVDDLEGSLEQEKKLRMDL	Q90339	2616,32	2,20E+05	#DIV/0!	8,58E+04	High	654,83557	82,9847	2,94
EIQLKEAKHIAEEADRKYEEVARKL	P13104	2996,62	5,54E+04	#DIV/0!	8,59E+04	High	500,27643	77,6794	2,76
VQMGVAFTEQIIQTFLSMFTREQL	Q8QHJ8	2833,43	7,79E+04	#DIV/0!	8,97E+04	High	709,11304	58,6914	2,6
RLEDEEEINAELT	Q90339	1560,74	#DIV/0!	#DIV/0!	9,49E+04	High	780,87592	60,1056	1,95
TKLEQQVDDLEGSLEQEKKLR	Q90339	2486,31	2,41E+05	#DIV/0!	9,96E+04	High	622,33282	67,8542	4,7
IIDQNRDGIISKDDLRDVLATM	Q66I73	2501,30	2,03E+05	#DIV/0!	1,04E+05	High	626,08228	92,7526	2,69
SLGLAVGTSTGQILVYDLRSSRPL	Q802W4	2503,39	3,70E+05	#DIV/0!	1,13E+05	High	501,48273	53,8476	2,93

SLNRRIQLVEEELDRAQERLAT	P13104	2639,42	1,80E+05	#DIV/0!	1,16E+05	High	660,61084	83,3492	2,77
EIQTALEEAEGTLEHEESKIL	Q90339	2369,17	2,84E+05	#DIV/0!	1,16E+05	High	790,39703	112,7591	3,21
KSYELPDGQVITIGNERFRCPETLFQ	P53479	3097,54	1,88E+05	#DIV/0!	1,17E+05	High	1033,1803	93,1257	2,77
EMGLGKTCQTISLLAYARGSLKMNGPF	Q7ZU90	2943,49	2,46E+05	#DIV/0!	1,23E+05	High	589,50775	80,7408	3,42
SFTPTLAAHFNENLIKHVQGWPAEHV	Q7ZUK7	2943,49	2,46E+05	#DIV/0!	1,23E+05	High	589,50708	80,55	3,95
AVQAGAATAGTVVTASGPAGRAGTTQWTRATG	A0A0R4IBL7	2943,50	2,46E+05	#DIV/0!	1,23E+05	High	589,50775	80,7408	3,43
STFQQMWITKQEYDEAGPSIVHRK	P53479	2879,41	1,50E+05	#DIV/0!	1,30E+05	High	576,69067	66,9325	2,68
LAGRDLTDYLMKIL	P83750	1621,90	6,92E+05	#DIV/0!	1,35E+05	High	541,30585	114,4809	3,69
APSQTKVDGVEIVVQ	A4IG42	1569,85	8,63E+05	#DIV/0!	1,46E+05	High	785,42877	114,4252	2,08
EYLENPKKYIPGTKMIFAGIK	Q6IQM2	2440,33	1,51E+05	#DIV/0!	1,49E+05	High	610,8399	73,7149	2,78
FRENLGKLMTNL	Q90339	1435,77	1,67E+05	#DIV/0!	1,52E+05	High	479,26431	69,8103	2,85
SLIDRMLLSSSVKTGAAGAEALSLL	Q499B3	2503,38	5,31E+05	#DIV/0!	1,54E+05	High	501,48273	53,8476	3,06
QKQRGSSSRAHSAVTDEGISLQPRDKLH	A1L253	3088,60	6,26E+05	#DIV/0!	1,69E+05	High	515,604	70,7123	2,77
HIKGGAKRVIISAPSADAPMFVM	Q5XJ10	2396,29	2,63E+05	#DIV/0!	1,90E+05	High	599,83014	64,7171	2,77
EGTDKGKIKASHALAKIAAVSNPEIAFP	Q6DGE9	2863,57	7,75E+05	#DIV/0!	1,96E+05	High	573,51825	92,8623	2,72
VQQLAKVMGWHVLSFSNHVGLGSIESI	Q08BY1	2952,54	2,27E+05	#DIV/0!	2,11E+05	High	591,31921	69,1614	3,1
KPSTGQQQLSFQLLTSRDSLTLYKNV	Q8JGS1	2952,58	2,27E+05	#DIV/0!	2,11E+05	High	591,31683	69,1537	2,85
AEGTLEHEESKILR	Q90339	1611,83	#DIV/0!	#DIV/0!	2,47E+05	High	537,95178	36,9946	3,83
KLTGSMTNIQLHTFGLARYTYTVLSCL	A2VD33	3088,60	6,35E+05	#DIV/0!	2,53E+05	High	618,52429	70,8831	3,13
PAYAAINHNILSTSTLTSPAVSLGGFA	Q5U3U3	2673,39	5,79E+05	#DIV/0!	2,55E+05	High	535,48315	64,2329	3,21
QKTVDGPSAKAWRDGRGAHQNIIPA	Q5MJ86	2673,40	5,79E+05	#DIV/0!	2,55E+05	High	535,48242	64,2842	3,36
EQIDNLQRVKQKLEKEKSE	Q90339	2342,27	2,84E+05	#DIV/0!	2,56E+05	High	586,32416	39,0709	3,13
EQQVDDLEGSLEQEKKLRMDLE	Q90339	2632,28	5,77E+05	#DIV/0!	2,59E+05	High	658,82556	77,418	4,02
EPLKPYEEPSGYSSLPLTGESQLQ	B0S6T2	2649,29	8,69E+05	#DIV/0!	2,79E+05	High	883,77466	114,7214	2,43
THRALETDRVIHEQQLGLEWASPPLP	F1QRC1	2993,56	2,15E+05	#DIV/0!	2,80E+05	High	599,52338	68,9334	2,67
KDLLEREKWDLRRQTKEATEHAGAL	A9C3W3	2993,59	2,15E+05	#DIV/0!	2,80E+05	High	599,52338	68,9334	2,81
PVQGGLEKYRGKNMDTVQILLDFMIK	Q6DRJ9	2993,60	2,15E+05	#DIV/0!	2,80E+05	High	599,52338	68,9334	2,7
LKMVEKDPNGGRRLTPQGTRDLDRIAG	Q90YQ4	2993,61	2,15E+05	#DIV/0!	2,80E+05	High	599,52338	68,9334	2,71
APLNPKANREKMTQIMFETF	P83750	2366,20	7,06E+05	#DIV/0!	2,83E+05	High	789,40717	75,956	2,47
GLPKMNTSIMANVKKAFIGENRDLVDP	Q5SPC5	2974,55	9,36E+05	#DIV/0!	2,91E+05	High	496,59753	60,0174	3,66
ATVLKQGSNQAIRFYVMTALRNWYK	F1R4U0	2974,57	9,36E+05	#DIV/0!	2,91E+05	High	496,59753	60,0174	3,63
GHSERRHVFGESDELIGQKVAHAL	Q90XG0	2672,37	3,42E+05	#DIV/0!	2,97E+05	High	668,84772	49,2846	3,85
KLKYKAISEELDHALNDMTSI	P13104	2419,25	6,01E+05	#DIV/0!	3,01E+05	High	605,57086	89,7138	4,73

LSKIEDEQSLGAQLQKKIKELQARIE	Q90339	2995,68	3,00E+05	#DIV/0!	3,13E+05	High	599,94354	90,7782	4,49
QSASFSPSLKSLPLSSSVHAATATLTSL	F1QLG5	2788,47	1,61E+06	#DIV/0!	3,14E+05	High	930,16901	113,2126	2,62
KSASTIEASPGVIANPFAAGIVRKNSMESI	Q4H4B6	3045,60	1,59E+06	#DIV/0!	3,33E+05	High	762,16217	107,4835	2,84
KEAFTIIDQNRDGIISKDDLRDVLATM	Q66I73	3077,59	1,18E+06	#DIV/0!	3,34E+05	High	616,32764	100,0423	4,72
NNRFASFIDKVRF	Q6NWF6	1613,85	2,59E+05	#DIV/0!	3,42E+05	High	538,62543	75,3944	3,54
TKAKTKLEQQVDDLEGSLEQEKKLR	Q90339	2914,58	3,74E+05	#DIV/0!	3,51E+05	High	583,72327	62,5289	5,18
KIEDEQSLGAQLQKKIKELQARIE	Q90339	2795,56	3,80E+05	#DIV/0!	3,59E+05	High	559,91919	89,1393	4,47
EIQLKEAKHIAEE	P13104	1537,82	1,70E+05	#DIV/0!	3,86E+05	High	513,28021	32,2297	2,31
EKGAVQVADGLTLPQFILKEE	O93430	2285,24	5,96E+05	#DIV/0!	4,05E+05	High	572,06903	71,6706	2,98
GLELVGTGSSVLRDERLASAVR	A3KPN8	2285,26	5,96E+05	#DIV/0!	4,05E+05	High	572,06903	71,6706	2,86
TIRNKLVRLMTHVDMAVKQ	Q6DRJ9	2285,26	5,96E+05	#DIV/0!	4,05E+05	High	572,0694	71,7424	2,62
EEIIKVELVSATSGGNIGLEKV	Q6JAN0	2285,26	5,96E+05	#DIV/0!	4,05E+05	High	572,06879	71,5387	2,98
DIDIRKDLYANNVLSGGTTMYPGIADRM	P53479	3099,52	1,51E+06	#DIV/0!	4,29E+05	High	775,63812	102,0758	5,67
LAVSQVESVQQAANTPIANNLLQ	Q90WY5	2408,28	6,17E+05	#DIV/0!	4,78E+05	High	602,82275	71,619	2,76
TLDDLQAEED	Q90339	1148,50	3,46E+05	#DIV/0!	4,83E+05	High	574,75323	50,5248	2,19
LGSIAVIL	F8W3X3	785,51	7,04E+04	#DIV/0!	5,25E+05	High	393,26144	65,2654	2,2
KALMKKDNPVGKYPLAAGNLLASAVAQGQ	Q9DDD7	2969,62	2,42E+06	#DIV/0!	5,61E+05	High	594,73376	102,2117	2,71
ILTLMAFQNRLVQLGEERLNSGILGAI	A5WUT8	2969,66	2,42E+06	#DIV/0!	5,61E+05	High	594,73352	102,2111	2,62
LEEISERLE	Q90339	1117,57	#DIV/0!	#DIV/0!	5,81E+05	High	559,29169	47,6357	2,72
AGAERIFQNNGVPLLMQLIDTGKPEMIL	Q6DGE9	3084,62	1,34E+06	#DIV/0!	6,08E+05	High	617,73041	80,5456	2,75
AKVEEISGVAFIFNQRFFQDLRQATGI	F1QWK4	3084,63	1,34E+06	#DIV/0!	6,08E+05	High	617,72943	81,3063	3,69
LSKIEDEQSLGAQLQKKIKEL	Q90339	2398,36	3,86E+05	#DIV/0!	6,97E+05	High	600,3465	73,8755	3,87
ERTQAHVLSLNKDI	P0CK98	1623,88	8,69E+05	#DIV/0!	7,07E+05	High	541,96704	27,0538	2,45
KFTKESTSLLDELI	Q7SX85-2	1623,88	8,69E+05	#DIV/0!	7,07E+05	High	541,96704	27,0538	2,44
GSLEQEKKLRMDLERAKRKLE	Q90339	2557,42	7,61E+05	#DIV/0!	7,60E+05	High	512,29211	45,4906	2,63
KQRADLSRELEEISERL	Q90339	2072,11	9,79E+05	#DIV/0!	8,24E+05	High	518,7843	77,1918	3,44
RGRKVAEQELVDASERVGLL	Q90339	2225,24	1,07E+06	#DIV/0!	8,30E+05	High	557,06616	64,347	5,31
TATRSNIDAMFE	Q6NWF6	1371,62	7,25E+05	#DIV/0!	8,47E+05	High	686,31647	56,0622	1,91
TKLEQQVDDLEGSLEQEKKLRMDLE	Q90339	2974,50	1,97E+06	#DIV/0!	8,68E+05	High	744,3822	86,8596	5,37
GSRGLGHQVATDALVAMEKAMKRDRITV	Q6NZS4	3042,59	1,88E+06	#DIV/0!	9,32E+05	High	507,94147	71,0145	3,38
VLDNAIRICQAMLDVVANEGWLVSALS	E7F8F4	2957,52	3,43E+06	#DIV/0!	9,62E+05	High	592,30994	78,3812	2,69
LNYPPHSVSPTDINASLPPM	A0A0R4IBL7	2150,06	8,53E+05	#DIV/0!	1,02E+06	High	717,36041	41,4275	2,43
VGDSLLEPFWPMGTGIARGF	F1QWK4	2150,07	8,53E+05	#DIV/0!	1,02E+06	High	717,36041	41,4275	2,42

KIWHHTFYNEL	P83750	1487,74	1,77E+06	#DIV/0!	1,04E+06	High	496,58636	44,8195	3,43
DGSGLVQIPVSMYQTVVTSLAQGNRPVQ	Q90X44	2944,52	3,53E+06	#DIV/0!	1,11E+06	High	589,71448	79,8496	2,81
VFTFLFWHLEDLKGTTTLFGICSVL	B0UYT5	2944,53	3,53E+06	#DIV/0!	1,11E+06	High	589,71301	80,761	2,65
NTSVESLIEKLAESRGTGKERPGPIEF	Q502I9	2944,54	3,53E+06	#DIV/0!	1,11E+06	High	589,71356	79,9889	3,17
AANLDKKQRNFDKVLAEWKQKYEE	Q90339	2951,54	1,82E+06	#DIV/0!	1,44E+06	High	738,64069	58,9311	5,98
SKIEDEQSLGAQLQKKIKELQARIE	Q90339	2882,59	1,78E+06	#DIV/0!	1,63E+06	High	721,40588	88,393	5,55
SLNRRIQLVEEELDRAQE	P13104	2198,15	2,11E+06	#DIV/0!	1,80E+06	High	550,29517	70,7671	4,68
PQMVLAQNQISQPPNSAVVTPGLMPPVQ	A4QP16	2957,52	7,59E+06	#DIV/0!	2,13E+06	High	493,75992	78,1333	3,74
ESVGLTLVNRDLTSLQLKTPAGQILT	F1Q4S1	2767,56	4,18E+06	#DIV/0!	3,04E+06	High	554,31934	69,3975	2,7
SLPAGPASSTQPIQLSDLQSILATMN	Q6NZ09	2656,35	6,81E+06	#DIV/0!	3,26E+06	High	532,07745	74,2899	3,05
MKKDIQDKTTKVEEAQLHNAALE	P0CK98	2656,36	6,81E+06	#DIV/0!	3,26E+06	High	532,07745	74,2899	3,03
KEDTTVPTPAPTPEPSLKDPPSPVR	H6D7E6	2656,38	6,81E+06	#DIV/0!	3,26E+06	High	532,07739	74,7314	2,68
GQVITIGNE	P83750	930,49	5,47E+06	#DIV/0!	3,33E+06	High	465,74997	51,5905	2,29
WASPPLPFMERSGAVQ	F1QRC1	1788,87	3,09E+06	#DIV/0!	5,08E+06	High	894,94177	57,6182	2,03
LSTIQAADLICVMSNGRIVEAGTHLEL	Q56A55	2927,50	3,32E+07	#DIV/0!	1,24E+07	High	732,63373	91,0955	2,83
SAIHVRASVPMRFPPPFPERLSPNNL	A2RRV3	2929,56	1,19E+05	#DIV/0!	#DIV/0!	High	586,71777	83,363	2,64
NLRRQLDSLGNDKMKLEADLHNMQGL	Q6NWF6	3009,54	2,08E+05	#DIV/0!	#DIV/0!	High	753,14124	81,5154	3,03
WADLSPGSGPVKKHGKTIMGAV	Q90487	2236,19	1,03E+06	#DIV/0!	#DIV/0!	High	559,80359	44,7693	3,25
KTVNELQNLTSAEVVVP	Q9PW80	1841,00	2,04E+06	#DIV/0!	#DIV/0!	High	614,3338	54,5981	2,75
AVIDQLGGDLNSTPLHWAIRQGHLSMV	A0A0R4IQZ2	2944,51	3,14E+06	#DIV/0!	#DIV/0!	High	736,88995	79,9113	3,41
MKRDPLNISQSGGEFPLILGRDVSGEI	Q7T3C7	2944,52	3,14E+06	#DIV/0!	#DIV/0!	High	736,8894	80,0446	2,81
VRGMTELDRSAFNQ	B8A5G9	1639,79	#DIV/0!	#DIV/0!	#DIV/0!	High	820,39893	81,0372	1,99
KLTEQERQGLSAAVNE	P0CK98	1772,91	#DIV/0!	#DIV/0!	#DIV/0!	High	591,64362	36,1144	2,4
TTASAAAAAAAAAAAAAAAAAAAGVS	Q7SYL3	1972,01	#DIV/0!	#DIV/0!	#DIV/0!	High	493,75882	42,2489	2,61
IMEAQLLQELERQRQA	Q6P5L7	1972,03	#DIV/0!	#DIV/0!	#DIV/0!	High	493,75882	42,2489	2,66
KYDGLDANGTGLLNQEEQF	G9G127	2111,99	#DIV/0!	#DIV/0!	#DIV/0!	High	704,66992	34,0627	2,37
YLAQQKSVMSSPVALTASSASP	A5PLF5	2239,13	#DIV/0!	#DIV/0!	#DIV/0!	High	560,53473	31,8977	2,91
KLGMLQPMAAMTFLNKDSNI	A2VCZ5	2239,13	#DIV/0!	#DIV/0!	#DIV/0!	High	560,53473	31,8977	2,87
FPRDGGEKYEKYFQHSSSLF	A8CVX7	2422,15	#DIV/0!	#DIV/0!	#DIV/0!	High	808,05225	52,7794	2,62
KDDLRDVLATMGQLNVKNEELE	Q66I73	2546,28	#DIV/0!	#DIV/0!	#DIV/0!	High	637,32831	36,8737	2,71
AEISAVPWTVHVVDSPTMNAFVLP	E9QBI7	2580,32	#DIV/0!	#DIV/0!	#DIV/0!	High	645,83099	55,5733	2,7
TVVAHDHGQTSLSASAYIVIYLSPD	F8W3X3	2644,33	#DIV/0!	#DIV/0!	#DIV/0!	High	529,66821	63,4238	2,68
EQTERGRKVAEQELVDASERVGLL	Q90339	2712,43	#DIV/0!	#DIV/0!	#DIV/0!	High	678,86395	75,0605	2,72

VSGLPKTMTQKELEQLFSQYGRIITS	A0A0R4IEW8	2954,57	#DIV/0!	#DIV/0!	#DIV/0!	High	591,71869	69,1354	2,62
KSGLSLSDLQVEYKDLTGELIPYKQLG	E7FDW8	2994,60	#DIV/0!	#DIV/0!	#DIV/0!	High	599,72534	68,719	2,72
KKAFEIIDEDKSGFIEEEELKLFLQ	P05939	2998,57	#DIV/0!	#DIV/0!	#DIV/0!	High	750,4024	102,2336	3,29
KLRKTASLESSQWRRASSPMDEVLASL	Q6NVC9	3046,61	#DIV/0!	#DIV/0!	#DIV/0!	High	610,13257	107,5298	2,64

Table S5. Peptides Sequences identified in SDG HH ctrl, SDG HH40 and SDG HH80.

Sequence	Master Protein Accessions	Theo. MH+ [Da]	SDG HH ctrl	SDG HH40	SDG HH80	Confidence (by Search Engine): Sequest HT	m/z [Da] (by Search Engine): Sequest HT	RT [min] (by Search Engine): Sequest HT	XCorr (by Search Engine): Sequest HT
ELTYQTEEDKK	Q90339	1383,66382	6,42E+04	1,89E+04	3,07E+04	High	692,33643	20,9965	2,19
KLQQFFNH	Q90339	1061,55269	1,18E+05	1,20E+05	4,50E+04	High	531,28149	36,6295	1,98
IEELEEEIEAER	Q90339	1488,70641	3,31E+04	2,67E+04	4,94E+04	High	744,85840	66,1651	2,07
EAFSLFDKDGDGTITTK	Q6PI52	1844,89125	3,03E+05	1,67E+04	5,42E+04	High	615,63660	69,6232	3,18
RVGLLHSQNTSL	Q90339	1324,73317	4,01E+05	5,85E+04	5,73E+04	High	442,25021	36,9180	4,07
AEGTLEHEESKI	Q90339	1342,64850	1,13E+05	3,74E+04	5,78E+04	High	671,82977	30,2337	2,57
MDLENEKQQSDEKIK	Q90339	1834,88512	1,59E+05	#DIV/0!	5,91E+04	High	612,30121	28,2235	2,38
RKLEGDLKL	Q90339	1071,65207	1,54E+06	5,89E+05	6,20E+04	High	357,88977	29,2550	3,11
SDHHVYLEGTL	P53448	1270,60624	2,21E+05	9,92E+04	6,28E+04	High	635,80743	46,8190	2,33
ALEEAEGTLEHEE	Q90339	1456,64381	9,95E+04	6,10E+04	6,31E+04	High	728,82635	48,3307	2,65
TIIDQNRDGIISKD	Q66I73	1587,83368	1,31E+05	6,83E+04	6,39E+04	High	794,42188	43,3534	2,47
RVFDKEGNGTV	Q6P0G6	1221,62223	3,12E+05	1,92E+05	6,42E+04	High	407,88046	23,2706	2,70
GWLDKNKDPLND	Q90339	1414,69612	3,40E+05	9,46E+04	6,49E+04	High	707,85321	54,6634	2,59
DNEFGYSNR	Q5XJ10	1101,45958	1,61E+05	1,49E+05	6,80E+04	High	551,23517	33,5481	2,10
LTDYLMK	P83750	883,45937	7,62E+04	4,09E+04	6,84E+04	High	442,23407	47,0839	1,91
PDVAGNVDYK	Q66I73	1077,52111	1,01E+05	8,74E+04	7,02E+04	High	539,26575	30,7780	2,11
EEAEGTLEHEESKI	Q90339	1600,73369	1,85E+05	2,54E+04	8,11E+04	High	800,87213	42,6892	2,87
IPSAVGYQPTL	Q9PTY0	1145,62010	8,95E+04	4,65E+04	8,28E+04	High	573,31500	76,1528	1,90
ISDLTEQLGETGK	Q90339	1390,70602	1,30E+05	4,21E+04	8,68E+04	High	695,85791	50,9945	2,64
ILPDGDHDLKR	P53448	1278,68008	2,17E+05	1,53E+05	9,21E+04	High	426,89935	24,6921	2,42
LSGGTTMYPGIADR	P83750	1438,69949	1,58E+05	7,45E+04	9,71E+04	High	719,85400	53,1679	2,21
KQEYDESGPSIVHR	P83750	1644,79762	1,81E+05	1,27E+05	1,07E+05	High	548,93884	27,0255	4,06
TTMYPGIADR	P83750	1124,54047	1,25E+05	9,33E+04	1,07E+05	High	562,77490	44,0964	1,97
YVGDEAQSKR	P83750	1152,56438	1,57E+05	7,34E+04	1,09E+05	High	576,78778	18,8473	2,16
VDDLEGSLEQEK	Q90339	1361,64308	1,46E+05	4,70E+04	1,10E+05	High	681,32776	48,2545	2,93
LTRPASY	Q5MJ86	807,43593	1,80E+05	8,31E+04	1,14E+05	High	404,22244	23,2552	1,93
IGGIGTVPVGR	Q92005	1025,61020	1,48E+05	1,01E+05	1,18E+05	High	513,30988	48,1628	2,22
HVFGESDEL	Q1MTI4	1032,46327	1,70E+05	5,36E+04	1,22E+05	High	516,73657	49,2669	2,25
GVQVDVDAPK	Q7ZTS4	1027,54185	1,28E+05	1,22E+05	1,24E+05	High	514,27686	34,6854	2,31

KDDLRDVLA	Q66I73	1044,56840	2,15E+05	6,04E+04	1,28E+05	High	522,78931	43,5079	1,98
FDKEGNGTVM	Q6P0G6	1097,49319	2,62E+05	2,42E+05	1,34E+05	High	549,25067	30,2805	2,23
KNLEVTVK	Q90339	930,56186	2,61E+04	1,57E+05	1,38E+05	High	465,78574	17,9217	2,01
KLDKENALDRAEQAE	P13104	1729,87152	7,63E+05	#DIV/0!	1,39E+05	High	577,29639	31,9754	3,41
DGTEGLVR	Q9PTY0	846,43157	2,08E+05	2,72E+05	1,42E+05	High	423,72021	25,9518	2,22
ELGTVMR	Q6PI52	805,42365	3,08E+05	#DIV/0!	1,42E+05	High	403,21649	28,8927	2,12
ALEEAEGTLEHE	Q90339	1327,60122	1,75E+05	1,46E+05	1,43E+05	High	664,30573	44,6419	2,68
LEEISERL	Q90339	988,53095	1,10E+05	7,06E+04	1,43E+05	High	494,77029	50,2288	2,09
KKDIDDLEL	Q90339	1088,58338	2,96E+05	7,05E+04	1,47E+05	High	544,79651	45,5185	2,37
NVLSGGTTMYPGIADR	P53479	1667,80575	2,53E+05	1,42E+05	1,47E+05	High	834,40765	59,2441	2,74
GIHETAYN	P53479	904,41592	3,94E+04	3,66E+05	1,47E+05	High	452,71283	18,7386	1,90
DSGDGVTHTVPIYEGY	P83750	1709,76532	1,55E+05	1,07E+05	1,49E+05	High	855,38678	72,9909	2,21
KLEQQVDDLE	Q90339	1216,60557	2,30E+05	1,92E+05	1,51E+05	High	608,80774	40,5937	2,07
KGQTVPQVN	Q90339	970,53162	1,02E+05	3,50E+05	1,51E+05	High	485,77017	19,8485	2,20
KDDLRDVL	Q66I73	973,53129	5,27E+05	1,61E+05	1,53E+05	High	487,27029	44,0894	2,12
DKENALDRAE	P13104	1160,55421	1,64E+05	2,15E+05	1,62E+05	High	580,78149	17,8200	2,12
TMYPGIADRM	P83750	1154,53328	1,54E+05	7,53E+04	1,64E+05	High	577,77167	59,3663	2,05
FDKDGDGTITTK	Q6PI52	1297,62704	3,87E+05	1,31E+05	1,72E+05	High	649,31927	21,2024	2,30
VIGEHGDSSVPVWSGV	Q9W7K5	1624,79656	2,61E+05	1,89E+05	1,74E+05	High	812,90356	74,0435	2,59
LEGEEDRL	Q6NWF6	960,46327	2,44E+05	2,39E+05	1,77E+05	High	480,73627	31,5876	2,14
KQEYDEAGPSIVHRK	P53479	1756,89767	1,19E+06	5,81E+04	1,81E+05	High	439,98090	21,2887	4,48
RIPSAVGYQPT	Q9PTY0	1188,63715	1,63E+05	1,65E+05	2,05E+05	High	594,82428	41,3481	2,14
NALDRAEQA	P13104	987,48540	1,82E+05	9,98E+04	2,06E+05	High	494,24850	25,4382	2,14
NKYEDEINKR	Q6NWF6	1308,65425	9,12E+05	1,57E+05	2,07E+05	High	436,89072	19,9017	2,97
RLDIAGR	P83750	800,47371	9,13E+04	3,75E+05	2,09E+05	High	400,74179	20,0300	2,32
FDKDGDGTITT	Q6PI52	1169,53207	4,08E+05	#DIV/0!	2,09E+05	High	585,27094	31,4331	2,03
ADLHNMQGL	Q6NWF6	998,47239	2,49E+05	3,11E+05	2,27E+05	High	499,74179	45,7188	2,06
GTTMYPGIADRM	P83750	1312,60242	2,48E+05	1,10E+05	2,27E+05	High	656,80609	61,9587	2,02
EAEGTLEHEESK	Q90339	1358,60703	4,85E+05	2,81E+05	2,37E+05	High	679,80841	15,5276	2,87
EKSYELPDGQ	P83750	1165,53716	4,04E+05	7,01E+04	2,41E+05	High	583,27301	36,8365	2,26
SMFEQSQIQEYK	Q66I73	1517,69407	3,92E+05	1,18E+05	2,43E+05	High	759,35284	56,9466	2,68
DHALNDMTSI	P13104	1116,49900	3,40E+05	2,71E+05	2,48E+05	High	558,75446	57,5106	2,31
LSKIEDEQSLGA	Q90339	1289,65834	2,22E+05	5,35E+04	2,48E+05	High	645,33319	40,7068	2,20

RLQGEVEDL	Q90339	1058,54766	3,96E+05	3,51E+05	2,49E+05	High	529,77911	43,1325	2,19
VETGVLKPGM	Q92005	1030,56014	3,23E+05	1,29E+05	2,51E+05	High	515,78516	42,9821	2,13
KTEIADL	Q6NWF6	789,43526	2,11E+05	3,18E+05	2,54E+05	High	395,22220	35,6094	1,96
EYDESGPSIVH	P83750	1232,54297	2,55E+05	2,85E+05	2,56E+05	High	616,77551	39,8832	2,36
KVIDDTNL	Q7ZTS4	917,49384	3,21E+05	3,48E+05	2,63E+05	High	459,25165	35,7819	1,99
IPELNGK	Q5MJ86	770,44068	4,55E+05	6,26E+05	2,64E+05	High	385,72498	27,9256	1,98
KDSYVGDEAQSKR	P83750	1482,71831	1,10E+05	1,90E+05	2,70E+05	High	494,91187	12,8877	3,06
DLEESTLQHE	Q90339	1200,53789	2,44E+05	1,34E+05	2,85E+05	High	600,77386	41,6368	2,08
ERLEDEEE	Q90339	1048,44292	4,45E+05	5,34E+05	2,86E+05	High	524,72601	18,9573	2,12
TAYNSIMK	P53479	927,46043	4,40E+05	2,31E+05	2,87E+05	High	464,23468	31,7795	1,93
MDLENEKQQSDEK	Q90339	1593,70609	4,81E+05	2,79E+05	2,89E+05	High	531,90790	20,5384	2,82
KIEDEQSLG	Q90339	1018,50513	3,40E+05	4,76E+05	2,91E+05	High	509,75754	25,1793	2,09
PPDVAGNVDYK	Q66I73	1174,57388	7,93E+05	7,34E+05	3,06E+05	High	587,79187	36,0955	3,64
VIGEHGDSSVPVWSGVN	Q9W7K5	1738,83949	4,16E+05	2,88E+05	3,08E+05	High	869,92505	67,5510	2,19
DEAGPSIVH	P53479	924,44214	3,33E+05	5,33E+05	3,09E+05	High	462,72589	30,3118	1,93
LHVDPDNFK	Q90485	1084,54218	4,56E+05	1,60E+05	3,18E+05	High	542,77618	36,9579	2,36
MLVVYPQTK	P82315	1078,59653	2,67E+05	1,30E+05	3,18E+05	High	539,80292	49,4130	1,93
EAEGTLEHEE	Q90339	1143,48004	3,96E+05	5,77E+05	3,24E+05	High	572,24414	21,3879	2,50
DVAGNVDYK	Q66I73	980,46835	9,45E+05	1,61E+06	3,25E+05	High	490,73917	27,6851	2,45
KLEQQVDDL	Q90339	1087,56298	6,28E+05	4,15E+05	3,28E+05	High	544,28607	40,2860	2,59
GAHQNIIPA	Q5MJ86	920,49484	3,19E+05	2,72E+05	3,38E+05	High	460,75262	30,8331	2,16
FRENLGKL	Q90339	976,55744	1,45E+06	7,07E+05	3,39E+05	High	488,78381	35,0716	2,08
GTTMYPGIADR	P83750	1181,56193	3,61E+05	3,10E+05	3,45E+05	High	591,28595	44,8653	2,49
NSEVAQWR	Q90339	989,47992	#DIV/0!	#DIV/0!	3,49E+05	High	495,24530	30,2842	2,31
GIHETAYNSIM	P53479	1235,57250	4,92E+05	2,37E+05	3,56E+05	High	618,29095	52,0370	2,72
LEEAEGTLEHEE	Q90339	1385,60669	2,95E+05	4,06E+05	3,58E+05	High	693,30798	37,3452	3,00
RVIISAPSAD	Q5XJ10	1028,57348	5,79E+05	5,08E+05	3,62E+05	High	514,79181	38,4981	2,61
GGTTMYPGIADRM	P83750	1369,62388	5,58E+05	2,17E+05	3,79E+05	High	685,31720	61,9216	2,61
KVAEQELVD	Q90339	1030,54152	6,01E+05	1,37E+05	3,79E+05	High	515,77539	29,7238	2,46
SQDESIAKL	Q90339	990,51022	3,48E+05	4,16E+05	3,82E+05	High	495,75977	36,9209	2,43
LEEAEGTLEHE	Q90339	1256,56410	4,25E+05	3,67E+05	3,87E+05	High	628,78729	34,6097	2,25
AQYEDIANR	Q6NWF6	1079,51161	2,62E+05	4,04E+05	3,87E+05	High	540,26080	29,9987	2,07
KLHVDPDNFK	Q90485	1212,63715	1,44E+06	4,08E+05	3,93E+05	High	404,88492	27,7519	2,77

SGGTTMYPGIADR	P83750	1341,61034	3,89E+05	4,66E+05	4,14E+05	High	671,30957	44,9796	1,99
VGLLHSQNTSL	Q90339	1168,63206	8,45E+05	1,08E+05	4,14E+05	High	584,82117	49,8368	2,78
SEVAQWR	Q90339	875,43699	2,97E+05	4,81E+05	4,31E+05	High	438,22351	28,2959	1,93
YEDIANRS	Q6NWF6	967,44795	4,87E+05	4,08E+05	4,34E+05	High	484,22922	21,6570	2,22
ELINNVAK	Q9PTY0	900,51491	4,61E+05	6,91E+05	4,36E+05	High	450,76233	29,5870	2,23
IGMESAGIHETAY	P53479	1378,63074	1,07E+06	1,33E+05	4,70E+05	High	689,82007	54,9945	3,31
MYPGIADRM	P83750	1069,48051	2,89E+05	2,87E+05	4,75E+05	High	535,24518	48,2035	1,94
LEEAEGTLEHEESK	Q90339	1600,73369	5,66E+05	2,62E+05	4,77E+05	High	534,25073	29,4822	4,22
EKLHVDPDNF	Q90485	1213,58478	8,97E+05	4,94E+05	4,88E+05	High	607,29730	43,7797	2,65
YDEAGPSIVHRK	P53479	1371,70154	1,07E+06	#DIV/0!	5,03E+05	High	457,90414	21,9565	2,43
ETAYNSIMK	P53479	1056,50302	7,61E+05	4,82E+05	5,04E+05	High	528,75665	37,6459	2,24
LNDHFVK	Q5XJ10	872,46248	4,58E+05	1,34E+06	5,08E+05	High	436,73590	20,4666	2,00
GASQNIIPASTGAAK	Q5XJ10	1385,73832	6,13E+05	1,57E+05	5,09E+05	High	693,37323	36,3161	3,42
HQGVMVGM	P83750	874,39097	7,68E+05	6,02E+05	5,15E+05	High	437,70004	27,7556	2,01
HALNDMTSI	P13104	1001,47206	2,83E+05	#DIV/0!	5,22E+05	High	501,24094	48,1000	2,35
RIATAI	P13104	644,40899	6,04E+04	3,63E+05	5,29E+05	High	322,70868	26,5664	2,13
GGTTMYPGIADR	P83750	1238,58340	6,91E+05	5,61E+05	5,61E+05	High	619,79669	44,9322	2,27
IGMESAGIHET	P53479	1144,53030	1,35E+06	4,04E+05	5,73E+05	High	572,77002	40,1676	2,57
LGEQIDNLQR	Q90339	1185,62223	4,78E+05	5,34E+05	5,82E+05	High	593,31537	43,9597	2,70
FEQSQIQE	Q66I73	1008,46327	1,09E+06	2,31E+05	6,17E+05	High	504,73642	36,6862	2,07
GFAGDDAPRA	P83750	976,44828	1,01E+06	2,61E+05	6,24E+05	High	488,72873	28,4814	2,38
EAGPSIVHR	P53479	965,51630	4,69E+05	1,53E+06	6,44E+05	High	483,26276	19,3288	2,10
TKLEQQVDD	Q90339	1075,52659	8,52E+05	5,50E+05	6,53E+05	High	538,26837	20,6975	2,42
HVDPDNFK	Q90485	971,45812	4,90E+05	6,15E+05	6,58E+05	High	486,23376	19,6576	3,09
GQKDSYVGDEAQSKR	P83750	1667,79835	1,50E+06	1,39E+05	6,59E+05	High	556,60498	13,9289	4,56
RDLEESTL	Q90339	962,47892	9,57E+05	1,01E+06	6,74E+05	High	481,74448	35,2759	2,32
KTEIADLN	Q6NWF6	903,47819	5,39E+05	9,77E+05	6,80E+05	High	452,24365	28,8352	2,34
RVPTPNVSVV	Q5XJ10	1067,62077	6,59E+05	6,62E+05	7,31E+05	High	534,31512	50,2273	1,99
DEAGPSIVHR	P53479	1080,54325	1,05E+06	1,13E+06	7,47E+05	High	540,77606	24,4366	2,86
KNLEVTV	Q90339	802,46689	1,26E+06	1,87E+06	7,61E+05	High	401,73779	34,5416	2,29
EAFTIIDQNR	Q66I73	1206,61133	1,43E+06	3,49E+05	7,66E+05	High	603,81042	56,0343	2,78
EQQVDDLEGSLE	Q90339	1361,60669	9,74E+05	6,13E+05	7,80E+05	High	681,30792	67,3731	2,22
IGMESAGIHE	P53479	1043,48262	1,38E+06	3,07E+05	8,28E+05	High	522,24579	41,9591	2,46

FTIIDQNR	Q66I73	1006,53162	9,50E+05	5,96E+05	8,35E+05	High	503,77072	44,4732	2,30
APIHAEAPE	Q9PTY0	934,46287	7,53E+05	1,33E+06	9,05E+05	High	467,73627	22,9394	2,21
LGEQIDNLQ	Q90339	1029,52111	6,51E+05	1,06E+06	9,38E+05	High	515,26587	55,7363	2,06
LSKIEDEQSL	Q90339	1161,59976	1,03E+06	3,55E+05	9,65E+05	High	581,30457	39,5798	3,78
SAGIHETAY	P53479	948,44214	1,52E+06	1,28E+06	9,65E+05	High	474,72601	25,0994	2,18
LSKIEDEQSLGAQ	Q90339	1417,71691	1,03E+06	1,88E+05	1,01E+06	High	709,36279	38,5699	2,71
PDGDHDLK	P53448	896,41084	1,50E+06	1,67E+06	1,02E+06	High	448,71030	29,7329	2,45
NALDRAEQAE	P13104	1116,52799	8,50E+05	5,28E+05	1,03E+06	High	558,76910	30,2212	3,55
GPNFSTTVGTSLQYSSSTYPSAKT	P26632	2481,17799	1,27E+06	2,94E+05	1,07E+06	High	827,72943	92,3242	2,50
FPSIVGRPR	P83750	1028,59997	3,68E+06	1,32E+06	1,07E+06	High	343,53925	39,2935	2,81
EPVWAIGTGK	Q1MTI4	1057,56767	1,23E+06	1,11E+06	1,13E+06	High	529,28912	57,8055	2,70
KDIDDL	Q90339	718,36176	1,19E+06	2,20E+06	1,15E+06	High	359,68488	29,5133	2,04
RVQLELN	Q90339	871,49959	7,12E+05	1,03E+06	1,19E+06	High	436,25458	37,7456	2,27
IIDQDKSGFIE	P02618	1264,64196	1,59E+06	4,25E+05	1,19E+06	High	632,82581	50,2718	2,04
GLNSADMLK	Q90339	948,48189	7,39E+05	1,07E+06	1,19E+06	High	474,74530	39,6889	2,12
VITIGNER	P83750	901,51016	2,47E+06	6,18E+05	1,22E+06	High	451,25967	29,1862	2,03
KQEYDEAGPSIVH	P53479	1472,70160	1,35E+06	1,24E+06	1,23E+06	High	491,57324	33,8141	4,01
GEQIDNLQR	Q90339	1072,53816	1,46E+06	1,76E+06	1,25E+06	High	536,77356	27,7850	2,48
KSYELPDGQVITIGNE	P83750	1762,88577	1,26E+06	1,59E+06	1,27E+06	High	881,94836	93,8628	4,59
SKIEDEQSLG	Q90339	1105,53716	1,05E+06	1,33E+06	1,29E+06	High	553,27374	26,8011	2,18
SYVGDEAQSKR	P83750	1239,59641	1,14E+06	9,11E+05	1,30E+06	High	413,87112	15,2475	2,51
YEDIANR	Q6NWF6	880,41592	1,20E+06	2,69E+06	1,30E+06	High	440,71289	21,0005	1,90
DLTEQLGETGK	Q90339	1190,58992	1,52E+06	1,24E+06	1,31E+06	High	595,79938	45,9859	2,68
APEEHPTL	P53479	893,43632	3,62E+06	7,03E+06	1,34E+06	High	447,22281	27,2274	2,10
RVAPEEHPVL	P83750	1146,62658	6,16E+05	6,59E+05	1,35E+06	High	382,88144	30,0578	2,71
LTEQLGETGK	Q90339	1075,56298	1,74E+06	1,05E+06	1,38E+06	High	538,28625	26,8293	2,61
DKENALDRAEQAE	P13104	1488,69249	1,99E+06	7,44E+05	1,41E+06	High	744,85107	32,3358	2,95
RLDIAGRD	P83750	915,50065	2,52E+06	2,56E+06	1,41E+06	High	458,25473	21,5756	2,62
EEAEGTLEHEE	Q90339	1272,52263	1,74E+06	1,61E+06	1,43E+06	High	636,76526	26,7069	2,84
LDHALNDMT	P13104	1029,46697	9,01E+05	1,38E+06	1,44E+06	High	515,23798	37,4173	2,41
EDNSAPVGLK	A0JME2	1029,52111	1,34E+06	1,86E+06	1,54E+06	High	515,26501	29,4364	1,92
EKSYELPDGQVIT	P83750	1478,73732	3,63E+06	2,71E+05	1,56E+06	High	739,87384	64,3460	2,71
HSQNTSLI	Q90339	899,45812	3,38E+05	1,32E+06	1,59E+06	High	450,23413	26,3565	2,20

LDKENAL	P13104	802,43051	6,92E+05	1,97E+06	1,61E+06	High	401,72006	21,9220	2,01
MAAQETQATQQSSVSNGEVSSNGAAASGQVAQTG	Q6DEL2	3121,42644	2,40E+06	7,64E+05	1,67E+06	High	1041,14368	57,2798	2,45
AAQETQATQQSSVSNGEVSSNGAAASGQVAQTG	Q6DEL2	3121,42644	2,40E+06	7,64E+05	1,67E+06	High	1041,14368	57,2798	2,45
KVIPELNGK	Q5MJ86	997,60406	3,57E+06	2,67E+06	1,69E+06	High	499,30673	26,4668	2,70
SKIEDEQSLGAQLQ	Q90339	1545,77549	8,46E+05	7,98E+05	1,73E+06	High	773,39233	49,5643	3,17
ATTYPSATSTFQTQVATSFPTSVASNIYSSPV	P26632	3311,59541	4,38E+06	5,11E+05	1,74E+06	High	828,65771	38,7606	3,17
EYDESGPSIVHR	P83750	1388,64408	1,85E+06	1,86E+06	1,77E+06	High	694,82684	33,0217	2,78
AHQQTLDDL	Q90339	1040,50071	7,19E+05	1,35E+06	1,79E+06	High	520,75519	36,3303	2,97
KLHVDPDNF	Q90485	1084,54218	3,76E+06	2,36E+06	1,79E+06	High	542,77588	38,3209	2,62
KLDKENALDRA	P13104	1272,69064	2,87E+06	1,88E+06	1,81E+06	High	424,90274	18,4335	2,90
FPSIVGR	P83750	775,44610	2,62E+06	2,66E+06	1,86E+06	High	388,22760	49,1833	2,04
ESAGIHETAY	P53479	1077,48473	2,38E+06	1,83E+06	1,88E+06	High	539,24744	29,0116	1,90
ELPDGQVITIGNE	P83750	1384,69545	1,62E+06	1,31E+06	1,94E+06	High	692,85229	80,4142	2,50
EDELDKY	P13104	911,39927	1,66E+06	2,45E+06	1,94E+06	High	456,20432	32,7649	1,99
KSYELPDGQVI	P83750	1248,64704	1,85E+06	1,76E+06	1,95E+06	High	624,82782	69,9984	2,10
KEITAL	P53479	674,40832	5,00E+05	3,34E+06	1,96E+06	High	337,70844	27,0541	2,27
KIEDEQSLGAQL	Q90339	1330,68489	1,60E+06	1,46E+06	2,02E+06	High	665,84717	53,3097	2,99
QRLQGEVEDL	Q90339	1186,60624	3,54E+06	2,28E+05	2,06E+06	High	593,80890	46,1534	2,89
EDQLSEIK	Q90339	961,48367	1,53E+06	2,46E+06	2,07E+06	High	481,24677	31,2134	2,32
NVLSGGTTMYPGIADR	P53479	1651,81083	4,64E+06	1,61E+06	2,08E+06	High	826,41138	69,0109	2,52
EYDEAGPSIVHRK	P53479	1500,74413	5,11E+06	3,31E+05	2,10E+06	High	500,92099	25,6152	3,77
YETDAIQR	Q90339	995,47925	1,79E+06	3,07E+06	2,19E+06	High	498,24442	25,1582	2,21
SYVGDEAQSK	P83750	1083,49529	1,30E+06	4,18E+06	2,20E+06	High	542,25238	17,7437	2,07
YDEAGPSIVH	P53479	1087,50546	1,84E+06	2,87E+06	2,23E+06	High	544,25775	38,2294	3,26
FPPDVAGNVDYK	Q66I73	1321,64229	3,62E+06	2,02E+06	2,25E+06	High	661,32599	59,8962	3,12
AFTIIDQNR	Q66I73	1077,56873	3,03E+06	1,31E+06	2,28E+06	High	539,28894	49,7041	2,02
LTEAPLNPK	P83750	982,55677	3,91E+06	1,77E+06	2,33E+06	High	491,78348	30,1791	2,68
RVFDKEGNGTVM	Q6P0G6	1352,66271	4,31E+06	2,72E+06	2,42E+06	High	451,56049	32,8608	3,59
DLENEKQQSDE	Q90339	1334,57064	3,29E+06	4,71E+06	2,51E+06	High	667,78931	18,8102	2,00
QAEEDKVNTL	Q90339	1146,56371	2,00E+06	2,63E+06	2,51E+06	High	573,78748	28,5912	2,37
HNTLATTNSSLSSSPPQTPLMNGT	A0JME2	2472,16711	4,28E+06	8,44E+05	2,62E+06	High	824,72144	56,3011	2,36
LKEADIT	P05939	789,43526	2,64E+06	3,75E+06	2,83E+06	High	395,22232	23,3792	2,19
KLEGDL	Q90339	674,37193	9,16E+05	3,33E+06	2,85E+06	High	337,69022	24,3393	2,03

EEAEGTLEHEESK	Q90339	1487,64962	3,98E+06	2,21E+06	2,88E+06	High	496,55594	20,4185	3,63
EEISERL	Q90339	875,44689	3,30E+06	3,04E+06	2,90E+06	High	438,22800	35,6574	2,02
APEEHPTLL	P53479	1006,52039	2,36E+06	4,20E+06	3,05E+06	High	503,76450	43,1647	2,89
KLEGDLKL	Q90339	915,55096	7,88E+06	3,91E+06	3,14E+06	High	458,28033	38,9409	3,44
PSIVGRPR	P83750	881,53156	2,54E+06	2,35E+06	3,23E+06	High	441,27039	16,2027	2,92
KEFLEEL	Q66I73	907,47713	3,24E+06	3,19E+06	3,52E+06	High	454,24310	61,8318	2,19
LVVYPQTK	P82315	947,55604	4,43E+06	2,40E+06	3,55E+06	High	474,28290	33,8711	1,97
KIEDEQSLGA	Q90339	1089,54224	3,84E+06	5,88E+06	3,92E+06	High	545,27612	29,2896	3,15
SKIEDEQSLGAQL	Q90339	1417,71691	2,06E+06	2,19E+06	4,17E+06	High	709,36304	54,8756	2,45
EKTIDDLE	P13104	962,46768	4,24E+06	3,63E+06	4,22E+06	High	481,73865	33,9511	2,38
SGFIEEEELK	P05939	1180,57321	6,42E+06	2,06E+06	4,31E+06	High	590,79126	53,9911	1,98
TKLEQQVDDLE	Q90339	1317,65325	4,85E+06	2,71E+06	4,31E+06	High	659,33105	48,7272	2,60
SDHHVYLE	P53448	999,45304	6,33E+06	7,56E+06	4,40E+06	High	500,23132	21,7771	2,22
VFDKEGNGTVM	Q6P0G6	1196,56160	4,87E+06	4,62E+06	4,49E+06	High	598,78546	39,8696	3,43
KDIDDLE	Q90339	847,40435	5,03E+06	8,14E+06	4,90E+06	High	424,20703	31,0015	2,21
PEILPDGDHDLK	P53448	1348,67432	8,78E+06	2,84E+06	4,94E+06	High	450,23056	43,4915	3,23
LEQQVDDLE	Q90339	1088,51061	4,10E+06	5,43E+06	5,21E+06	High	544,76025	48,1809	1,96
NWDDMEK	P83750	937,37201	6,06E+06	4,95E+06	5,29E+06	High	469,19086	35,9681	2,11
SGGTTMYPGIADRM	P83750	1456,65591	7,04E+06	2,59E+06	5,85E+06	High	728,83289	61,6956	2,68
RVPTPNVSV	Q5XJ10	968,55236	5,64E+06	4,54E+06	5,96E+06	High	484,78088	42,1787	2,38
YPGIADR	P83750	791,40463	4,96E+06	7,77E+06	6,29E+06	High	396,20728	28,4543	2,02
KKDIDDLE	Q90339	975,49932	2,27E+06	5,96E+06	6,99E+06	High	488,25430	19,8214	2,25
LHVDPDNF	Q90485	956,44722	8,07E+06	5,47E+06	7,04E+06	High	478,72867	51,9674	2,14
NFDKVLAE	Q90339	935,48327	8,74E+06	1,76E+06	7,05E+06	High	468,24628	45,0160	1,98
AGFAGDDAPR	P83750	976,44828	1,55E+07	6,88E+06	7,18E+06	High	488,72873	24,9707	2,17
TVDGPSGKL	Q5XJ10	873,46762	1,17E+07	8,17E+06	7,31E+06	High	437,23816	24,7861	2,35
SGGTTMYPGIADR	P83750	1325,61543	7,66E+06	6,51E+06	7,37E+06	High	663,31268	44,6828	2,78
DNGSGLVKA	P53479	860,44722	9,45E+06	1,02E+07	7,61E+06	High	430,72812	24,4373	2,58
KSYELPDGQ	P83750	1036,49457	8,37E+06	7,69E+06	7,95E+06	High	518,75201	33,9752	2,89
IEDEQSLGAQ	Q90339	1089,50586	7,94E+06	1,19E+07	8,04E+06	High	545,25775	34,9011	3,47
KQEYDEAGPSIVHR	P53479	1628,80271	1,54E+07	1,12E+07	9,25E+06	High	543,60693	28,7563	5,55
MDAIKK	P13104	747,40694	1,19E+07	1,39E+07	9,35E+06	High	374,20819	27,9986	1,95
EYDEAGPSIVH	P53479	1216,54806	7,06E+06	1,06E+07	9,63E+06	High	608,77844	42,0206	2,77

YPGIADRM	P83750	922,44511	6,65E+06	5,56E+06	9,63E+06	High	461,72751	47,1153	2,20
KSYELPDGQV	P83750	1135,56298	1,04E+07	6,08E+06	9,64E+06	High	568,28687	46,9146	3,33
SKIEDEQSLGA	Q90339	1176,57427	8,00E+06	1,11E+07	9,90E+06	High	588,79199	31,0267	3,02
KVLDPEAT	Q66I73	872,47237	8,57E+06	1,73E+07	1,01E+07	High	436,74020	23,1740	2,34
DKENALDRAEQA	P13104	1359,64990	1,49E+07	1,05E+07	1,12E+07	High	680,32935	27,4534	2,34
RVIDSM	Q90339	720,37089	6,45E+06	1,73E+07	1,13E+07	High	360,69034	24,4207	2,07
YDEAGPSIVHR	P53479	1243,60658	1,16E+07	1,43E+07	1,26E+07	High	415,20801	30,8969	3,59
RVIISAPSA	Q5XJ10	913,54654	7,46E+05	7,13E+05	1,26E+07	High	457,27795	38,3893	2,60
IIAPPER	P83750	795,47231	1,73E+07	1,49E+07	1,28E+07	High	398,24078	27,0746	1,99
EKTIDDLEDE	P13104	1206,53722	1,46E+07	1,00E+07	1,33E+07	High	603,77399	41,3949	2,02
KTIDDLEDE	P13104	1077,49463	1,42E+07	1,47E+07	1,34E+07	High	539,25189	36,0021	2,67
TKLEQQVDDL	Q90339	1188,61066	1,48E+07	7,97E+06	1,35E+07	High	594,81079	47,4857	3,27
SYELPDGQVIT	P83750	1221,59976	1,89E+07	1,10E+07	1,52E+07	High	611,30511	82,2403	2,47
KIEDEQSLGAQ	Q90339	1217,60082	1,63E+07	2,12E+07	1,55E+07	High	609,30493	27,5526	3,69
NLEVTVK	Q90339	802,46689	1,35E+07	1,32E+07	1,57E+07	High	401,73831	29,1183	2,06
LDHALNDM	P13104	928,41929	9,09E+06	1,57E+07	1,58E+07	High	464,71469	35,4201	2,21
IIDQDKSGF	P02618	1022,51530	3,15E+07	4,37E+06	1,69E+07	High	511,76260	35,4479	1,94
QDLVDKL	Q90339	830,46181	1,59E+07	2,01E+07	2,20E+07	High	415,73572	48,5782	2,55
FAGDDAPR	P83750	848,38971	4,03E+07	5,78E+07	3,80E+07	High	424,69943	19,0487	2,08
TEAPLNPK	P83750	869,47271	1,38E+07	8,12E+07	4,20E+07	High	435,24106	19,5408	2,06
SKIEDEQSLGAQ	Q90339	1304,63285	3,33E+07	4,53E+07	4,45E+07	High	652,82135	30,2191	2,36
KSYELPDGQVIT	P83750	1349,69472	6,76E+07	3,45E+07	5,49E+07	High	675,35260	62,6497	3,16
DSYVGDEAQSKR	P83750	1354,62335	1,06E+08	4,02E+07	6,67E+07	High	452,21390	18,8076	3,19
DSYVGDEAQSK	P83750	1198,52224	9,18E+07	1,26E+08	7,98E+07	High	599,76617	22,7211	2,71
EYDEAGPSIVHR	P53479	1372,64917	7,45E+07	8,36E+07	7,99E+07	High	458,22208	34,4207	3,78
RVAPEEHPTL	P53479	1148,60585	5,40E+07	7,85E+07	8,07E+07	High	574,80768	27,1336	1,95
RVAPEEHPTLL	P53479	1261,68991	5,04E+07	5,91E+07	8,46E+07	High	421,23615	38,9356	2,52
VAPEEHPTL	P53479	992,50474	7,76E+07	1,14E+08	9,61E+07	High	496,75656	33,4382	2,09
VAPEEHPTLL	P53479	1105,58880	2,94E+07	3,31E+07	1,02E+08	High	553,29938	49,9830	2,14
GFAGDDAPR	P83750	905,41117	2,82E+08	3,34E+08	2,71E+08	High	453,21024	22,6187	2,24
RVFDKDGNGYISA	Q6PI52	1441,70702	4,05E+05	#DIV/0!	#DIV/0!	High	481,24185	41,3208	3,54

Table S6. Peptides Sequences identified in AP and BL HH ctrl, AP and BL HH40 and AP and BL HH80.

Sequence	Master Protein Accessions	Theo. MH+ [Da]	AP HH ctrl	BL HH ctrl	AP HH40	BL HH40	AP HH80	BL HH80	m/z [Da] (by Search Engine): Sequest HT
LDIAGR	P83750	644,37260	4,93E+05	5,31E+06	1,07E+06	7,79E+06	4,86E+05	4,82E+06	322,6907
RGILTL	P83750	672,44029	1,56E+07	0,00E+00	1,72E+07	0,00E+00	2,81E+07	0,00E+00	336,7245
KLEGDL	Q90339	674,37193	0,00E+00	1,35E+07	0,00E+00	2,13E+07	0,00E+00	1,77E+07	337,6906
KEITAL	P53479	674,40832	0,00E+00	3,08E+06	3,35E+05	7,43E+06	0,00E+00	5,32E+06	337,7085
PDLSLH	Q7SXS7	681,35662	9,12E+06	7,72E+06	1,20E+07	1,35E+07	1,18E+07	8,23E+06	341,1828
KLEADL	Q6NWF6	688,38758	3,62E+05	4,56E+05	2,74E+05	3,46E+05	3,07E+05	3,62E+05	344,6985
KEAFSL	Q6PI52	694,37702	8,71E+05	3,76E+05	0,00E+00	1,53E+05	3,63E+05	2,10E+05	347,6934
KLVIVE	P13104	700,46035	2,48E+06	0,00E+00	2,19E+06	0,00E+00	2,90E+06	0,00E+00	350,7347
KDIDDL	Q90339	718,36176	5,94E+05	9,78E+06	1,27E+06	1,02E+07	5,78E+05	8,02E+06	359,6855
RVIDSM	Q90339	720,37089	1,70E+06	1,51E+07	7,32E+06	1,48E+07	1,75E+06	2,03E+07	360,6901
PSIVGRP	P83750	725,43045	4,31E+06	1,29E+07	3,24E+06	8,32E+06	2,19E+06	5,49E+06	363,2196
EHAVTAL	Q5PYH5	740,39373	8,71E+05	2,12E+06	1,66E+06	3,21E+06	1,92E+06	2,91E+06	370,7019
KLLGSID	Q90339	745,44543	6,16E+06	2,11E+06	8,70E+06	3,31E+06	6,76E+06	3,06E+06	373,2275
MDAIKK	P13104	747,40694	3,64E+06	5,55E+07	4,20E+06	4,81E+07	4,27E+06	4,26E+07	374,2082
KGELVPL	P12115	755,46617	1,81E+06	0,00E+00	0,00E+00	1,02E+05	4,52E+06	0,00E+00	378,2379
RVIISAP	Q5XJ10	755,47740	5,46E+05	0,00E+00	8,61E+05	0,00E+00	4,26E+05	0,00E+00	378,2433
ADLGNIR	Q9W7R3	758,41553	2,61E+05	8,43E+05	6,48E+05	1,33E+06	4,84E+05	1,34E+06	379,7125
KFFVGGN	Q1MTI4	768,40390	1,93E+06	1,02E+06	1,59E+06	1,37E+06	2,96E+06	1,31E+06	384,7073
KVEIVAI	Q5XJ10	771,49747	4,97E+05	0,00E+00	9,49E+05	8,89E+04	1,16E+06	1,18E+05	386,2536
MDAKLR	F1QCY8	775,41309	1,16E+06	5,19E+06	1,17E+06	3,27E+06	1,36E+06	4,63E+06	388,2108
FPSIVGR	P83750	775,44610	2,21E+06	8,55E+04	2,20E+06	5,04E+05	1,63E+06	2,22E+05	388,2281
IDNIFR	Q9PTY0	777,42536	3,17E+05	4,87E+04	4,04E+05	1,61E+05	1,28E+05	0,00E+00	389,2170
LEGDLKL	Q90339	787,45600	8,39E+06	3,90E+06	7,61E+06	5,52E+06	8,34E+06	5,82E+06	394,2328
KLPFQR	Q6PI20	788,47773	6,03E+05	6,59E+05	8,31E+05	6,27E+05	7,99E+05	6,61E+05	394,7441
KDGLEVM	Q1LUA6	791,39677	5,23E+05	2,92E+05	5,43E+05	1,72E+05	3,29E+05	2,12E+05	396,2032
KEFLEE	Q66I73	794,39306	1,34E+07	7,02E+07	2,38E+07	9,80E+07	2,14E+07	8,81E+07	397,7013
IIAPPER	P83750	795,47231	5,39E+06	4,64E+07	6,26E+06	3,22E+07	3,82E+06	3,34E+07	398,2409
YTVFDR	Q9DEX3	800,39373	1,09E+07	1,47E+07	2,28E+07	1,53E+07	1,97E+07	1,74E+07	400,7022
RLDIAGR	P83750	800,47371	0,00E+00	1,11E+06	2,94E+04	3,42E+06	0,00E+00	1,73E+06	400,7420

NLEVTVK	Q90339	802,46689	8,21E+07	2,59E+07	8,48E+07	3,32E+07	1,33E+08	3,88E+07	401,7384
KNLEVTV	Q90339	802,46689	1,02E+07	1,59E+06	1,13E+07	3,07E+06	5,94E+06	1,35E+06	401,7384
KVIPELN	Q5MJ86	812,48763	1,53E+06	6,97E+05	2,45E+06	1,07E+06	1,08E+06	6,84E+05	406,7489
DHALNDM	P13104	815,33523	2,58E+05	5,84E+06	1,00E+06	5,47E+06	3,01E+05	5,49E+06	408,1724
IKESDII	F1QBY1	817,46656	9,26E+07	2,02E+07	9,52E+07	3,81E+07	9,21E+07	3,14E+07	409,2384
FDKVLAE	Q90339	821,44035	5,25E+05	2,82E+06	4,52E+05	8,08E+05	4,17E+05	2,12E+06	411,2253
KEAFTII	Q66I73	821,47673	3,89E+05	0,00E+00	3,35E+05	0,00E+00	5,50E+05	0,00E+00	411,2434
RVIQYF	Q90339	825,46175	7,37E+06	0,00E+00	7,64E+06	0,00E+00	1,95E+07	0,00E+00	413,2361
RDILEAL	Q1LUA6	829,47779	5,89E+05	3,02E+04	1,15E+06	1,62E+05	4,67E+05	3,89E+04	415,2437
QDLVDKL	Q90339	830,46181	8,81E+07	3,00E+07	1,12E+08	6,97E+07	1,16E+08	5,18E+07	415,7359
VWAIGTGK	Q1MTI4	831,47231	1,57E+06	1,47E+06	1,36E+06	1,50E+06	1,51E+06	1,39E+06	416,2409
GLAGPLHGL	Q7ZVY5	834,48321	7,79E+05	0,00E+00	1,35E+06	7,06E+04	9,45E+05	6,70E+04	417,7466
GPPGLIGPK	A0MSJ1	835,50361	1,43E+06	1,05E+06	1,41E+06	1,34E+06	1,59E+06	1,23E+06	418,2568
RDTFGEL	Q7SX99	837,41011	1,74E+06	7,50E+05	3,19E+06	1,53E+06	1,66E+06	8,84E+05	419,2100
FKEAFSL	Q6PI52	841,44543	4,77E+06	0,00E+00	7,90E+04	0,00E+00	2,04E+06	0,00E+00	421,2279
RVIISAPS	Q5XJ10	842,50943	7,41E+05	7,94E+04	7,18E+05	2,25E+05	5,42E+05	8,95E+04	421,7599
HVDPDNF	Q90485	843,36316	6,89E+06	1,97E+07	1,09E+07	1,90E+07	7,99E+06	1,68E+07	422,1870
VNGIDLRG	Q5PYH5	843,46829	1,10E+08	1,59E+08	2,11E+08	2,89E+08	1,79E+08	2,17E+08	422,2392
DDLRDVL	Q66I73	845,43632	1,21E+07	2,11E+06	6,29E+06	2,47E+06	9,37E+06	2,29E+06	423,2227
DGTEGLVR	Q9PTY0	846,43157	1,30E+05	1,92E+06	2,47E+05	2,15E+06	1,74E+05	1,34E+06	423,7208
KDIDDLE	Q90339	847,40435	1,64E+06	3,21E+07	2,32E+06	3,04E+07	2,07E+06	2,87E+07	424,2074
FAGDDAPR	P83750	848,38971	2,41E+06	1,83E+08	6,22E+06	1,74E+08	1,72E+06	1,55E+08	424,6998
KSYELPD	P83750	851,41452	5,34E+05	0,00E+00	4,79E+05	1,10E+06	6,52E+05	1,07E+06	426,2125
GVVGLPGPR	C7DZK3	851,50976	8,83E+05	7,47E+05	1,97E+05	2,73E+05	5,40E+05	3,84E+05	426,2599
GIGTVPVGR	Q92005	855,50468	9,94E+05	5,94E+06	1,69E+06	5,37E+06	1,11E+06	4,83E+06	428,2575
HQGVMVGM	P83750	858,39605	1,73E+06	3,02E+05	2,10E+06	0,00E+00	1,96E+06	3,45E+05	429,7028
DNGSGLVKA	P53479	860,44722	1,62E+06	7,46E+07	2,23E+06	5,36E+07	1,39E+06	6,53E+07	430,7286
TEAPLNPK	P83750	869,47271	1,45E+05	1,56E+08	1,04E+06	4,20E+08	1,11E+05	2,87E+08	435,2410
KVIPELNG	Q5MJ86	869,50909	8,00E+05	3,56E+05	9,82E+05	4,55E+05	4,07E+05	4,20E+04	435,2596
RVQLELN	Q90339	871,49959	1,49E+07	0,00E+00	2,67E+07	0,00E+00	2,69E+07	0,00E+00	436,2550
KVLDPEAT	Q66I73	872,47237	1,00E+06	2,61E+07	2,79E+06	2,98E+07	1,21E+06	3,46E+07	436,7414
TVDGPSGKL	Q5XJ10	873,46762	3,96E+06	5,84E+07	3,27E+06	2,95E+07	2,59E+06	3,66E+07	437,2389
GLQFPVGR	Q71PD7	873,49411	4,38E+06	9,00E+05	4,88E+06	1,24E+06	1,81E+06	6,16E+05	437,2519

VFPSIVGR	P83750	874,51451	4,10E+06	2,29E+05	4,40E+06	7,34E+05	2,55E+06	3,16E+05	437,7621
SEVAQWR	Q90339	875,43699	5,15E+05	4,05E+05	8,03E+05	6,84E+05	7,29E+05	6,41E+05	438,2237
EEISERL	Q90339	875,44689	2,12E+07	1,13E+07	2,39E+07	1,43E+07	1,78E+07	1,14E+07	438,2281
ISERLEE	Q90339	875,44689	5,06E+06	7,32E+06	4,02E+06	6,73E+06	5,76E+06	1,06E+07	438,2288
LESRLEE	A0A2R8QCI3	875,44689	5,06E+06	7,32E+06	4,02E+06	6,73E+06	5,76E+06	1,06E+07	438,2288
EISERLE	Q90339	875,44689	5,06E+06	7,32E+06	4,02E+06	6,73E+06	5,76E+06	1,06E+07	438,2287
YEDIANR	Q6NWF6	880,41592	1,21E+05	6,84E+06	4,50E+05	1,05E+07	2,11E+05	7,88E+06	440,7129
PSIVGRPR	P83750	881,53156	6,15E+05	2,21E+07	3,67E+05	1,51E+07	2,86E+05	1,42E+07	441,2709
LTDYLMK	P83750	883,45937	2,49E+05	0,00E+00	1,69E+05	0,00E+00	1,73E+05	0,00E+00	442,2351
GIALNDHF	Q5XJ10	886,44174	6,01E+06	0,00E+00	6,29E+06	0,00E+00	4,80E+06	0,00E+00	443,7263
ELEEELK	P13104	889,45130	1,34E+06	9,16E+06	2,11E+06	1,31E+07	1,27E+06	6,68E+06	445,2307
RDLTDYL	P83750	895,45197	2,78E+06	7,85E+04	3,81E+06	3,26E+05	1,29E+06	9,64E+04	448,2309
PDGDHDLK	P53448	896,41084	5,02E+05	2,48E+06	5,71E+05	2,42E+06	5,14E+05	2,42E+06	448,7104
HSQNTSLI	Q90339	899,45812	4,00E+05	2,17E+05	1,59E+06	1,23E+06	7,09E+05	9,78E+05	450,2343
ELINNVAK	Q9PTY0	900,51491	5,02E+05	1,34E+06	8,47E+05	1,76E+06	5,43E+05	1,34E+06	450,7623
LGEQIDNL	Q90339	901,46254	1,57E+07	5,35E+06	2,37E+07	1,06E+07	2,04E+07	8,12E+06	451,2364
VITIGNER	P83750	901,51016	5,85E+06	7,71E+06	1,41E+06	3,28E+06	3,28E+06	4,05E+06	451,2601
GFAGDDAPR	P83750	905,41117	5,95E+07	2,04E+09	9,75E+07	1,78E+09	7,81E+07	1,72E+09	453,2106
KEFLEEL	Q66I73	907,47713	4,38E+07	1,21E+06	4,51E+07	2,91E+06	6,14E+07	2,70E+06	454,2439
LFLQNFK	P05939	909,51926	1,86E+07	0,00E+00	1,27E+06	0,00E+00	7,23E+06	0,00E+00	455,2650
EDELDKY	P13104	911,39927	5,52E+05	8,55E+06	9,56E+05	1,29E+07	9,06E+05	1,10E+07	456,2049
GGDRIPADL	P25489	913,47377	3,27E+05	2,68E+05	4,16E+05	3,40E+05	2,87E+05	2,50E+05	457,2421
RVIISAPSA	Q5XJ10	913,54654	4,99E+06	0,00E+00	5,69E+06	0,00E+00	3,83E+06	0,00E+00	457,2782
LPESDLGAL	Q9W7R4	914,48294	5,57E+06	8,88E+05	7,43E+06	1,73E+06	8,02E+06	1,62E+06	457,7471
RLDIAGRD	P83750	915,50065	8,78E+05	9,76E+06	1,78E+06	6,36E+06	2,71E+05	8,17E+06	458,2550
KLEGDLKL	Q90339	915,55096	5,53E+07	4,55E+06	2,73E+07	4,12E+06	5,58E+07	8,15E+06	458,2807
DLQHGSLF	Q9W7K5	916,45231	5,16E+06	4,12E+05	8,65E+06	1,14E+06	4,75E+06	6,31E+05	458,7312
GIHETTFN	P83750	918,43157	4,47E+05	4,46E+05	9,65E+05	1,30E+06	7,30E+05	7,39E+05	459,7206
GAHQNIIPA	Q5MJ86	920,49484	5,31E+05	1,00E+06	7,56E+05	1,01E+06	7,59E+05	1,08E+06	460,7525
MYPGIADR	P83750	922,44511	2,43E+07	1,79E+07	3,73E+07	1,72E+07	4,01E+07	2,56E+07	461,7279
YPGIADRM	P83750	922,44511	2,82E+06	2,73E+05	2,51E+06	5,85E+05	3,63E+06	6,07E+05	461,7275
HTFYNEL	P83750	923,42576	9,10E+05	0,00E+00	1,34E+06	0,00E+00	1,18E+06	0,00E+00	462,2177
LDHALNDM	P13104	928,41929	1,21E+07	9,33E+06	2,44E+07	9,40E+06	2,41E+07	1,71E+07	464,7146

KIDDLVGGL	Q90487	929,53022	1,80E+06	0,00E+00	1,66E+06	1,21E+05	1,32E+06	5,73E+04	465,2702	
GQVITIGNE	P83750	930,48909	1,32E+06	6,58E+05	2,01E+06	1,42E+06	1,67E+06	7,82E+05	465,7494	
LDESKLIL	A0A0R4IBK5	930,55062	8,94E+05	0,00E+00	8,14E+05	0,00E+00	1,12E+06	0,00E+00	465,7809	
RDNLGEDI	Q92155	931,44795	2,10E+06	9,33E+05	2,94E+06	1,74E+06	1,96E+06	1,09E+06	466,2288	
IEKPMGIF	Q90339	934,50665	1,02E+06	0,00E+00	7,42E+05	0,00E+00	1,03E+06	0,00E+00	467,7588	
NFDKVLAE	Q90339	935,48327	4,22E+07	2,05E+07	5,99E+06	9,02E+06	2,99E+07	1,70E+07	468,2466	
PEEHPTLL	P53479	935,48327	6,51E+05	1,58E+05	1,24E+06	4,83E+05	1,92E+06	5,23E+05	468,2466	
REYQDLL	Q92155	936,47852	2,79E+06	1,72E+05	3,19E+06	5,41E+05	2,66E+06	3,15E+05	468,7444	
NWDDMEK	P83750	937,37201	5,56E+06	2,10E+07	5,09E+06	9,37E+06	5,78E+06	1,58E+07	469,1914	
MYPGIADR	P83750	938,44003	5,03E+05	4,58E+06	1,52E+06	9,58E+06	1,30E+06	7,77E+06	469,7255	
TFYNELR	P83750	942,46796	4,44E+06	0,00E+00	4,42E+06	0,00E+00	3,68E+06	0,00E+00	471,7392	
LDHALNDM	P13104	944,41421	2,96E+05	4,08E+06	1,44E+06	9,61E+06	9,06E+05	1,03E+07	472,7124	
AGLQFPVGR	Q71PD7	944,53123	1,18E+07	2,06E+06	5,85E+06	6,49E+06	4,55E+06	1,91E+06	472,7705	
AVFPSIVGR	P83750	945,55163	1,60E+06	1,61E+05	9,14E+05	2,39E+05	1,59E+06	4,09E+05	473,2806	
KSKELDII	Q7ZUV7	945,56152	3,99E+07	3,54E+06	2,79E+07	7,90E+06	4,63E+07	6,84E+06	473,2856	
SAGIHETAY	P53479	948,44214	6,61E+05	1,61E+06	5,62E+05	1,60E+06	4,68E+05	1,33E+06	474,7259	
GLNSADMLK	Q90339	948,48189	1,31E+06	1,70E+06	2,00E+06	1,75E+06	1,83E+06	2,44E+06	474,7462	
NGLAGPLHGL	Q7ZVY5	948,52614	4,69E+05	0,00E+00	5,49E+05	0,00E+00	5,64E+05	0,00E+00	474,7677	
LTPTTPGGLP	Q6IQE0	953,53022	1,19E+06	1,45E+04	1,71E+06	4,39E+04	1,37E+05	1,12E+04	477,2701	
LHVDPDNF	Q90485	956,44722	4,29E+07	1,43E+06	3,63E+07	4,79E+06	3,85E+07	2,46E+06	478,7285	
LEQQVDDL	Q90339	959,46802	1,05E+07	1,33E+07	1,42E+07	1,38E+07	7,21E+06	1,17E+07	480,2392	
DLEDALQR	Q6NWF6	959,47925	3,78E+06	0,00E+00	5,04E+06	0,00E+00	2,10E+06	1,84E+06	480,2447	
KIEDEQSL	Q90339	961,48367	3,25E+06	3,96E+07	5,44E+06	5,31E+07	3,36E+06	4,19E+07	481,2473	
EDQLSEIK	Q90339	961,48367	6,13E+05	8,38E+06	1,21E+06	1,20E+07	1,21E+06	9,40E+06	481,2470	
YYTVFDR	Q9DEX3	963,45706	2,70E+07	0,00E+00	5,03E+07	0,00E+00	2,93E+07	0,00E+00	482,2339	
EAGPSIVHR	P53479	965,51630	3,08E+04	1,38E+06	1,02E+05	9,96E+05	2,33E+04	1,16E+06	483,2634	
YEDIANRS	Q6NWF6	967,44795	7,29E+04	2,19E+06	8,55E+04	1,41E+06	7,81E+04	2,14E+06	484,2295	
DIVWPEPL	F1QCV2	968,50876	3,92E+06	4,85E+05	6,03E+06	9,59E+05	4,21E+06	7,42E+05	484,7578	
RVPTPNVSV	Q5XJ10	968,55236	4,53E+07	9,39E+06	4,35E+07	1,39E+07	3,30E+07	1,11E+07	484,7810	
KETDSKVY	Q5U3G1	969,48875	9,68E+05	1,28E+05	1,78E+06	5,64E+05	2,09E+06	2,86E+05	485,2502	
DRVINQIL	Q7ZU99	970,56801	5,47E+05	0,00E+00	3,77E+05	0,00E+00	5,53E+05	0,00E+00	485,7890	
HVDPDNFK	Q90485	971,45812	5,39E+04	2,96E+06	1,20E+05	2,11E+06	2,52E+04	2,78E+06	486,2335	
ELPDGQVIT	P83750	971,50440	7,52E+06	1,40E+07	1,44E+07	2,05E+07	7,95E+06	1,02E+07	486,2570	

GYALPHAIM	P53479	972,49715	3,58E+06	0,00E+00	3,19E+06	0,00E+00	3,36E+06	0,00E+00	486,7537
KDDLRDVL	Q66I73	973,53129	3,06E+06	0,00E+00	9,97E+05	0,00E+00	2,11E+06	0,00E+00	487,2705
KKDIDDLE	Q90339	975,49932	2,35E+04	1,24E+07	5,66E+04	2,12E+07	2,04E+04	2,17E+07	488,2547
DGIISKDDL	Q66I73	975,49932	2,94E+06	1,68E+06	8,47E+05	1,12E+06	2,29E+06	1,51E+06	488,2549
AGFAGDDAPR	P83750	976,44828	2,65E+06	2,64E+07	1,56E+06	1,91E+07	1,63E+06	1,42E+07	488,7296
GFAGDDAPRA	P83750	976,44828	3,13E+05	4,44E+06	1,26E+05	1,57E+06	3,15E+05	2,41E+06	488,7297
FRENLGKL	Q90339	976,55744	9,15E+06	0,00E+00	6,60E+06	0,00E+00	6,22E+06	0,00E+00	488,7841
DVAGNVDYK	Q66I73	980,46835	6,47E+05	5,96E+06	7,86E+05	5,23E+06	2,44E+05	3,16E+06	490,7395
LTEAPLNPK	P83750	982,55677	8,14E+05	1,01E+07	5,84E+05	4,74E+06	6,86E+05	6,14E+06	491,7835
NVIGEPIDE	Q9PTY0	985,48367	1,02E+06	5,24E+05	1,97E+06	9,22E+05	9,74E+05	5,33E+05	493,2477
SILNNGHSF	Q92051	988,48467	2,50E+06	0,00E+00	2,66E+06	0,00E+00	2,73E+06	0,00E+00	494,7476
LEEISERL	Q90339	988,53095	6,93E+05	0,00E+00	4,26E+05	0,00E+00	6,98E+05	0,00E+00	494,7705
NSEVAQWR	Q90339	989,47992	1,76E+06	0,00E+00	4,06E+06	0,00E+00	2,53E+06	0,00E+00	495,2460
SQDESIAKL	Q90339	990,51022	3,71E+06	9,61E+06	4,76E+06	9,96E+06	9,79E+06	1,81E+07	495,7599
VAPEEHPVL	P83750	990,52547	3,71E+06	9,61E+06	4,76E+06	9,96E+06	9,79E+06	1,81E+07	495,7677
VAPEEHPTL	P53479	992,50474	3,67E+07	2,81E+08	6,29E+07	3,04E+08	4,72E+07	3,56E+08	496,7577
YVVNETIGV	Q6JAN0	993,52514	6,13E+05	4,46E+04	4,69E+05	1,04E+05	9,42E+04	0,00E+00	497,2674
YETDAIQR	Q90339	995,47925	2,90E+05	5,96E+06	7,28E+05	7,67E+06	4,80E+05	8,47E+06	498,2447
KVIPELNGK	Q5MJ86	997,60406	4,05E+06	2,55E+06	3,90E+06	2,32E+06	3,02E+06	2,49E+06	499,3075
DLTDYLMK	P83750	998,48631	3,50E+06	4,72E+04	1,52E+06	9,07E+04	4,55E+06	1,17E+05	499,7479
SDHHVYLE	P53448	999,45304	4,48E+06	5,56E+06	1,14E+07	9,62E+06	3,45E+06	7,83E+06	500,2322
DYLVGDHAI	Q7SXS7	1002,48909	6,03E+06	1,20E+06	4,72E+06	1,80E+06	5,51E+06	1,46E+06	501,7497
IEEEELKL	P05939	1002,53537	1,49E+06	7,02E+05	0,00E+00	1,45E+05	1,85E+05	1,89E+05	501,7726
APEEHPTLL	P53479	1006,52039	2,60E+06	3,65E+06	4,31E+06	4,08E+06	5,23E+06	7,52E+06	503,7653
FTIIDQNR	Q66I73	1006,53162	7,06E+06	2,06E+06	3,88E+06	2,27E+06	5,30E+06	2,06E+06	503,7713
SYELPDGQV	P83750	1007,46802	3,96E+06	2,56E+06	4,40E+06	2,48E+06	2,56E+06	2,03E+06	504,2392
SGLDLVDKY	Q5U3Q6	1009,52005	8,42E+05	8,04E+06	1,01E+06	6,15E+06	7,66E+05	7,05E+06	505,2649
AINDPFIDL	Q5MJ86	1017,52514	3,35E+06	0,00E+00	5,32E+06	6,54E+04	3,42E+06	2,35E+04	509,2677
IIDQDKSGF	P02618	1022,51530	2,37E+07	8,74E+07	2,91E+06	2,24E+07	1,18E+07	4,13E+07	511,7629
IGGIGTVPVGR	Q92005	1025,61020	3,75E+05	9,21E+05	2,19E+05	3,11E+06	2,68E+05	8,31E+05	513,3104
KMTQIMFE	P83750	1027,49510	1,71E+06	0,00E+00	9,34E+05	8,06E+04	2,52E+06	3,37E+04	514,2520
NFMLIIDY	A0A0R4IES7	1028,51213	2,71E+06	1,44E+05	2,88E+06	1,66E+05	1,67E+06	2,22E+05	514,7629
RVIISAPSAD	Q5XJ10	1028,57348	2,73E+06	2,44E+05	2,46E+06	7,01E+05	2,01E+06	2,62E+05	514,7926

FPSIVGRPR	P83750	1028,59997	5,02E+06	3,24E+04	2,79E+06	7,69E+04	3,99E+06	3,81E+04	343,5392
LDHALNDMT	P13104	1029,46697	1,18E+06	7,57E+05	3,98E+06	1,05E+06	2,34E+06	1,21E+06	515,2385
LGEQIDNLQ	Q90339	1029,52111	1,33E+06	6,92E+05	2,42E+06	1,16E+06	1,64E+06	1,01E+06	515,2658
LDDISKIPE	Q7SYK7	1029,54627	3,22E+05	5,01E+05	5,42E+05	8,83E+05	2,58E+05	4,08E+05	515,2772
WAAFPPDVAG	Q66I73	1030,49926	5,71E+05	0,00E+00	2,43E+05	0,00E+00	3,72E+05	0,00E+00	515,7544
HVFGESDEL	Q1MTI4	1032,46327	1,57E+06	4,63E+05	5,78E+05	3,03E+05	1,06E+06	3,62E+05	516,7368
YRPGTVALR	Q6PI20	1032,59489	2,19E+05	1,20E+05	8,81E+04	4,40E+05	1,99E+05	1,07E+05	344,8707
EITALAPSTM	P53479	1033,52342	6,93E+05	3,70E+04	7,06E+05	8,91E+04	4,45E+05	4,26E+04	517,2671
VEVTVIMLN	Q6JAN0	1033,55981	2,16E+06	0,00E+00	4,08E+06	0,00E+00	5,43E+06	0,00E+00	517,2813
KSYELPDGQ	P83750	1036,49457	2,87E+06	1,66E+07	3,12E+06	1,28E+07	3,16E+06	1,70E+07	518,7521
AHQQTLDDL	Q90339	1040,50071	1,40E+06	0,00E+00	2,63E+06	0,00E+00	3,19E+06	0,00E+00	520,7551
NIIPASTGAAK	Q5MJ86	1042,58914	5,60E+05	1,26E+06	4,12E+05	8,19E+05	5,80E+05	1,26E+06	521,8002
IGMESAGIHE	P53479	1043,48262	2,77E+06	1,71E+06	4,69E+05	5,28E+05	1,19E+06	8,50E+05	522,2468
MEIGTTGAVGAAP	A3KPK0	1043,53676	2,65E+05	0,00E+00	6,75E+05	7,11E+04	3,72E+05	4,79E+04	522,2728
EIGTTGAVGAAP	A3KPK0	1043,53677	2,65E+05	0,00E+00	6,75E+05	7,11E+04	3,72E+05	4,79E+04	522,2728
KDDLRDVLA	Q66I73	1044,56840	8,50E+05	2,31E+05	1,83E+05	1,05E+05	7,10E+05	3,06E+05	522,7891
DGQVITIGNE	P83750	1045,51603	1,16E+06	1,35E+06	2,08E+06	1,37E+06	1,05E+06	1,03E+06	523,2632
LIEKPMGIF	Q90339	1047,59071	1,20E+06	0,00E+00	7,81E+05	0,00E+00	1,43E+06	0,00E+00	524,3007
SKIEDEQSL	Q90339	1048,51570	1,01E+07	4,42E+07	1,45E+07	4,57E+07	1,37E+07	5,44E+07	524,7631
SSLTGSQQLQ	Q6NSM8	1048,52693	1,01E+07	4,42E+07	1,45E+07	4,57E+07	1,37E+07	5,44E+07	524,7684
MYPGIADRM	P83750	1053,48560	9,60E+07	0,00E+00	7,52E+07	0,00E+00	1,16E+08	0,00E+00	527,2477
GYLIDGYPR	P12115	1053,53637	1,97E+06	0,00E+00	7,16E+05	6,78E+04	1,31E+06	4,38E+04	527,2731
SGGTTMYPGIA	P83750	1054,48737	6,06E+06	3,94E+06	1,08E+07	3,95E+06	6,12E+06	4,48E+06	527,7486
GPPGLPGPPGIP	C7DZK3	1055,58841	2,23E+05	0,00E+00	4,09E+05	0,00E+00	4,50E+05	3,15E+04	528,3003
ETAYNSIMK	P53479	1056,50302	1,33E+06	9,34E+05	1,27E+06	6,28E+05	1,26E+06	6,52E+05	528,7565
ELDHALNDM	P13104	1057,46189	3,15E+06	6,34E+05	5,44E+06	6,90E+05	3,83E+06	9,58E+05	529,2362
AQKQALGDNL	A0A8M2BIB6	1057,56365	7,82E+05	0,00E+00	0,00E+00	0,00E+00	7,21E+05	7,62E+04	529,2870
EPVWAIGTGK	Q1MTI4	1057,56767	2,22E+06	9,58E+04	2,05E+06	5,27E+05	2,04E+06	2,10E+05	529,2890
RLQGEVEDL	Q90339	1058,54766	7,15E+06	1,94E+06	3,22E+06	1,21E+06	3,69E+06	1,34E+06	529,7793
NLQQEISDL	Q90339	1059,53168	6,53E+06	8,46E+04	4,66E+06	2,47E+05	7,82E+06	2,09E+05	530,2713
HHTFYNEL	P83750	1060,48467	3,32E+06	0,00E+00	1,43E+06	0,00E+00	2,11E+06	0,00E+00	530,7477
ALEEAEGTLE	Q90339	1061,49971	1,23E+06	9,94E+05	1,45E+06	1,00E+06	6,04E+05	7,01E+05	531,2553
FIGMESAGIH	P53479	1061,50844	2,02E+06	6,77E+04	1,46E+06	8,54E+04	1,29E+06	5,76E+04	531,2591

NASVIPEGQF	Q90339	1061,52620	8,29E+06	5,02E+05	9,04E+06	2,03E+06	6,70E+06	9,51E+05	531,2687
KLNVDPDNF	P82316	1061,52620	2,94E+06	2,96E+05	2,57E+06	5,63E+05	3,43E+06	6,17E+05	531,2686
IVYPWTQR	P82316	1062,57309	3,00E+06	0,00E+00	1,32E+06	0,00E+00	1,96E+06	0,00E+00	531,7916
FTPNLKVDM	A1A5H6	1064,54449	9,73E+06	2,72E+04	3,28E+06	0,00E+00	6,08E+06	6,89E+04	532,7773
LTAGFMQALL	F1QCV2	1064,58088	5,59E+06	1,12E+05	3,94E+06	4,70E+05	4,22E+06	2,62E+05	532,7921
KPDQLGPAEL	Q5U3J8	1067,57315	2,46E+05	1,62E+06	5,56E+05	1,96E+06	6,18E+05	3,39E+06	534,2926
RVPTPNVSVV	Q5XJ10	1067,62077	6,33E+06	0,00E+00	8,03E+06	1,01E+05	5,79E+06	0,00E+00	534,3156
MYPGIADRM	P83750	1069,48051	2,36E+06	0,00E+00	3,19E+06	0,00E+00	4,35E+06	0,00E+00	535,2454
SGGTTMYPGIA	P83750	1070,48229	1,21E+06	1,82E+06	7,65E+05	4,35E+06	0,00E+00	2,62E+06	535,7464
ALGQNPTNKE	Q6P0G6	1071,54291	0,00E+00	1,85E+06	9,21E+03	8,04E+06	0,00E+00	3,63E+06	536,2766
RKLEGDLKL	Q90339	1071,65207	8,10E+06	0,00E+00	3,25E+06	0,00E+00	8,53E+06	0,00E+00	357,8900
GEQIDNLQR	Q90339	1072,53816	7,32E+05	1,13E+07	1,18E+06	1,04E+07	9,07E+05	9,98E+06	536,7745
QRLQGEVED	Q90339	1073,52218	2,64E+05	0,00E+00	1,09E+04	0,00E+00	1,23E+05	0,00E+00	537,2662
VTVGTPNTATI	Q6JAN0	1073,58372	2,17E+06	1,03E+06	2,09E+06	1,49E+06	1,86E+06	1,02E+06	537,2958
LTEQLGETGK	Q90339	1075,56298	6,93E+05	3,81E+06	4,35E+05	1,98E+06	5,55E+05	4,04E+06	538,2868
ESAGIHETAY	P53479	1077,48473	5,29E+06	5,44E+06	2,66E+06	3,61E+06	2,55E+06	3,79E+06	539,2481
KTIDDLEDE	P13104	1077,49463	5,70E+06	3,62E+07	7,89E+06	2,66E+07	5,61E+06	3,30E+07	539,2529
FIGMESAGIH	P53479	1077,50336	3,02E+05	0,00E+00	6,14E+05	9,65E+05	2,67E+05	5,78E+05	539,2567
PDVAGNVDYK	Q66I73	1077,52111	4,44E+05	3,15E+05	3,00E+05	3,81E+05	2,69E+05	2,99E+05	539,2656
AFTIIDQNR	Q66I73	1077,56873	2,56E+07	1,29E+06	1,23E+07	3,72E+06	1,77E+07	1,95E+06	539,2895
EGLQPRPVAL	Q6JAN0	1079,62077	1,64E+06	0,00E+00	2,72E+06	1,81E+05	1,71E+06	1,02E+05	540,3154
DEAGPSIVHR	P53479	1080,54325	3,63E+05	1,06E+07	8,75E+05	8,59E+06	5,05E+05	8,69E+06	540,7765
RDYIWNTL	Q7ZVY5	1080,54727	1,49E+05	0,00E+00	3,21E+05	0,00E+00	2,10E+05	0,00E+00	540,7794
AFFVIDQDK	Q9I8V0	1082,55169	6,92E+05	0,00E+00	1,22E+05	0,00E+00	2,82E+05	0,00E+00	541,7807
SYVGDEAQSK	P83750	1083,49529	6,23E+04	1,05E+07	2,57E+05	1,18E+07	4,60E+04	1,28E+07	542,2527
LHVDPDNFK	Q90485	1084,54218	1,06E+06	8,13E+04	4,41E+05	1,31E+05	9,16E+05	1,16E+05	362,1867
KLHVDPDNF	Q90485	1084,54218	2,59E+07	2,44E+05	2,10E+07	1,39E+05	3,19E+07	8,65E+04	542,7765
ELPDGQVITI	P83750	1084,58847	2,83E+06	7,77E+04	6,01E+06	4,23E+05	6,56E+06	2,81E+05	542,7996
YDEAGPSIVH	P53479	1087,50546	1,32E+06	6,98E+06	3,33E+06	7,00E+06	1,71E+06	7,15E+06	544,2586
KLEQQVDDL	Q90339	1087,56298	8,97E+06	3,57E+06	6,51E+06	2,56E+06	6,58E+06	3,43E+06	544,2871
WFDNQIHE	P25489	1088,47958	7,31E+05	0,00E+00	1,07E+06	0,00E+00	8,66E+05	0,00E+00	544,7454
LEQQVDDLE	Q90339	1088,51061	6,70E+06	5,66E+06	1,06E+07	6,96E+06	6,24E+06	6,65E+06	544,7611
KKDIDDLEL	Q90339	1088,58338	1,66E+06	0,00E+00	2,75E+05	0,00E+00	1,24E+06	0,00E+00	544,7969

IEDEQSLGAQ	Q90339	1089,50586	0,00E+00	4,96E+07	0,00E+00	5,31E+07	0,00E+00	4,49E+07	545,2584
KIEDEQSLGA	Q90339	1089,54224	1,46E+06	1,35E+07	2,06E+06	1,38E+07	1,58E+06	1,41E+07	545,2762
QYYTVFDR	Q9DEX3	1091,51564	1,19E+08	2,55E+06	2,14E+08	2,08E+07	1,37E+08	7,94E+06	546,2629
DTDSEEEIR	Q6PI52	1093,46439	2,90E+05	1,39E+06	2,69E+05	3,13E+06	2,69E+05	2,77E+06	547,2379
GFIEEEELK	Q804W2	1093,54118	1,77E+06	8,57E+05	6,88E+05	3,66E+05	1,16E+06	6,60E+05	547,2759
HLPNDPMFK	Q7ZVY5	1098,54008	4,00E+05	0,00E+00	4,92E+05	0,00E+00	1,62E+05	0,00E+00	549,7752
PERTSLSPIT	A0A2R8QCI3	1100,59461	2,09E+06	2,25E+05	3,28E+06	6,03E+05	2,50E+06	4,01E+05	550,8026
AADILAERLE	Q1LX78	1100,59461	2,09E+06	2,25E+05	3,28E+06	6,03E+05	2,50E+06	4,01E+05	550,8025
AELGEQIDNL	Q90339	1101,54224	2,34E+06	0,00E+00	3,37E+06	2,56E+05	2,39E+06	1,09E+05	551,2760
VAPEEHPTLL	P53479	1105,58880	1,06E+08	2,67E+07	1,43E+08	5,91E+07	2,41E+08	1,05E+08	553,2996
ISAPSADAPMF	Q5XJ10	1106,51867	7,07E+05	0,00E+00	8,55E+05	0,00E+00	3,26E+05	0,00E+00	553,7644
KTMVGNVETL	Q5RFW0	1107,57144	5,53E+06	7,11E+05	4,43E+06	1,25E+06	8,99E+06	1,63E+06	554,2889
LSDHHVYLE	P53448	1112,53710	3,64E+05	1,78E+05	3,82E+05	1,28E+05	3,84E+05	1,18E+05	556,7744
FRVPTPNVSV	Q5XJ10	1115,62077	5,20E+06	0,00E+00	1,06E+06	0,00E+00	3,32E+06	3,07E+04	558,3153
DHALNDMTSI	P13104	1116,49900	3,97E+06	4,53E+05	4,72E+06	5,03E+05	3,14E+06	5,55E+05	558,7546
NALDRAEQAE	P13104	1116,52799	1,58E+06	1,15E+06	1,48E+06	1,01E+06	2,01E+06	1,35E+06	558,7689
LGDAENALKDA	Q7ZU45	1116,55314	1,31E+06	6,85E+04	0,00E+00	0,00E+00	6,54E+05	0,00E+00	558,7820
SYELPDGQVI	P83750	1120,55208	8,89E+06	7,54E+04	1,60E+07	6,12E+05	9,22E+06	2,35E+05	560,7812
ILPDGDHDLK	P53448	1122,57896	1,14E+07	6,12E+07	1,47E+07	5,87E+07	1,04E+07	5,09E+07	374,8658
IVVIGHVDSGK	Q92005	1123,64698	8,61E+05	6,84E+04	2,06E+06	0,00E+00	1,07E+06	0,00E+00	375,2210
PGMRPPMGGPM	Q8JGS0	1127,51585	1,12E+06	0,00E+00	2,00E+06	0,00E+00	1,70E+06	2,10E+04	564,2575
VFPSIVGRPR	P83750	1127,66839	4,24E+06	7,52E+04	1,43E+06	3,28E+05	2,56E+06	1,68E+05	376,5619
INLASANTVIL	B0R061	1128,66230	3,59E+07	1,80E+04	2,38E+07	0,00E+00	6,13E+07	0,00E+00	564,8381
GYSFVTTAER	P53479	1130,54766	7,52E+05	6,70E+05	3,71E+05	2,36E+06	1,13E+06	5,72E+05	565,7790
RDGIISKDDL	Q66I73	1131,60043	4,51E+06	1,89E+05	0,00E+00	1,38E+05	0,00E+00	1,71E+05	566,3044
YELPDGQVIT	P83750	1134,56773	3,54E+07	3,16E+06	2,17E+07	3,77E+06	1,86E+07	3,18E+06	567,7888
KSYELPDGQV	P83750	1135,56298	1,95E+07	9,15E+06	1,32E+07	7,88E+06	1,51E+07	9,65E+06	568,2866
NALAHAVQSAR	Q90339	1137,61233	3,56E+05	0,00E+00	5,30E+04	0,00E+00	1,76E+05	0,00E+00	379,8767
NVIGEPIDER	Q9PTY0	1141,58478	5,08E+05	1,63E+05	4,43E+05	2,00E+05	4,08E+05	1,72E+05	571,2972
ELPDGQVITIG	P83750	1141,60993	1,71E+07	7,04E+05	2,71E+07	2,92E+06	2,17E+07	1,61E+06	571,3103
EEAEGTLEHE	Q90339	1143,48004	7,40E+05	1,15E+07	8,08E+05	1,20E+07	6,65E+05	1,04E+07	572,2450
IGMESAGIHET	P53479	1144,53030	1,86E+06	1,45E+06	5,98E+05	4,54E+05	7,12E+05	6,29E+05	572,7697
WAAFPPDVAGN	Q66I73	1144,54218	3,00E+05	0,00E+00	2,17E+05	0,00E+00	4,41E+05	0,00E+00	572,7759

LDKENALDRA	P13104	1144,59568	4,80E+04	0,00E+00	0,00E+00	0,00E+00	8,51E+04	7,43E+05	572,8029
LEQQVDDLEG	Q90339	1145,53207	9,73E+05	9,98E+05	9,41E+05	1,76E+06	6,32E+05	1,06E+06	573,2710
RLEDEEEIN	Q90339	1146,52732	2,87E+05	1,16E+06	2,44E+05	1,14E+06	2,65E+05	1,17E+06	573,7691
QAEEDKVNTL	Q90339	1146,56371	4,97E+05	4,17E+06	7,90E+05	5,33E+06	7,81E+05	6,21E+06	573,7871
RVAPEEHPVL	P83750	1146,62658	1,34E+07	3,44E+06	2,07E+07	6,12E+06	2,92E+07	1,02E+07	382,8817
RVAPEEHPTL	P53479	1148,60585	1,05E+08	8,21E+07	2,35E+08	1,16E+08	1,72E+08	1,71E+08	383,5417
TMYPGIADRM	P83750	1154,53328	2,38E+06	1,02E+05	1,72E+06	0,00E+00	2,51E+06	1,17E+05	577,7714
PKDAAANRAIAG	Q5U3G1	1154,62765	1,28E+06	1,33E+06	1,23E+06	1,16E+06	8,55E+05	9,13E+05	577,8187
VHKTMVQALQ	Q7SXG4-2	1154,63504	1,28E+06	1,33E+06	1,23E+06	1,16E+06	8,55E+05	9,13E+05	577,8187
FVIDQDKSGF	Q9I8V0	1155,56807	2,33E+06	1,72E+05	3,04E+05	1,67E+05	1,03E+06	1,35E+05	578,2901
DKENALDRAE	P13104	1160,55421	1,04E+04	8,28E+05	2,45E+04	6,19E+05	4,10E+03	8,08E+05	580,7816
VLDPDEGIRF	Q7ZVY5	1160,59461	1,56E+06	0,00E+00	1,22E+06	8,01E+04	9,61E+05	4,95E+04	580,8027
LSKIEDEQSL	Q90339	1161,59976	1,39E+06	6,26E+05	5,12E+05	5,43E+05	1,33E+06	8,21E+05	581,3047
TALEEAEGTLE	Q90339	1162,54739	4,18E+06	3,04E+05	5,19E+06	6,14E+05	4,09E+06	4,87E+05	581,7792
LFETQPLDSI	Q5PZ43	1162,59903	3,33E+06	7,59E+04	3,00E+06	1,92E+05	3,21E+06	1,60E+05	581,8048
SGFIEEDELK	P02618	1166,55756	5,86E+05	1,47E+05	1,45E+05	9,91E+04	2,17E+05	9,41E+04	583,7842
LDNFPTNLHP	Q7ZVY5	1167,57930	1,17E+06	0,00E+00	1,50E+06	0,00E+00	4,44E+05	0,00E+00	584,2952
FGLHHLGMQI	Q1LX78	1168,59317	3,52E+06	2,24E+05	2,75E+06	5,08E+05	2,65E+06	3,55E+05	584,8052
VGLLHSQNTSL	Q90339	1168,63206	4,82E+06	2,60E+05	6,30E+05	2,52E+05	2,72E+06	2,76E+05	584,8209
KYPIEHGIIT	P53479	1170,65174	2,36E+06	1,42E+05	2,47E+06	1,47E+05	2,99E+06	0,00E+00	585,8318
VIISAPSADAPM	Q5XJ10	1171,60274	1,11E+07	3,09E+05	8,46E+06	5,99E+05	2,62E+06	1,93E+05	586,3064
NHSFMPIVVE	Q9MIY7	1172,57686	9,20E+05	0,00E+00	7,34E+05	0,00E+00	8,90E+05	0,00E+00	586,7938
SKIEDEQSLGA	Q90339	1176,57427	5,77E+06	1,01E+07	7,44E+06	1,08E+07	5,92E+06	1,39E+07	588,7927
DGAITEQMAEL	B0R061	1177,54053	1,71E+06	5,75E+06	1,53E+06	5,64E+06	1,14E+06	4,51E+06	589,2740
SGFIEEEELK	Q804W2	1180,57321	5,70E+07	6,50E+06	1,74E+07	5,47E+06	3,23E+07	5,72E+06	590,7918
GTTMYPGIADR	P83750	1181,56193	1,06E+06	4,75E+05	1,23E+06	3,26E+05	8,78E+05	4,12E+05	591,2866
ALSDHHVYLE	P53448	1183,57421	1,20E+06	0,00E+00	1,12E+06	0,00E+00	1,18E+06	0,00E+00	592,2926
GPGMRPPMGGPM	Q8JGS0	1184,53732	1,15E+06	5,46E+04	2,46E+06	0,00E+00	1,47E+06	8,62E+04	592,7680
DNFPTNLHPM	Q7ZVY5	1185,53572	1,05E+06	0,00E+00	2,06E+06	0,00E+00	1,44E+06	1,02E+05	593,2723
QRLQGEVEDL	Q90339	1186,60624	4,56E+07	0,00E+00	3,48E+06	0,00E+00	2,48E+07	0,00E+00	593,8084
AFRVPTPNVSV	Q5XJ10	1186,65788	3,16E+06	0,00E+00	0,00E+00	0,00E+00	1,07E+06	0,00E+00	593,8345
TKLEQQVDDL	Q90339	1188,61066	8,83E+07	5,79E+06	6,05E+07	1,08E+07	8,50E+07	1,10E+07	594,8107
DLTEQLGETGK	Q90339	1190,58992	2,85E+06	3,13E+06	2,31E+06	2,64E+06	2,25E+06	2,60E+06	595,8005

ESAGIHETAYN	P53479	1191,52766	4,17E+05	9,32E+04	2,76E+05	1,21E+05	2,43E+05	7,60E+04	596,2684
FPPDVAGNVDY	Q66I73	1193,54733	4,05E+06	5,18E+05	3,26E+06	8,99E+05	1,33E+06	3,90E+05	597,2783
VFDKEGNGTVM	Q6P0G6	1196,56160	5,53E+06	9,43E+06	4,86E+06	3,72E+06	4,73E+06	8,23E+06	598,7861
RVIISAPSADAP	Q5XJ10	1196,66336	3,10E+06	8,11E+04	1,91E+06	2,44E+05	2,14E+05	0,00E+00	598,8367
DSYVGDEAQSK	P83750	1198,52224	9,13E+06	4,79E+08	1,44E+07	3,67E+08	1,14E+07	4,29E+08	599,7662
MEIQLSHANR	Q90339	1198,59972	4,93E+05	0,00E+00	0,00E+00	0,00E+00	2,49E+05	0,00E+00	599,8049
ELVQQNNLLE	P85001	1199,62664	4,34E+05	0,00E+00	6,05E+05	0,00E+00	4,15E+05	0,00E+00	600,3202
DLEESTLQHE	Q90339	1200,53789	5,12E+05	1,12E+06	3,49E+05	7,61E+05	5,26E+05	1,10E+06	600,7736
IEDEQSLGAQL	Q90339	1202,58992	4,71E+06	1,74E+06	4,01E+06	1,91E+06	3,89E+06	2,43E+06	601,8002
EKTIDDLEDE	P13104	1206,53722	2,18E+07	3,23E+07	1,79E+07	2,29E+07	1,73E+07	3,00E+07	603,7742
EAFTIIDQNR	Q66I73	1206,61133	1,24E+07	3,58E+05	3,87E+06	9,68E+05	6,32E+06	4,68E+05	603,8110
LTGMAFIDTQL	A0A0R4IC37	1209,61839	1,10E+07	0,00E+00	1,19E+07	0,00E+00	1,87E+07	0,00E+00	605,3110
VFDKEGNGTVM	Q6P0G6	1212,55651	1,55E+05	4,20E+06	2,39E+05	4,34E+06	2,29E+05	4,92E+06	606,7836
KLHVDPDNFK	Q90485	1212,63715	2,21E+06	4,03E+05	7,76E+05	2,68E+05	1,86E+06	5,79E+05	606,8235
EKLHVDPDNF	Q90485	1213,58478	9,41E+06	3,31E+05	5,02E+06	4,13E+05	6,78E+06	4,79E+05	607,2978
EYDEAGPSIVH	P53479	1216,54806	8,42E+06	1,94E+07	1,95E+07	2,38E+07	1,12E+07	2,43E+07	608,7795
KIEDEQSLGAQ	Q90339	1217,60082	5,50E+06	4,99E+07	6,89E+06	4,34E+07	4,96E+06	5,32E+07	609,3057
IISAPSADAPMF	Q5XJ10	1219,60274	2,17E+06	0,00E+00	1,42E+06	0,00E+00	8,80E+05	0,00E+00	610,3064
PEILPDGDHDL	P53448	1220,57936	1,07E+06	1,23E+05	6,78E+05	8,51E+04	6,47E+05	1,24E+05	610,7946
SYELPDGQVIT	P83750	1221,59976	1,69E+08	6,40E+06	1,88E+08	1,98E+07	1,17E+08	1,16E+07	611,3054
SLVHYAGTVDY	Q90339	1224,58953	1,81E+06	0,00E+00	2,42E+05	0,00E+00	6,54E+05	0,00E+00	612,8003
DLQHRLDEAE	Q90339	1225,58076	2,33E+05	0,00E+00	3,03E+05	0,00E+00	2,08E+05	1,05E+05	613,2965
VDIGIPDATGRL	Q7ZU99	1226,67393	3,89E+05	0,00E+00	2,13E+05	0,00E+00	2,95E+05	0,00E+00	613,8419
DNYRPPQPLK	Q92051	1227,64805	1,81E+05	3,45E+05	2,35E+05	2,72E+05	1,69E+05	3,58E+05	614,3295
FVPISGWHGDN	Q92005	1228,57455	6,18E+05	0,00E+00	4,34E+05	6,22E+04	4,74E+05	0,00E+00	614,7930
LDHALNDMTSI	P13104	1229,58306	3,72E+06	0,00E+00	4,19E+06	0,00E+00	5,83E+06	0,00E+00	615,2968
EYDESGPSIVH	P83750	1232,54297	1,82E+05	6,12E+05	3,89E+05	6,98E+05	2,15E+05	7,01E+05	616,7761
GIHETAYNSIM	P53479	1235,57250	4,51E+06	0,00E+00	2,18E+06	1,24E+05	2,56E+06	6,76E+04	618,2909
GGTTMYPGIADR	P83750	1238,58340	2,41E+06	2,37E+06	2,97E+06	1,15E+06	1,85E+06	1,74E+06	619,7971
YDEAGPSIVHR	P53479	1243,60658	8,24E+06	3,41E+07	2,02E+07	3,06E+07	1,16E+07	3,42E+07	622,3080
KQLQDEGIDAE	F1RE08	1245,59574	3,33E+06	1,83E+06	9,81E+05	8,67E+05	1,65E+06	1,52E+06	623,3028
LGDAENALKDAE	Q7ZU45	1245,59574	3,33E+06	1,83E+06	9,81E+05	8,67E+05	1,65E+06	1,52E+06	623,3024
VASDIVEAAGGSGL	H6D7E6	1245,63212	1,40E+06	0,00E+00	2,01E+06	0,00E+00	1,04E+06	0,00E+00	623,3204

DGIISKDDLRD	Q66I73	1246,62737	2,16E+05	1,53E+05	0,00E+00	1,18E+05	0,00E+00	8,83E+04	416,2151
GIYPAVDPLDST	Q9PTY0	1247,61541	4,65E+05	0,00E+00	2,60E+05	0,00E+00	2,06E+05	0,00E+00	624,3120
SVLDPDEGIRF	Q7ZVY5	1247,62664	1,06E+06	0,00E+00	1,03E+06	0,00E+00	7,37E+05	0,00E+00	624,3182
YELPDGQVITI	P83750	1247,65179	1,62E+06	0,00E+00	1,59E+06	0,00E+00	2,20E+06	0,00E+00	624,3311
KSYELPDGQVI	P83750	1248,64704	1,65E+07	0,00E+00	1,57E+07	0,00E+00	1,63E+07	0,00E+00	624,8281
GIHETAYNSIM	P53479	1251,56741	1,43E+06	7,26E+06	2,53E+06	1,02E+07	1,87E+06	6,38E+06	626,2883
DLAGRDLTDYL	P83750	1251,62156	5,64E+05	0,00E+00	4,63E+05	0,00E+00	4,33E+05	0,00E+00	626,3157
GTLEHEESKIL	Q90339	1255,65286	7,00E+05	8,04E+04	6,45E+04	0,00E+00	6,93E+05	6,44E+04	628,3315
LEEAEGTLEHE	Q90339	1256,56410	3,74E+05	1,18E+06	4,61E+05	8,74E+05	2,97E+05	1,09E+06	628,7868
EELALRAEEAE	P85001	1259,61139	1,81E+06	7,45E+05	6,07E+05	4,26E+05	1,38E+06	6,74E+05	630,3112
RVAPEEHPTLL	P53479	1261,68991	2,80E+08	1,62E+07	4,26E+08	6,53E+07	4,91E+08	7,27E+07	631,3500
DDLTDPAPATTF	Q9PTY0	1263,57394	8,01E+05	0,00E+00	1,16E+06	0,00E+00	7,54E+05	3,54E+04	632,2936
IIDQDKSGFIE	P02618	1264,64196	3,27E+06	1,07E+06	8,58E+05	7,80E+05	1,81E+06	9,47E+05	632,8265
SDHHVYLEGTL	P53448	1270,60624	9,44E+06	0,00E+00	7,12E+06	0,00E+00	7,28E+06	0,00E+00	424,2085
EEAEGTLEHEE	Q90339	1272,52263	7,35E+05	6,73E+06	8,58E+05	6,01E+06	6,27E+05	5,83E+06	636,7658
SEELDHALNDM	P13104	1273,53651	1,28E+06	0,00E+00	3,02E+06	0,00E+00	2,55E+06	4,80E+04	637,2728
VLIVYPWTQR	P82316	1274,72557	3,42E+05	0,00E+00	6,48E+05	1,16E+05	3,72E+05	1,65E+04	637,8677
ASGDIDRDAWAV	Q7SYK7	1275,59641	4,68E+05	4,94E+05	6,65E+05	9,21E+05	3,66E+05	4,20E+05	638,3038
SDLTEQLGETGK	Q90339	1277,62195	1,65E+06	1,37E+06	8,92E+05	8,46E+05	9,20E+05	8,96E+05	639,3163
ILPDGDHDLKR	P53448	1278,68008	5,67E+04	2,16E+05	6,31E+04	1,27E+05	5,13E+04	2,17E+05	426,8996
VFDKDGNGYISA	Q6PI52	1285,60591	1,32E+06	3,34E+05	1,05E+05	0,00E+00	4,66E+05	1,57E+05	643,3074
DVLATMGQLNVK	Q66I73	1288,69295	3,52E+05	0,00E+00	8,14E+04	0,00E+00	2,35E+05	0,00E+00	644,8511
SGFIEEEELKL	P05939	1293,65727	8,71E+05	0,00E+00	4,22E+05	0,00E+00	1,60E+06	0,00E+00	647,3344
LDNFPTNLHPM	Q7ZVY5	1298,61978	1,29E+06	0,00E+00	2,10E+06	3,31E+05	1,33E+06	1,86E+05	649,8155
GANHSFMPIVVE	Q9MIY7	1300,63543	2,66E+05	0,00E+00	2,70E+05	0,00E+00	2,01E+05	0,00E+00	650,8226
RIPSAVGYQPTL	Q9PTY0	1301,72121	6,28E+05	3,14E+04	5,91E+05	1,07E+05	4,65E+05	3,02E+04	651,3658
SKIEDEQSLGAQ	Q90339	1304,63285	1,89E+07	5,22E+07	2,06E+07	5,41E+07	2,18E+07	6,72E+07	652,8223
NMGMNINQQQQ	Q67FY3	1305,56743	1,07E+06	1,22E+07	1,29E+06	3,91E+06	1,07E+06	6,64E+06	653,2888
GTTMYPGIADRM	P83750	1312,60242	5,02E+06	0,00E+00	3,27E+06	0,00E+00	5,22E+06	0,00E+00	656,8063
PVADFFPIQSPP	F1QCY8	1314,67286	8,66E+05	1,29E+07	1,56E+06	4,45E+07	1,01E+06	1,04E+07	657,8369
TKLEQQVDDLE	Q90339	1317,65325	3,03E+07	1,53E+06	2,09E+07	3,22E+06	2,96E+07	3,12E+06	659,3312
VIISAPSADAPMF	Q5XJ10	1318,67115	3,23E+07	5,98E+04	1,09E+07	2,06E+05	1,56E+07	8,30E+04	659,8409
KDKELNDTLSR	P85001	1318,69612	2,54E+07	0,00E+00	5,00E+06	0,00E+00	1,51E+07	0,00E+00	659,8554

FPPDVAGNVDYK	Q66I73	1321,64229	3,27E+07	5,38E+05	2,12E+07	2,67E+06	2,11E+07	7,20E+05	661,3267
DTGAPIRIPVGPE	Q9PTY0	1321,71104	7,64E+05	3,37E+04	6,35E+05	0,00E+00	5,79E+05	2,59E+04	661,3594
LYSADGEAQQLE	Q5BLC7	1323,60630	6,05E+06	1,66E+07	3,40E+06	1,08E+07	4,09E+06	1,76E+07	662,3087
RVGLLHSQNTSL	Q90339	1324,73317	5,15E+06	5,18E+04	8,52E+05	9,48E+04	3,21E+06	7,63E+04	442,2508
SGGTTMYPGIADR	P83750	1325,61543	2,03E+07	8,35E+06	2,88E+07	6,43E+06	1,87E+07	8,34E+06	663,3134
AISEELDHALND	P13104	1326,61720	4,89E+05	4,93E+04	3,93E+05	0,00E+00	9,36E+05	3,76E+04	663,8138
ALEEAEGTLEHE	Q90339	1327,60122	1,27E+06	4,31E+05	1,23E+06	3,72E+05	9,10E+05	3,80E+05	664,3073
RVIISAPSADAPM	Q5XJ10	1327,70385	2,91E+07	0,00E+00	2,04E+07	0,00E+00	7,56E+06	0,00E+00	664,3574
IEDEQSLGAQLQ	Q90339	1330,64850	1,44E+06	5,10E+05	1,16E+06	5,59E+05	1,46E+06	7,90E+05	665,8282
KIEDEQSLGAQL	Q90339	1330,68489	1,73E+07	1,60E+06	1,63E+07	2,17E+06	1,78E+07	2,90E+06	665,8469
VIISAPSADAPMF	Q5XJ10	1334,66606	1,68E+06	1,71E+04	1,40E+06	1,89E+05	8,54E+05	7,21E+04	667,8384
SYELPDGQVITI	P83750	1334,68382	1,08E+07	0,00E+00	1,28E+07	1,92E+05	1,90E+07	7,35E+04	667,8469
GKDLEILEYQQ	P85001	1335,67907	1,43E+06	2,43E+05	9,22E+05	2,11E+05	1,10E+06	3,64E+05	668,3452
HGDSSVPVWSGVN	Q9W7K5	1340,62295	5,67E+05	5,91E+04	8,47E+05	8,50E+04	4,59E+05	6,99E+04	670,8161
SGGTTMYPGIADR	P83750	1341,61034	1,29E+06	3,51E+06	2,50E+06	6,03E+06	2,11E+06	4,35E+06	671,3106
EKLHVDPDNFK	Q90485	1341,67974	9,80E+05	1,92E+05	3,06E+05	1,37E+05	6,54E+05	2,14E+05	447,8994
AEGTLEHEESKI	Q90339	1342,64850	4,74E+05	0,00E+00	1,61E+05	0,00E+00	2,84E+05	0,00E+00	671,8295
RVIISAPSADAPM	Q5XJ10	1343,69876	9,23E+05	1,07E+05	1,06E+06	5,78E+05	3,19E+05	7,38E+04	672,3547
TIIDQNRDGIIS	Q66I73	1344,71177	3,76E+06	0,00E+00	5,51E+06	0,00E+00	3,58E+06	0,00E+00	672,8616
LEQQVDDLEGSL	Q90339	1345,64817	2,22E+07	2,96E+05	1,36E+07	1,05E+06	1,70E+07	5,96E+05	673,3287
GADPEDVIVSAFK	Q66I73	1347,67907	1,76E+06	0,00E+00	2,08E+06	1,04E+05	9,28E+05	0,00E+00	674,3440
PEILPDGDHDLK	P53448	1348,67432	6,71E+07	1,10E+07	2,10E+07	9,45E+06	3,89E+07	1,02E+07	450,2308
TSVLDPDEGIRF	Q7ZVY5	1348,67432	1,83E+05	0,00E+00	1,23E+05	0,00E+00	1,19E+05	0,00E+00	674,8420
KSYELPDGQVIT	P83750	1349,69472	1,87E+08	1,34E+07	1,64E+08	2,51E+07	1,53E+08	2,54E+07	675,3524
RVFDKEGNGTVM	Q6P0G6	1352,66271	9,24E+06	6,87E+05	7,36E+06	3,56E+05	1,12E+07	9,61E+05	451,5603
DSYVGDEAQSKR	P83750	1354,62335	7,99E+06	3,79E+08	4,56E+06	1,30E+08	7,19E+06	2,61E+08	452,2140
VDIGIPDATGRLE	Q7ZU99	1355,71652	5,87E+05	0,00E+00	3,62E+05	0,00E+00	3,64E+05	0,00E+00	678,3620
WLDKNKDPLND	Q90339	1357,67466	3,56E+06	4,31E+05	1,33E+06	1,70E+05	1,45E+06	2,94E+05	679,3431
DKENALDRAEQA	P13104	1359,64990	3,86E+05	4,71E+05	0,00E+00	2,04E+05	4,03E+05	4,47E+05	680,3290
EQQVDDLEGSLE	Q90339	1361,60669	4,64E+06	1,30E+06	4,68E+06	2,05E+06	2,53E+06	1,24E+06	681,3081
VDDLEGSLEQEK	Q90339	1361,64308	2,08E+05	5,12E+04	0,00E+00	0,00E+00	0,00E+00	0,00E+00	681,3279
GGTTMYPGIADRM	P83750	1369,62388	9,68E+06	5,71E+04	6,55E+06	8,52E+04	6,66E+06	3,63E+04	685,3180
IGIDEFEALVHE	Q804W2	1371,67907	1,38E+06	0,00E+00	2,68E+05	0,00E+00	9,23E+05	0,00E+00	686,3443

DLEESTLQHEAT	Q90339	1372,62268	6,16E+05	4,15E+05	4,03E+05	2,56E+05	5,84E+05	4,61E+05	686,8172
EYDEAGPSIVHR	P53479	1372,64917	9,21E+07	1,36E+08	1,80E+08	1,38E+08	9,64E+07	1,50E+08	686,8302
KIWHHTFYNE	P83750	1374,65895	3,67E+05	0,00E+00	8,01E+04	0,00E+00	1,18E+05	0,00E+00	458,8925
TKLEQQVDDLEG	Q90339	1374,67471	6,59E+06	3,40E+05	3,49E+06	5,48E+05	5,40E+06	5,67E+05	687,8426
EKSYELPDGQVI	P83750	1377,68964	4,75E+05	0,00E+00	0,00E+00	0,00E+00	3,98E+05	0,00E+00	689,3503
IGMESAGIHETAY	P53479	1378,63074	5,49E+06	1,62E+05	6,07E+05	0,00E+00	1,70E+06	1,16E+05	689,8210
NVLSGGTTMYPGIA	P53479	1380,68278	7,60E+07	3,23E+05	4,86E+07	1,36E+06	4,84E+07	5,07E+05	690,8464
VLSGGTTMYPGIAD	P83750	1381,66679	9,56E+05	0,00E+00	2,36E+05	0,00E+00	3,60E+05	0,00E+00	691,3386
VIGEHGDSSVPVW	Q9W7K5	1381,67466	7,03E+05	0,00E+00	6,20E+05	0,00E+00	4,38E+05	0,00E+00	691,3435
SDHHVYLEGTLL	P53448	1383,69031	1,27E+06	0,00E+00	3,00E+05	0,00E+00	1,31E+06	0,00E+00	461,9040
GQPQVQGQGGAVQM	Q7ZVN7	1384,66377	1,02E+06	2,95E+06	1,63E+06	3,27E+06	1,40E+06	2,94E+06	692,8348
ELPDGQVITIGNE	P83750	1384,69545	2,44E+07	1,01E+06	2,69E+07	2,92E+06	2,28E+07	1,81E+06	692,8526
LEEAEGTLEHEE	Q90339	1385,60669	2,51E+05	1,06E+06	3,00E+05	0,00E+00	3,16E+05	1,02E+06	693,3090
GASQNIIPASTGAAK	Q5XJ10	1385,73832	4,34E+05	6,30E+05	0,00E+00	3,76E+05	4,60E+05	5,11E+05	693,3737
EYDESGPSIVHR	P83750	1388,64408	1,59E+06	4,07E+06	3,38E+06	3,93E+06	2,06E+06	4,16E+06	463,5549
ISDLTEQLGETGK	Q90339	1390,70602	6,30E+05	6,91E+04	1,70E+05	0,00E+00	3,80E+05	1,35E+05	695,8581
SYELPDGQVITIG	P83750	1391,70529	5,00E+07	1,15E+05	5,21E+07	1,07E+06	4,59E+07	3,12E+05	696,3585
AFPPDVAGNVDYK	Q66I73	1392,67941	5,53E+05	0,00E+00	8,35E+05	0,00E+00	1,01E+06	0,00E+00	696,8448
SAGIHETAYNSIM	P53479	1393,64164	1,59E+06	0,00E+00	6,02E+05	7,41E+05	7,41E+05	0,00E+00	697,3262
IGMESAGIHETAY	P53479	1394,62566	0,00E+00	0,00E+00	0,00E+00	0,00E+00	0,00E+00	0,00E+00	697,8192
NVLSGGTTMYPGIA	P53479	1396,67769	2,73E+06	2,22E+05	3,46E+06	2,07E+06	2,34E+06	5,14E+05	698,8447
GWLDKNKDPLND	Q90339	1414,69612	3,95E+06	0,00E+00	1,01E+06	0,00E+00	2,20E+06	0,00E+00	472,2383
SKIEDEQSLGAQL	Q90339	1417,71691	2,92E+07	2,19E+06	3,38E+07	6,99E+06	3,13E+07	4,12E+06	709,3642
LSKIEDEQSLGAQ	Q90339	1417,71691	7,84E+05	7,94E+05	1,48E+05	5,14E+05	6,27E+05	8,72E+05	709,3640
TGAPIRIPVGPETL	Q9PTY0	1420,81584	1,43E+05	0,00E+00	0,00E+00	0,00E+00	7,33E+04	0,00E+00	710,9137
DTGAPIRIPVGPET	Q9PTY0	1422,75872	4,71E+05	0,00E+00	1,48E+05	0,00E+00	1,27E+05	0,00E+00	711,8837
TALEEAEGTLEHE	Q90339	1428,64889	6,48E+06	1,53E+05	3,85E+06	2,99E+05	5,73E+06	3,14E+05	714,8295
IRPIYSNPPMNGA	Q7SYK7	1429,72565	3,96E+05	5,19E+04	3,64E+05	1,12E+05	2,71E+05	0,00E+00	715,3674
QGAGTGIPSSKPSSTG	Q7SXT7	1431,70741	8,73E+06	0,00E+00	1,01E+07	1,13E+05	1,12E+07	0,00E+00	716,3613
AGTNGETTTQGLDGL	P53448	1434,67069	5,26E+05	3,01E+05	3,91E+05	2,69E+05	3,71E+05	3,11E+05	717,8416
SHWADLSPGSGPVK	Q90487	1437,71210	4,52E+05	8,57E+05	5,39E+05	1,21E+06	3,04E+05	3,87E+05	719,3598
LSGGTTMYPGIADR	P83750	1438,69949	6,81E+05	0,00E+00	3,76E+05	0,00E+00	3,33E+05	0,00E+00	719,8541
RVFDKDGNGYISA	Q6PI52	1441,70702	1,60E+06	1,09E+05	0,00E+00	1,26E+05	5,64E+05	9,84E+04	481,2419

GVERPGTSRSGWR	X1WHY6	1444,74038	2,00E+06	0,00E+00	1,28E+06	0,00E+00	1,65E+06	0,00E+00	482,2481
AAMSKAMLKEPTH	B0R061	1446,70795	0,00E+00	0,00E+00	0,00E+00	0,00E+00	0,00E+00	0,00E+00	723,8581
AEGTLEHEESKIL	Q90339	1455,73256	1,25E+06	0,00E+00	1,96E+05	0,00E+00	1,01E+06	0,00E+00	728,3705
SGGTTMYPGIADRM	P83750	1456,65591	6,80E+07	0,00E+00	3,71E+07	0,00E+00	4,76E+07	0,00E+00	728,8339
AVINETPLPIDLY	Q9W7R3	1457,78862	3,64E+07	0,00E+00	2,74E+07	0,00E+00	3,29E+07	0,00E+00	729,3996
KIEDEQSLGAQLQ	Q90339	1458,74346	3,61E+06	3,84E+05	3,13E+06	6,01E+05	4,01E+06	7,13E+05	729,8770
LLIVMEGRGSAVNT	Q6DRD4	1459,79372	2,54E+06	0,00E+00	1,14E+06	0,00E+00	1,67E+06	0,00E+00	487,2740
TKLEQQVDDLEGS	Q90339	1461,70674	1,04E+06	4,00E+04	8,69E+05	1,51E+05	8,98E+05	1,47E+05	731,3600
GLGMGMGTGMGAGVVAP	Q6NWF6	1462,68510	3,15E+07	3,88E+07	4,71E+07	4,57E+07	2,80E+07	3,81E+07	731,8514
KSYELPDGQVITI	P83750	1462,77879	1,27E+07	2,50E+04	9,16E+06	9,71E+04	2,16E+07	5,48E+04	731,8945
DIVLDPLGGSDTHK	Q8JFV8	1466,74855	2,92E+05	0,00E+00	3,22E+05	0,00E+00	2,71E+05	0,00E+00	489,5893
EHGDSSVPVWSGVN	Q9W7K5	1469,66555	5,49E+05	0,00E+00	7,79E+05	8,87E+04	3,88E+05	0,00E+00	735,3377
LQSLPPEVGCLSGL	Q1L8Y7	1469,76684	8,85E+05	0,00E+00	1,83E+05	0,00E+00	5,09E+05	0,00E+00	735,3937
EAEGTLEHEESKI	Q90339	1471,69109	7,82E+05	7,35E+05	0,00E+00	7,82E+05	5,91E+05	7,96E+05	736,3511
SGGTTMYPGIADRM	P83750	1472,65083	3,44E+06	2,97E+05	3,64E+06	7,98E+05	4,26E+06	3,96E+05	736,8295
DAGDGVTHNVPVYE	P53479	1472,66521	9,57E+05	0,00E+00	0,00E+00	0,00E+00	0,00E+00	0,00E+00	736,8356
FVPISGWHGDNML	Q92005	1472,69910	2,89E+05	0,00E+00	1,44E+05	0,00E+00	2,35E+05	0,00E+00	736,8547
KQEYDEAGPSIVH	P53479	1472,70160	1,87E+05	1,09E+05	2,54E+05	0,00E+00	2,24E+05	1,65E+05	736,8561
RVVVSAPSPDAPMF	Q5MJ86	1472,75661	6,82E+05	0,00E+00	3,71E+05	0,00E+00	5,04E+05	0,00E+00	736,8832
KLEQQVDDLEGSL	Q90339	1473,74313	1,46E+06	0,00E+00	6,09E+05	0,00E+00	9,12E+05	0,00E+00	737,3768
LEQQVDDLEGSLE	Q90339	1474,69076	3,55E+07	5,66E+05	2,68E+07	2,45E+06	3,04E+07	8,94E+05	737,8502
RVIISAPSADAPMF	Q5XJ10	1474,77226	1,35E+07	0,00E+00	4,64E+06	0,00E+00	7,70E+06	0,00E+00	737,8912
VYVLMAFFREVT	A0A0R4IBK5	1474,77628	1,57E+06	0,00E+00	1,60E+06	0,00E+00	1,94E+06	0,00E+00	492,2658
ETSVLDPDEGIRF	Q7ZVY5	1477,71691	3,15E+05	0,00E+00	1,86E+05	0,00E+00	1,42E+05	0,00E+00	739,3643
EKSYELPDGQVIT	P83750	1478,73732	2,26E+07	3,92E+05	1,47E+06	8,46E+05	8,66E+06	4,50E+05	739,8742
KDSYVGDEAQSKR	P83750	1482,71831	0,00E+00	9,44E+05	0,00E+00	1,26E+06	0,00E+00	9,03E+05	494,9121
EEAEGTLEHEESK	Q90339	1487,64962	5,75E+05	1,36E+07	3,78E+05	7,42E+06	4,69E+05	1,12E+07	496,5563
DKENALDRAEQAE	P13104	1488,69249	4,41E+06	2,65E+06	2,70E+06	1,49E+06	3,56E+06	2,67E+06	496,9042
DSGDGVTHTVPIYE	P83750	1489,68053	5,51E+05	2,19E+05	9,35E+05	3,49E+05	5,82E+05	2,88E+05	745,3472
GIYPAVDPLDSTSR	Q9PTY0	1490,74855	8,38E+05	0,00E+00	3,59E+05	0,00E+00	3,70E+05	0,00E+00	745,8797
SYVTPLHHFRHT	Q6DRD4	1494,76006	4,82E+05	0,00E+00	1,14E+06	1,03E+05	7,47E+05	0,00E+00	498,9255
NVLSGGTTMYPGIAD	P53479	1495,70972	1,24E+07	0,00E+00	4,99E+06	1,03E+05	6,19E+06	1,70E+04	748,3599
VPTPNVSVVDLTVR	Q5XJ10	1495,84787	3,73E+05	0,00E+00	5,58E+05	0,00E+00	4,19E+05	0,00E+00	748,4304

VLNMAAQGNPNIKI	A1XQY0	1498,80462	1,31E+06	0,00E+00	1,02E+06	0,00E+00	6,90E+05	0,00E+00	500,2765
QEYDEAGPSIVHR	P53479	1500,70775	2,78E+06	2,77E+06	3,48E+06	2,45E+06	2,30E+06	2,29E+06	500,9094
EYDEAGPSIVHRK	P53479	1500,74413	1,78E+06	5,85E+06	2,20E+05	1,26E+06	1,31E+06	4,41E+06	500,9207
PEILPDGDHDLKR	P53448	1504,77543	3,33E+05	6,45E+04	1,24E+05	0,00E+00	2,15E+05	5,81E+04	376,9502
HGDSSVPVWSGVNVA	Q9W7K5	1510,72848	5,64E+05	0,00E+00	4,13E+05	0,00E+00	4,09E+05	0,00E+00	755,8711
SSQRNSTHLGDELA	Q5RFW0	1514,71937	4,19E+06	4,42E+05	5,54E+06	5,48E+05	5,05E+06	5,46E+05	505,5782
QEYDESGPSIVHR	P83750	1516,70266	6,91E+06	3,40E+06	1,66E+07	6,32E+06	9,96E+06	4,40E+06	758,8512
SMFEQSQIQEYK	Q66I73	1517,69407	2,31E+06	0,00E+00	6,44E+05	0,00E+00	1,08E+06	5,03E+04	759,3528
KSYELPDGQVITIG	P83750	1519,80025	7,13E+07	1,50E+05	3,57E+07	6,28E+05	6,11E+07	2,52E+05	760,4056
ESAGIHETAYNSIM	P53479	1522,68423	4,12E+05	0,00E+00	1,30E+05	0,00E+00	2,46E+05	0,00E+00	761,8472
QGYPAQGYPPQGYP	A5PLE2	1522,69612	4,12E+05	0,00E+00	1,30E+05	0,00E+00	2,46E+05	0,00E+00	761,8482
VIGEHGDSSVPVWSG	Q9W7K5	1525,72815	5,17E+05	0,00E+00	7,26E+05	7,56E+04	4,47E+05	0,00E+00	763,3698
LSKIEDEQSLGAQL	Q90339	1530,80098	3,81E+05	6,39E+04	7,03E+04	0,00E+00	3,01E+05	0,00E+00	765,9058
DTGAPIRIPVGPETL	Q9PTY0	1535,84278	6,73E+06	3,07E+04	1,56E+06	1,02E+05	3,82E+06	5,43E+04	768,4266
VLSGGTTMYPGIADR	P83750	1537,76790	1,49E+06	0,00E+00	0,00E+00	0,00E+00	6,32E+05	0,00E+00	769,3906
ELPDGQVITIGNER	P83750	1540,79656	2,45E+06	8,42E+04	9,21E+05	2,04E+05	1,32E+06	1,18E+05	770,9042
FIGMESAGIHETAY	P53479	1541,69407	0,00E+00	0,00E+00	0,00E+00	0,00E+00	0,00E+00	0,00E+00	771,3489
QSVSMELPQGAVGPE	A5D8S5	1544,72610	5,25E+06	9,20E+04	4,93E+06	3,34E+05	2,03E+06	1,14E+05	772,8690
SKIEDEQSLGAQLQ	Q90339	1545,77549	4,23E+06	3,54E+05	3,38E+06	1,02E+06	5,37E+06	5,22E+05	773,3931
YELPDGQVITIGNE	P83750	1547,75878	9,80E+06	2,25E+04	3,64E+06	1,45E+05	4,69E+06	6,00E+04	774,3851
TALEEAEGTLEHEE	Q90339	1557,69149	1,87E+06	3,99E+05	1,22E+06	2,17E+05	1,68E+06	2,05E+05	779,3506
YDRLPSPPVTPGME	Q8JFV8	1558,75700	3,76E+05	0,00E+00	2,14E+05	0,00E+00	2,16E+05	0,00E+00	779,8852
AFTIIDQNRDGIIS	Q66I73	1562,81730	3,58E+05	0,00E+00	2,78E+05	0,00E+00	3,47E+05	0,00E+00	781,9147
MADQLTEEQIAEFK	Q6PI52	1563,75369	8,26E+06	2,13E+05	0,00E+00	7,35E+05	4,43E+06	2,19E+05	782,3821
IREADIDGDGQVNY	Q6PI52	1564,72379	1,53E+06	7,96E+04	0,00E+00	0,00E+00	5,08E+05	5,19E+04	782,8663
WVIGEHGDSSVPVW	Q9W7K5	1567,75397	5,18E+05	0,00E+00	3,55E+05	0,00E+00	3,76E+05	0,00E+00	784,3828
TKLEQQVDDLEGSL	Q90339	1574,79081	1,91E+07	3,84E+04	4,01E+06	0,00E+00	1,33E+07	5,94E+04	787,9008
KNGSFPGDSIGMEKN	Q5PZ43	1580,73733	3,74E+06	1,03E+05	5,82E+06	4,65E+05	2,35E+06	1,47E+05	790,8776
SLSEIEESMLSALR	A0A0R4IES7	1580,78361	2,57E+06	0,00E+00	2,80E+05	0,00E+00	7,80E+05	0,00E+00	527,6011
PGQSPPNSGMGGITVPA	E7F1H9	1582,75298	4,64E+05	3,49E+06	1,15E+06	4,10E+06	5,76E+05	3,55E+06	791,8858
TIIDQNRDGIISKD	Q66I73	1587,83368	7,70E+05	0,00E+00	5,61E+05	0,00E+00	5,20E+05	0,00E+00	794,4219
EKDKTPMAEGGLNLS	Q1LX78	1589,78395	9,43E+05	0,00E+00	0,00E+00	0,00E+00	7,68E+05	0,00E+00	795,3991
EKSYELPDGQVITI	P83750	1591,82138	4,10E+05	0,00E+00	5,80E+04	0,00E+00	4,34E+05	0,00E+00	796,4164

LEEAEGTLEHEESK	Q90339	1600,73369	2,48E+05	9,83E+05	1,05E+05	4,63E+05	2,04E+05	7,38E+05	534,2518
EEAEGTLEHEESKI	Q90339	1600,73369	2,06E+06	0,00E+00	3,31E+05	0,00E+00	8,85E+05	6,25E+04	534,2510
PGPDGNPGEIGAPGPVGI	A0MSJ1	1600,79656	1,40E+06	1,77E+05	1,22E+06	1,77E+05	1,42E+06	2,49E+05	800,8962
KLDKENALDRAEQA	P13104	1600,82892	4,19E+05	0,00E+00	0,00E+00	0,00E+00	2,75E+05	0,00E+00	534,2826
AGGSGAAVGDEGEPGSKR	Q8UW00	1601,75140	0,00E+00	0,00E+00	0,00E+00	0,00E+00	0,00E+00	0,00E+00	801,3870
KLEQQVDDLEGSLE	Q90339	1602,78572	3,80E+06	1,04E+05	4,87E+06	1,25E+05	3,64E+06	3,82E+05	801,8970
VIGEHGDSSVPVWSGV	Q9W7K5	1624,79656	2,28E+06	0,00E+00	2,17E+06	0,00E+00	1,69E+06	0,00E+00	812,9042
KQEYDEAGPSIVHR	P53479	1628,80271	6,97E+06	3,15E+06	7,06E+06	4,50E+06	6,28E+06	3,73E+06	543,6072
SYELPDGQVITIGNE	P83750	1634,79081	8,90E+07	0,00E+00	5,67E+07	1,34E+06	6,11E+07	3,76E+05	817,9017
TALEEAEGTLEHEES	Q90339	1644,72352	4,93E+05	0,00E+00	1,98E+05	0,00E+00	3,45E+05	0,00E+00	822,8678
EKSYELPDGQVITIG	P83750	1648,84284	1,33E+06	0,00E+00	1,86E+05	0,00E+00	8,79E+05	0,00E+00	824,9272
NVLSGGTTMYPGIADR	P53479	1651,81083	3,02E+07	0,00E+00	1,02E+07	4,00E+05	1,31E+07	1,05E+05	826,4111
NVLSGGTTMYPGIADR	P53479	1667,80575	1,92E+06	0,00E+00	1,36E+06	3,15E+05	1,21E+06	4,47E+04	834,4093
ALEEAEGTLEHEESK	Q90339	1671,77080	1,32E+05	1,66E+05	1,06E+05	1,30E+05	1,74E+05	1,22E+05	836,3919
SKIEDEQSLGAQLQK	Q90339	1673,87045	1,40E+05	0,00E+00	0,00E+00	0,00E+00	1,73E+05	0,00E+00	837,4405
LILPENEPGSSIMPGK	Q7SX99	1681,88293	7,52E+05	0,00E+00	2,05E+05	0,00E+00	2,20E+05	0,00E+00	841,4470
DAGDGVTHNVPVYEGY	P53479	1692,75000	2,38E+06	5,99E+04	1,31E+06	1,26E+05	1,21E+06	6,40E+04	846,8798
GEHGDSSVPVWSGVNVA	Q9W7K5	1696,79254	6,87E+05	0,00E+00	1,00E+06	0,00E+00	6,10E+05	0,00E+00	848,9025
TKLEQQVDDLEGSLE	Q90339	1703,83340	2,07E+07	5,91E+04	6,58E+06	1,51E+05	1,67E+07	5,98E+04	852,4222
YELPDGQVITIGNER	P83750	1703,85989	3,12E+06	0,00E+00	7,98E+05	0,00E+00	1,20E+06	0,00E+00	852,4362
WVIGEHGDSSVPVWSG	Q9W7K5	1711,80746	5,73E+05	0,00E+00	3,16E+05	0,00E+00	3,40E+05	0,00E+00	856,4110
LEEAEGTLEHEESKI	Q90339	1713,81775	5,10E+05	0,00E+00	8,67E+04	0,00E+00	2,67E+05	0,00E+00	571,9451
KLDKENALDRAEQAE	P13104	1729,87152	2,09E+06	1,21E+05	3,73E+05	1,67E+05	1,18E+06	0,00E+00	577,2965
ESIPSSGRSTPAMMNGL	Q7SXV2	1734,81493	2,06E+06	7,19E+05	8,49E+05	2,82E+05	8,90E+05	4,36E+05	434,4597
VIGEHGDSSVPVWSGVN	Q9W7K5	1738,83949	3,60E+06	0,00E+00	2,46E+06	0,00E+00	2,12E+06	3,13E+04	869,9256
VFDKDGNGYISAAELR	Q6PI52	1754,87079	1,99E+06	0,00E+00	9,85E+04	0,00E+00	7,37E+05	0,00E+00	585,6300
KQEYDEAGPSIVHRK	P53479	1756,89767	1,54E+05	1,36E+05	2,06E+04	6,49E+04	9,97E+04	1,23E+05	439,9813
KSYELPDGQVITIGNE	P83750	1762,88577	1,20E+08	3,55E+05	5,19E+07	1,37E+06	9,31E+07	4,88E+05	881,9493
GWVIGEHGDSSVPVWSG	Q9W7K5	1768,82892	2,89E+05	0,00E+00	1,48E+05	0,00E+00	2,13E+05	0,00E+00	884,9188
TALEEAEGTLEHEESK	Q90339	1772,81848	2,42E+06	0,00E+00	1,10E+05	0,00E+00	1,56E+06	0,00E+00	591,6127
SYELPDGQVITIGNER	P83750	1790,89192	1,36E+07	6,34E+05	4,55E+06	2,06E+06	8,11E+06	4,95E+05	895,9515
EQFRQHMAATNNLVH	Q8JI10	1795,86566	0,00E+00	0,00E+00	8,99E+05	0,00E+00	0,00E+00	0,00E+00	599,2941
NVLSGGTTMYPGIADRM	P53479	1798,84623	2,24E+06	0,00E+00	9,20E+05	0,00E+00	1,45E+06	0,00E+00	899,9307

TIIDQNRDGIISKDDL	Q66I73	1815,94468	4,77E+06	0,00E+00	6,01E+05	0,00E+00	3,87E+06	0,00E+00	908,4799
EAFTIIDQNRDGIISK	Q66I73	1819,95485	3,77E+05	0,00E+00	9,60E+04	0,00E+00	1,62E+05	0,00E+00	607,3243
EAFSLFDKDGDGTITTK	Q6PI52	1844,89125	0,00E+00	0,00E+00	0,00E+00	0,00E+00	0,00E+00	0,00E+00	922,9515
AAAAAAAEAGSPWSSSPVGMA	P56224	1859,85924	3,14E+06	8,09E+05	5,80E+06	2,49E+05	3,14E+06	1,58E+06	930,4384
EKSYELPDGQVITIGNE	P83750	1891,92836	3,53E+06	0,00E+00	1,47E+05	0,00E+00	1,43E+06	0,00E+00	946,4714
KALDVDASGFIEEEELK	Q804W2	1892,94876	1,20E+06	0,00E+00	8,61E+04	0,00E+00	3,89E+05	0,00E+00	631,6560
KEWLQEAESLQVGGQVP	Q6IQX0-2	1897,96542	0,00E+00	0,00E+00	0,00E+00	0,00E+00	4,99E+05	0,00E+00	949,4938
KSYELPDGQVITIGNER	P83750	1918,98688	5,04E+06	0,00E+00	5,38E+05	0,00E+00	2,32E+06	0,00E+00	960,0002
WVIGEHGDSSVPVWSGVN	Q9W7K5	1924,91880	1,69E+06	0,00E+00	7,36E+05	0,00E+00	9,85E+05	0,00E+00	962,9669
GWVIGEHGDSSVPVWSGVN	Q9W7K5	1981,94027	7,91E+05	0,00E+00	3,44E+05	0,00E+00	4,54E+05	0,00E+00	991,4764
NLQQEISDLTEQLGETGK	Q90339	2002,99275	5,26E+04	0,00E+00	0,00E+00	0,00E+00	0,00E+00	0,00E+00	1002,0003
EISERLEEAGGATAAQIEM	Q90339	2004,95426	7,45E+05	0,00E+00	3,09E+05	0,00E+00	4,62E+05	0,00E+00	501,9932
HCVSQSSVTTTPPPEGDVQ	X1WE18	2025,91821	1,70E+06	0,00E+00	6,79E+05	0,00E+00	9,65E+05	0,00E+00	507,2359
FKWGESDPLPYAVYGHGI	B3DIV9	2035,99124	9,68E+04	0,00E+00	2,01E+05	0,00E+00	1,24E+05	0,00E+00	1018,4910
KNKTKNLWNPTYGSWVL	F1QVU0	2049,09162	1,94E+06	0,00E+00	3,19E+05	0,00E+00	1,10E+06	0,00E+00	513,0321
ESGSVGTGGRVCNRTSRGTD	Q92048	2052,94755	2,94E+06	0,00E+00	4,45E+05	0,00E+00	1,40E+06	0,00E+00	684,9934
LTNEASNFEFLPSAQRND	F1QB81	2052,96212	2,94E+06	0,00E+00	4,45E+05	0,00E+00	1,40E+06	0,00E+00	684,9935
PQRGGGGGGGGGMGRGRGRRDND	Q9DDT5	2200,02451	1,24E+06	1,49E+05	3,02E+05	0,00E+00	6,08E+05	1,44E+05	550,7564
EAVAGTVLSSLASPAHILSQQM	Q6IQE0	2210,14854	3,85E+05	0,00E+00	0,00E+00	0,00E+00	1,71E+05	0,00E+00	553,2941
TLQLLNGMGPLGRRASDGGANI	Q6NSM8	2211,16626	4,88E+05	0,00E+00	0,00E+00	0,00E+00	0,00E+00	0,00E+00	553,5455
AAAAAEAGSPWSSSPVGMAGSPQQ	P56224	2215,00842	1,02E+06	0,00E+00	2,41E+05	0,00E+00	5,00E+05	0,00E+00	739,0114
SPMHPGGGGQPQRGGGGGGGGGMGRG	Q9DDT5	2219,98937	8,15E+06	0,00E+00	3,12E+06	0,00E+00	4,85E+06	0,00E+00	740,6672
AFFVIDQDKSGFIEEDELK	Q9I8V0	2230,09141	1,38E+05	0,00E+00	0,00E+00	0,00E+00	0,00E+00	0,00E+00	744,0364
DIGVAMGIAGSDVSKQAADMILL	P25489	2291,16214	3,69E+06	0,00E+00	1,61E+06	0,00E+00	3,86E+06	0,00E+00	764,3909
KADVSSTGQSVIDKDALGPMML	Q98TW1	2295,12067	1,62E+05	0,00E+00	4,00E+05	7,15E+04	1,73E+05	0,00E+00	765,7056
PSEVSINQTPVEANEFPQLPE	A0A0R4IES7	2325,12450	8,89E+07	1,15E+06	1,72E+08	6,89E+06	1,11E+08	1,47E+06	775,7098
QPFCRGSPDPIDIISQNQPAS	Q9W7R4	2327,10847	2,96E+06	0,00E+00	2,67E+06	1,50E+05	2,10E+06	2,99E+04	776,3799
SFPTSVASNIYSSPVTTPLPDM	P26632	2327,11116	2,96E+06	0,00E+00	2,67E+06	1,50E+05	2,10E+06	2,99E+04	776,3799
YPFPTGGFPPPMQGAVNPWPGL	Q5XJD3	2327,13177	2,96E+06	0,00E+00	2,67E+06	1,50E+05	2,10E+06	2,99E+04	776,3798
PGLDGAAGKDGAKGMPGDLGRDGDV	A0MSJ1	2342,10411	1,84E+05	0,00E+00	3,91E+05	0,00E+00	2,62E+05	0,00E+00	781,3691
ISGAVHMVNTEIMQSTEGHGVR	A0A8M2BIB6	2353,13872	2,28E+05	0,00E+00	8,71E+04	0,00E+00	1,07E+05	0,00E+00	785,0480
KAFEIIDEDKSGFIEEEELK	P05939	2369,17586	0,00E+00	0,00E+00	0,00E+00	0,00E+00	0,00E+00	0,00E+00	790,4043
QTQAGSGSRYMPQQNSPVPSPY	F1QBY1	2396,09355	2,44E+05	0,00E+00	4,61E+05	0,00E+00	2,17E+05	0,00E+00	799,3734

LTSPNKEESSRGSAAASSAHTGKE	A0A0R4IC37	2402,15423	5,36E+06	5,03E+05	3,55E+05	1,68E+05	2,94E+06	2,10E+05	801,3904
KDYFIQKEKDCQKPDQLGPA	Q5U3J8	2408,19147	6,39E+06	4,55E+05	2,31E+06	5,25E+05	3,55E+06	8,94E+05	803,4014
WARSTGYDPIKLFNKLFKDD	Q7SXG4-2	2414,25031	3,79E+05	0,00E+00	2,68E+05	0,00E+00	3,14E+05	0,00E+00	805,4183
DELDLQLFNNESIAVTGGTSSGPG	F1QQA8	2421,14160	5,03E+05	4,65E+05	5,24E+05	5,83E+05	3,85E+05	4,52E+05	807,7191
GPPACRSSSRKGSSGNTSEVSVAC	Q2KN93	2438,11469	1,44E+06	0,00E+00	3,85E+05	0,00E+00	5,76E+05	0,00E+00	813,3762
GTPGAKGSSGSPGQMGPPGLPGPRGDM	A8WGB1	2450,15510	3,63E+06	4,71E+04	1,27E+06	7,66E+04	1,96E+06	2,72E+04	817,3942
LEDVQKPEQDLYRCVTQSSR	B3DK56	2451,19326	0,00E+00	0,00E+00	0,00E+00	0,00E+00	0,00E+00	0,00E+00	817,7424
KDANQVHSTTRRNSNSPLSPNQ	Q6NZT6	2451,20833	0,00E+00	0,00E+00	0,00E+00	0,00E+00	0,00E+00	0,00E+00	817,7426
ISQETNGGLPDSPIEFKGLPSPPA	H6D7E6	2451,24019	2,40E+06	0,00E+00	3,28E+06	0,00E+00	3,28E+06	4,30E+04	817,7435
HLGDSGHAPIDGIHAYTPQMHVS	Q6R005	2456,14117	9,04E+06	3,95E+05	4,00E+06	1,11E+06	4,16E+06	4,34E+05	819,3882
SGLSTPVSPTPAHLQHVREQMAV	X1WE18	2458,25072	4,43E+05	0,00E+00	0,00E+00	0,00E+00	1,50E+05	0,00E+00	820,0894
GTPGAKGSSGSPGQMGPPGLPGPRGDM	A8WGB1	2466,15002	7,83E+06	2,72E+04	2,87E+06	9,79E+04	4,02E+06	7,65E+04	822,7256
TPGAKGSSGSPGQMGPPGLPGPRGDMG	A8WGB1	2466,15002	7,83E+06	2,72E+04	2,87E+06	9,79E+04	4,02E+06	7,65E+04	822,7265
PGPEGPLQFPNQSSFPGGQVDGPY	Q67FY3	2472,14663	7,09E+06	7,97E+05	3,09E+06	1,30E+06	2,85E+06	6,95E+05	824,7211
GPNFSTTVGTSLQYSSSTYPSAKT	P26632	2481,17799	2,82E+07	2,85E+05	1,07E+07	9,53E+05	1,42E+07	3,00E+05	827,7302
LNDNPRFGMFQSDLILMINDAG	B3DIV9	2481,19009	2,82E+07	2,85E+05	1,07E+07	9,53E+05	1,42E+07	3,00E+05	827,7290
GLRDDLESISSVEAAFEDLEKAS	Q5PZ43	2481,19912	1,90E+06	0,00E+00	9,54E+05	0,00E+00	1,95E+06	0,00E+00	827,7438
PQSTPSSPQTELYAPVSPCPLPE	F1QQA8	2482,18063	2,04E+06	4,07E+04	4,55E+05	6,95E+04	8,91E+05	1,74E+04	828,0730
TVMWHIQGPNSNEVLPSMNTDI	Q6JAN0	2483,16936	3,27E+06	3,60E+04	1,15E+06	1,10E+05	1,58E+06	3,45E+04	828,4011
QPPMAPVGPPMGPMPPVGGMNPMAL	Q6DRG1	2487,17253	6,18E+07	1,68E+06	1,77E+08	1,75E+07	7,96E+07	2,92E+06	829,7281
LMGAAMVEPTVHNSDKLNGQDTQ	Q1LUT1	2488,14426	6,60E+05	8,79E+04	2,52E+06	4,80E+05	7,59E+05	6,27E+04	830,0570
EADIDGDGQVNYEEFVQMMTAK	Q6PI52	2490,07993	9,97E+04	0,00E+00	0,00E+00	0,00E+00	6,08E+04	0,00E+00	830,6999
SPSPVGYSPMTPGAPSPGGYNPHTPG	Q9DDT5	2525,14016	7,64E+05	0,00E+00	3,29E+05	0,00E+00	4,03E+05	1,62E+04	842,3841
TLDVAHNQLEHLPKEIGNCTQI	Q1L8Y7	2530,27185	9,23E+05	0,00E+00	0,00E+00	0,00E+00	0,00E+00	0,00E+00	633,3185
LSEPVGASIWGPSGSVDLSLEANAIA	Q8JGS1	2540,28787	0,00E+00	0,00E+00	0,00E+00	0,00E+00	0,00E+00	0,00E+00	847,4285
EKDVEFEVVGDAPDKAPAPSAPEEG	Q7SXG4-2	2583,20968	6,94E+05	7,08E+04	1,12E+06	3,57E+05	5,01E+05	0,00E+00	861,7346
AAQMKTGEVGRQLYELNMHTGVF	A0JMP0	2596,26465	7,26E+05	0,00E+00	4,64E+05	0,00E+00	4,05E+05	0,00E+00	866,0916
SYNYPPAGYPPQTPVAPGYSPGGAPP	Q503S1	2602,22488	2,18E+06	0,00E+00	8,07E+05	0,00E+00	8,77E+05	0,00E+00	868,0752
YPFPTGGFPPPMQGAVNPWPGLME	Q5XJD3	2619,20468	0,00E+00	0,00E+00	0,00E+00	0,00E+00	0,00E+00	0,00E+00	873,7416
FISPQMDPQIHQQPEMFQFDR	F1RE08	2619,21189	0,00E+00	0,00E+00	0,00E+00	0,00E+00	0,00E+00	0,00E+00	873,7416
SNKNYKNASGGGGGGGSSSPRGHGTANGS	Q2TLY1	2620,18430	4,90E+06	4,75E+04	2,18E+06	0,00E+00	0,00E+00	1,45E+05	874,0732
FGEPPVLEECLQKPGDEVNDIDK	Q6IQX0-2	2628,24977	2,49E+06	5,87E+04	1,80E+06	3,38E+05	1,53E+06	0,00E+00	876,7554
EMGLAGEKGDRGETGQPGPPGEKGAMG	A0MSJ1	2629,19809	1,47E+05	0,00E+00	0,00E+00	0,00E+00	0,00E+00	0,00E+00	877,0790

LPQRSAYGQGAGTGIPSSKPSSTGSIK	Q7SXT7	2632,36892	2,94E+05	5,84E+04	2,77E+05	0,00E+00	3,49E+05	1,07E+05	439,5717
TLGGAMIDGQSPFAANEPLNKAVGMN	E7F1H9	2635,24906	1,55E+06	0,00E+00	6,57E+05	2,15E+05	6,68E+05	6,23E+04	879,0820
DVYSQQELHINGDVILAFQQYY	A0JMP0	2643,27256	2,73E+06	0,00E+00	3,03E+06	0,00E+00	2,99E+06	0,00E+00	881,7607
NGPGLSPAPCLTTGPQHYGMAKQYV	A3KNJ3	2644,26465	3,22E+06	0,00E+00	9,23E+05	0,00E+00	1,50E+06	0,00E+00	882,0911
KSPVCYHSTPPHYSPSSGPCVPTI	Q8JGS1	2655,23302	1,86E+06	7,60E+05	4,77E+05	5,35E+05	8,99E+05	4,72E+05	885,7504
RQGEPTNVSSSSTTVQKASEMLQDL	H6D7E6	2693,30466	3,95E+06	0,00E+00	8,32E+05	0,00E+00	1,36E+06	3,88E+04	898,4465
GLSPWMWSLTLGCIVGSVWSSSPGL	A1XQY0	2693,31021	3,95E+06	0,00E+00	8,32E+05	0,00E+00	1,36E+06	3,88E+04	898,4465
PIRMEHVEPTATYVTEVPCIKQN	Q5BLC7	2728,34330	1,43E+06	0,00E+00	1,31E+06	2,03E+05	1,63E+06	4,49E+04	910,1207
AGEKGDRGEMGLPGPPGEKGSTGHPGTPG	C7DZK3	2730,29002	0,00E+00	0,00E+00	0,00E+00	0,00E+00	0,00E+00	0,00E+00	683,3308
MGGITVPAEPHPVLEKLRMVNNYNP	E7F1H9	2776,42730	0,00E+00	0,00E+00	0,00E+00	0,00E+00	0,00E+00	0,00E+00	694,8644
FQGQLSGLYYNGLKVLNMAAQGNPNI	A1XQY0	2810,42941	1,85E+06	0,00E+00	5,95E+05	0,00E+00	1,08E+06	0,00E+00	703,3655
GGGRGGGFNRGGGGGRGGGFGGGRGGGFGGGRGGG	Q7ZVE0	2837,32962	6,51E+05	0,00E+00	0,00E+00	0,00E+00	1,30E+05	0,00E+00	710,0860
GGGGGRGGGFNRGGGGGRGGGFGGGRGGGFGGGRG	Q7ZVE0	2837,32962	4,96E+05	0,00E+00	0,00E+00	0,00E+00	1,30E+05	0,00E+00	710,0860
ANGGGRGGFGGRGGGFGGRGGGGGGFRGGRGGGGG	Q7ZVE0	2851,34527	1,91E+06	9,76E+04	1,27E+05	0,00E+00	7,83E+05	0,00E+00	951,1212
NGVLGSDLMGAAMVEPTVHNSDKLNGQD	Q1LUT1	2869,34548	9,77E+05	0,00E+00	0,00E+00	0,00E+00	2,69E+05	0,00E+00	574,6771
VGPPQPPMAPVGPPMGPMPPVGGMNPMAL	Q6DRG1	2869,35777	9,77E+05	0,00E+00	0,00E+00	0,00E+00	2,69E+05	0,00E+00	574,6771
YKNASGGGGGGGSSSPRGHGTANGSVPSSSGPS	Q2TLY1	2875,29498	1,16E+06	0,00E+00	0,00E+00	0,00E+00	3,98E+05	0,00E+00	719,5823
SSPRGHGTANGSVPSSSGPSSSASSSSKGDR	Q2TLY1	2875,31611	1,16E+06	0,00E+00	0,00E+00	0,00E+00	3,98E+05	0,00E+00	719,5825
QSGLATHSSINRVLAGASIGDGQSAVASNI	X1WHY6	2881,47624	4,89E+05	0,00E+00	2,17E+05	0,00E+00	3,24E+05	0,00E+00	721,1257
LPDNPRSTFLLAFSPDRNLVASTHVN	X1WHY6	2881,49552	4,89E+05	0,00E+00	2,17E+05	0,00E+00	3,24E+05	0,00E+00	721,1248
VQYQAAIAASRASSVAPSPSLSAGPATQPV	Q6NYI0	2882,50066	0,00E+00	0,00E+00	0,00E+00	0,00E+00	0,00E+00	0,00E+00	721,3760
PPSSQPNSVSSGPTPSPGGFQPSPSPQPSQ	Q7ZVN7	2917,35987	0,00E+00	0,00E+00	1,48E+06	1,65E+05	0,00E+00	0,00E+00	973,1260
STPDGGPASSLISMSSAAALSSSSSPTASVNP	Q2KN93	2921,36805	7,41E+05	0,00E+00	0,00E+00	0,00E+00	4,12E+05	0,00E+00	585,0763
SLAMKEQQAAVTSSIMQAMRSAAGTPVPS	Q1LUT1	2964,45874	1,61E+06	0,00E+00	1,07E+05	0,00E+00	6,24E+05	0,00E+00	988,8308
GPATQPVQPNEPAAPMGPNPAPEDRPANPN	Q6NYI0	3031,43266	1,88E+06	4,21E+04	5,42E+05	0,00E+00	8,84E+05	6,31E+04	1011,1419
PGDSPSPEPGIPGPLHSMSSDVFGPSPSFTS	Q90476	3083,39387	8,36E+05	0,00E+00	3,51E+05	0,00E+00	5,19E+05	0,00E+00	617,4867
LNEMNDYAGQRELIAENMMINICVEL	Q5U3Q6	3083,43047	8,36E+05	0,00E+00	3,51E+05	0,00E+00	5,19E+05	0,00E+00	617,4862
